# Electrostatic Perturbations in the Substrate‐Binding Pocket of Taurine/α‐Ketoglutarate Dioxygenase Determine its Selectivity

**DOI:** 10.1002/chem.202104167

**Published:** 2022-01-22

**Authors:** Hafiz Saqib Ali, Sam P. de Visser

**Affiliations:** ^1^ Manchester Institute of Biotechnology The University of Manchester 131 Princess Street Manchester M1 7DN UK; ^2^ Department of Chemistry The University of Manchester Oxford Road Manchester M13 9PL UK; ^3^ Department of Chemical Engineering and Analytical Science The University of Manchester Oxford Road Manchester M13 9PL UK

**Keywords:** density functional theory, enzyme mechanism, hydrogen atom abstraction, inorganic reaction mechanisms, iron

## Abstract

Taurine/α‐ketoglutarate dioxygenase is an important enzyme that takes part in the cysteine catabolism process in the human body and selectively hydroxylates taurine at the C^1^‐position. Recent computational studies showed that in the gas‐phase the C^2^−H bond of taurine is substantially weaker than the C^1^−H bond, yet no evidence exists of 2‐hydroxytaurine products. To this end, a detailed computational study on the selectivity patterns in TauD was performed. The calculations show that the second‐coordination sphere and the protonation states of residues play a major role in guiding the enzyme to the right selectivity. Specifically, a single proton on an active site histidine residue can change the regioselectivity of the reaction through its electrostatic perturbations in the active site and effectively changes the C^1^−H and C^2^−H bond strengths of taurine. This is further emphasized by many polar and hydrogen bonding interactions of the protein cage in TauD with the substrate and the oxidant that weaken the *pro*‐*R* C^1^−H bond and triggers a chemoselective reaction process. The large cluster models reproduce the experimental free energy of activation excellently.

## Graphical abstract

**Density functional theory** calculations on large active site models of taurine/α‐ketoglutarate dioxygenase show that second‐coordination sphere effects of polar and charged amino acid residues are essential for the correct description of the regioselectivity of taurine activation by this enzyme. In particular, a charged histidine residue incurs a local electric field effect that guides the reactivity to C^1^‐hydroxylation.

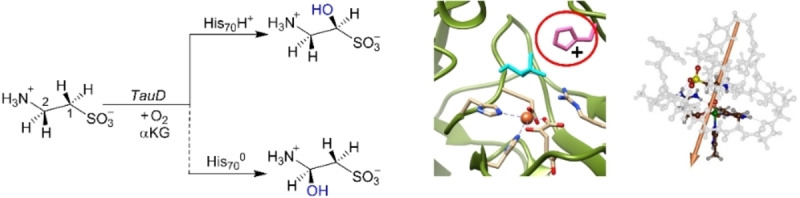

## Introduction

The nonheme iron dioxygenases belong to the large and most diverse class of mononuclear metalloenzymes that utilize molecular oxygen.^[1]^ In general, there are two types of oxygenases, namely those that utilize both atoms of molecular oxygen (O_2_) in substrate activation, i. e. the dioxygenases, and those that use only one oxygen atom of O_2_ to activate the substrate while the other oxygen atom is reduced to a water molecule, i. e. the monooxygenases.^[2]^ Some of these oxygenases, particularly the dioxygenases, often require a co‐substrate, such as α‐ketoglutarate (α‐KG) to generate the active species in the catalytic cycle that reacts with substrate.[^1^, ^3^] These so‐called α‐KG‐dependent dioxygenases generally contain a ferrous iron and react with α‐KG through oxidative decarboxylation to form succinate and with release of carbon dioxide produce a high‐valent iron(IV)‐oxo species. Many biomimetic iron(IV)‐oxo models have been created and studied and it was shown that they are highly reactive and often react with substrates through oxygen atom transfer.[^1d^, ^1g^, ^1h^, ^4^] The most common mechanism in nonheme iron dioxygenases is the activation of an aliphatic C−H bond that is then converted into an alcohol.[^1^, ^2^, ^3^, ^4^, ^5^] The α‐KG‐dependent nonheme iron dioxygenases are an important class of metalloenzymes which are present in almost all forms of life and carry out a range of important biochemical transformations. For instance, they are involved in biosynthesis reactions in the human body, including that of 4‐hydroxyproline, which is an essential component in collagen formation.^[6]^ In bacteria, the α‐KG‐dependent nonheme iron dioxygenases have been implicated with the biosynthesis of antibiotics,^[7]^ while in plants hormones such as flavonols are synthesized by a series of enzymes that includes several nonheme iron dioxygenases.^[5a]^

**Figure 1:**
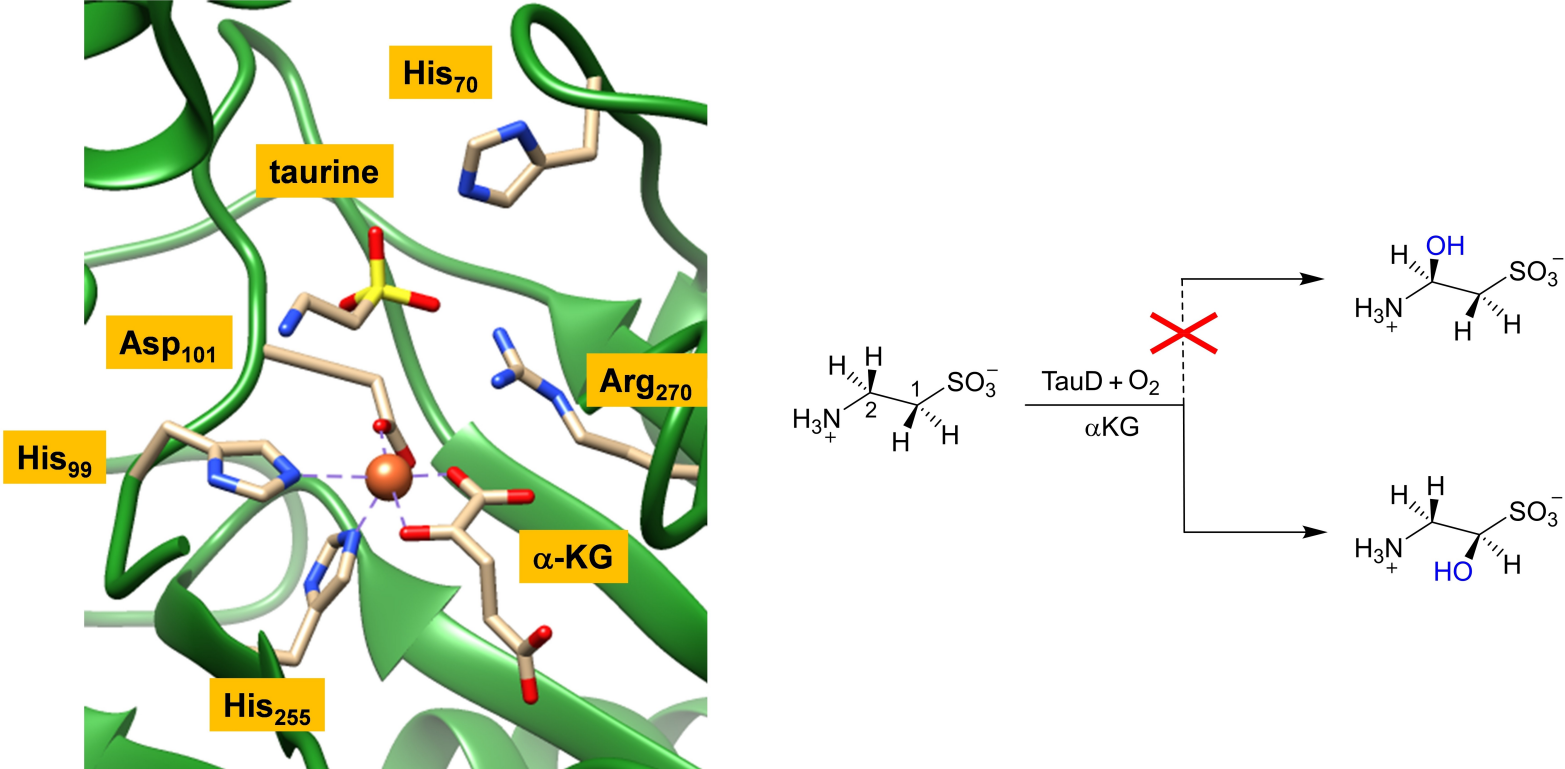
*Left*: Active site structure of TauD enzyme as taken from the 1OS7 pdb file. *Right*: General reaction mechanism of hydroxylation of taurine by TauD and products obtained.

Taurine/α‐KG‐dependent dioxygenase (TauD) is one of the best examples of an α‐KG‐dependent dioxygenase and has been extensively studied.[^1^, ^3^, ^5^] The human TauD isozyme takes part in the cysteine metabolism pathway, where it hydroxylates taurine selectively at the *pro*‐*R* C^1^‐position. In a subsequent reaction step hydroxytaurine splits off sulfate to trigger the sulfur recycling mechanism in the body.^[8]^ Figure 1 shows the extract of the active site of TauD alongside its general hydroxylation reaction mechanism. The TauD active site structure shown in Figure 1 was taken from the crystal structure coordinates deposited under the 1OS7 protein data bank (pdb) file.^[9]^ The central iron (Fe) atom in TauD is connected to the protein via covalent bonds with two histidine (His_99_ and His_255_) and the carboxylate group of an aspartic acid (Asp_101_) residue. In addition, the iron(II) binds α‐KG to give the iron a pentacoordinated ligand structure. Substrate taurine is bound close to the iron center and forms hydrogen bonding interactions with the side chains of His_70_ and Arg_270_. Experimental studies showed that TauD uses α‐KG and dioxygen to react on the iron(II) center to form an iron(IV)‐oxo active species.^[10]^ It was characterized for TauD using UV‐Vis absorption, Mössbauer spectroscopic, electron paramagnetic resonance and extended X‐ray absorption fine structure (EXAFS) studies.^[10]^ Moreover, kinetic isotope effect studies and the measurements of rate constants for substrate activation identified the iron(IV)‐oxo species as the active species of TauD.[^5c^, ^10a^] In particular, the iron(IV)‐oxo species was found to abstract a hydrogen atom from taurine at a rate of *k*_exp_=13 s^−1^ at 5 °C.

Many computational studies have subsequently focused on the catalytic cycle of TauD and the C^1^−H substrate activation pathways using density functional theory (DFT) cluster models as well as quantum mechanics/molecular mechanics methods (QM/MM).^[11]^ All of these studies, however, only considered *pro*‐*R* C^1^−H activation of taurine, even though taurine has four aliphatic C−H bonds in close proximity to the iron(IV)‐oxo active species that are chemically different. A recent report showed that in the gas‐phase the C^2^−H bond of taurine is considerably weaker than the C^1^−H bond.^[12]^ Based on these bond strengths, TauD should react through C^2^‐hydroxylation rather than C^1^‐hydroxylation, which it does not do. Therefore, TauD reacts through negative catalysis, whereby a thermodynamically unfavorable channel is catalyzed over one that has a larger driving force.[^12^, ^13^] The question is how TauD manages this negative catalysis and how the thermodynamically favorable C^2^−H activation channel is blocked.

To find out why and how TauD reacts regio‐ and stereoselectively on the *pro*‐*R* C^1^‐position of taurine, and whether alternative pathways are feasible, we decided to do a computational study on the substrate hydroxylation of taurine at the *pro*‐*R* C^1^−H, *pro*‐*S* C^1^−H, *pro*‐*R* C^2^−H and *pro*‐*S* C^2^−H positions using a combination of density functional theory (DFT) on cluster models and QM/MM on complete enzymatic structures. Furthermore, we investigate how electrostatic perturbations of, for example, the protein, can trigger a selective reaction process through studying residues in different protonation states. Our work shows that electrostatic perturbations of charged residues in the second‐coordination sphere of the protein guide an otherwise unselective reaction process to a regio‐ and stereoselective reaction mechanism. In particular, the protonated histidine group in the substrate binding pocket is vital to direct the local dipole moment in such a way to guide the reaction to C^1^‐hydroxylation.

## Results and Discussion

**Figure 2:**
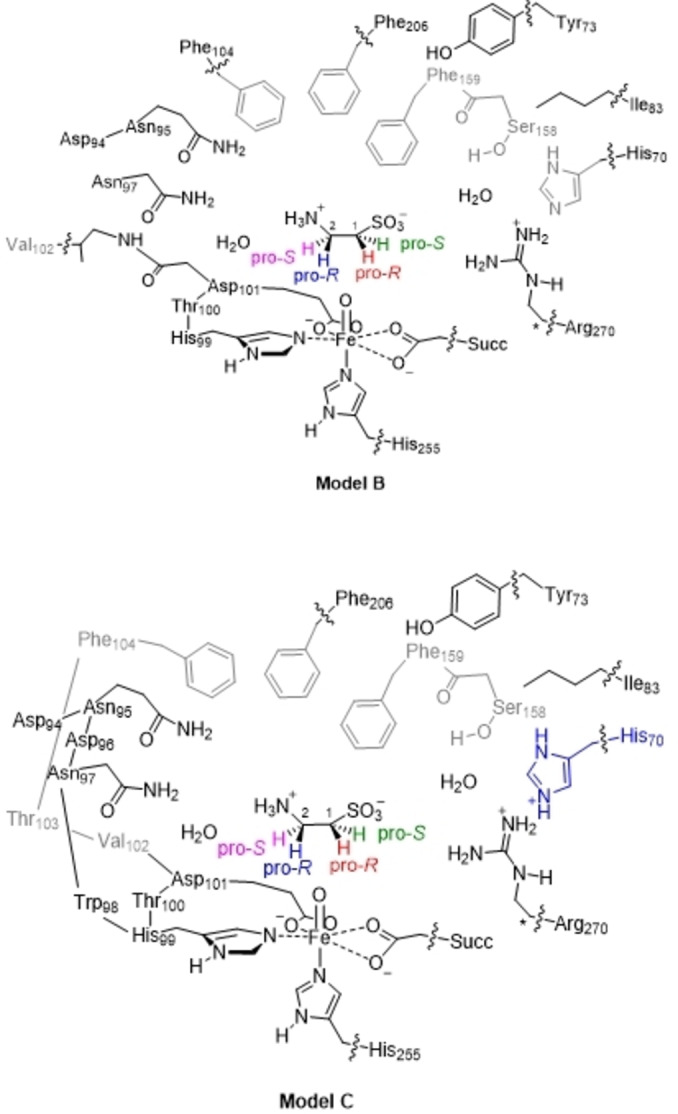
DFT cluster models **B** and **C** used in this work. Wiggly lines show where bonds were cut and link‐atoms were added. Atoms labelled with a star were kept fixed during the geometry optimizations.

To gain mechanistic insight into the substrate selectivity process of taurine hydroxylation by TauD enzymes, we performed a series of DFT and QM/MM calculations. As shown by kinetic isotope effect measurements on the enzyme, the taurine activation is performed by an iron(IV)‐oxo species.^[10a]^ Therefore, we created the corresponding iron(IV)‐oxo species (**Re**) using quantum chemical cluster models but also performed QM/MM on the full structure. The models were based on the 1OS7 pdb file^[9]^ and after initial set‐up that included a molecular dynamics (MD) simulation (see details in the Methods section and Supporting Information), we established that the protein structure and substrate placement are highly rigid. We initially created three cluster models based on the last step of the MD simulation, namely a minimal model **A** and two larger cluster models **B** and **C** (Figure 2). Data on the small cluster model **A** can be found in the Supporting Information, while we focus on the larger cluster models only. The large cluster models had 244 (model **B**) and 279 (model **C**) atoms in total and contain the iron(IV)‐oxo and substrate with their first‐ and second coordination sphere included. In particular, several protein chains were part of the cluster models, whereby the amino acid residues of those chains that are pointing into the active site were included in the model, while all other residues from the chosen chains were truncated to Gly residues. The cluster models, therefore, include the hydrogen bonding network around the substrate and oxidant that positions these groups in the active site. In model **B** the His_70_ side chain is singly protonated while it is doubly protonated in model **C**. For optimal comparison, we did some test calculations, whereby we took the model **C** structures and removed the proton from His_70_: Model **C2**.

**Figure 3:**
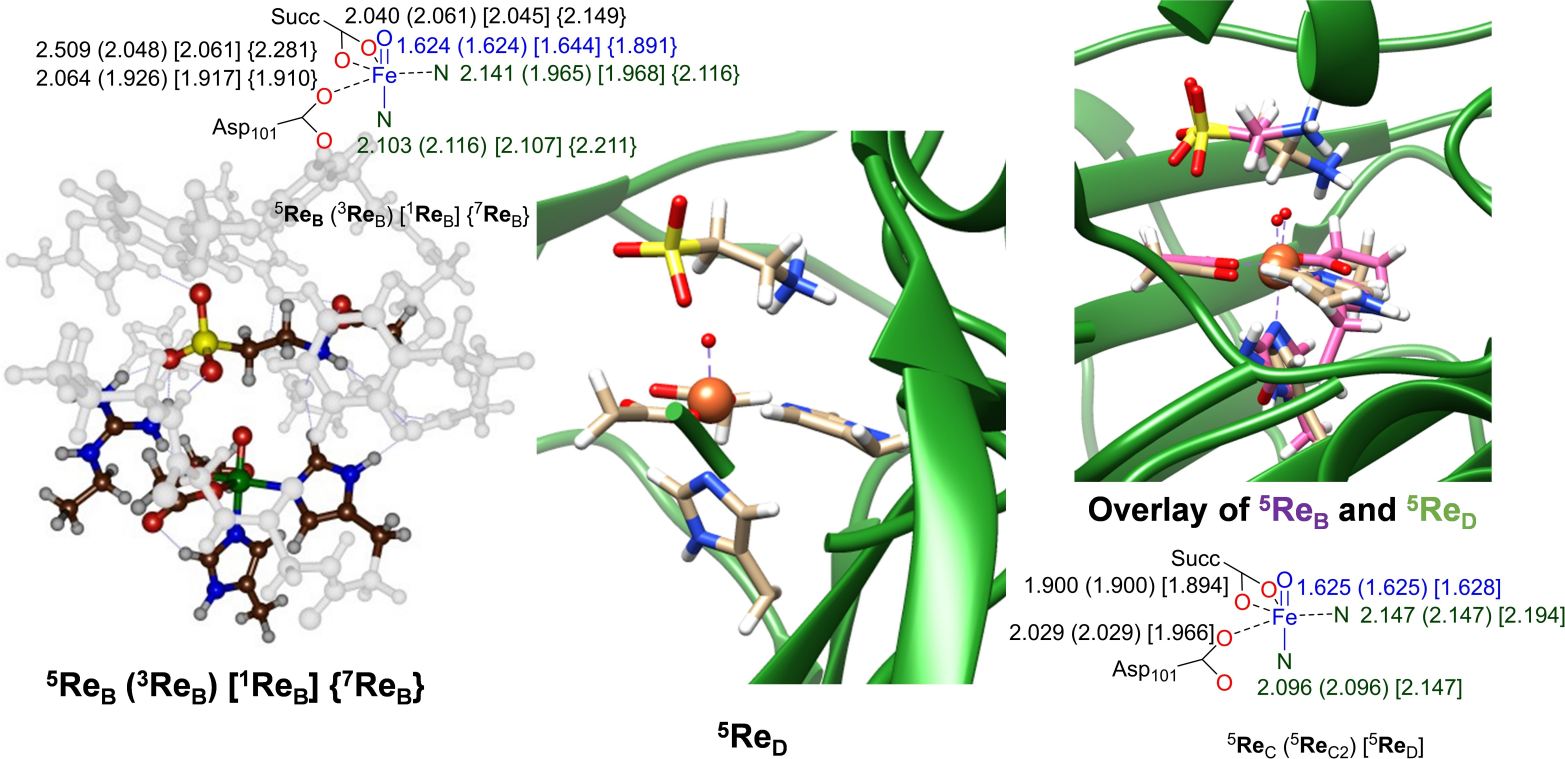
Optimized geometries of iron(IV)‐oxo complexes calculated in this work. Left hand‐side: DFT cluster results of ^1,3,5,7^ **Re**_B_. Middle and bottom‐right: Optimized geometries of ^5^ **Re**_C_, ^5^ **Re**_C2_ and ^5^ **Re**_D_. Right‐hand‐side: overlay of the ^5^ **Re**_B_ and ^5^ **Re**_D_ optimized geometries. Bond lengths are given in angstroms.

Subsequently, a geometry optimization using four DFT cluster models (**Re**_A_, **Re**_B_, **Re**_C_ and **Re**_C2_) and one QM/MM model **Re**_D_ was performed. A comparison of the optimized geometries of **Re**_B_, **Re**_C_, **Re**_C2_ and **Re**_D_ are given in Figure 3. The cluster models **Re**_B_ were calculated in multiple spin states. The quintet spin state ^5^ **Re**_B_ is the ground state with the triplet spin state (^3^ **Re**_B_) higher in energy by 10.4 kcal mol^−1^ at UB3LYP‐D3/BS2//UB3LYP/BS1+ZPE+E_solv_ level of theory with ZPE=zero‐point energy and E_solv_=solvation energy, while the singlet and septet structures are higher in energy by 19.2 and 10.9 kcal mol^−1^. Our calculated electronic configuration of the iron(IV)‐oxo species, therefore, is in agreement with electron paramagnetic resonance (EPR) studies that characterized the iron(IV)‐oxo species as a quintet spin state.^[10a]^ Geometrically, the ^5^ **Re**_B_ structure has the carboxylate group of succinate bound as a bidentate ligand with Fe−O distances of 2.040 and 2.509 Å. Similar distances are seen for the interactions of the carboxylate group of the Asp_101_ ligand with iron, while the two nitrogen ligands (of His_99_ and His_255_) are at an Fe−N distance of 2.141 and 2.103 Å. The iron(IV)‐oxo bond is short at 1.624 Å. This value is in excellent agreement with experimental EXAFS studies that identified an Fe−O distance of 1.62 Å.^[10c]^ The singlet and triplet geometries similarly to the quintet spin state have short Fe−O distances of 1.644 and 1.624 Å, while the septet spin state has the distance elongated to 1.891 Å. The iron first‐coordination sphere distances in models **C** and **C2** are virtually identical; hence addition of a second‐coordination sphere proton does not affect the electronic configuration and geometry of the iron center. Using model **C** and QM/MM we find short Fe−O distances of 1.625 and 1.628 Å, while the Fe−N distances are 2.147/2.194 (equatorial) and 2.096/2.147 (axial) Å, respectively. As such, structures ^5^ **Re**_B_, ^5^ **Re**_C_ and ^5^ **Re**_D_ have a similar first‐coordination sphere. Indeed, an overlay of the optimized geometries of ^5^ **Re**_B_ and ^5^ **Re**_D_ (right‐hand‐side of Figure 3) puts most atoms in a similar position and shows that the first‐coordination sphere of the two models as well as the protein chains are matching well. Our optimized geometries also compare well to previous DFT and QM/MM calculations on the iron(IV)‐oxo species of TauD as well as analogous αKG‐dependent nonheme iron dioxygenases.[^11^, ^14^]

The resonance Raman difference spectrum generated of TauD in a reaction with ^16^O_2_ gave a fingerprint for the iron(IV)‐oxo stretch vibration at 821 cm^−1^ that dropped to 787 cm^−1^ when the heavy isotope ^18^O_2_ was used.[^10b^, ^15^] A geometry optimization of ^**5**^ **Re**_B_ using a modest LANL2DZ basis set with core potential on iron and 6‐31G on the rest of the atoms reproduces the experimental vibrational spectrum well and gives an Fe−O bond of 1.655 Å and an Fe−O stretch vibration of 817 cm^−1^. At the UB3LYP/BS1 level of theory the calculated vibrational frequencies of ^5^ **Re**_B_ for Fe=^16^O versus Fe=^18^O gives a drop of the iron(IV)‐oxo stretch vibration by 38 cm^−1^ using the recommended scaling factor of 0.95 of Scott and Radom.^[16]^ The calculated vibrational frequency difference is in good agreement with the difference spectrum measured by Proshlyakov et al.^[15]^ As such, we decided to continue our studies with UB3LYP/BS1 as it matches the experimental structure the best. In particular, our calculated optimized geometry, vibrational frequencies and electronic configuration of the quintet spin state excellently reproduces experimental characterization.

Iron(IV)‐oxo complexes have close‐lying spin states as a result of their orbital occupation, which is dependent on their ligand features. Thus, nonheme iron(IV)‐oxo species in octahedral ligand environment typically have a triplet spin ground state with the quintet spin higher in energy.^[17]^ By contrast, trigonal bipyramidal coordinated iron(IV)‐oxo is often found in a quintet spin ground state. Furthermore, the effect of solvation and external perturbations has been shown to affect spin‐state ordering and reactivities dramatically.^[18]^ The ordering of the spin states in the reactants and local minima along the reaction mechanism may affect the kinetics and as such all structures were calculated in multiple spin states.^[19]^

**Figure 4:**
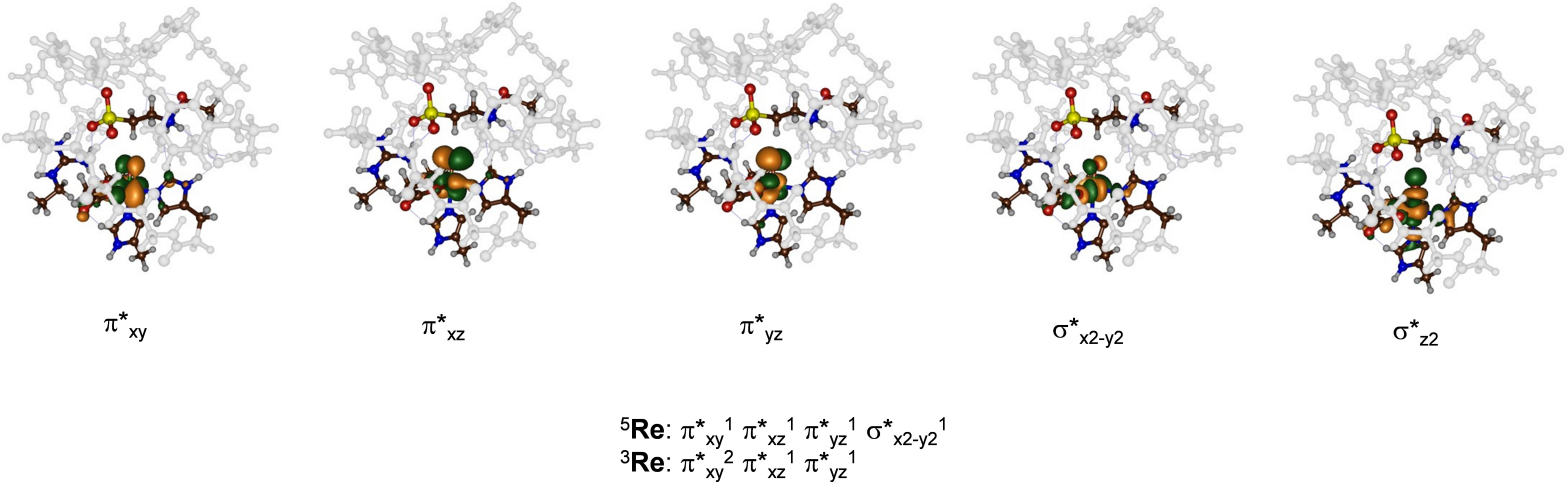
Molecular valence orbitals of the iron(IV)‐oxo species of TauD.

Figure 4 displays the high‐lying occupied and virtual molecular orbitals that determine the spin state ordering in the reactants complex. All contain a metal 3d‐contribution and hence are labelled with the 3d‐type. These orbitals are the π*_xy_, π*_xz_, π*_yz_, σ*_x2‐y2_ and σ*_z2_. The lowest in energy are the three π* orbitals for the antibonding interactions of the metal 3d orbitals with the 2p_x_ and 2p_y_ atomic orbitals on the oxo group along the z‐axis (π*_xz_ and π*_yz_), while the π*_xy_ orbital is in the equatorial plane. In addition, there is the σ*_x2‐y2_ orbital for the antibonding interactions of the 3d_x2‐y2_ orbital on iron with σ‐orbitals in the xy‐plane from the nitrogen of His_99_ and the oxygen atoms of Asp_101_ and succinate. Finally, the σ*_z2_ orbital along the z‐axis is based on the interactions of the 3d_z2_ on iron with the 2p_z_ on the oxo group and the 2p_z_ on the nitrogen atom of the axial histidine ligand (His_255_). In the quintet spin ground state the structure has electronic configuration of π*_xy_^1^ π*_xz_^1^ π*_yz_^1^ σ*_x2‐y2_^1^, while the σ*_z2_ orbital is virtual. By contrast, the triplet spin state has configuration π*_xy_^2^ π*_xz_^1^ π*_yz_^1^. The septet spin state can be reached from the quintet spin state by a promotion of an electron from the doubly occupied π_O_ orbital on the oxo group to σ*_z2_. For our TauD model the optimized singlet and septet spin state structures are high in energy and hence the singlet and septet spin states were not considered further.

### DFT cluster model B calculations on the stereoselectivity of taurine hydroxylation

**Figure 5:**
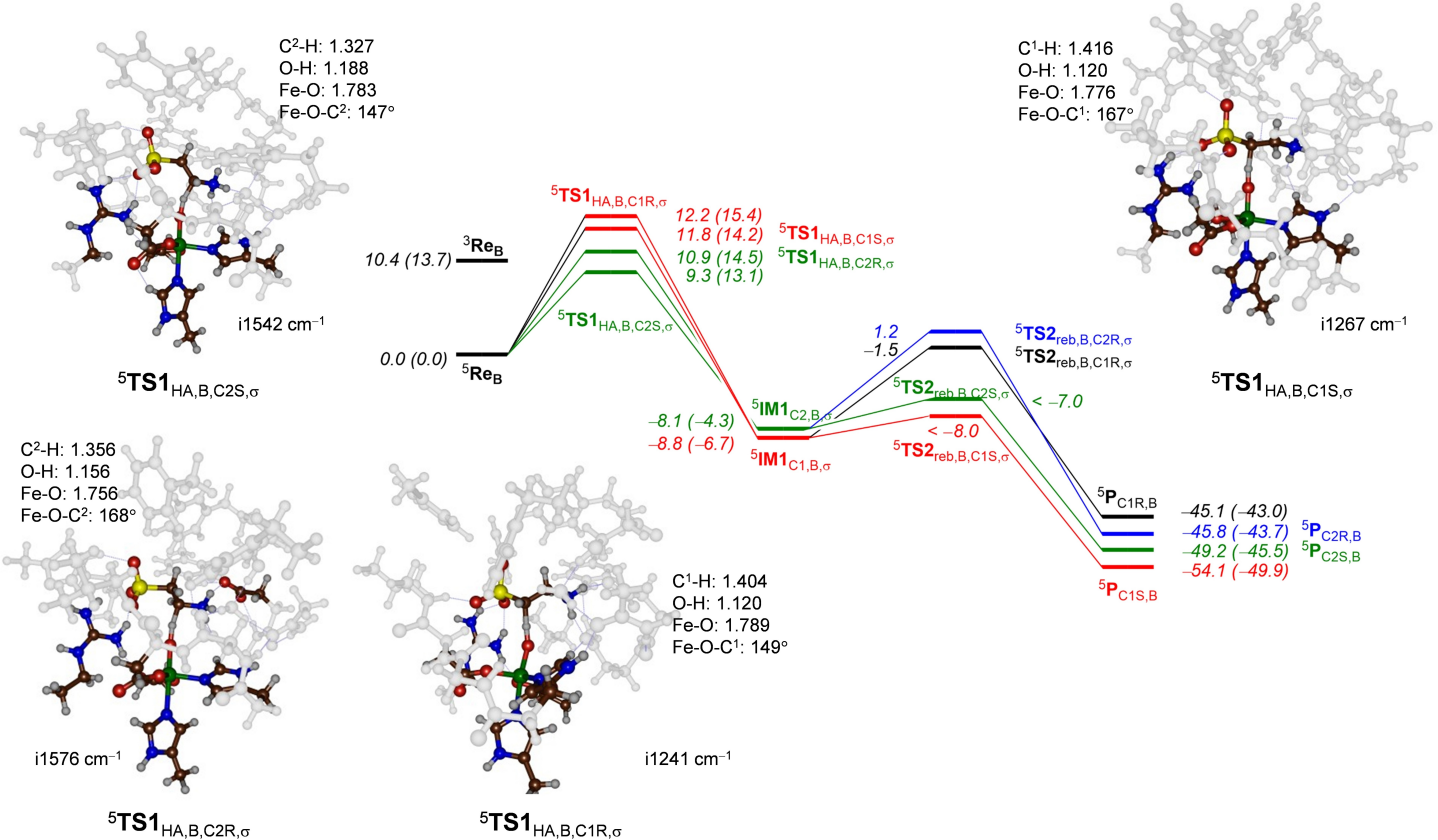
UB3LYP‐D3/BS2//UB3LYP/BS1 calculated potential energy landscape for taurine hydroxylation at the *pro*‐*R* and *pro*‐*S* C^1^−H and C^2^−H positions of taurine in ^5^ **Re**_B_ as calculated in Gaussian‐09. Energies (in kcal mol^−1^) contain solvent, dispersion and ZPE corrections, while free energies are given in parenthesis. Optimized geometries of the hydrogen atom abstraction transition states give bond lengths in angstroms, angles in degrees in the imaginary frequency in cm^−1^.

Next, we considered taurine hydroxylation at the C^1^ and C^2^ positions of the substrate which we investigated for the cluster model **B**. Thus, we studied the activation of all four C−H bonds of taurine, namely the *pro*‐*R* as well as the *pro*‐*S* sites of the substrate. These C−H bonds are defined as C1R, C1S, C2R and C2S and give hydroxytaurine with the corresponding stereochemistry. We calculated a stepwise mechanism with an initial hydrogen atom abstraction leading to a radical intermediate (**IM1**) via a transition state **TS1**_HA_. In a subsequent OH rebound step via transition state **TS2**_reb_ the hydroxytaurine product complexes (**P**_H_) are reached. Details of the mechanism and the nomenclature of the structures are given in Scheme S1 (Supporting Information). The potential energy landscape for taurine hydroxylation leading to *R*‐1‐hydroxytaurine, *S*‐1‐hydroxytaurine, *R*‐2‐hydroxytaurine and *S*‐2‐hydroxytaurine for model ^5^ **Re**_B_ is shown in Figure 5. In agreement with previous computational studies and experimental observation,^[10a]^ the rate‐determining step is the hydrogen atom abstraction via ^5^ **TS1**_HA_. Interestingly, we find all four hydrogen atom abstraction barriers within a window of 3.0 kcal mol^−1^. In particular, the lowest energy barrier is for the *pro*‐*S* C^2^−H hydrogen atom abstraction (^5^ **TS1**_HA,B,C2S,σ_) with a magnitude of 9.3 kcal mol^−1^. About 1.6 kcal mol^−1^ higher in energy, is the barrier passing ^5^ **TS1**_HA,B,C2R,σ_, which leads to the same radical intermediate ^5^ **IM1**_C2,B_ as the transition state ^5^ **TS1**_HA,B,C2S,σ_. The hydrogen atom abstraction steps from the C^2^‐position to reach ^5^ **IM1**_C2,B_ is exothermic by ΔE+ZPE=−8.1 kcal mol^−1^ with respect to ^5^ **Re**_B_, while the radical intermediate at C^1^ (^5^ **IM1**_C1,B_) is −8.8 kcal mol^−1^ more stable. The lowest energy C^1^−H hydrogen atom abstraction barrier is from the *pro*‐*S* C^1^−H group and has a barrier of 11.8 kcal mol^−1^, while the *pro*‐*R* hydrogen abstraction is at 12.2 kcal mol^−1^ with respect to ^5^ **Re**_B_. As such the calculations predict dominant C^2^‐hydroxylation of taurine, although some small amount of C^1^‐hydroxylation could be possible. The results of the calculations on model **B**, therefore, are in disagreement with the natural system as only C^1^‐hydroxylation products are observed. Therefore, alternative models and methods were applied that investigated this discrepancy.

The optimized transition state structures are given in Figure 5 as well and show the typical features of hydrogen atom abstraction transition states by nonheme iron(IV)‐oxo complexes.[^17^, ^20^] In all structures the Fe−O bond has elongated to 1.78 Å in the transition state and the substrate approaches the oxidant under a large Fe−O−C angle that ranges from 147° for ^5^ **TS1**_HA,B,C2S,σ_ to 167° for ^5^ **TS1**_HA,B,C1S,σ_. In general, all structures are product‐like with a short O−H and long C−H distances. For all four hydrogen atom abstraction pathways, a radical intermediate with electronic configuration π*_xy_^1^ π*_xz_^1^ π*_yz_^1^ σ*_x2‐y2_^1^ σ*_z2_^1^ ϕ_Sub_^1^ is formed, whereby the substrate orbital (ϕ_Sub_) contains a down‐spin electron, while the other orbitals have a single up‐spin electron. In previous studies this pathway was called the ^5^σ‐pathway and it often shows attack of substrate from the top as the σ*_z2_ orbital is being filled.^[21]^ Structures of hydrogen atom abstraction transition states, radical intermediates and rebound transition states with this electronic configuration have the subscript σ added to the label.

The alternative electron transfer during the hydrogen atom abstraction leads to radical intermediates **IM1**_C1,B,π_ and **IM1**_C2,B,π_ with configuration π*_xy_^2^ π*_xz_^1^ π*_yz_^1^ σ*_x2‐y2_^1^ σ*_z2_^0^ ϕ_Sub_^1^, whereby the substrate radical is up‐spin and ferromagnetically coupled to the metal 3d unpaired electrons. The latter configuration is called the ^5^π‐pathway configuration and usually has a transition state structure with more bent Fe−O−C angle (typically around 120°).^[21]^ Although we were unable to characterize any ^5^π‐type hydrogen atom abstraction transition states, we were able to find the radical intermediates ^5^ **IM1**_C1,B,π_ and ^5^ **IM1**_C2,B,π_ that have a ^5^π‐type electronic configuration. These two radical intermediates are well higher in energy than the ^5^ **IM1**_C1,B,σ_ and ^5^ **IM1**_C2,B,σ_ structures shown in Figure 5 by 16.2 and 15.6 kcal mol^−1^, respectively. Therefore, the ^5^π‐pathway is a high energy pathway that is inaccessible for our TauD model studied here. These pathways also have substantial rebound barriers of well over 12 kcal mol^−1^ with respect to the reactants complex (Supporting Information, Table S6) and hence the ^5^π‐pathway will not be able to compete with the ^5^σ‐pathway for this oxidant.

Despite the fact that the lowest energy barriers correspond to C2S and C2R hydrogen atom abstraction pathways, the lowest energy intermediate is the C^1^‐radical although by only a small margin (ΔE+ZPE=0.7 kcal mol^−1^). Clearly, the substrate binding pocket and the protein environment bind and position the substrate in a specific orientation to guide the reaction towards a specific regio‐ and stereoselectivity. After the radical intermediates a small rebound barrier for the C1S and C2S pathways leads to hydroxytaurine product complexes, while the C1R and C2R pathways have considerable rebound barriers of 7.3 and 9.3 kcal mol^−1^ as estimated from constraint geometry scans. The rebound steps have large exothermicity and generate products within a small margin of energy with the most stable product complex to be the *S*‐1‐hydroxytaurine product. All hydrogen atom abstraction transition states calculated for model **B** have a large imaginary frequency (>i1000 cm^−1^) and hence the reaction is expected to proceed with a large amount of quantum chemical tunneling and will be affected by the substitution of a hydrogen atom by deuterium.

We repeated several of the geometry optimizations for the hydrogen atom abstraction step with different density functional theory methods and basis sets, however, all these calculations (see Supporting Information) predicted the same product distributions and did not change the order of the transition states.

### DFT cluster model C calculations on the regioselectivity of taurine hydroxylation

**Figure 6:**
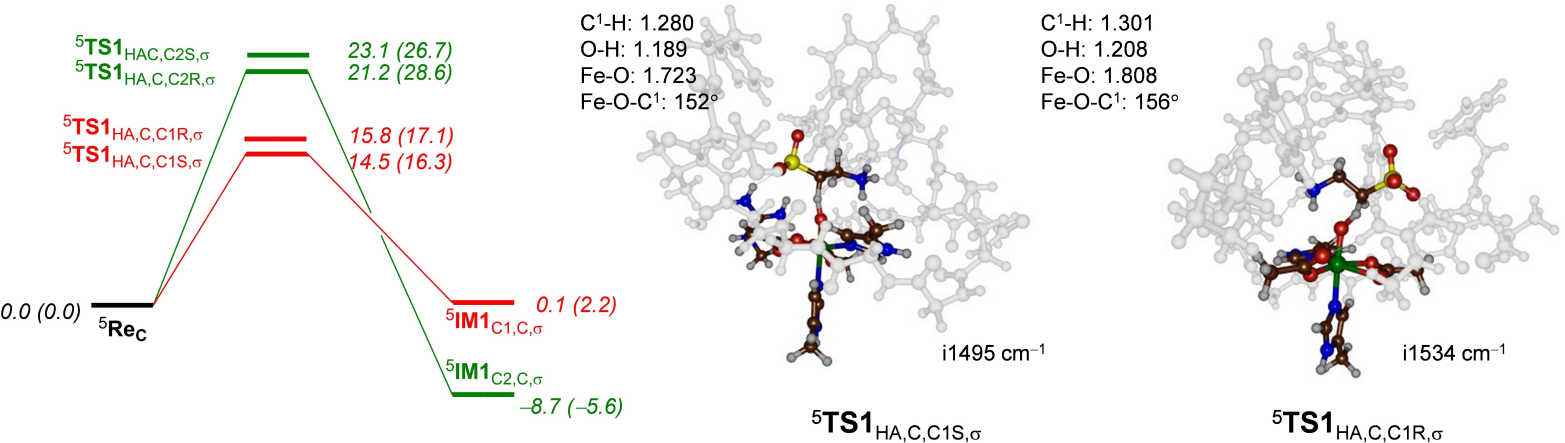
UB3LYP‐D3/BS2//UB3LYP/BS1 calculated potential energy landscape for taurine hydroxylation at the *pro*‐*R* and *pro*‐*S* C^1^−H and C^2^−H positions of taurine in ^5^ **Re**_C_ as calculated in Gaussian‐09. Energies (in kcal mol^−1^) contain solvent, dispersion and ZPE corrections, while free energies are given in parenthesis. Optimized geometries of the hydrogen atom abstraction transition state give bond lengths in angstroms, angles in degrees in the imaginary frequency in cm^−1^.

As the DFT cluster calculations on Model **B** predict the wrong product distributions as compared to experiment, we decided to expand the cluster structure to model **C** that has most of the protein chains around substrate and oxidant included and therefore restricts substrate approach. Moreover, the model has the active site His_70_ residue in its doubly protonated form. Figure 6 gives the hydrogen atom abstraction energy landscape for the pathways for activation of the C1R, C1S, C2S and C2R positions of taurine as calculated with model **C**. As can be seen the C^1^−H hydrogen atom abstraction barriers are by far the lowest at ΔE+ZPE=14.5 and 15.8 kcal mol^−1^, while the two transition states for C^2^−H hydrogen atom abstraction are well higher at 21.2 and 23.1 kcal mol^−1^. As such, the model **C** calculations predict dominant C^1^‐hydroxylation of taurine in agreement with experimental observation. As the margins between the C^1^−H and C^2^−H hydrogen atom abstraction barriers are large, the probability of obtaining C^2^‐hydroxylated products appears negligible. Interestingly, the radical intermediate ^5^ **IM1**_C2,C,σ_ is more stable than ^5^ **IM1**_C1,C,σ_ by almost 9 kcal mol^−1^. Therefore, the order of the radical intermediates and transition states is opposite and TauD appears to react by negative catalysis, where a thermodynamically less favorable pathway is kinetically favored.

The optimized geometry of the ^5^ **TS1**_HA,C,C1S,σ_ transition state is shown on the right‐hand‐side of Figure 6. It has a large imaginary frequency of i1495 cm^−1^ that indicates the reaction will proceed with a significant amount of quantum tunneling and will have a large kinetic isotope effect. The geometry has a relatively central hydrogen atom at a distance of the donor carbon atom of 1.280 Å and at a distance of 1.189 Å to the acceptor oxo group. The C^1^−H−O angle is 152°. The *pro*‐*R* hydrogen atom abstraction transition state also has a large imaginary frequency of i1534 cm^−1^ and consequently, will experience a large kinetic isotope effect. The structure is relatively central with similar O−H and C−H distances, i. e. 1.208 and 1.301 Å, respectively. After the formation of the C^1^‐radical (^5^ **IM1**_C1,C,σ_) a favorable rebound pathway with a negligible barrier leads to *R*‐1‐hydroxytaurine products selectively in agreement with experimental observation. It appears, therefore, that large cluster models that incorporate a significant number of atoms of the second coordination sphere in TauD and particularly a charged His_70_ residue give a good representation of the actual enzyme. To test the effect of the proton on the His_70_ residue, we created model **C2** that is model **C** without the proton on His_70_. Subsequently, the reactants and hydrogen atom abstraction transition states were reoptimized. Indeed, we see a change in regioselectivity in model **C2** as compared to model **C**. In particular, the lowest hydrogen atom abstraction barriers for model **C2** are the C^2^−H hydrogen atom abstraction barriers by more than 3 kcal mol^−1^ over the C^1^−H barriers. Therefore, the choice of the model in TauD and particularly the inclusion of protonated groups in the substrate binding pocket such as His_70_ are essential for the proper description of the long‐range electrostatic effects of the active site and influence barrier heights and selectivities.

### QM/MM calculations on the stereoselectivity of taurine hydroxylation

**Figure 7:**
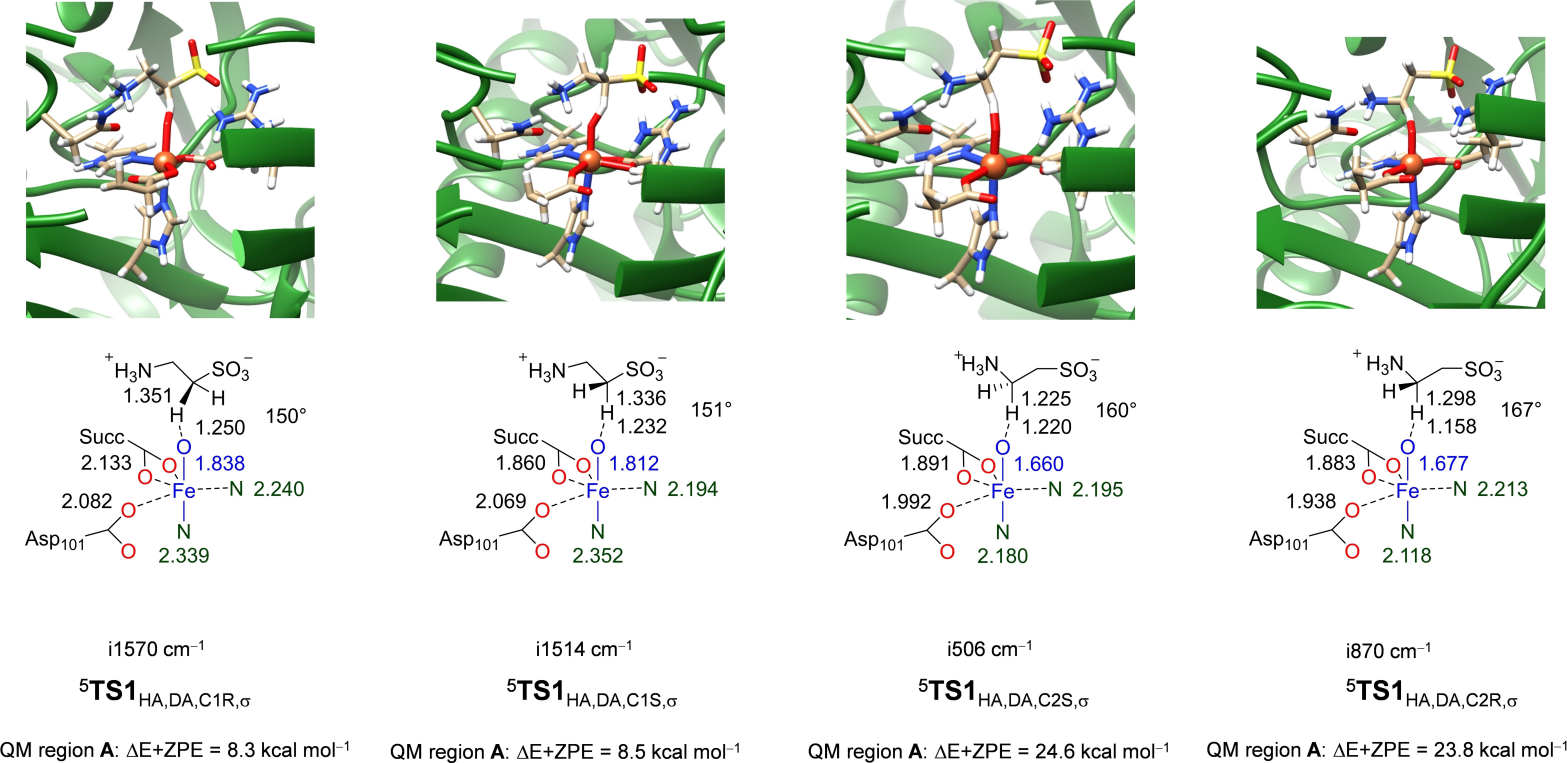
QM/MM optimized transition state geometries using QM region **A** calculated at ONIOM(UB3LYP‐D3/BS1:Amber) level of theory. Relative energies include ZPE and were obtained with basis set BS2 relative to ^5^ **Re**_D_. Bond lengths are in angstroms and the C−H−O angles in degrees.

In addition to the DFT cluster calculations, we also decided to study the structure and reactivity of TauD with QM/MM methods on the full protein. Figure 7 gives the hydrogen atom abstraction transition state structures for the C1R, C1S, C2S and C2R pathways of TauD as calculated with QM/MM using model **D** and minimal QM region **A**. In agreement with experimental observation the QM/MM calculated reaction mechanisms give the ^5^ **TS1**_HA,DA,C1R,σ_ barrier as the lowest in energy. In particular, the ^5^ **TS1**_HA,DA,C1R,σ_ and ^5^ **TS1**_HA,DA,C1S,σ_ barriers are at ΔE+ZPE=8.3 and 8.5 kcal mol^−1^ with respect to the iron(IV)‐oxo reactant complex and are considerably lower in energy than the ones for the C^2^−H hydrogen atom abstractions, namely ΔE+ZPE=24.6 kcal mol^−1^ for ^5^ **TS1**_HA,DA,C2S,σ_ and ΔE+ZPE=23.8 kcal mol^−1^ for ^5^ **TS1**_HA,DA,C2R,σ_. These energetic differences implicate that the reaction will be highly selective and virtually no C^2^‐hydroxylation will happen by the enzyme. As such, the QM/MM calculations predict the correct selectivity as determined experimentally and give a reversal of selectivity as compared to the cluster models mentioned above. A previous QM/MM study on TauD on a considerably smaller enzyme model reported a hydrogen atom abstraction barrier of ΔE+ZPE=6.7 kcal mol^−1^ for the *pro*‐*R*‐C^1^−H abstraction reaction based on constraint geometry scans, which is a result that is in good agreement with what we find here.^[11e]^ To find out if the choice of the QM region affects the energetics of the calculation, we expanded the QM region to the same atoms as described in cluster model **B** and did a single point QM/MM calculation at ONIOM(UB3LYP‐D3/BS1 : Amber) level of theory. However, the choice of the QM region has a dramatic effect on the energetics and reverses the ordering of the transition states and predicts selective C^2^‐hydroxylation rather than C^1^‐hydroxylation. This is opposite of experimental observation and therefore the choice of the QM region and the protonation state of the second‐coordination sphere residues is essential for the correct description of the transition state energies. Moreover, the same selectivity is obtained with QM/MM with QM region **DA** and the cluster model **A**, which both treat the same atoms with a QM method.

The ^5^ **TS1**_HA,DA,C2S,σ_ and ^5^ **TS1**_HA,DA,C2R,σ_ transition states have short Fe−O distances of 1.660 and 1.677 Å, respectively, and also have a relatively small imaginary frequency of i506 and i870 cm^−1^. These unusual distances and frequency values point to large constraints on the chemical structure of the transition states. Indeed the O−H−C^2^ angle is relatively large, namely 160 and 167°. By contrast, the ^5^ **TS1**_HA,DA,C1S,σ_ and ^5^ **TS1**_HA,DA,C1R,σ_ structures have a significantly longer Fe−O bond of 1.812 and 1.838 Å due to electron transfer from the substrate into the antibonding σ*_z2_ orbital, while the electron transfer is later in the C^2^ transition states. Thus, the ^5^ **TS1**_HA,DA,C1R,σ_ and ^5^ **TS1**_HA,DA,C1S,σ_ have a spin density of −0.60 and −0.58 on the substrate, which is typical for ^5^σ‐type transition states and the transfer of an up‐spin electron from substrate into the σ*_z2_ orbital. The ^5^ **TS1**_HA,DA,C1R,σ_ and ^5^ **TS1**_HA,DA,C1S,σ_ structures are product‐like with a shorter O−H distances of 1.250/1.232 Å than the C^1^−H distance of 1.351/1.336 Å. Interestingly, both structures have a bent O−H−C^1^ orientation of 150–151°, while typically values around 180° are expected for this pathway.

### Comparison of DFT and QM/MM results and link to experiment

Experimentally a rate constant k_exp_=13 s^−1^ at 5 °C was determined for hydrogen atom abstraction by an iron(IV)‐oxo species leading to predominant *R*‐1‐hydroxytaurine products.^[10a]^ Using transition state theory, this rate constant corresponds to a free energy of activation at 5 °C of ΔG^≠^_exp,5 °C_=14.83 kcal mol^−1^. To find out how our various models compare to experiment, we summarize the various transition state energies obtained with QM cluster and QM/MM methods described in this work in Table 1. Using cluster model **B** a ΔG^≠^_HA,B,C1R,σ_=15.4 kcal mol^−1^ is obtained (Figure 5, Table 1), which is in excellent agreement with the experimentally obtained result. Unfortunately, we located lower energy barriers for hydrogen atom abstraction from the C^2^ and *pro*‐*S* C^1^ positions of taurine. Therefore, cluster model **B** predicts the correct kinetics for the *pro*‐*R* C^1^−H abstraction pathway but for this model the wrong kinetics for the alternative hydrogen atom abstraction pathways is obtained and hence it gives an overall wrong selectivity.

**Table 1:** DFT calculated free energies of activation (Δ*G* ^≠^) for the *pro*‐*R* C^1^−H, *pro*‐*S* C^1^−H, *pro*‐*S* C^2^−H and *pro*‐*R* C^2^−H hydrogen atom abstraction pathways from taurine by TauD models.^[a,b]^

Method/Model	**TS1**_C1R,σ_	**TS1**_C1S,σ_	**TS1**_C2S,σ_	**TS1**_C2R,σ_
DFT model **A**	4.2	18.1	13.8	15.0
DFT model **B**	15.4	14.2	13.1	14.5
DFT model **B2**	27.8	26.8	24.4	30.5
DFT model **C**	17.1	16.3	26.6	28.6
DFT model **C2**	30.9	25.7	24.9	28.6
QM/MM model **DA**	6.7	8.3	26.4	24.9
QM/MM model **DB**	22.6	21.8	13.3	9.4
Experiment^[c]^	14.83

[a] Δ*G*_BS2_ values contain energies calculated at UB3LYP/BS2 with solvent model included, while geometries are optimized at UB3LYP/BS1. [b] Values in kcal mol^−1^. [c] Rate constant from Ref. [10a] converted into a free energy using transition state theory.

We then expanded the cluster model to model **C** and obtained a ΔG^≠^_HA,C,C1R,σ_=17.1 kcal mol^−1^, while the corresponding C^2^−H hydrogen atom abstraction barriers are well higher in energy. Moreover, both ^5^ **TS1**_HA,C,C1R,σ_ and ^5^ **TS1**_HA,C,C1S,σ_ transition states lead to the same radical intermediate that preferentially reacts to form *R*‐1‐hydroxytaurine. Consequently, the slightly enlarged model **C** predicts the correct kinetics and regio‐ and enantioselectivity of the TauD enzyme well. Finally, with QM/MM a free energy of activation of ΔG^≠^_HA,DA,C1R,σ_=6.7 kcal mol^−1^ is found that is well lower in energy than the other C−H abstraction barriers. Therefore, QM/MM with QM region **A** gives the correct selectivity but its barriers are well underestimated with respect to experiment. Most probably this is the result of the choice of a small QM region for the QM/MM calculations as it matches the result of the minimal cluster model **A** the best. Finally, to establish the effect of the His_70_ protonation state on the reactivity and selectivity of TauD enzymes, we took model **C** and removed one of the His_70_ protons to obtain model **C2**. We then reoptimized all four hydrogen atom abstraction transition states and find the C1S and C2S barriers to be close in free energy, namely ΔG=25.7 kcal mol^−1^ for ^5^ **TS1**_HA,C2,C1S,σ_ and ΔG=24.9 kcal mol^−1^ for ^5^ **TS1**_HA,C2,C2S,σ_. Therefore, the His_70_ protonation state is essential for TauD to obtain the correct selectivity and guide the reaction to C^1^‐hydroxylation. The absolute barriers of the model **C2** transition states; however, are well higher than those for model **B** by more than ΔG=10 kcal mol^−1^ (see Table 1). Therefore, we analyzed the structural differences between model **B** and **C** further to find the origin of the lowering of the transition state barriers.

**Figure 8:**
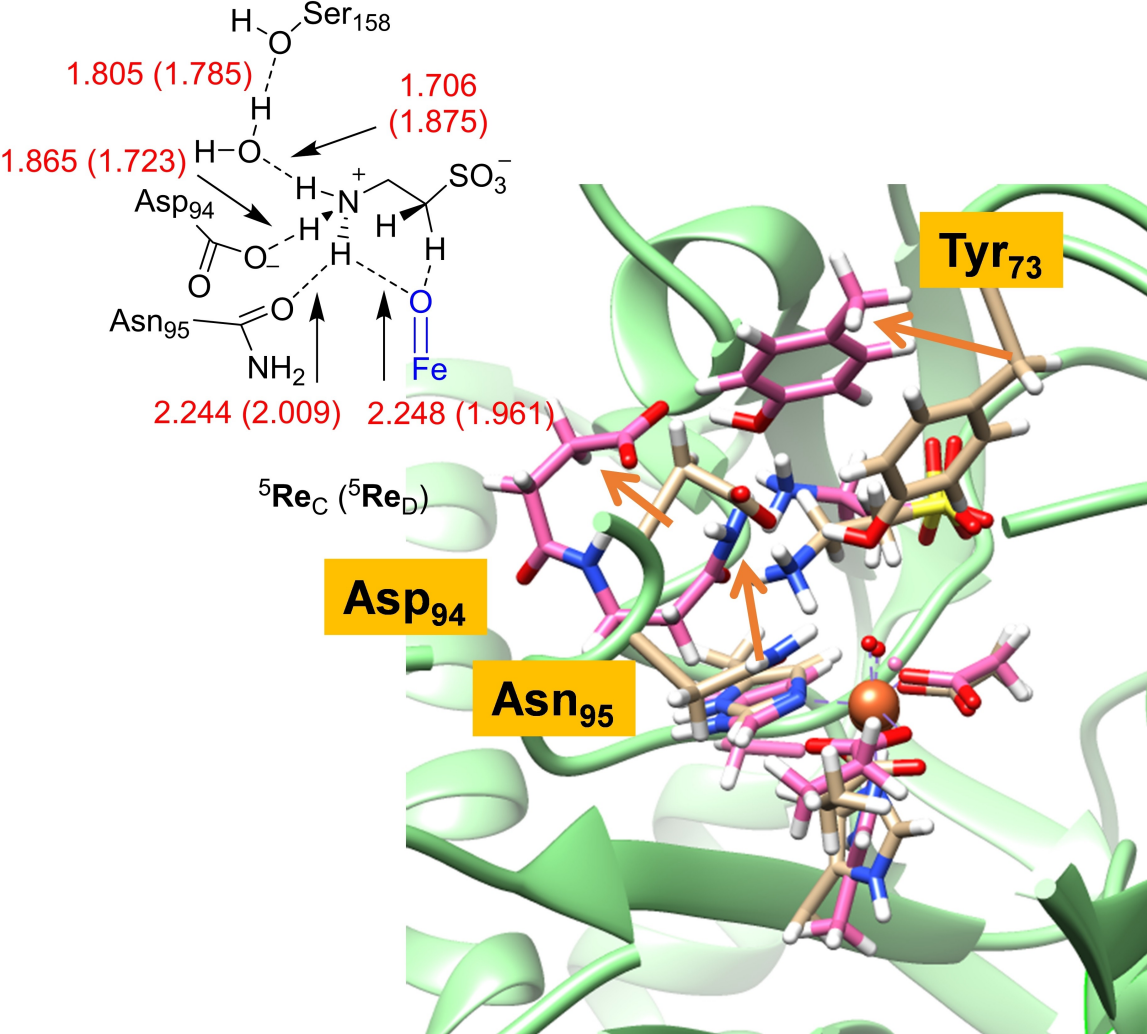
Hydrogen bonding network surrounding taurine substrate in ^5^ **Re**_C_ and ^5^ **Re**_D_ and an overlay of ^5^ **Re**_B_ (in purple) and ^5^ **Re**_D_. Bond lengths are given in angstroms. The changes in position of protein residues between model **B** and **D** are identified with orange arrows.

To understand the second‐coordination sphere better, we created a geometric overlay of the ^5^ **Re**_B_ and ^5^ **Re**_D_ structures. As discussed above in Figure 2 an overlay of the first‐coordination sphere of the iron center gives negligible differences between the two structures that would not explain reactivity differences. Therefore, long‐range perturbations of the second‐coordination sphere must affect substrate binding and positioning and its thermochemical properties. We then focused on the second‐coordination sphere and particularly the taurine binding position, see Figure 8. Although the C^1^H_2_−SO_3_^−^ group of taurine is virtually in the same position in ^5^ **Re**_B_, ^5^ **Re**_C_ and ^5^ **Re**_D_, actually the amine group of the substrate has shifted slightly and in ^5^ **Re**_C_/^5^ **Re**_D_ it forms a hydrogen bond with the oxo group. As a consequence of this new hydrogen bond there is a shift in the hydrogen bonding network around the taurine amine group and also changes in the positions of the Tyr_73_, Asp_94_, and Asn_95_ side chains in the substrate binding pocket are observed. Most probably this hydrogen bonding interaction from the taurine amine group to the oxo triggers a hydrogen atom from the C1S position and makes hydrogen atom abstraction from the other three positions more difficult. Thus, a hydrogen bond of 1.961 Å between the taurine amine group and oxo is formed, while it also interacts with the oxygen atom of Asn_95_ at a distance of 2.009 Å.

The amine group of taurine interacts further with the carboxylate of Asp_94_ and through a crystal water molecule with the alcohol group of Ser_158_ in a tight hydrogen bonding network. A similar hydrogen bonding network is seen in cluster model ^5^ **Re**_C_. Our calculations, therefore, show the importance of the hydrogen bonding network around taurine involving the side‐chains of Asp_94_, Asn_95_ and Ser_158_. Indeed, active site mutations where these residues were replaced by alternative (smaller) side‐chains led to reduced activity of TauD and much smaller rate constants.^[22]^

As this hydrogen bonding network has shifted in the DFT cluster model ^5^ **Re**_B_ with respect to that in ^5^ **Re**_C_/^5^ **Re**_D_, we decided to explore the effect of this hydrogen bonding network further and considered an alternative cluster model ^5^ **Re**_B2_ that is based on ^5^ **Re**_B_ and the corresponding hydrogen atom transition states but has the groups Asp_94_, Asn_95_, Ser_158_ and Phe_159_ removed. The calculations on Model **B2** raise the barrier of ^5^ **TS1**_HA,B2,C1S,σ_ to ΔG^≠^=26.8 kcal mol^−1^ as compared to the model **B** transition state. By contrast, the ^5^ **TS1**_HA,B2,C1R,σ_, ^5^ **TS1**_HA,B2,C2S,σ_ and ^5^ **TS1**_HA,B2,C2R,σ_ barriers are raised even higher to ΔG^≠^=27.8, 24.4 and 30.5 kcal mol^−1^, respectively. As such, the hydrogen bonding network lowers the C^1^−H and C^2^−H abstraction barriers dramatically and affects the rate of the reaction by positioning the substrate in a better orientation. Despite the fact that the hydrogen bonding network of Asp_94_, Asn_95_ and Ser_158_ influences the kinetics of the reaction dramatically, actually it does not influence the regioselectivity dramatically and the same ordering of the transition states is obtained with the lowest barrier for ^5^ **TS1**_HA,B2,C2S,σ_ for models **B** and **B2**. Our calculations, therefore, match experimental data on TauD variants with Asp94Ala, Asn95Ala, Asn95Asp, Ser158Ala, Phe159Leu and Phe159Val mutations that showed a considerable reduction of the rate constant of taurine hydroxylation as compared to the wild‐type enzyme.[^22^, ^23^] It was suggested that these mutants will bring additional crystal water molecules into the substrate binding pocket that disrupt the hydrogen bonding network and make the substrate more mobile.

**Figure 9:**
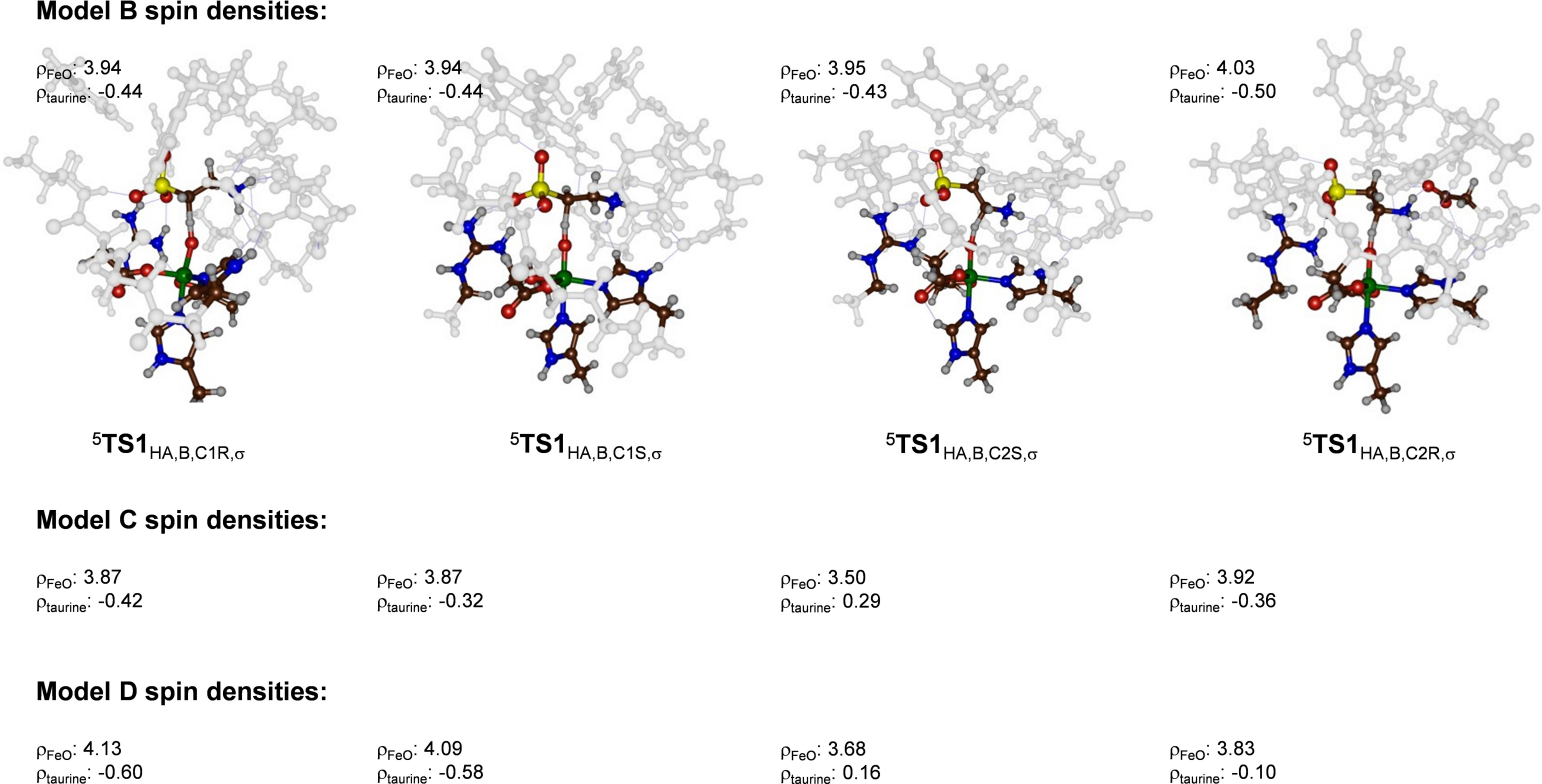
Group spin densities of the hydrogen atom abstraction transition states for the DFT model **B** and **C** and the QM/MM model **D** with QM region **A**.

To understand how the hydrogen bonding network and particularly the close approach of the protonated amine group of substrate to the oxidant affects the charge and spin distributions we analyzed the spin densities in detail. Figure 9 shows the optimized hydrogen atom abstraction transition states and the spin density obtained on the FeO and taurine groups. In the QM/MM optimized transition states for C^1^−H abstraction, a large amount of spin is found on the substrate moiety (−0.60 in ^5^ **TS1**_HA,DA,C1R,σ_ and −0.58 in ^5^ **TS1**_HA,DA,C1S,σ_). By contrast, in the corresponding model **B** transition states only −0.44 spin is found on the substrate. Therefore, the hydrogen bonding interaction between the amine group of substrate and the iron(IV)‐oxo species polarizes the C^1^−H bond stronger and causes an earlier electron transfer from substrate to oxidant. In the QM/MM structure, the C^2^−H transition states are late with little electron transfer in the transition states: ^5^ **TS1**_HA,D,C2S,σ_ has ρ_taurine_=0.16 and ^5^ **TS1**_HA,D,C2R,σ_ has ρ_taurine_=−0.10. By contrast, these transition states have significant radical character in the cluster model **B** optimized structures with values of −0.43 and −0.50, respectively. As a consequence, a local electric field and dipole moment perturbs the electronic configuration of the C^2^−H hydrogen atom abstraction transition states dramatically and results in a change of ordering from the large cluster model to the QM/MM system. A comparison of the spin densities for ^5^ **TS1**_HA,B,C1R,σ_ and ^5^ **TS1**_HA,C,C1R,σ_ gives very similar values, which is as expected as their free energy of activation was very much alike. By contrast, the spin density values for the two *pro*‐*S* and *pro*‐*R* C^2^−H hydrogen atom abstraction transition states for model **B** and **C** are very different as expected from the dramatic differences in barrier height.

### Environmental effects on C−H bond strengths

**Figure 10:**
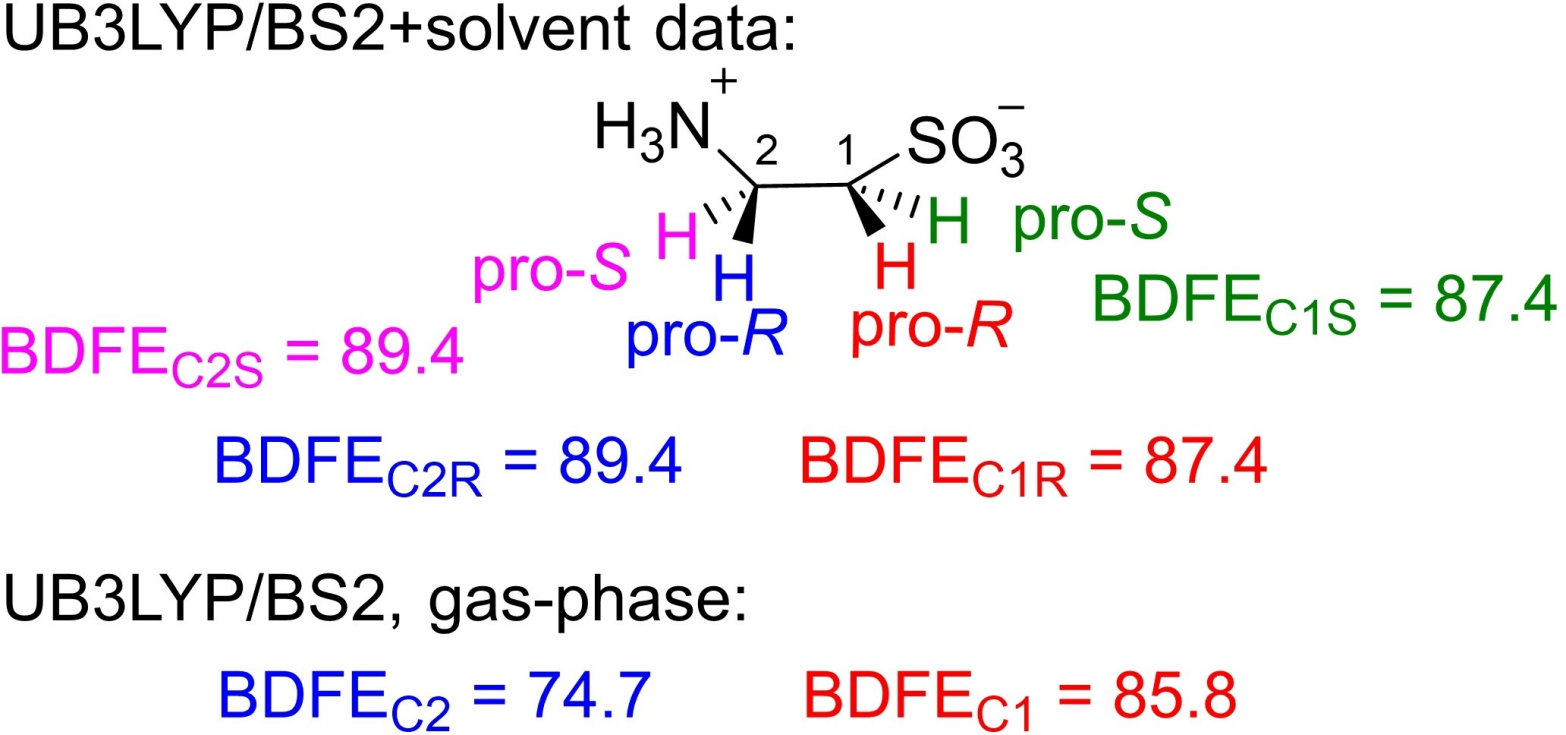
Calculated BDFE values for the *pro*‐*R* and *pro*‐*S* C−H bonds of an isolated taurine molecule as calculated at UB3LYP/BS2 level of theory: Data obtained at ΔG_solv_ (top) or ΔG_gas_ level of theory (in kcal mol^−1^).

Previously, we showed that external perturbations such as hydrogen bonding interactions, electric dipole moments and electric field effects can influence C−H bond strengths and selectivities.^[24]^ In particular, electric field effect calculations by various groups identified changes in reaction rates, as well as reaction mechanism with external electric field perturbations included.^[25]^ Therefore, we initially calculated the substrate C−H bond dissociation free energy (BDFE) values of taurine for all four C−H bonds from the homolytic cleavage of the C−H bond by calculating the substrate, a hydrogen atom and the substrate with a hydrogen atom removed. The gas‐phase calculated BDFE values have been taken from Ref. [12]. The data obtained at the UB3LYP/BS2 level of theory are shown in Figure 10. As can be seen from Figure 10, with a solvent model included in the calculations the four C−H bond strengths are in a narrow window of 2.0 kcal mol^−1^ with the C^1^−H bond the weakest of those bonds. Nevertheless, the small differences in substrate C−H bond strength would imply products originating from all four possible hydrogen atom abstraction channels.

We also calculated the BDFE values in the gas‐phase (without solvent model) and found BDFE_C1,no solvent_=85.8 kcal mol^−1^ and BDFE_C2,no solvent_=74.7 kcal mol^−1^.^[12]^ Consequently, the addition of a solvent model changes the ordering of the BDFE values for the C^1^−H and C^2^−H bond strengths. As such the C−H bond strengths will be sensitive to external perturbations and polar groups. As the BDFE values of taurine appear to be sensitive to external polarization effects, we decided to calculate them using electric field effect perturbations included.

**Figure 11:**
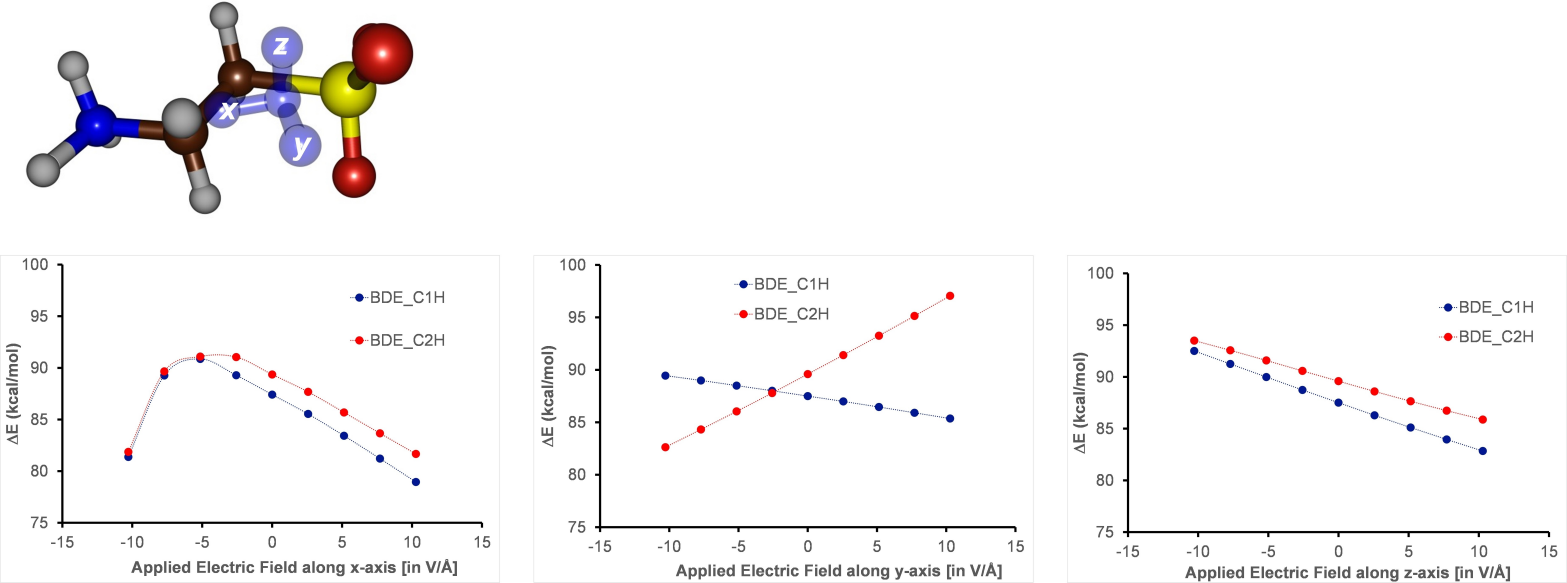
Electric field effect perturbations on the C^1^−H (in blue) and C^2^−H (in red) bond strengths (BDFE) of taurine as calculated at UB3LYP/6‐311+G* in the gas‐phase. The x‐, y‐, and z‐axis are defined through the centre of mass of taurine with the positive direction as defined in Gaussian identified with the arrows in blue. The field values are reported in V/Å. The electric field perturbation was added to the solvent‐corrected BDFE values for *pro*‐*R* C^1^−H and C^2^−H from Figure 10.

Experimentally, only *pro*‐*R* C^1^−H hydrogen atom abstraction takes place, which implies that the protein environment through external electrostatic perturbations must affect the regioselectivity of the reaction and maybe even the C−H bond strengths. In particular, in recent work of our group we showed that external electric fields along a specific direction can change C−H bond strengths and in enzymes guide a regioselectivity pathway.[^24a^, ^24c^] Therefore, we performed electric field based calculations on an isolated taurine substrate using fields along the positive and negative x‐, y‐ and z‐axis of the molecules. Figure 11 shows the evolvement of the BDFE values for C1R and C2R under electric field perturbations along the molecular x‐, y‐ and z‐axis of taurine. As can be seen a field along the molecular skeleton of taurine (z‐axis) has little effect on the relative BDFE values for *pro*‐*R* C^1^−H and C^2^−H bond strengths and along the whole series the C^1^−H is the weakest of the two. By contrast, a field along the molecular y‐axis, i. e. along the *pro*‐*R* C^1^−H bond, either pushes electron density towards the carbon or pulls it away. As a consequence with a positive field along the y‐axis the C^1^−H bond is weakest, while with a field in the opposite direction the C^2^−H bond becomes the weakest of the two bonds. Finally, with a field along the x‐direction the two BDFE values become almost equal, which should result in a mixture of products originating from C^1^−H and C^2^−H hydrogen atom abstraction processes. These electric field results show that the electrostatic perturbations of the protein can have a major effect on the regioselectivity of the reaction and push the mechanism into a specific direction.

**Figure 12:**
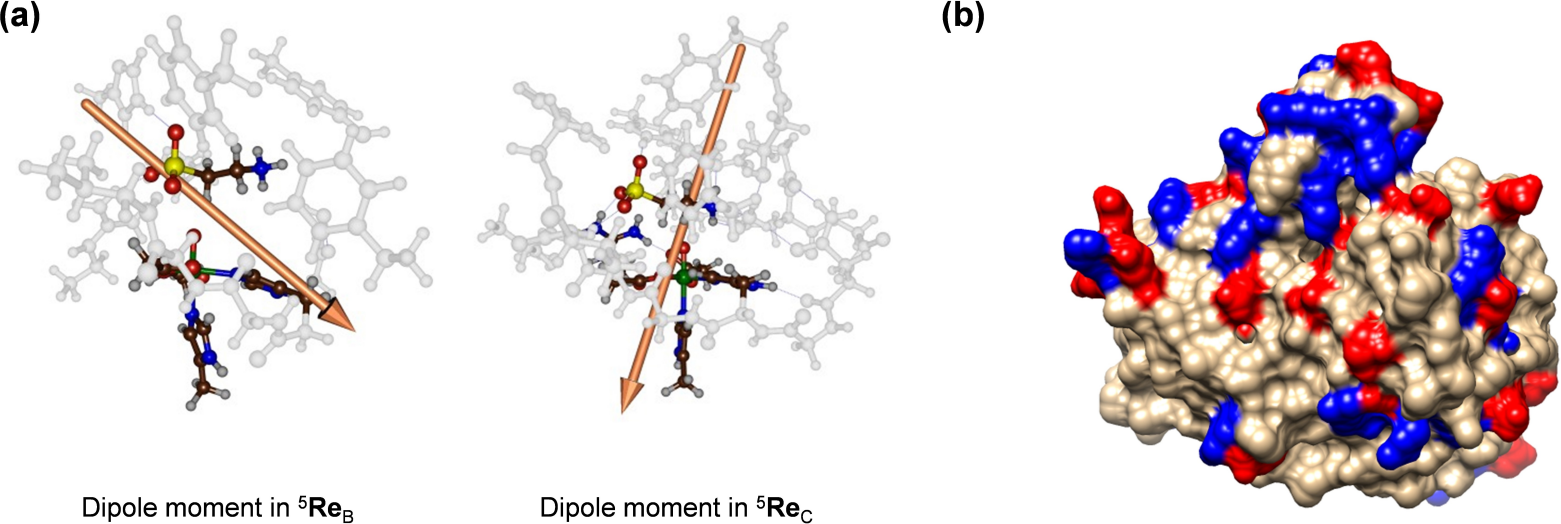
(a) Electric dipole moment vector of the reactant complexes ^5^ **Re**_B_ and ^5^ **Re**_C_. (b) Charge distribution in TauD as analysed from the 1OS7 pdb file: positively charged amino acids in blue and negatively charged ones in red.

Figure 12 shows the electric dipole moment vector in complexes ^5^ **Re**_B_ and ^5^ **Re**_C_. Thus, in ^5^ **Re**_C_ the vector points along the C^2^−H bond of the substrate and according to the electric field effect perturbations from Figure 11 that perturbs along the molecular y‐axis of the substrate and strengthens these C^2^−H bonds. Indeed, model **C** shows high energy barriers for hydrogen atom abstraction from the C^2^−H bonds of taurine. By contrast to the electric dipole vector in model ^5^ **Re**_C_, the vector in ^5^ **Re**_B_ points in a very different direction that corresponds to the x‐ and z‐direction electric fields of Figure 11. As shown in Figure 11, fields along the molecular x‐ and z‐axis of the substrate have little effect on the relative bond strengths of the C^1^−H and C^2^−H bonds. As a consequence, model **B** has an electric dipole vector that little affects the C−H bonds strengths at the C^1^−H and C^2^−H bonds of taurine. Indeed, using model **B** we find all hydrogen atom abstraction barriers within a small window of about 3 kcal mol^−1^ that would predict an unselective reaction with multiple products. A further analysis of the 1OS7 pdb file (right‐hand‐side of Figure 12) focuses on the charged residues in the protein structure. The TauD structure contains 19 glutamate and 14 aspartate amino acids with a negatively charged side‐chain, while there are 19 arginine and 9 lysine amino acids with a positively charged side‐chain. Therefore, the protein structure is overall negatively charged and these polar amino acids induce an electric dipole moment and a local electric field on the reaction center. The dipole moment shown for large cluster model **C** indeed points in the same direction as the favorable electric field direction for selective C^1^‐hydroxylation. By contrast, the dipole moment for model **B** points in the wrong direction as compared to the favorable electric field direction for selective C^1^‐hydroxylation and indeed predicts favorable C^2^‐hydroxylation instead. Consequently, large cluster models like model **C** reproduce the structure and reactivity of TauD well thanks to the long‐large interactions and charge and dipole moments of the second‐coordination sphere that influence substrate positioning and its reactivity.

## Conclusion

A combination of density functional theory cluster calculations and quantum mechanics/molecular mechanics studies are presented on taurine/α‐ketoglutarate‐dependent dioxygenase. In particular, pathways leading to *R*‐ and *S*‐1‐hydroxytaurine and *R*‐ and *S*‐2‐hydroxytaurine have been performed. The reactions are stepwise via a radical intermediate and create an intermediate of σ‐type with a high‐spin iron(III) antiferromagnetically coupled to a substrate radical. A number of large cluster models as well as QM/MM with various size QM region have been studied. These cluster models range from a minimal model that contains the first‐coordination sphere and substrate only (model **A**) to models with expanded second‐coordination sphere environments surrounding the oxidant and substrate. We show that the choice of the model is crucial for the calculations and particularly there is an important dipole moment in the active site structure that guides the reaction to the correct selectivity. This dipole moment is accomplished through a protonated His residue in the vicinity of the substrate (His_70_), which needs to be in its protonated form in the catalytic cycle. Models without a proton on this His_70_ residue predict the wrong selectivity and/or kinetics. In addition, the calculations highlight the hydrogen bonding network of Asp_94_, Asn_95_ and Ser_158_ that position the amine group of taurine substrate and position it in an ideal orientation for optimal kinetics. Our calculations using large cluster models that include key hydrogen bonding interactions in the substrate binding pocket and His_70_ protonated reproduce experimental rates and selectivity excellently and give a dominant C^1^‐hydroxylation channel leading to *R*‐1‐hydroxytaurine products. This is triggered by charged active site residues including a protonated His_70_ group.

## Experimental Section

**Set up of the system**: Our model set‐up starts from the crystal structure coordinates of substrate‐ and α‐KG‐bound TauD as deposited by the 1OS7 pdb file, which is an enzymatic monomer.^[9]^ Hydrogen atoms were added to the structure based on pH 7 conditions with the H++ webserver.^[26]^ The MCPB.py tool as implemented in the AMBER‐18 software package was used to generate the force field parameters for a penta‐coordinated iron(II) centre, while for the description of the protein atoms the ff14SB4 force field parameters were used.[^27^, ^28^] Substrate taurine and α‐KG were described with the second generation General Amber Force Field (GAFF2) using AM1‐BCC charges.^[29]^ These GAFF2 parameters were generated with the antechamber and parmchk2 modules of the AMBER‐18 software package. Finally, the substrate‐bound enzyme structure was solvated in a cubic box of TIP3P water molecules and subsequently neutralized by adding Na^+^ ions. Thereafter, a molecular dynamics (MD) simulation was carried out using the Particle Mesh Ewald Molecular Dynamics (PMEMD) module as implemented in the AMBER‐18 software package.^[30]^ The system was minimized using 2000 steps of steepest decent minimization with a starting restraint potential of 2.0 kcal mol^−1^ on all heavy atoms of the protein, which was gradually released. After that the system was heated for 100 ps from a temperature of 0 K to 298.15 K under NVT ensemble conditions with the Langevin thermostate and subsequently equilibrated for 1 ns under NPT conditions using the Berendsen barostate without constraints to the energy and the structure.^[31]^ Thereafter, a 20 ns MD simulation under NPT conditions was carried out in 2 fs time‐steps, using the SHAKE protocol on hydrogen atoms and a 10 Å non‐bonded cut‐off with periodic boundary conditions. The MD simulation (Supporting Information, Figures S1 and S2) shows that the system is highly rigid and little changes with respect to the crystal structure coordinates are seen.

**QM/MM approaches**: QM/MM calculations follow procedures as reported previously.^[32]^ In general, the QM/MM calculations were carried out using ONIOM scheme as implemented in the Gaussian software package.[^33^, ^34^] The QM/MM calculations were setup using molUP, a VMD plugin, from the equilibrated final structure of the MD simulation.^[35]^ The QM/MM model **D** consist of the complete protein, substrate and water molecules within 15 Å of the protein and has a total of 9,551 atoms and has His_70_ in its singly protonated form. The QM region consist of the first‐coordination sphere around the iron(IV)‐oxo group and substrate and the link‐atom procedure was used to bridge the QM and MM regions.^[36]^ QM region **A** has 53 atoms, while QM region **B** contains the atoms as shown in Figure 2a. The QM region is treated using DFT with the unrestricted B3LYP hybrid functional and a basis set with LANL2DZ (with electron core potential) on iron and 6‐31G* on the rest of the atoms: basis set BS1,[^37^, ^38^] while the MM region was treated with the Amber force field.[^28^, ^30^] We initially ran QM/MM geometry optimizations using QM region **A** and followed these up with a set of single point calculations whereby the QM region was expanded to the atoms shown in Figure 2 under model **B**. The electrostatic interactions between the QM and MM regions were described with the electronic embedding scheme. The geometry optimizations were performed with UB3LYP/BS1 level of DFT and followed by an analytical frequency calculation at 298.15 K and 1 atm. The transition states were located using potential energy scan calculations followed by full geometry optimization with the Berny algorithm. The energy values were corrected through single point calculations at the ONIOM(UB3LYP/BS2:Amber) level of theory with BS2 a basis set representing LACV3P+ (with electron core potential) on iron and 6‐311+G* on the rest of the atoms.

The M06 density functional method was also tested for the QM region in the QM/MM calculations,^[39]^ however this gave essentially similar energetics than those found with B3LYP, see Supporting Information.

**DFT cluster models**: Cluster models focus on the substrate and oxidant binding environment and the second‐coordination sphere and consider all atoms in the model with a quantum chemical approach.^[40]^ As the MD simulation gives little variety between the active site orientation of the various snapshots, we created five cluster models (**A**, **B**, **B2**, **C** and **C2**) based on the last step of the MD simulation, see Figure 2. We truncated the active site model by selecting the residues of amino acids and second coordination sphere groups, which determine substrate and oxidant binding and positioning and particularly include all polar (hydrogen bonded and π‐stacking interactions) close to the metal and substrate. In the active site model we replaced the iron(II) ion by iron(IV)‐oxo species, while α‐KG was replaced by succinate. A truncated model (minimal) cluster model was considered, namely a minimal cluster model **A** that contains only the first coordination sphere of residues to iron and the substrate and had 72 atoms. Thereafter, larger cluster models **B** and **C** were created that incorporate the environments around substrate and oxidant and their hydrogen bonding interactions. The active site cluster model **B** incorporates the metal and its first‐coordination sphere as well as the substrate and a number of second‐coordination sphere residues that determine the size and shape of the substrate binding pocket and incur hydrogen bonding interactions. Thus, the model includes a short protein chain of amino acids that links to the equatorial ligands of the iron, namely His_99_−Thr_100_−Asp_101_−Val_102_. In addition, two short chains for Ser_158_−Phe_159_ and Asp_94_−Asn_95_ were included. The axial histidine ligand (His_255_) of iron was abbreviated to methylimidazole and α‐KG replaced by acetate. Finally, the side chains of the residues His_70_, Tyr_73_, Ile_83_, Asn_97_, Phe_104_, Phe_206_ and Arg_270_ as well as substrate taurine were included. Overall our cluster model contains 244 atoms and has a neutral charge. To prevent the model from changing dramatically in structure during the geometry optimizations we placed some constraints on some of the C^α^‐protein atoms as identified with a star in Figure 2. Cluster Model **C** contains 279 atoms and is based on the QM/MM optimized geometry and had the atoms of cluster model **B** expanded with the full peptide chain Asp_94_−Asn_95_−Asp_96_−Asn_97_−Trp_98_−His_99_−Thr_100_−Asp_101_−Val_102_−Thr_103_−Phe_104_ with Asp_96_, Trp_98_ and Thr_100_ truncated to a Gly residue. In addition, model **C** has the His_70_ residue doubly protonated. To test the protein environment on the kinetics and selectivity of the reactions, we created two further models based on models **B** and **C**, namely **B2** and **C2**. Model **C2** is similar to model **C** but has His_70_ singly protonated, while model **B2** is model **B** with the chains of Asp_94_, Asn_95_, Ser_158_ and Phe_159_ removed.

**DFT procedures**: The Gaussian‐09 software package was used for all quantum chemical calculations discussed here.^[33]^ Following previous experience with cluster models of nonheme iron complexes,^[41]^ we utilized the unrestricted B3LYP density functional method in combination with a LANL2DZ (with electron core potential) on iron and 6‐31G* on the rest of the atoms: basis set BS1.[^37^, ^38^] This method was shown to reproduce experimental spin‐state assignments and rate‐constants well.^[42]^ All geometry optimizations were performed with these basis sets in which all local minima were verified by the absence of negative eigenvalues in the vibrational frequency analysis while all the transition state structures were found using the Berny algorithm, and verified by vibrational analysis and visualized by animating the imaginary frequency. For key transition states also intrinsic reaction coordinate calculations were done, which confirmed the transition states to connect with the local minima as identified. In order to improve the energetics, single point energies on the optimized geometries were calculated at UB3LYP/BS2, whereby BS2 represents the LACV3P+ (with electron core potential) on iron and 6‐311+G* on the rest of the atoms. The latter set of calculations included a continuum polarized conductor model (CPCM) with a dielectric constant mimicking chlorobenzene and the dispersion correction (D3) developed by Grimme.^[43]^ For a selection of structures, we ran full geometry optimizations at UB3LYP‐D3/BS1 level of theory, but these studies gave the same trends as those obtained at UB3LYP/BS1, see Supporting Information for details. To obtain the free energies at 298.15 K and 1 atm, the zero‐point energy (ZPE), thermal corrections and entropy contribution evaluated from the unscaled vibrational frequencies at the UB3LYP/BS1 level of theory were then added to the electronic energies calculated from the same level of DFT.

The primary kinetic isotopic effect (KIE′s) were calculated using the classical equations due to Eyring (Eq. (1)) and with tunnelling corrections included due to Wigner (Eq. (2) and 3).^[44]^ In the Eyring KIE, the activation free energy (ΔG^≠^) of hydrogen and deuterium substituted reaction was considered in the gas phase at room temperature (T=298.15 K) with R being the gas constant. By contrast, the Wigner tunnelling factor incorporates a factor that considers the change in the magnitude of the imaginary vibrational frequency in the transition state for the hydrogen and deuterium substituted systems. In Equation (3), h is Planck's constant and k_B_ is the Boltzmann constant, The KIE values for the various hydrogen atom abstraction pathways were calculated by replacing the both *pro*‐*R* and *pro*‐*S* hydrogen atoms at the C^1^ and C^2^ positions of taurine substrate.

(1) $$\mathrm{KIE}{}_{\mathrm{Eyring}}=\exp\left[\left(\Delta G{}^{\neq }{}_{D}-\Delta G{}^{\neq }{}_{H}\right)/\mathrm{RT}\right]$$

(2) $$\mathrm{KIE}{}_{\mathrm{Wigner}}=\mathrm{KIE}{}_{\mathrm{Eyring}}\times Q{}_{t,H}/Q{}_{t,D}$$

(3) $$Q{}_{t}=1+\left(h\nu /k{}_{B}T\right)){}^{2}/24$$

Finally, we performed the electric field effect (EFE) calculations as implemented in Gaussian software package using the “field” keyword.^[33]^ The EFE is calculated by performing a single point energy calculation on various optimized geometries with an electric field perturbation along the molecular x, y or z‐axis with the positive direction as defined in Gaussian. These electric field perturbations were done with various field magnitudes in either positive or negative field directions along each principal axis.

## Conflict of interest

The authors declare no conflict of interest.

1

## Supporting information

As a service to our authors and readers, this journal provides supporting information supplied by the authors. Such materials are peer reviewed and may be re‐organized for online delivery, but are not copy‐edited or typeset. Technical support issues arising from supporting information (other than missing files) should be addressed to the authors.

Supporting Information

## Data Availability

The data that support the findings of this study are available in the supplementary material of this article.
