# Supplementary material for: Electrostatic Perturbations in the Substrate‐Binding Pocket of Taurine/α‐Ketoglutarate Dioxygenase Determine its Selectivity
Source: Chemistry. 2022 Jan 22;28(9):e202104167. doi: 10.1002/chem.202104167 (PMC9304159; doi:10.1002/chem.202104167)
Supplement: Supplementary file 1 — Supporting Information [file CHEM-28-0-s001.pdf]

Chemistry–A European Journal

Supporting Information

Electrostatic Perturbations in the Substrate-Binding Pocket of Taurine/α-Ketoglutarate Dioxygenase Determine its Selectivity

Hafiz Saqib Ali and Sam P. de Visser*

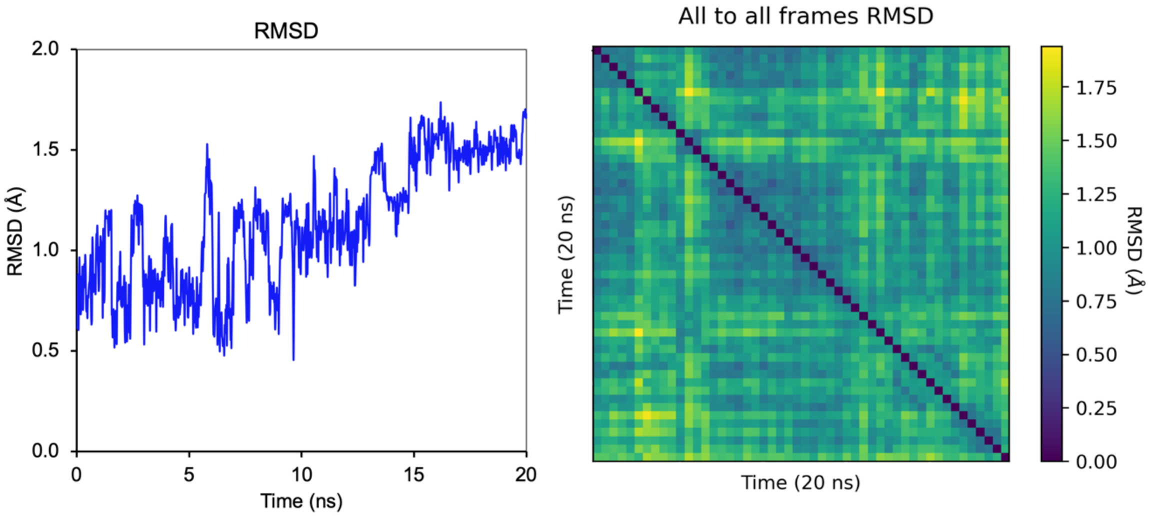

Figure S1: Convergence analysis used in this study to assess the stability/flexibility of the system for 20 ns of the MD trajectories: RMSD and all-to-all frames RMSD.

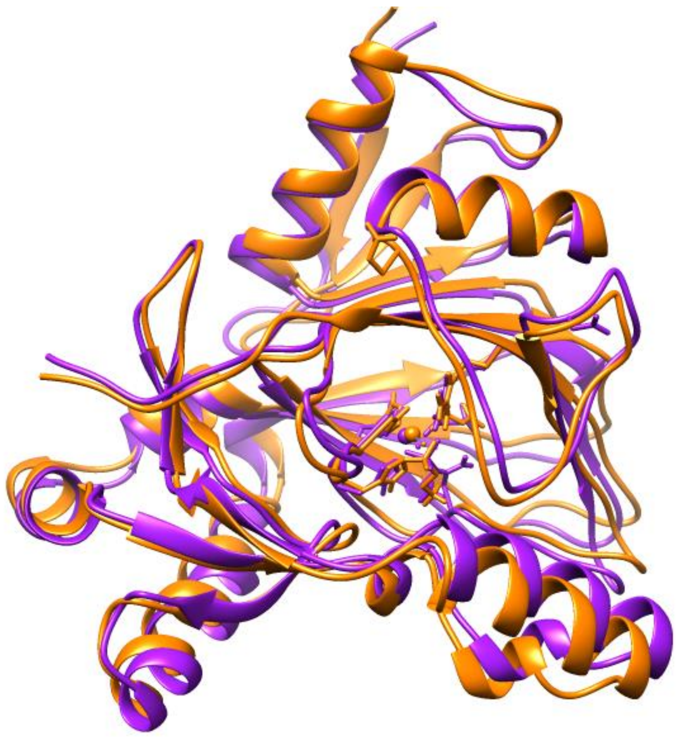

Figure S2: Overlay of the structures of the last point of the 20 ns MD simulation (in purple) with the crystal structure coordinates of the 1OS7 pdb file (in orange).

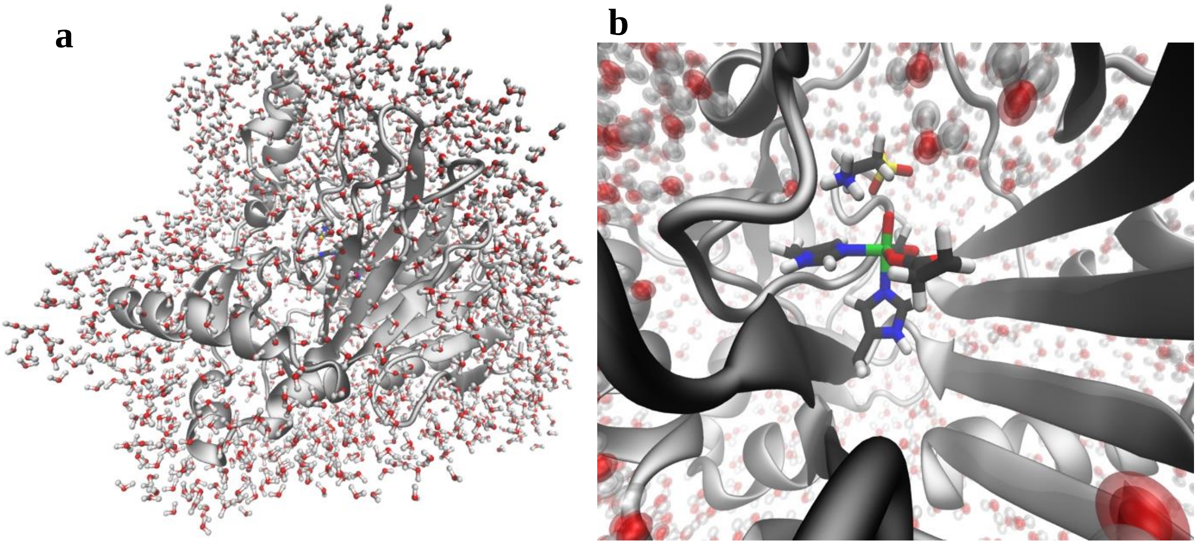

Figure S3: (a) QM/MM optimized geometry of the quintet spin reactant complex (^{5}**Re**$_{D}$) of the TauD enzyme. **(b)** Zoom-in into the QM region of the system.

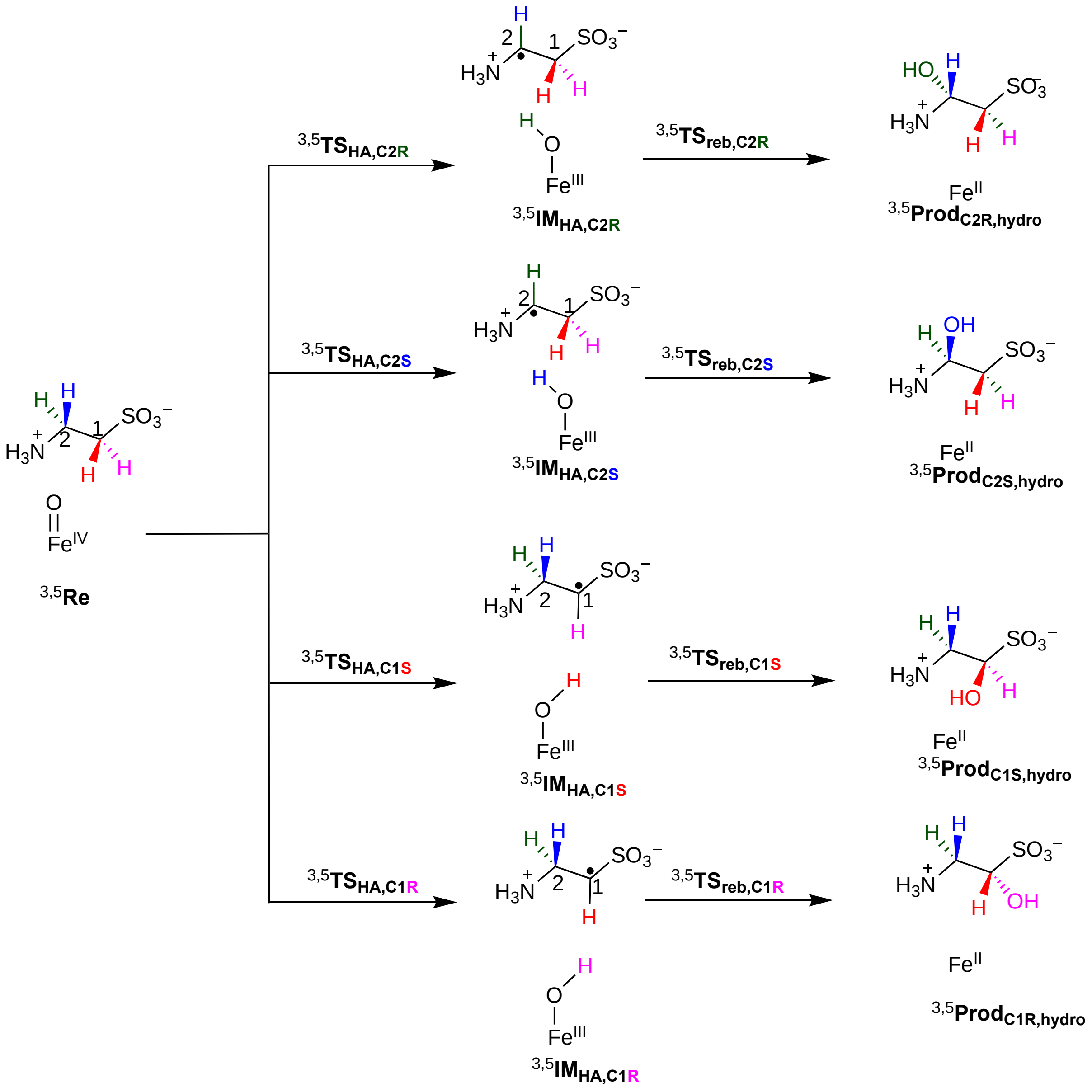

Scheme S1. Reaction mechanisms explored in this work for taurine hydroxylation at the pro-*R* and pro-*S* C^1–H and C^2–H positions by the iron(IV)-oxo species of TauD with definition of labels of local minima and transition states.

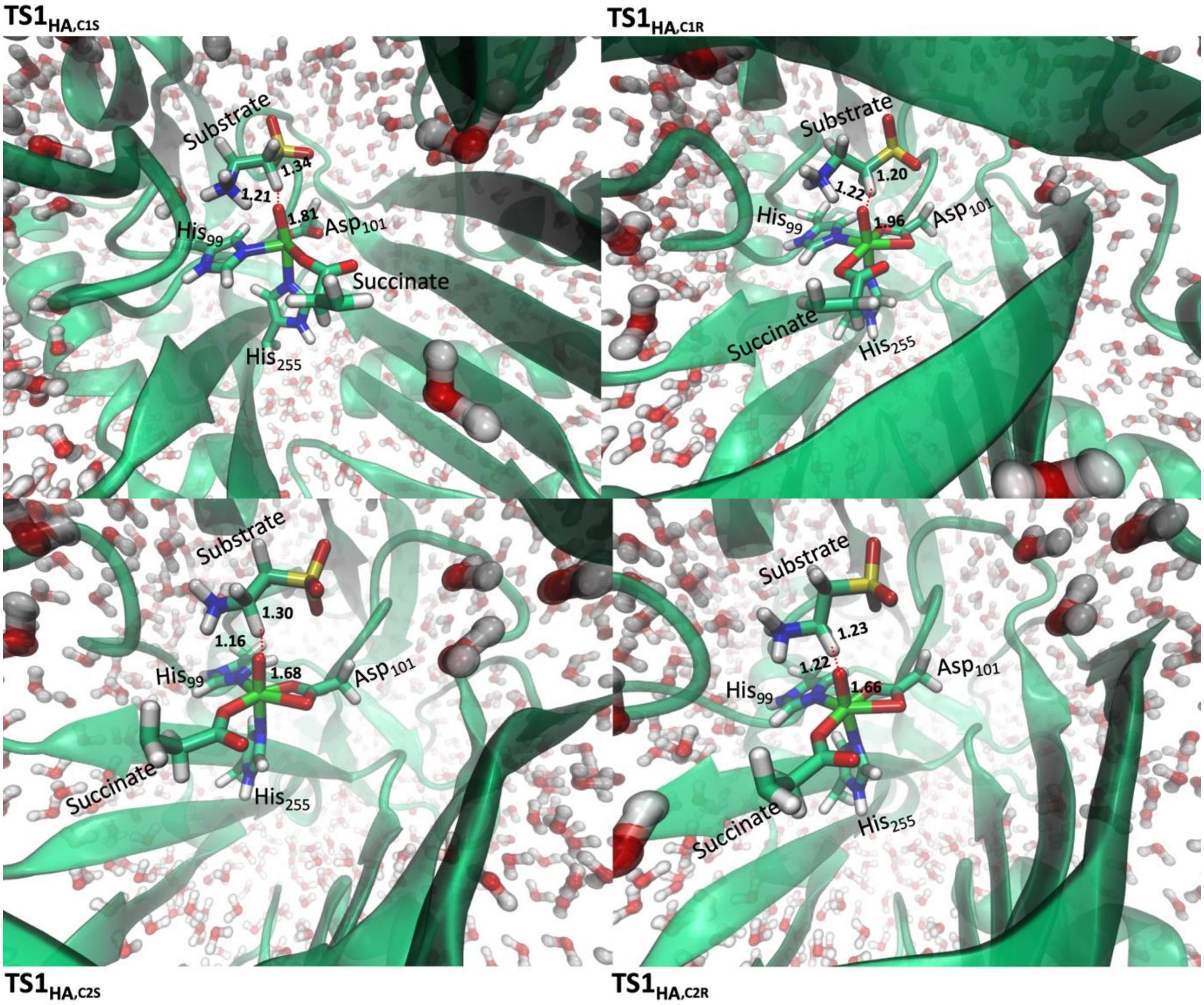

Figure S4: QM/MM optimized geometries of the transition states for the hydrogen atom (HA) abstraction from the C^1 and C^2 positions of the substrate. Distances are given in Å.

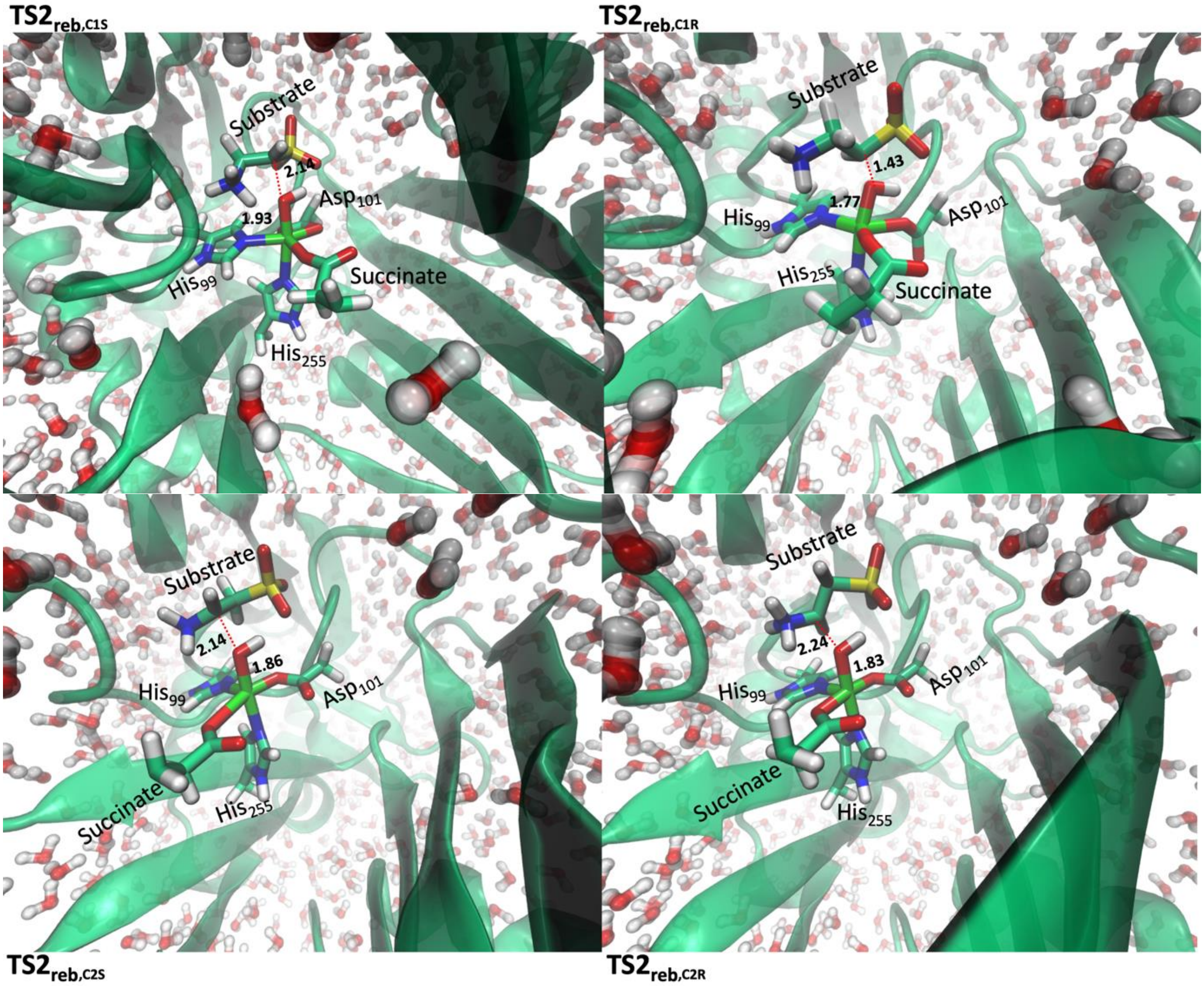

Figure S5: QM/MM optimized geometries of transition states for OH rebound from the iron(III)-hydroxo complexes to the C^1 or C^2 positions of the radical. Distances are given in Å.

Model A data:

Table S1: Absolute (free) energies (in au) of the optimized geometries for the hydroxylation reaction of taurine by the iron(IV)-oxo model ^{5}**Re**$_A$ of TauD after a geometry optimization at the UB3LYP/6-31G* level of theory.

System	BS1			BS2+Solv+Dispersion		
	E	E+ZPE	G	Edisp	Edisp +ZPE	Edisp + G
^{5}**Re**$_A$	-2439.153063	-2438.578252	-2438.659356	-2439.94723	-2439.37242	-2439.45352
^{5}**TS1**$_{HA,C1S,A}$	-2439.135828	-2438.568324	-2438.647720	-2439.91286	-2439.34535	-2439.42475
^{5}**TS1**$_{HA,C1R,A}$	-2439.151445	-2438.584921	-2438.665249	-2439.93309	-2439.36656	-2439.44689
^{5}**TS1**$_{HA,C2S,A}$	-2439.121086	-2438.555617	-2438.636685	-2439.91598	-2439.35051	-2439.43158
^{5}**TS1**$_{HA,C2R,A}$	-2439.122092	-2438.557330	-2438.640065	-2439.91163	-2439.34687	-2439.42961
^{5}**IM1**$_{HA,C1S,A}$	-2439.178161	-2438.608043	-2438.690790	-2439.96084	-2439.39072	-2439.47347
^{5}**IM1**$_{HA,C1R,A}$	-2439.183664	-2438.612978	-2438.693333	-2439.96380	-2439.39311	-2439.47347
^{5}**IM1**$_{HA,C2S,A}$	-2439.175196	-2438.604834	-2438.686705	-2439.95711	-2439.38675	-2439.46862
^{5}**IM1**$_{HA,C2R,A}$	-2439.182553	-2438.613084	-2438.695239	-2439.95977	-2439.39031	-2439.47246

Table S2: Relative (free) energies (in kcal mol^{-1}) of the optimized geometries for the hydroxylation reaction of taurine by the iron(IV)-oxo model ^{5}**Re**$_A$ of TauD after a geometry optimization at the UB3LYP/6-31G* level of theory.

System	BS1			BS2+Solv+Dispersion		
	ΔE	ΔE+ZPE	ΔG	ΔEdisp	ΔEdisp +ZPE	ΔEdisp + G
^{5}**Re**$_A$	0.00	0.00	0.00	0.00	0.00	0.00
^{5}**TS1**$_{HA,C1S,A}$	10.82	6.23	7.30	21.57	16.99	18.06
^{5}**TS1**$_{HA,C1R,A}$	1.02	-4.18	-3.70	8.87	3.67	4.16
^{5}**TS1**$_{HA,C2S,A}$	20.07	14.20	14.23	19.61	13.75	13.77
^{5}**TS1**$_{HA,C2R,A}$	19.43	13.13	12.11	22.34	16.03	15.01
^{5}**IM1**$_{HA,C1S,A}$	-15.75	-18.69	-19.72	-8.54	-11.48	-12.51
^{5}**IM1**$_{HA,C1R,A}$	-19.20	-21.79	-21.32	-10.40	-12.99	-12.52
^{5}**IM1**$_{HA,C2S,A}$	-13.89	-16.68	-17.16	-6.20	-8.99	-9.47
^{5}**IM1**$_{HA,C2R,A}$	-18.51	-21.86	-22.52	-7.87	-11.22	-11.88

Table S3: Group spin densities of the optimized geometries for the hydroxylation reaction of taurine by the iron(IV)-oxo model ^{5}**Re**$_{\mathbf{A}}$ of TauD after a geometry optimization at the UB3LYP/6-31G* level of theory.

System		Fe	O	Substrate	Succinate	Amino Acids	Total
$^{5}\mathbf{Re}_{\mathbf{A}}$	Gas	3.079	0.542	0.010	0.153	0.215	4.00
$^{5}\mathbf{TS1}_{\mathbf{HA,C1S,A}}$	Gas	2.856	0.434	0.537	0.065	0.108	4.00
$^{5}\mathbf{TS1}_{\mathbf{HA,C1R,A}}$	Gas	3.913	0.058	-0.460	0.146	0.343	4.00
$^{5}\mathbf{TS1}_{\mathbf{HA,C2S,A}}$	Gas	3.969	-0.030	-0.439	0.125	0.374	4.00
$^{5}\mathbf{TS1}_{\mathbf{HA,C2R,A}}$	Gas	3.977	-0.039	-0.439	0.143	0.359	4.00
$^{5}\mathbf{IM1}_{\mathbf{HA,C1S,A}}$	Gas	4.068	0.313	-0.983	0.209	0.393	4.00
$^{5}\mathbf{IM1}_{\mathbf{HA,C1R,A}}$	Gas	4.064	0.315	-0.987	0.209	0.399	4.00
$^{5}\mathbf{IM1}_{\mathbf{HA,C2S,A}}$	Gas	4.035	0.462	-0.988	0.224	0.267	4.00
$^{5}\mathbf{IM1}_{\mathbf{HA,C2R,A}}$	Gas	4.071	0.361	-0.992	0.181	0.379	4.00

Table S4: Group charges of the optimized geometries for the hydroxylation reaction of taurine by the iron(IV)-oxo model ^{5}**Re**$_{\mathbf{A}}$ of TauD after a geometry optimization at the UB3LYP/6-31G* level of theory.

System		Fe	O	Substrate	Succinate	Amino Acids	Total
$^{5}\mathbf{Re}_{\mathbf{A}}$		0.644	-0.483	-0.046	-0.294	0.180	0.00
$^{5}\mathbf{TS1}_{\mathbf{HA,C1S,A}}$		0.695	-0.611	0.298	-0.485	0.103	0.00
$^{5}\mathbf{TS1}_{\mathbf{HA,C1R,A}}$		0.679	-0.644	0.301	-0.409	0.073	0.00
$^{5}\mathbf{TS1}_{\mathbf{HA,C2S,A}}$		0.831	-0.672	0.248	-0.493	0.086	0.00
$^{5}\mathbf{TS1}_{\mathbf{HA,C2R,A}}$		0.868	-0.678	0.202	-0.479	0.088	0.00
$^{5}\mathbf{IM1}_{\mathbf{HA,C1S,A}}$		0.718	-0.829	0.429	-0.398	0.081	0.00
$^{5}\mathbf{IM1}_{\mathbf{HA,C1R,A}}$		0.691	-0.819	0.436	-0.392	0.084	0.00
$^{5}\mathbf{IM1}_{\mathbf{HA,C2S,A}}$		0.704	-0.777	0.468	-0.386	-0.009	0.00
$^{5}\mathbf{IM1}_{\mathbf{HA,C2R,A}}$		0.867	-0.784	0.369	-0.468	0.015	0.00

Model B data:

Table S5: Absolute (free) energies (in au) of the optimized geometries for the hydroxylation reaction of taurine by the iron(IV)-oxo model ^{5,3}**Re$_B$** of TauD after a geometry optimization at the UB3LYP/6-31G* level of theory.

System	BS1			BS2+Solv+Dispersion		
	E	E+ZPE	G	Edisp	Edisp +ZPE	Edisp + G
^{1}**Re$_B$**	-6005.941169	-6003.871804	-6004.088042	-6007.94141	-6005.87204	-6006.08828
^{3}**Re$_B$**	-6005.955052	-6003.884848	-6004.101942	-6007.95628	-6005.88607	-6006.10317
^{7}**Re$_B$**	-6005.940829	-6003.874106	-6004.089955	-6007.95210	-6005.88538	-6006.10123
^{5}**Re$_B$**	-6005.955663	-6003.888681	-6004.110937	-6007.96968	-6005.90269	-6006.12495
^{5}**TS1$_{HA,C1S,B}$**	-6005.922456	-6003.863472	-6004.081885	-6007.94293	-6005.88395	-6006.10236
^{5}**TS1$_{HA,C1R,B}$**	-6005.925929	-6003.866331	-6004.083570	-6007.94282	-6005.88323	-6006.10047
^{5}**TS1$_{HA,C2S,B}$**	-6005.934965	-6003.874580	-6004.090701	-6007.94833	-6005.88795	-6006.10407
^{5}**TS1$_{HA,C2R,B}$**	-6005.939841	-6003.879133	-6004.095569	-6007.94606	-6005.88535	-6006.10179
^{5}**IM1$_{HA,C1,B,\sigma}$**	-6005.965763	-6003.900851	-6004.119750	-6007.98167	-6005.91676	-6006.13566
^{5}**IM1$_{HA,C1,B,\pi}$**	-6005.943682	-6003.877470	-6004.094649	-6007.95711	-6005.89089	-6006.10807
^{5}**IM1$_{HA,C2,B,\sigma}$**	-6005.963656	-6003.899321	-6004.115469	-6007.97997	-6005.91564	-6006.13178
^{5}**IM1$_{HA,C2,B,\pi}$**	-6005.953390	-6003.889379	-6004.107382	-6007.95478	-6005.89077	-6006.10877
^{5}**TS2$_{reb,C1S,B,\pi}$**	-6005.938952	-6003.873345	-6004.088462	-6007.94835	-6005.88274	-6006.09786
^{5}**TS2$_{reb,C1R,B,\pi}$**	-6005.936778	-6003.871187	-6004.086647	-6007.94828	-6005.88268	-6006.09814
^{5}**TS2$_{reb,C2S,B,\pi}$**	-6005.927993	-6003.863546	-6004.077971	-6007.93029	-6005.86584	-6006.08027
^{5}**TS2$_{reb,C2R,B,\pi}$**	-6005.947257	-6003.885780	-6004.100390	-6007.94424	-6005.88276	-6006.09737
^{5}**Prod$_{hydro,C1S,B}$**	-6006.066161	-6003.995727	-6004.211307	-6008.05926	-6005.98882	-6006.20440
^{5}**Prod$_{hydro,C1R,B}$**	-6006.041491	-6003.971571	-6004.190537	-6008.04449	-6005.97457	-6006.19354
^{5}**Prod$_{hydro,C2S,B}$**	-6006.068321	-6003.996772	-6004.213134	-6008.05260	-6005.98105	-6006.19741
^{5}**Prod$_{hydro,C2R,B}$**	-6006.063757	-6003.993361	-6004.212322	-6008.04603	-6005.97563	-6006.19459

Table S6: Relative (free) energies (in kcal mol^{-1}) of the optimized geometries for the hydroxylation reaction of taurine by the iron(IV)-oxo model ^{5,3}**Re$_B$** of TauD after a geometry optimization at the UB3LYP/6-31G* level of theory.

System	BS1			BS2+Solv+Dispersion		
	ΔE	ΔE+ZPE	ΔG	ΔEdisp	ΔEdisp +ZPE	ΔEdisp + G
^{1}**Re$_B$**	9.10	10.59	14.37	17.74	19.23	23.01
^{3}**Re$_B$**	0.38	2.41	5.64	8.41	10.43	13.67
^{7}**Re$_B$**	9.31	9.15	13.17	11.03	10.86	14.88
^{5}**Re$_B$**	0.00	0.00	0.00	0.00	0.00	0.00
^{5}**TS1$_{HA,C1S,B}$**	20.84	15.82	18.23	16.78	11.76	14.17
^{5}**TS1$_{HA,C1R,B}$**	18.66	14.02	17.17	16.85	12.22	15.36
^{5}**TS1$_{HA,C2S,B}$**	12.99	8.85	12.70	13.39	9.26	13.10
^{5}**TS1$_{HA,C2R,B}$**	9.93	5.99	9.64	14.82	10.88	14.53
^{5}**IM1$_{HA,C1,B,\sigma}$**	-6.34	-7.64	-5.53	-7.53	-8.83	-6.72
^{5}**IM1$_{HA,C1,B,\pi}$**	7.52	7.03	10.22	7.89	7.40	10.59
^{5}**IM1$_{HA,C2,B,\sigma}$**	-5.02	-6.68	-2.84	-6.46	-8.12	-4.29
^{5}**IM1$_{HA,C2,B,\pi}$**	1.43	-0.44	2.23	9.35	7.48	10.15
^{5}**TS2$_{reb,C1S,B,\pi}$**	10.49	9.62	14.10	13.38	12.52	17.00
^{5}**TS2$_{reb,C1R,B,\pi}$**	11.85	10.98	15.24	13.43	12.56	16.82
^{5}**TS2$_{reb,C2S,B,\pi}$**	17.36	15.77	20.69	24.72	23.13	28.04
^{5}**TS2$_{reb,C2R,B,\pi}$**	5.27	1.82	6.62	15.96	12.51	17.30
^{5}**Prod$_{hydro,C1S,B}$**	-69.34	-67.17	-62.98	-56.21	-54.05	-49.86
^{5}**Prod$_{hydro,C1R,B}$**	-53.86	-52.01	-49.95	-46.95	-45.11	-43.04
^{5}**Prod$_{hydro,C2S,B}$**	-70.69	-67.83	-64.13	-52.03	-49.17	-45.47
^{5}**Prod$_{hydro,C2R,B}$**	-67.83	-65.69	-63.62	-47.91	-45.77	-43.70

Table S7: Group spin densities of optimized geometries for the hydroxylation reaction of taurine by the iron(IV)-oxo model ^{5}**Re$_B$** of TauD after a geometry optimization at the UB3LYP/6-31G* level of theory.

System		Fe	O	Substrate	Amino Acids	Total
^{1}**Re$_B$**		0.446	-0.413	0.000	-0.033	0.00
^{3}**Re$_B$**		1.195	0.866	0.000	-0.060	2.00
^{7}**Re$_B$**		4.009	1.357	0.000	0.635	6.00
^{5}**Re$_B$**		3.033	0.666	0.000	0.299	4.00
^{5}**TS1$_{HA,C1S,B}$**		3.940	0.005	-0.436	0.491	4.00
^{5}**TS1$_{HA,C1R,B}$**		3.940	0.005	-0.436	0.491	4.00
^{5}**TS1$_{HA,C2S,B}$**		3.951	-0.002	-0.427	0.478	4.00
^{5}**TS1$_{HA,C2R,B}$**		3.910	0.123	-0.497	0.463	4.00
^{5}**IM1$_{HA,C1,B,\sigma}$**		4.077	0.337	-0.984	0.569	4.00
^{5}**IM1$_{HA,C1,B,\pi}$**		2.816	0.051	0.988	0.145	4.00
^{5}**IM1$_{HA,C2,B,\sigma}$**		4.077	0.344	-0.979	0.558	4.00
^{5}**IM1$_{HA,C2,B,\pi}$**		2.813	0.037	0.974	0.175	4.00
^{5}**TS2$_{reb,C1S,B,\pi}$**		3.592	-0.143	0.323	0.228	4.00
^{5}**TS2$_{reb,C1R,B,\pi}$**		3.260	-0.220	0.803	0.156	4.00
^{5}**TS2$_{reb,C2S,B,\pi}$**		3.639	-0.061	0.154	0.267	4.00
^{5}**TS2$_{reb,C2R,B,\pi}$**		3.617	-0.121	0.308	0.196	4.00
^{5}**Prod$_{hydro,C1S,B}$**		3.752	0.000	0.000	0.248	4.00
^{5}**Prod$_{hydro,C1R,B}$**		3.744	0.001	0.000	0.255	4.00
^{5}**Prod$_{hydro,C2S,B}$**		3.763	0.008	0.003	0.227	4.00
^{5}**Prod$_{hydro,C2R,B}$**		3.753	0.000	0.000	0.246	4.00

Table S8: Group charges of optimized geometries for the hydroxylation reaction of taurine by the iron(IV)-oxo model ^{5}**Re**$_{\mathrm{B}}$ of TauD after a geometry optimization at the UB3LYP/6-31G* level of theory.

System	Fe	O	Substrate	Amino Acids	Total
^{1}**Re**$_{\mathrm{B}}$	0.569	-0.418	-0.014	-0.137	0.00
^{3}**Re**$_{\mathrm{B}}$	0.565	-0.423	-0.013	-0.129	0.00
^{7}**Re**$_{\mathrm{B}}$	0.833	-0.415	-0.023	-0.394	0.00
^{5}**Re**$_{\mathrm{B}}$	0.613	-0.410	0.004	-0.208	0.00
^{5}**TS1**$_{\mathrm{HA,C1S,B}}$	0.815	-0.679	0.251	-0.387	0.00
^{5}**TS1**$_{\mathrm{HA,C1R,B}}$	0.815	-0.679	0.251	-0.387	0.00
^{5}**TS1**$_{\mathrm{HA,C2S,B}}$	0.808	-0.665	0.218	-0.361	0.00
^{5}**TS1**$_{\mathrm{HA,C2R,B}}$	0.820	-0.693	0.259	-0.385	0.00
^{5}**IM1**$_{\mathrm{HA,C1,B,}\sigma}$	0.813	-0.818	0.430	-0.425	0.00
^{5}**IM1**$_{\mathrm{HA,C1,B,}\pi}$	0.648	-0.736	0.405	-0.317	0.00
^{5}**IM1**$_{\mathrm{HA,C2,B,}\sigma}$	0.791	-0.822	0.435	-0.403	0.00
^{5}**IM1**$_{\mathrm{HA,C2,B,}\pi}$	0.654	-0.715	0.371	-0.310	0.00
^{5}**TS2**$_{\mathrm{reb,C1S,B,}\pi}$	0.655	-0.699	0.594	-0.549	0.00
^{5}**TS2**$_{\mathrm{reb,C1R,B,}\pi}$	0.642	-0.700	0.484	-0.426	0.00
^{5}**TS2**$_{\mathrm{reb,C2S,B,}\pi}$	0.665	-0.742	0.585	-0.508	0.00
^{5}**TS2**$_{\mathrm{reb,C2R,B,}\pi}$	0.618	-0.719	0.684	-0.583	0.00
^{5}**Prod**$_{\mathrm{hydro,C1S,B}}$	0.672	-0.669	0.610	-0.613	0.00
^{5}**Prod**$_{\mathrm{hydro,C1R,B}}$	0.681	-0.612	0.574	-0.644	0.00
^{5}**Prod**$_{\mathrm{hydro,C2S,B}}$	0.630	-0.648	0.662	-0.644	0.00
^{5}**Prod**$_{\mathrm{hydro,C2R,B}}$	0.645	-0.652	0.568	-0.561	0.00

Table S9: Calculated KIE effects on the transition states of model **B** using the Eyring and Wigner models for the primary (second column), secondary (third column) isotope effects as well as for taurine-d$_2$ substrate (fourth column).

System	D_aH_b	H_aD_b	D_aD_b
**KIE**$_{\mathrm{Eyring,C1S}}$	1.07	9.31	10.08
**KIE**$_{\mathrm{Eyring,C1R}}$	8.44	1.12	9.57
**KIE**$_{\mathrm{Eyring,C2S}}$	8.47	1.06	9.11
**KIE**$_{\mathrm{Eyring,C2R}}$	1.10	9.63	10.82
**KIE**$_{\mathrm{Wigner,C1S}}$	1.07	13.61	14.81
**KIE**$_{\mathrm{Wigner,C1R}}$	12.16	1.12	13.86
**KIE**$_{\mathrm{Wigner,C2S}}$	11.92	1.04	12.91
**KIE**$_{\mathrm{Wigner,C2R}}$	1.60	9.72	11.14

Test calculations with an alternative basis set:

Table S10: Absolute (free) energies (in au) of the optimized geometries for the hydroxylation reaction of taurine by the iron(IV)-oxo model ^{5}**Re$_B$** of TauD after a geometry optimization at the UB3LYP/6-31G (BS3) level of theory.

System	BS3			BS2+Solv+Dispersion		
	E	E+ZPE	G	E	E+ZPE	G
^{3}**Re$_B$**	-6004.421280	-6002.342784	-6002.558966	-6007.882189	-6005.803693	-6006.019875
^{5}**Re$_B$**	-6004.430416	-6002.353309	-6002.570746	-6007.897978	-6005.820871	-6006.038308
^{5}**TS1$_{HA,C1S,B}$**	-6004.394650	-6002.326974	-6002.541659	-6007.867403	-6005.799727	-6006.014412
^{5}**TS1$_{HA,C1R,B}$**	-6004.393232	-6002.325980	-6002.536518	-6007.866287	-6005.799035	-6006.009573
^{5}**TS1$_{HA,C2S,B}$**	-6004.409599	-6002.340783	-6002.554129	-6007.871120	-6005.802304	-6006.015650
^{5}**TS1$_{HA,C2R,B}$**	-6004.417368	-6002.347439	-6002.559746	-6007.867856	-6005.797927	-6006.010234
^{5}**IM1$_{HA,C1S,B}$**	-6004.417086	-6002.345140	-6002.557838	-6007.891120	-6005.819174	-6006.031872
^{5}**IM1$_{HA,C1R,B}$**	-6004.405938	-6002.331379	-6002.544870	-6007.880413	-6005.805854	-6006.019345
^{5}**TS2$_{reb,C1S,B}$**	-6004.411947	-6002.338165	-6002.551150	-6007.885723	-6005.811941	-6006.024926
^{5}**TS2$_{reb,C1R,B}$**	-6004.394134	-6002.320558	-6002.532579	-6007.885723	-6005.812147	-6006.024168
^{5}**Prod$_{hydro,C1S,B}$**	-6004.528068	-6002.450317	-6002.665982	-6007.974951	-6005.897200	-6006.112865
^{5}**Prod$_{hydro,C1R,B}$**	-6004.528068	-6002.450317	-6002.665982	-6007.974951	-6005.897200	-6006.112865
^{5}**Prod$_{hydro,C2S,B}$**	-6004.486877	-6002.415446	-6002.631190	-6007.914286	-6005.842855	-6006.058599
^{5}**Prod$_{hydro,C2R,B}$**	-6004.459839	-6002.387280	-6002.607778	-6007.884980	-6005.812421	-6006.032919

Table S11: Relative (free) energies (in kcal mol^{-1}) of the optimized geometries for the hydroxylation reaction of taurine by the iron(IV)-oxo model ^{5}**Re$_B$** of TauD after a geometry optimization at the UB3LYP/6-31G (BS3) level of theory.

System	BS3			BS2+Solv+Dispersion		
	ΔE	ΔE+ZPE	ΔG	ΔE	ΔE+ZPE	ΔG
^{3}**Re$_B$**	5.73	6.60	7.39	9.91	10.78	11.57
^{5}**Re$_B$**	0.00	0.00	0.00	0.00	0.00	0.00
^{5}**TS1$_{HA,C1S,B}$**	22.44	16.53	18.25	19.19	13.27	15.00
^{5}**TS1$_{HA,C1R,B}$**	23.33	17.15	21.48	19.89	13.70	18.03
^{5}**TS1$_{HA,C2S,B}$**	13.06	7.86	10.43	16.85	11.65	14.22
^{5}**TS1$_{HA,C2R,B}$**	8.19	3.68	6.90	18.90	14.40	17.62
^{5}**IM1$_{HA,C1S,B}$**	8.37	5.13	8.10	4.30	1.06	4.04
^{5}**IM1$_{HA,C1R,B}$**	15.36	13.76	16.24	11.02	9.42	11.90
^{5}**TS2$_{reb,C1S,B}$**	11.59	9.50	12.30	16.44	14.35	17.14
^{5}**TS2$_{reb,C1R,B}$**	22.77	20.55	23.95	24.33	22.11	25.51
^{5}**Prod$_{hydro,C1S,B}$**	-61.28	-60.87	-59.76	-48.30	-47.90	-46.78
^{5}**Prod$_{hydro,C1R,B}$**	-61.28	-60.87	-59.76	-48.30	-47.90	-46.78
^{5}**Prod$_{hydro,C2S,B}$**	-35.43	-38.99	-37.93	-10.23	-13.79	-12.73
^{5}**Prod$_{hydro,C2R,B}$**	-18.46	-21.32	-23.24	8.16	5.30	3.38

Table S12: Group spin densities of optimized geometries for the hydroxylation reaction of taurine by the iron(IV)-oxo model ^{5}**Re**$_{B}$ of TauD after a geometry optimization at the UB3LYP/6-31G (BS3) level of theory.

System		Fe	O	Substrate	Amino Acids	Total
^{3}**Re**$_{B}$		1.128	0.934	0.000	-0.061	2.00
^{5}**Re**$_{B}$		2.986	0.737	0.000	0.280	4.00
^{5}**TS1**$_{HA,C1S,B}$		3.914	0.155	-0.542	0.472	4.00
^{5}**TS1**$_{HA,C1R,B}$		3.934	0.109	-0.527	0.483	4.00
^{5}**TS1**$_{HA,C2S,B}$		3.921	0.067	-0.450	0.463	4.00
^{5}**TS1**$_{HA,C2R,B}$		3.905	0.131	-0.494	0.459	4.00
^{5}**IM1**$_{HA,C1S,B}$		4.070	0.369	-0.985	0.546	4.00
^{5}**IM1**$_{HA,C1R,B}$		2.829	0.003	0.991	0.176	4.00
^{5}**TS2**$_{reb,C1S,B}$		4.008	0.270	-0.745	0.467	4.00
^{5}**TS2**$_{reb,C1R,B}$		3.127	-0.141	0.843	0.171	4.00
^{5}**Prod**$_{hydro,C1S,B}$		3.756	0.001	0.000	0.244	4.00
^{5}**Prod**$_{hydro,C1R,B}$		3.756	0.001	0.000	0.244	4.00
^{5}**Prod**$_{hydro,C2S,B}$		4.076	0.339	-0.972	0.557	4.00
^{5}**Prod**$_{hydro,C2R,B}$		2.803	0.027	0.976	0.193	4.00

Table S13: Group charges of optimized geometries for the hydroxylation reaction of taurine by the iron(IV)-oxo model ^{5}**Re**$_{B}$ of TauD after a geometry optimization at the UB3LYP/6-31G (BS3) level of theory.

System		Fe	O	Substrate	Amino Acids	Total
^{3}**Re**$_{B}$		0.725	-0.354	-0.024	-0.346	0.00
^{5}**Re**$_{B}$		0.525	-0.348	-0.023	-0.153	0.00
^{5}**TS1**$_{HA,C1S,B}$		0.888	-0.658	0.238	-0.468	0.00
^{5}**TS1**$_{HA,C1R,B}$		0.879	-0.651	0.208	-0.435	0.00
^{5}**TS1**$_{HA,C2S,B}$		0.858	-0.617	0.169	-0.411	0.00
^{5}**TS1**$_{HA,C2R,B}$		0.874	-0.638	0.212	-0.448	0.00
^{5}**IM1**$_{HA,C1S,B}$		0.867	-0.722	0.289	-0.434	0.00
^{5}**IM1**$_{HA,C1R,B}$		0.716	-0.676	0.343	-0.383	0.00
^{5}**TS2**$_{reb,C1S,B}$		0.824	-0.661	0.357	-0.519	0.00
^{5}**TS2**$_{reb,C1R,B}$		0.761	-0.620	0.394	-0.536	0.00
^{5}**Prod**$_{hydro,C1S,B}$		0.811	-0.594	0.523	-0.741	0.00
^{5}**Prod**$_{hydro,C1R,B}$		0.811	-0.594	0.523	-0.741	0.00
^{5}**Prod**$_{hydro,C2S,B}$		0.872	-0.768	0.357	-0.461	0.00
^{5}**Prod**$_{hydro,C2R,B}$		0.706	-0.633	0.306	-0.379	0.00

Test calculations with a solvent model included during the geometry optimizations:

Table S14: Absolute (free) energies (in au) of the optimized geometries for the hydroxylation reaction of taurine by the iron(IV)-oxo model ^{5}**Re$_{B}$** of TauD after a geometry optimization at the UB3LYP/6-31G*+solvent (BS4) and UB3LYP+GD3/6-31G* (BS5) level of theory.

System	BS4			BS5		
	E	E+ZPE	G	E	E+ZPE	G
^{5}**Re$_{B}$**	-6004.50553	-6002.43297	-6002.65361	-6006.373865	-6004.295434	-6004.488240
^{5}**TS1$_{HA,C1R,B}$**	-6004.46866	-6002.40581	-6002.62011	-6006.215261	-6004.165742	-6004.366890
^{5}**TS1$_{HA,C2R,B}$**	-6004.48408	-6002.41907	-6002.63633	-6006.358733	-6004.287194	-6004.477865

Table S15: Relative (free) energies (in kcal mol^{-1}) of optimized geometries for the hydroxylation reaction of taurine by the iron(IV)-oxo model ^{5}**Re$_{B}$** of TauD after a geometry optimization at the UB3LYP/6-31G*+solvent (BS4) and UB3LYP+GD3/6-31G* (BS5) level of theory.

System	BS4			BS5		
	ΔE	ΔE+ZPE	ΔG	ΔEdisp	ΔEdisp +ZPE	ΔEdisp + G
^{5}**Re$_{B}$**	0.00	0.00	0.00	0.00	0.00	0.00
^{5}**TS1$_{HA,C1R,B}$**	23.14	17.05	21.03	17.74	19.23	23.01
^{5}**TS1$_{HA,C2R,B}$**	13.46	8.73	10.85	9.50	5.17	6.51

Test calculations of model B with groups Asp_{94}, Asn_{95}, Ser_{158} and Phe_{159} removed.

Table S16: Absolute (free) energies (in au) of the optimized geometries for the hydroxylation reaction of taurine by the iron(IV)-oxo model ^{5,3}**Re**$_{B2}$ of TauD after a geometry optimization at the UB3LYP/6-31G* level of theory.

System	BS1			BS2+Solv+Dispersion		
	E	E+ZPE	G	Edisp	Edisp +ZPE	Edisp + G
^{5}**Re**$_{B2}$	-4688.475364	-4686.855318	-4687.041626	-4690.035846	-4688.415800	-4688.602108
^{5}**TS1**$_{HA,C1S,B2}$	-4688.412945	-4686.802899	-4686.983078	-4689.989307	-4688.379261	-4688.559440
^{5}**TS1**$_{HA,C1R,B2}$	-4688.416333	-4686.805562	-4686.983543	-4689.990634	-4688.379863	-4688.557844
^{5}**TS1**$_{HA,C2S,B2}$	-4688.425104	-4686.813446	-4686.992035	-4689.996297	-4688.384639	-4688.563228
^{5}**TS1**$_{HA,C2R,B2}$	-4688.415926	-4686.804696	-4686.983024	-4689.986337	-4688.375107	-4688.553435

Table S17: Relative (free) energies (in kcal mol^{-1}) of the optimized geometries for the hydroxylation reaction of taurine by the iron(IV)-oxo model ^{5,3}**Re**$_{B2}$ of TauD after a geometry optimization at the UB3LYP/6-31G* level of theory.

System	BS1			BS2+Solv+Dispersion		
	ΔE	ΔE+ZPE	ΔG	ΔEdisp	ΔEdisp +ZPE	ΔEdisp + G
^{5}**Re**$_{B2}$	0.00	0.00	0.00	0.00	0.00	0.00
^{5}**TS1**$_{HA,C1S,B2}$	39.17	32.89	36.74	29.20	22.93	26.77
^{5}**TS1**$_{HA,C1R,B2}$	37.04	31.22	36.45	28.37	22.55	27.78
^{5}**TS1**$_{HA,C2S,B2}$	31.54	26.28	31.12	24.82	19.55	24.40
^{5}**TS1**$_{HA,C2R,B2}$	37.30	31.77	36.77	31.07	25.54	30.54

Model C data:

Table S18: Absolute (free) energies (in au) of the optimized geometries for the hydroxylation reaction of taurine by the iron(IV)-oxo model ^{5,3}**Re**$_{C}$ of TauD after a geometry optimization at the UB3LYP/6-31G* level of theory.

System	BS1			BS2+Solv+Dispersion		
	E	E+ZPE	G	Edisp	Edisp +ZPE	Edisp + G
^{5}**Re**$_{C}$	-7323.852057	-7321.520987	-7321.758426	-7326.398903	-7324.067833	-7324.305272
^{5}**TS1**$_{HA,C1S,C}$	-7323.815188	-7321.492907	-7321.727593	-7326.366935	-7324.044654	-7324.279340
^{5}**TS1**$_{HA,C1R,C}$	-7323.810712	-7321.487976	-7321.723412	-7326.365351	-7324.042615	-7324.278051
^{5}**TS1**$_{HA,C2S,C}$	-7323.807884	-7321.483482	-7321.715358	-7326.355382	-7324.030980	-7324.262856
^{5}**TS1**$_{HA,C2R,C}$	-7323.822312	-7321.499131	-7321.724857	-7326.357211	-7324.034030	-7324.259756
^{5}**IM1**$_{HA,C1,C}$	-7323.843164	-7321.515806	-7321.749864	-7326.395059	-7324.067701	-7324.301759
^{5}**IM1**$_{HA,C2,C}$	-7323.869200	-7321.542091	-7321.774620	-7326.408818	-7324.081709	-7324.314238

Table S19: Relative (free) energies (in kcal mol^{-1}) of the optimized geometries for the hydroxylation reaction of taurine by the iron(IV)-oxo model ^{5,3}**Re**$_{C}$ of TauD after a geometry optimization at the UB3LYP/6-31G* level of theory.

System	BS1			BS2+Solv+Dispersion		
	ΔE	ΔE+ZPE	ΔG	ΔEdisp	ΔEdisp +ZPE	ΔEdisp + G
^{5}**Re**$_{C}$	0.00	0.00	0.00	0.00	0.00	0.00
^{5}**TS1**$_{HA,C1S,C}$	23.14	17.62	19.35	20.06	14.54	16.27
^{5}**TS1**$_{HA,C1R,C}$	25.94	20.71	21.97	21.05	15.82	17.08
^{5}**TS1**$_{HA,C2S,C}$	27.72	23.53	27.03	27.31	23.13	26.62
^{5}**TS1**$_{HA,C2R,C}$	18.67	13.72	21.07	26.16	21.21	28.56
^{5}**IM1**$_{HA,C1,C}$	5.58	3.25	5.37	2.41	0.08	2.20
^{5}**IM1**$_{HA,C2,C}$	-10.76	-13.24	-10.16	-6.22	-8.71	-5.63

Table S20: Group spin densities of optimized geometries for the hydroxylation reaction of taurine by the iron(IV)-oxo model ^{5}**Re**$_{C}$ of TauD after a geometry optimization at the UB3LYP/6-31G* level of theory.

System		Fe	O	Substrate	Amino Acids	Total
^{5}**Re**$_{C}$		3.005	0.693	0.006	0.296	4.00
^{5}**TS1**$_{HA,C1S,C}$		3.752	0.118	-0.317	0.447	4.00
^{5}**TS1**$_{HA,C1R,C}$		3.974	-0.101	-0.418	0.546	4.00
^{5}**TS1**$_{HA,C2S,C}$		2.887	0.610	0.293	0.211	4.00
^{5}**TS1**$_{HA,C2R,C}$		3.784	0.134	-0.359	0.441	4.00
^{5}**IM1**$_{HA,C1,C}$		4.070	0.351	-0.980	0.559	4.00
^{5}**IM1**$_{HA,C2,C}$		4.085	0.295	-0.982	0.602	4.00

Table S21: Group charges of optimized geometries for the hydroxylation reaction of taurine by the iron(IV)-oxo model $^{5}\mathbf{Re_{C}}$ of TauD after a geometry optimization at the UB3LYP/6-31G* level of theory.

System		Fe	O	Substrate	Amino Acids	Total
$^{5}\mathbf{Re_{C}}$		0.737	-0.447	0.008	0.701	1.00
$^{5}\mathbf{TS1_{HA,C1S,C}}$		0.827	-0.653	0.184	0.642	1.00
$^{5}\mathbf{TS1_{HA,C1R,C}}$		0.822	-0.686	0.268	0.596	1.00
$^{5}\mathbf{TS1_{HA,C2S,C}}$		0.699	-0.576	0.178	0.698	1.00
$^{5}\mathbf{TS1_{HA,C2R,C}}$		0.830	-0.640	0.215	0.596	1.00
$^{5}\mathbf{IM1_{HA,C1,C}}$		0.840	-0.820	0.450	0.530	1.00
$^{5}\mathbf{IM1_{HA,C2,C}}$		0.826	-0.831	0.449	0.556	1.00

Model C2 data, i.e. model C with His_{70} singly protonated:

Table S22: Absolute (free) energies (in au) of the optimized geometries for the hydroxylation reaction of taurine by the iron(IV)-oxo model ^{5,3}**Re$_{C2}$** of TauD after a geometry optimization at the UB3LYP/6-31G* level of theory.

System	BS1			BS2+Solv+Dispersion		
	E	E+ZPE	G	Edisp	Edisp +ZPE	Edisp + G
^{5}**Re$_{C2}$**	-7323.3724	-7321.056402	-7321.286875	-7325.92193	-7323.60591	-7323.83638
^{5}**TS1$_{HA,C1S,C2}$**	-7323.3386	-7321.022883	-7321.253087	-7325.881009	-7323.56525	-7323.79546
^{5}**TS1$_{HA,C1R,C2}$**	-7323.3238	-7321.016333	-7321.243581	-7325.867247	-7323.55982	-7323.78707
^{5}**TS1$_{HA,C2S,C2}$**	-7323.3316	-7321.022312	-7321.249008	-7325.879325	-7323.57006	-7323.79675
^{5}**TS1$_{HA,C2R,C2}$**	-7323.3413	-7321.033597	-7321.254017	-7325.87819	-7323.57046	-7323.79088

Table S23: Relative (free) energies (in kcal mol^{-1}) of the optimized geometries for the hydroxylation reaction of taurine by the iron(IV)-oxo model ^{5,3}**Re$_{C2}$** of TauD after a geometry optimization at the UB3LYP/6-31G* level of theory.

System	BS1			BS2+Solv+Dispersion		
	ΔE	ΔE+ZPE	ΔG	ΔEdisp	ΔEdisp +ZPE	ΔEdisp + G
^{5}**Re$_{C2}$**	0.00	0.00	0.00	0.00	0.00	0.00
^{5}**TS1$_{HA,C1S,C2}$**	21.20	21.03	21.20	25.68	25.51	25.68
^{5}**TS1$_{HA,C1R,C2}$**	30.54	25.14	27.17	34.31	28.92	30.94
^{5}**TS1$_{HA,C2S,C2}$**	25.63	21.39	23.76	26.74	22.50	24.87
^{5}**TS1$_{HA,C2R,C2}$**	19.51	14.31	20.62	27.45	22.24	28.55

Data for QM/MM model D, QM region A:

Table S24: Absolute (free) energies (in au) of QM/MM optimized geometries for the hydroxylation reaction of taurine by the ^{5}**Re**$_D$ of TauD enzyme after a geometry optimization at the UB3LYP/6-31G*:Amber level of theory.

System	BS1					
	E_{tot}	E_{HL}	E_{LL}	E + ZPE	H	G
^{5}**Re**$_D$	-3164.20125	-3125.19306	-39.008186	-3125.39243	-3125.39148	-3129.92146
^{5}**TS1**$_{HA,C1S,D}$	-3164.17659	-3125.16401	-39.012575	-3125.37784	-3125.37690	-3129.90716
^{5}**TS1**$_{HA,C1R,D}$	-3164.17959	-3125.17489	-39.004699	-3125.38088	-3125.37994	-3129.91256
^{5}**TS1**$_{HA,C2S,D}$	-3164.15248	-3125.13494	-39.017536	-3125.35290	-3125.35196	-3129.87913
^{5}**TS1**$_{HA,C2R,D}$	-3164.15371	-3125.13904	-39.014675	-3125.35323	-3125.35229	-3129.88041
^{5}**IM1**$_{HA,C1S,D}$	-3164.21901	-3125.20496	-39.014048	-3125.41698	-3125.41604	-3129.95007
^{5}**IM1**$_{HA,C1R,D}$	-3164.22561	-3125.21724	-39.008369	-3125.42142	-3125.42047	-3129.95607
^{5}**IM1**$_{HA,C2S,D}$	-3164.18200	-3125.19429	-38.987703	-3125.37709	-3125.37615	-3129.90705
^{5}**IM1**$_{HA,C2R,D}$	-3164.18782	-3125.19978	-38.988037	-3125.38305	-3125.38211	-3129.91548
^{5}**TS2**$_{reb,C1S,D}$	-3164.19053	-3125.16142	-39.029112	-3125.38699	-3125.38605	-3129.91865
^{5}**TS2**$_{reb,C1R,D}$	-3164.22408	-3125.18346	-39.040622	-3125.41747	-3125.41653	-3129.94132
^{5}**TS2**$_{reb,C2S,D}$	-3164.15441	-3125.15853	-38.995876	-3125.35098	-3125.35004	-3129.88121
^{5}**TS2**$_{reb,C2R,D}$	-3164.16121	-3125.15745	-39.003759	-3125.35679	-3125.35585	-3129.89035
^{5}**Prod**$_{hydro,C1S,D}$	-3164.26563	-3125.20663	-39.058992	-3125.45594	-3125.45500	-3129.98762
^{5}**Prod**$_{hydro,C1R,D}$	-3164.24380	-3125.21099	-39.032810	-3125.43670	-3125.43576	-3129.96558
^{5}**Prod**$_{hydro,C2S,D}$	-3164.28783	-3125.24158	-39.046247	-3125.47886	-3125.47792	-3130.01383
^{5}**Prod**$_{hydro,C2R,D}$	-3164.27632	-3125.23713	-39.039193	-3125.46708	-3125.46614	-3130.00077

Table S25: Relative (free) energies (in kcal mol^{-1}) of QM/MM optimized geometries for the hydroxylation reaction of taurine by the ^{5}**Re**$_D$ of TauD enzyme after a geometry optimization at the UB3LYP/6-31G*:Amber level of theory.

System	BS1					
	ΔE_{tot}	ΔE_{HL}	ΔE_{LL}	ΔE + ZPE	ΔH	ΔG
^{5}**Re**$_D$	0.00	0.00	0.00	0.00	0.00	0.00
^{5}**TS1**$_{HA,C1S,D}$	15.47	18.23	-2.75	9.15	9.15	8.97
^{5}**TS1**$_{HA,C1R,D}$	13.59	11.40	2.19	7.24	7.24	5.58
^{5}**TS1**$_{HA,C2S,D}$	30.60	36.47	-5.87	24.80	24.80	26.56
^{5}**TS1**$_{HA,C2R,D}$	29.83	33.90	-4.07	24.59	24.59	25.76
^{5}**IM1**$_{HA,C1S,D}$	-11.14	-7.46	-3.68	-15.41	-15.41	-17.96
^{5}**IM1**$_{HA,C1R,D}$	-15.29	-15.17	-0.12	-18.19	-18.19	-21.72
^{5}**IM1**$_{HA,C2S,D}$	12.08	-0.77	12.85	9.62	9.62	9.04
^{5}**IM1**$_{HA,C2R,D}$	8.43	-4.22	12.64	5.88	5.88	3.75
^{5}**TS2**$_{reb,C1S,D}$	6.72	19.86	-13.13	3.41	3.41	1.76
^{5}**TS2**$_{reb,C1R,D}$	-14.33	6.03	-20.35	-15.72	-15.72	-12.46
^{5}**TS2**$_{reb,C2S,D}$	29.39	21.67	7.72	26.01	26.01	25.26
^{5}**TS2**$_{reb,C2R,D}$	25.12	22.35	2.78	22.36	22.36	19.52
^{5}**Prod**$_{hydro,C1S,D}$	-40.40	-8.52	-31.88	-39.86	-39.86	-41.52
^{5}**Prod**$_{hydro,C1R,D}$	-26.70	-11.25	-15.45	-27.78	-27.78	-27.69
^{5}**Prod**$_{hydro,C2S,D}$	-54.33	-30.45	-23.88	-54.24	-54.24	-57.97
^{5}**Prod**$_{hydro,C2R,D}$	-47.11	-27.65	-19.46	-46.85	-46.85	-49.77

Table S26: Group spin densities of optimized geometries for the hydroxylation reaction of taurine by the iron(IV)-oxo model ^{5}**Re$_D$** of TauD after a geometry optimization at the UB3LYP/6-31G*:Amber level of theory.

System	Fe	O	Substrate	Succinate	Amino Acids	Total
^{5}**Re$_D$**	2.975	0.684	0.000	0.055	0.286	4.00
^{5}**TS1$_{HA,C1S,D}$**	4.144	-0.046	-0.575	0.176	0.301	4.00
^{5}**TS1$_{HA,C1R,D}$**	4.208	-0.078	-0.597	0.148	0.318	4.00
^{5}**TS1$_{HA,C2S,D}$**	3.228	0.450	0.156	0.030	0.136	4.00
^{5}**TS1$_{HA,C2R,D}$**	3.582	0.247	-0.102	0.066	0.207	4.00
^{5}**IM1$_{HA,C1S,D}$**	4.253	0.195	-0.980	0.201	0.330	4.00
^{5}**IM1$_{HA,C1R,D}$**	4.264	0.218	-0.981	0.165	0.334	4.00
^{5}**IM1$_{HA,C2S,D}$**	2.799	0.160	1.011	-0.019	0.050	4.00
^{5}**IM1$_{HA,C2R,D}$**	2.815	0.160	1.002	-0.015	0.038	4.00
^{5}**TS2$_{reb,C1S,D}$**	4.160	0.146	-0.759	0.172	0.282	4.00
^{5}**TS2$_{reb,C1R,D}$**	3.749	0.045	0.015	0.089	0.101	4.00
^{5}**TS2$_{reb,C2S,D}$**	3.167	0.023	0.803	-0.003	0.010	4.00
^{5}**TS2$_{reb,C2R,D}$**	3.071	0.036	0.836	0.020	0.038	4.00
^{5}**Prod$_{hydro,C1S,D}$**	3.779	0.011	-0.011	0.092	0.129	4.00
^{5}**Prod$_{hydro,C1R,D}$**	3.794	0.003	0.010	0.058	0.135	4.00
^{5}**Prod$_{hydro,C2S,D}$**	3.801	0.000	0.005	0.084	0.110	4.00
^{5}**Prod$_{hydro,C2R,D}$**	3.814	0.006	0.002	0.061	0.118	4.00

Table S27: Group charges of optimized geometries for the hydroxylation reaction of taurine by the iron(IV)-oxo model ^{5}**Re$_D$** of TauD after a geometry optimization at the UB3LYP/6-31G*:Amber level of theory.

System	Fe	O	Substrate	Succinate	Amino Acids	Total
^{5}**Re$_D$**	1.150	-0.519	0.565	-1.170	-0.028	0.00
^{5}**TS1$_{HA,C1S,D}$**	1.324	-0.824	0.909	-1.178	-0.232	0.00
^{5}**TS1$_{HA,C1R,D}$**	1.330	-0.802	0.935	-1.177	-0.286	0.00
^{5}**TS1$_{HA,C2S,D}$**	1.230	-0.619	0.658	-1.146	-0.122	0.00
^{5}**TS1$_{HA,C2R,D}$**	1.275	-0.631	0.664	-1.157	-0.151	0.00
^{5}**IM1$_{HA,C1S,D}$**	1.315	-0.950	1.040	-1.180	-0.226	0.00
^{5}**IM1$_{HA,C1R,D}$**	1.318	-0.943	1.076	-1.193	-0.258	0.00
^{5}**IM1$_{HA,C2S,D}$**	1.171	-0.777	1.067	-1.180	-0.281	0.00
^{5}**IM1$_{HA,C2R,D}$**	1.164	-0.781	1.065	-1.182	-0.266	0.00
^{5}**TS2$_{reb,C1S,D}$**	1.232	-0.864	1.079	-1.187	-0.260	0.00
^{5}**TS2$_{reb,C1R,D}$**	0.986	-0.730	1.427	-1.220	-0.463	0.00
^{5}**TS2$_{reb,C2S,D}$**	1.157	-0.833	1.128	-1.185	-0.267	0.00
^{5}**TS2$_{reb,C2R,D}$**	1.171	-0.828	1.061	-1.171	-0.233	0.00
^{5}**Prod$_{hydro,C1S,D}$**	0.999	-0.712	1.275	-1.217	-0.345	0.00
^{5}**Prod$_{hydro,C1R,D}$**	1.004	-0.617	1.214	-1.202	-0.398	0.00
^{5}**Prod$_{hydro,C2S,D}$**	1.046	-0.470	1.030	-1.223	-0.383	0.00
^{5}**Prod$_{hydro,C2R,D}$**	1.000	-0.639	1.292	-1.240	-0.413	0.00

Table S28: Absolute (free) energies (in au) of QM/MM optimized geometries for the hydroxylation reaction of taurine by the ^{5}**Re**$_D$ of TauD enzyme after a geometry optimization at the UB3LYP/BS2:Amber level of theory.

System	BS2					
	E_{tot}	E_{HL}	E_{LL}	E + ZPE	H	G
^{5}**Re**$_D$	-3164.72344	-3125.71525	-39.008186	-3125.91461	-3125.91367	-3130.44365
^{5}**TS1**$_{HA,C1S,D}$	-3164.69978	-3125.68720	-39.012575	-3125.90103	-3125.90009	-3130.43035
^{5}**TS1**$_{HA,C1R,D}$	-3164.70005	-3125.69535	-39.004699	-3125.90134	-3125.90040	-3130.43302
^{5}**TS1**$_{HA,C2S,D}$	-3164.67497	-3125.65743	-39.017536	-3125.87539	-3125.87445	-3130.40162
^{5}**TS1**$_{HA,C2R,D}$	-3164.67721	-3125.66254	-39.014675	-3125.87673	-3125.87579	-3130.40390
^{5}**IM1**$_{HA,C1S,D}$	-3164.74342	-3125.72937	-39.014051	-3125.94139	-3125.94045	-3130.47449
^{5}**IM1**$_{HA,C1R,D}$	-3164.74831	-3125.73994	-39.008368	-3125.94412	-3125.94317	-3130.47876
^{5}**IM1**$_{HA,C2S,D}$	-3164.70903	-3125.72132	-38.987703	-3125.90412	-3125.90318	-3130.43408
^{5}**IM1**$_{HA,C2R,D}$	-3164.71392	-3125.72589	-38.988037	-3125.90915	-3125.90821	-3130.44158
^{5}**TS2**$_{reb,C1S,D}$	-3164.70345	-3125.67434	-39.029112	-3125.89991	-3125.89897	-3130.43157
^{5}**TS2**$_{reb,C1R,D}$	-3164.72285	-3125.70223	-39.020622	-3125.91625	-3125.91530	-3130.44010
^{5}**TS2**$_{reb,C2S,D}$	-3164.68312	-3125.68724	-38.995876	-3125.87969	-3125.87875	-3130.40992
^{5}**TS2**$_{reb,C2R,D}$	-3164.68905	-3125.68529	-39.003759	-3125.88463	-3125.88369	-3130.41819
^{5}**Prod**$_{hydro,C1S,D}$	-3164.80412	-3125.74513	-39.058993	-3125.99444	-3125.99350	-3130.52612
^{5}**Prod**$_{hydro,C1R,D}$	-3164.78133	-3125.74969	-39.031647	-3125.97424	-3125.97329	-3130.50312
^{5}**Prod**$_{hydro,C2S,D}$	-3164.82708	-3125.78083	-39.046246	-3126.01811	-3126.01717	-3130.55308
^{5}**Prod**$_{hydro,C2R,D}$	-3164.81565	-3125.77645	-39.039191	-3126.00641	-3126.00547	-3130.54010

Table S29: Relative (free) energies (in kcal mol^{-1}) of QM/MM optimized geometries for the hydroxylation reaction of taurine by the ^{5}**Re**$_D$ of TauD enzyme after a geometry optimization at the UB3LYP/BS2:Amber level of theory.

System	BS2					
	ΔE_{tot}	ΔE_{HL}	ΔE_{LL}	ΔE + ZPE	ΔH	ΔG
^{5}**Re**$_D$	0.00	0.00	0.00	0.00	0.00	0.00
^{5}**TS1**$_{HA,C1S,D}$	14.84	17.60	-2.75	8.52	8.52	8.34
^{5}**TS1**$_{HA,C1R,D}$	14.68	12.49	2.19	8.33	8.33	6.67
^{5}**TS1**$_{HA,C2S,D}$	30.41	36.28	-5.87	24.61	24.61	26.37
^{5}**TS1**$_{HA,C2R,D}$	29.01	33.08	-4.07	23.77	23.77	24.94
^{5}**IM1**$_{HA,C1S,D}$	-12.54	-8.86	-3.68	-16.80	-16.80	-19.35
^{5}**IM1**$_{HA,C1R,D}$	-15.61	-15.49	-0.11	-18.51	-18.51	-22.04
^{5}**IM1**$_{HA,C2S,D}$	9.04	-3.81	12.85	6.58	6.58	6.01
^{5}**IM1**$_{HA,C2R,D}$	5.97	-6.68	12.64	3.43	3.43	1.29
^{5}**TS2**$_{reb,C1S,D}$	12.54	25.67	-13.13	9.22	9.22	7.58
^{5}**TS2**$_{reb,C1R,D}$	0.36	8.17	-7.80	-1.02	-1.02	2.23
^{5}**TS2**$_{reb,C2S,D}$	25.30	17.58	7.72	21.91	21.91	21.16
^{5}**TS2**$_{reb,C2R,D}$	21.58	18.80	2.78	18.81	18.81	15.97
^{5}**Prod**$_{hydro,C1S,D}$	-50.63	-18.75	-31.88	-50.09	-50.09	-51.75
^{5}**Prod**$_{hydro,C1R,D}$	-36.33	-21.61	-14.72	-37.41	-37.41	-37.32
^{5}**Prod**$_{hydro,C2S,D}$	-65.04	-41.15	-23.88	-64.94	-64.94	-68.67
^{5}**Prod**$_{hydro,C2R,D}$	-57.86	-38.41	-19.46	-57.60	-57.60	-60.52

Test calculations with an alternative DFT Method:

Table S30: Absolute (free) energies (in au) of QM/MM optimized geometries for the hydroxylation reaction of taurine by the ^{5}**Re$_D$** of TauD enzyme after a geometry optimization at the M06/BS1:Amber level of theory.

System	BS1			
	E_{tot}	E + ZPE	H	G
^{5}**Re$_D$**	-3124.23875	-3085.42993	-3085.42898	-3089.95896
^{5}**TS1$_{HA,C1S,D}$**	-3124.21452	-3085.41577	-3085.41483	-3089.93957
^{5}**TS1$_{HA,C1R,D}$**	-3124.23255	-3085.43384	-3085.43289	-3089.96020
^{5}**TS1$_{HA,C2S,D}$**	-3124.18184	-3085.38227	-3085.38132	-3089.90996
^{5}**TS1$_{HA,C2R,D}$**	-3124.18563	-3085.38515	-3085.38421	-3089.90288

Table S31: Relative (free) energies (in kcal mol^{-1}) of QM/MM optimized geometries for the hydroxylation reaction of taurine by the ^{5}**Re$_D$** of TauD enzyme after a geometry optimization at the M06/BS1:Amber level of theory.

System	BS1			
	ΔE_{tot}	ΔE + ZPE	ΔH	ΔG
^{5}**Re$_D$**	0.00	0.00	0.00	0.00
^{5}**TS1$_{HA,C1S,D}$**	15.20	8.88	8.88	12.17
^{5}**TS1$_{HA,C1R,D}$**	3.89	-2.45	-2.45	-0.78
^{5}**TS1$_{HA,C2S,D}$**	35.71	29.91	29.91	30.75
^{5}**TS1$_{HA,C2R,D}$**	33.33	28.09	28.09	35.19

QM/MM Test calculations with Larger QM region:

Table S32: Absolute (free) energies (in au) of QM/MM optimized geometries for the hydroxylation reaction of taurine by the $^{5}\mathbf{Re}_{\mathbf{DB}}$ of TauD enzyme after a geometry optimization at the UB3LYP/BS1:Amber level of theory.

System	BS1			
	E_{tot}	E + ZPE	H	G
$^{5}\mathbf{Re}_{\mathbf{DB}}$	-8311.74999	-8272.94117	-8272.94022	-8277.47020
$^{5}\mathbf{TS1}_{\mathbf{HA,C1S,DB}}$	-8311.70491	-8272.90616	-8272.90521	-8277.43548
$^{5}\mathbf{TS1}_{\mathbf{HA,C1R,DB}}$	-8311.70125	-8272.90254	-8272.90160	-8277.43422
$^{5}\mathbf{TS1}_{\mathbf{HA,C2S,DB}}$	-8311.72241	-8272.92284	-8272.92189	-8277.44906
$^{5}\mathbf{TS1}_{\mathbf{HA,C2R,DB}}$	-8311.72854	-8272.92806	-8272.92711	-8277.45523

Table S33: Relative (free) energies (in kcal mol^{-1}) of QM/MM optimized geometries for the hydroxylation reaction of taurine by the $^{5}\mathbf{Re}_{\mathbf{DB}}$ of TauD enzyme after a geometry optimization at the UB3LYP/BS1:Amber level of theory.

System	BS1			
	ΔE_{tot}	ΔE + ZPE	ΔH	ΔG
$^{5}\mathbf{Re}_{\mathbf{DB}}$	0.00	0.00	0.00	0.00
$^{5}\mathbf{TS1}_{\mathbf{HA,C1S,DB}}$	28.29	21.97	21.97	21.79
$^{5}\mathbf{TS1}_{\mathbf{HA,C1R,DB}}$	30.58	24.24	24.24	22.58
$^{5}\mathbf{TS1}_{\mathbf{HA,C2S,DB}}$	17.30	11.50	11.50	13.26
$^{5}\mathbf{TS1}_{\mathbf{HA,C2R,DB}}$	13.46	8.23	8.23	9.40

Cartesian Coordinates.

Model A structures:

^{5}Re$_A$

C	24.60266000	77.02466900	21.76916400
C	25.59686900	77.67619300	20.80347900
O	26.55191500	77.04617800	20.34170800
C	24.57157100	77.67210200	23.18364700
C	25.90822700	77.74221500	23.85868400
N	26.53912400	76.65864400	24.45739900
C	26.82036100	78.76627600	23.94552700
C	27.77594700	77.04885500	24.86275200
N	27.98000100	78.31893900	24.55793300
H	23.59193800	77.07089300	21.34421700
H	23.85492500	77.11197500	23.79844300
H	24.18062900	78.69302400	23.12074700
H	26.73752100	79.77061600	23.55587700
H	28.50307400	76.40219600	25.32950300
H	26.15003000	75.73333800	24.57616600
N	25.33847200	78.97698000	20.49645200
C	26.33984100	79.85987800	19.92657300
C	26.61765500	80.98940100	20.94294100
O	25.82485300	81.17550600	21.86895600
H	24.68836400	79.48225900	21.09128100
H	27.23563600	79.27446600	19.70772400
N	27.73138700	81.71624200	20.72151200
C	28.17953300	82.80509700	21.59693800
C	29.60444200	82.62186600	22.12516000
C	29.85801400	81.36454700	22.92706900
O	28.88221300	80.72104700	23.46645800
O	31.02206200	80.92419000	23.09917700
H	28.32017300	81.46269700	19.93833200
H	27.47203100	82.87551100	22.41983700
H	30.35471900	82.66372600	21.32645800
H	29.78264000	83.47635500	22.79391300
C	31.62476200	77.33420100	19.13482700
C	30.85876000	77.64889100	20.37750100
N	29.51073900	77.35506900	20.53777200
C	31.23123000	78.24083000	21.55785300
C	29.12633900	77.75944600	21.76557900
N	30.14358700	78.30237700	22.41231400
H	31.19550800	77.82549600	18.25240600
H	32.65616100	77.68106900	19.23899600
H	32.18938300	78.63284900	21.85964100
H	28.11866100	77.65263800	22.11879500
H	28.86279600	76.94120700	19.87801900
Fe	30.12078900	79.23707400	24.27305500
C	27.55486700	82.51731500	25.91716800
C	28.58799300	83.54767700	26.31564900
N	27.86046500	81.15400600	26.50967800
S	28.20178900	85.11074900	25.39521300
O	29.20150500	86.07726800	25.88667400
O	28.37962800	84.68626800	23.97062400
O	26.78948600	85.36714000	25.77131000
H	27.55571800	82.37229000	24.83771700
H	28.55208100	83.77794600	27.38553800
H	29.60135200	83.23919400	26.04077000
H	27.90049300	81.19343600	27.53173400
H	27.15841000	80.45841400	26.23835700
C	31.79384900	77.49185700	24.99051400
O	32.08881700	78.59199100	24.41883200
O	30.57755800	77.20812800	25.17989800
C	32.91228300	76.57403500	25.44397000
C	32.45080300	75.18213500	25.87629300
H	31.97273600	74.64812000	25.04812200
H	33.30335400	74.58580000	26.21838600
H	31.72592100	75.24704700	26.69312600
H	33.64626500	76.52229500	24.63121700
H	33.42183000	77.08988200	26.26912100
O	30.15129800	79.97275800	25.73177700
H	31.65308000	76.25591300	18.93316400
H	25.98171000	80.29598900	18.98455400
H	24.88533800	75.97068400	21.83480400
H	26.55895200	82.81766700	26.24715200
H	28.78968600	80.77189700	26.16267200
H	28.12783000	83.75544700	21.05495700

^{5}TS1$_{HA,C1S,A}$

C	24.95052000	76.94879000	21.22340600
C	25.72838100	78.05065000	20.49796600
O	26.71445000	77.79875900	19.79993200
C	24.87276700	77.10745200	22.76802900
C	26.20458600	77.17258000	23.45000300
N	27.03024100	76.07008100	23.63359900
C	26.92909400	78.23613000	23.92886400
C	28.19366500	76.50109200	24.18999300
N	28.16548300	77.80660700	24.37053400
H	23.92818200	76.91276200	20.82491700
H	24.27553400	76.27419600	23.16211300
H	24.32087000	78.01665000	23.02957500
H	26.65788100	79.28150300	23.95276300
H	29.02377800	75.84989600	24.42204400
H	26.79901000	75.11017800	23.42048700
N	25.22972900	79.30773700	20.65186900
C	25.97651100	80.49962400	20.29367700
C	26.17333600	81.33998200	21.57368900
O	25.42121400	81.16391400	22.53107400
H	24.58422900	79.48158200	21.41677800
H	26.92096400	80.19393900	19.83696600
N	27.17176300	82.24931000	21.52951800
C	27.60905200	83.02594700	22.69340400
C	29.10856200	82.89433500	22.96739000
C	29.57045200	81.50570900	23.37074500
O	28.70708700	80.55821800	23.45374900
O	30.77058600	81.28098900	23.66705200
H	27.76102200	82.26748700	20.70708800
H	27.03847800	82.66770700	23.55054400
H	29.71521000	83.23364900	22.11631200
H	29.35065100	83.54615200	23.81477500
C	31.49537900	76.36900800	19.58064300
C	30.81423200	77.23281800	20.59088300
N	29.55759100	77.78572000	20.39492800
C	31.20491700	77.67722200	21.83109100
C	29.24074100	78.51668200	21.48416500
N	30.21544700	78.47185500	22.37536800
H	31.69103300	76.90891800	18.64550000
H	32.45657000	76.02580500	19.97295800
H	32.11683000	77.48145000	22.37264500
H	28.30962400	79.03763700	21.61057100
H	28.88652800	77.60321400	19.65657200
Fe	29.96381700	79.21071900	24.31244700
C	31.55203500	81.23074700	27.81969900
C	30.77301700	81.90446000	26.71524700
N	30.64120700	80.21993000	28.47231700

S	29.43202700	82.95890200	27.30514000
O	28.63958800	83.33082200	26.11444100
O	28.71505200	82.04833700	28.27322800
O	30.16198900	84.06709500	27.97279700
H	31.86261800	81.95389300	28.58099300
H	31.37550800	82.36392400	25.93401700
H	30.12337900	80.85885400	26.10573200
H	30.58505100	79.40310000	27.82729100
H	30.99551400	79.90709800	29.37713400
C	31.91397400	77.63075600	25.92394800
O	31.69926600	78.10739800	27.04100700
O	31.36408500	77.98492000	24.79158600
C	32.92444900	76.49120600	25.76272700
C	32.29704900	75.21776300	25.17698100
H	31.87119200	75.41410800	24.18797800
H	33.04632100	74.42458000	25.07739100
H	31.49845500	74.83973200	25.82717000
H	33.72557400	76.84139700	25.09863800
H	33.36396000	76.29129000	26.74392300
O	29.63384300	79.81417400	25.89937700
H	30.90016300	75.48116900	19.33133100
H	25.42408700	81.09329300	19.55260200
H	25.43801900	76.00643300	20.95888800
H	32.42035000	80.68850100	27.44142400
H	29.70470800	80.71659100	28.57127500
H	27.36109100	84.08339200	22.54078600

^{5}TS1$_{HA,C1R,A}$

C	24.71162200	77.01273700	21.05122500
C	25.73193300	77.96303700	20.41659800
O	26.75897800	77.54050400	19.87858900
C	24.49689700	77.22448700	22.57703200
C	25.75957800	77.19581800	23.38251000
N	26.44623500	76.03531600	23.72538800
C	26.55827400	78.21938700	23.82622300
C	27.60535100	76.38896400	24.33782100
N	27.70501000	77.70437000	24.39885200
H	23.74446100	77.12269000	20.54378700
H	23.78791100	76.46441000	22.93088400
H	24.01354500	78.19071800	22.75733700
H	26.40295200	79.28415800	23.74613700
H	28.34795800	75.69588200	24.70187400
H	26.13262900	75.08798900	23.56865300
N	25.41356700	79.28480100	20.47778800
C	26.38415500	80.33250200	20.22301400
C	26.40455500	81.27230000	21.44683900
O	25.48293200	81.23268100	22.26060300
H	24.69435600	79.58604800	21.13004800
H	27.35876600	79.86999700	20.04732200
N	27.46039800	82.11484300	21.51252700
C	27.79416800	82.89912300	22.70703900
C	29.20047900	82.58915800	23.22389500
C	29.38454700	81.16433500	23.70360600
O	28.39813000	80.36757500	23.74907000
O	30.52490400	80.75474600	24.08417500
H	28.19595400	81.99181800	20.82810300
H	27.04751900	82.65815400	23.46422200
H	29.96789700	82.80264900	22.46641600
H	29.43247000	83.23259400	24.08123100
C	32.09328000	77.96327600	19.46434200
C	31.14039000	78.03088900	20.61244200
N	29.77980800	77.78414200	20.48884100
C	31.32055100	78.31834700	21.94390900
C	29.21108300	77.92265200	21.70818700
N	30.11493200	78.24540300	22.61447200
H	31.82710800	78.66788200	18.66578500
H	33.10140700	78.21498200	19.80486100
H	32.22684700	78.58033500	22.46861300
H	28.15587900	77.79906700	21.87046100
H	29.24886200	77.55487400	19.65802300
Fe	29.59848600	78.83168400	24.70239800
C	31.34937100	80.89589800	27.94752000
C	29.89258300	81.27358900	27.77803700
N	32.02782100	80.87471400	26.60137600
S	29.64029500	82.97249900	27.16168100
O	28.27907900	83.04092100	26.60665500
O	29.90959200	83.76327500	28.38839900
O	30.72520700	83.11487800	26.11744700
H	31.45494400	79.90662500	28.39793500
H	29.41704500	80.43332300	26.93954300
H	29.30701300	81.11056700	28.68400900
H	31.63750300	81.71143900	26.07208100
H	33.04057600	80.97315300	26.68825200
C	31.49602100	77.21569100	25.44784700
O	31.82330600	78.41941200	25.18160900
O	30.30373800	76.83291500	25.28429100
C	32.55741000	76.25317700	25.95618100
C	32.07375000	74.81787000	26.16098900
H	31.71796700	74.38548300	25.22031600
H	32.88568100	74.18825000	26.54125600
H	31.24591900	74.78226200	26.87547100
H	33.39670000	76.29396300	25.25007900
H	32.94500200	76.67195000	26.89511900
O	29.31860000	79.34983900	26.35991700
H	32.13244200	76.96017600	19.02015000
H	26.11765000	80.90669400	19.32466400
H	25.07471300	76.00087700	20.85190700
H	31.86535000	81.63577500	28.56742800
H	31.83233400	79.99804400	26.07489900
H	27.71384600	83.96839200	22.47750100

^{5}TS1$_{HA,C2S,A}$

C	24.73740700	77.25642100	20.99852000
C	25.61554400	78.34107400	20.36415800
O	26.55133300	78.05027400	19.61299400
C	24.62749700	77.29629100	22.54721000
C	25.94174400	77.20932200	23.26020200
N	26.68935900	76.04131300	23.36811400
C	26.72179100	78.18106000	23.83247800
C	27.86631800	76.34220300	23.97258600
N	27.91608400	77.63011600	24.25548900
H	23.72642200	77.33057900	20.57688800
H	23.96246200	76.48014300	22.86001100
H	24.13430000	78.21883600	22.87157400
H	26.52838400	79.23968600	23.92662900
H	28.65168600	75.62556800	24.16176500
H	26.40349500	75.12052600	23.06672600
N	25.25997600	79.62192200	20.64482300
C	26.09306400	80.76532200	20.31789400
C	26.36910300	81.54217900	21.62466600
O	25.59643300	81.41279200	22.57233200
H	24.65041400	79.80921700	21.43582100
H	27.00413600	80.40430600	19.83479400
N	27.45849500	82.34075800	21.59891700
C	28.01294600	83.03365800	22.76829900
C	29.44733200	82.59686000	23.08582500

C	29.54266700	81.18848100	23.62001800
O	28.55228700	80.39352800	23.55601500
O	30.63239800	80.73942000	24.11571800
H	28.03587300	82.31280500	20.76818500
H	27.36613100	82.82379500	23.61990200
H	30.09424300	82.66449300	22.19714200
H	29.87040900	83.27568500	23.83549500
C	31.05957400	76.05465700	19.51250400
C	30.49640800	76.98228300	20.53907500
N	29.34322700	77.72612000	20.34059800
C	30.92182700	77.32776200	21.79994700
C	29.12304500	78.46325900	21.45209600
N	30.05809100	78.24831200	22.36070700
H	31.35461100	76.58496200	18.59800100
H	31.94952100	75.55795800	19.90908500
H	31.78479100	76.97510300	22.34373200
H	28.27891500	79.11707100	21.58079900
H	28.66481800	77.66447500	19.58834900
Fe	29.69260700	78.88352100	24.54959400
C	30.04090200	80.93331700	27.72354000
C	30.74596900	82.08101400	27.07535900
N	30.94430700	79.84946700	28.22437400
S	29.46724700	83.36761500	26.70003400
O	30.18013700	84.35854700	25.85764000
O	28.42925100	82.57889200	25.97088400
O	29.05706200	83.82378100	28.04824700
H	29.38061700	80.28426000	26.82904800
H	31.47388900	82.54682200	27.75470400
H	31.23884900	81.78241000	26.14675100
H	31.65449300	80.21080300	28.87083500
H	30.39699400	79.13308500	28.70960200
C	32.09419700	77.46199400	25.72131800
O	32.33176800	78.26159200	26.64828600
O	31.13682200	77.54434500	24.86322800
C	33.01826800	76.25951200	25.52149000
C	34.04066400	76.04938000	26.63693200
H	33.54601100	75.87268600	27.59783100
H	34.67783500	75.18531000	26.41792900
H	34.67914300	76.92966300	26.75286500
H	32.38305100	75.37471300	25.38833500
H	33.52329100	76.40578700	24.55646100
O	29.23837000	79.22838200	26.23988900
H	30.34277000	75.27492500	19.22386600
H	25.57774200	81.42542100	19.60664000
H	25.15659500	76.30151700	20.66951700
H	29.31516200	81.21364700	28.48884000
H	31.44321600	79.32138200	27.42619400
H	27.99777800	84.11412400	22.58537200

^{5}TS1$_{HA,C2R,A}$

C	24.12079400	78.50405600	22.91224700
C	25.07620400	78.55415100	21.71696800
O	25.69353000	77.55623800	21.33912400
C	24.58771300	79.34183600	24.13898800
C	26.01644300	79.09340100	24.50664700
N	26.46966200	78.10843900	25.37172400
C	27.14208200	79.64461300	23.96011400
C	27.81829200	78.11936700	25.34192700
N	28.26012800	79.01615500	24.47639700
H	23.12386200	78.84668800	22.60578400
H	23.92028700	79.12879500	24.98183900
H	24.47437900	80.41077000	23.92536100
H	27.22738500	80.45024100	23.25053500
H	28.41806400	77.48931600	25.97680800
H	25.98634100	77.74658800	26.18965600
N	25.19184400	79.76244300	21.09023100
C	26.23006000	80.01935300	20.11069200
C	26.97087600	81.31449400	20.48419600
O	26.43419200	82.14699100	21.20840700
H	24.83532500	80.60059700	21.53777100
H	26.89539400	79.15277600	20.08464100
N	28.20281100	81.46679600	19.92477000
C	29.10566700	82.55460500	20.29631000
C	30.46709000	82.06225600	20.80889400
C	30.39011300	81.23378100	22.07940600
O	29.28002700	81.06320500	22.65241600
O	31.43227000	80.67211500	22.55463600
H	28.59487100	80.67878400	19.42613000
H	28.58748100	83.12666100	21.06761900
H	30.99004500	81.46394400	20.05039100
H	31.11637100	82.92634500	20.99910800
C	31.04238000	76.16997900	19.28804600
C	30.41788600	76.91208500	20.42392300
N	29.07319200	76.81186700	20.75255900
C	30.93393300	77.77934200	21.35448700
C	28.83545600	77.59051900	21.83790300
N	29.94417800	78.19489000	22.22504000
H	30.57380700	76.41429700	18.32579800
H	32.10240500	76.42839000	19.21576300
H	31.94858900	78.13266500	21.46048200
H	27.85595900	77.68238000	22.27766200
H	28.36656600	76.26083000	20.28455900
Fe	30.23253100	79.63892600	23.99788100
C	29.97963400	80.56319500	27.72912700
C	29.43666700	79.26843900	28.25420900
N	31.47426200	80.62252100	27.74755500
S	27.58350700	79.37192000	28.44779300
O	27.41604300	79.90123000	29.81565600
O	27.18273300	80.29573000	27.35304600
O	27.12879400	77.96418700	28.23827100
H	29.54050800	81.47373600	28.13667100
H	29.70291600	78.44084100	27.59114400
H	29.83858100	79.04234400	29.25168400
H	31.85528200	80.61509000	28.69987300
H	31.87517600	79.77467300	27.19124100
C	32.38526800	77.96450000	25.46170700
O	31.48482100	78.21896800	24.57896700
O	32.54822500	78.55274400	26.55240400
C	33.31414200	76.80780800	25.09934400
C	34.34899100	76.45743700	26.16652800
H	33.86528600	76.16910200	27.10496700
H	34.97634600	75.62295000	25.83391600
H	34.99904100	77.31123400	26.38090800
H	32.67608100	75.94630500	24.86400300
H	33.79741200	77.07692700	24.15059900
O	30.35715300	80.78349700	25.36844200
H	30.97443300	75.08259500	19.42152100
H	25.80129900	80.13903600	19.10471400
H	24.04331400	77.44911900	23.18659800
H	29.80631800	80.66746900	26.44203500
H	31.79181800	81.46314000	27.25659300
H	29.26627000	83.21427500	19.43376700

^{5}IM1$_{HA,C1S,A}$

C	24.74622500	76.92770100	20.96712300
C	25.77571600	77.88276600	20.35683900
O	26.83460400	77.47208100	19.87560800
C	24.49261500	77.15387200	22.48559300
C	25.73515400	77.14723200	23.32218800
N	26.41817200	75.99687200	23.70321300
C	26.51572500	78.18188600	23.77299100
C	27.55603100	76.36703400	24.34492200
N	27.64831500	77.68429400	24.38852500
H	23.79141500	77.02638900	20.43475900
H	23.78315000	76.39030300	22.83062500
H	23.99554200	78.11717200	22.64251000
H	26.35153000	79.24388200	23.67840300
H	28.29083500	75.68379100	24.74132400
H	26.11400700	75.04515800	23.55420100
N	25.42933200	79.19994800	20.37965700
C	26.39694800	80.26227900	20.18065000
C	26.35569900	81.18099600	21.41893600
O	25.39557200	81.12609300	22.18668000
H	24.66963300	79.49000600	20.98929100
H	27.38279300	79.81354400	20.03533400
N	27.40408100	82.02342500	21.56041300
C	27.62378400	82.82850400	22.76556700
C	29.01343100	82.60903900	23.36766500
C	29.27773600	81.18789700	23.82542400
O	28.30836000	80.37224300	23.95458100
O	30.44918400	80.79899800	24.09779700
H	28.17113800	81.94116300	20.90545600
H	26.85057700	82.53847800	23.47736200
H	29.81110300	82.90991600	22.67586100
H	29.10944000	83.24321700	24.25757300
C	32.12011800	78.24685500	19.54286500
C	31.15061800	78.20311300	20.67814200
N	29.82380500	77.82009400	20.53290100
C	31.28406800	78.49074800	22.01444400
C	29.22804200	77.88208400	21.74528000
N	30.08303100	78.28588700	22.66713800
H	31.79094700	78.92597000	18.74573500
H	33.09118300	78.60103200	19.89934100
H	32.15092800	78.83864100	22.55513200
H	28.18952400	77.64615200	21.88893900
H	29.32765100	77.55954400	19.68996500
Fe	29.55467500	78.83275200	24.76485400
C	32.14616000	81.21939000	26.90341300
C	31.39037100	82.45997600	26.55283500
N	31.35424400	80.33804600	27.82737700
S	29.89863400	82.96276200	27.37016200
O	28.80746800	82.18653900	26.68959800
O	30.12433800	82.49383300	28.78390400
O	29.80633400	84.41800200	27.18224100
H	33.09145200	81.45910900	27.41324000
H	31.68568600	83.01277500	25.66918900
H	28.80996400	80.29648800	26.55713100
H	30.54412400	79.86107200	27.28558200
H	31.93895300	79.62286300	28.25953200
C	31.38583700	77.17018100	25.45086800
O	31.66898000	78.38969800	25.22173600
O	30.18846900	76.77633800	25.33051000
C	32.49160700	76.20661600	25.84662000
C	32.00588100	74.82565400	26.28726900
H	31.44545300	74.33451400	25.48613100
H	32.85399200	74.18637200	26.55581700
H	31.34315700	74.89934600	27.15544900
H	33.16664400	76.12701600	24.98338900
H	33.08332200	76.69158200	26.63337400
O	29.26269400	79.41874700	26.51040500
H	32.27110500	77.25741000	19.09216700
H	26.15833600	80.84957900	19.28301000
H	25.12182900	75.91697300	20.78636000
H	32.37052900	80.62976300	26.00989800
H	30.90217300	80.96758300	28.54277200
H	27.48939600	83.89124000	22.52990000

^{5}IM1$_{HA,C1R,A}$

C	24.70956100	77.04483100	20.99156900
C	25.74516700	77.98655000	20.37005700
O	26.77701100	77.55782700	19.84697300
C	24.49494200	77.25003900	22.51861600
C	25.75557800	77.19771900	23.32616900
N	26.41790400	76.02427800	23.67244900
C	26.57299700	78.20511600	23.77280500
C	27.58063900	76.35462400	24.29015900
N	27.70722300	77.66834500	24.35164900
H	23.74534900	77.17255700	20.48282800
H	23.77467800	76.49799500	22.86656100
H	24.02543800	78.22211500	22.70385200
H	26.43505300	79.27246400	23.69541600
H	28.30767000	75.64708200	24.65760800
H	26.08623600	75.08312000	23.51521300
N	25.43463900	79.31113200	20.42840900
C	26.42076800	80.35165100	20.20563000
C	26.45129300	81.25632700	21.45525100
O	25.52386500	81.21141300	22.26270900
H	24.70719400	79.61118000	21.07176200
H	27.38847200	79.87955200	20.01815200
N	27.52301800	82.07418800	21.55592100
C	27.84107400	82.84393400	22.76378000
C	29.24756800	82.54086000	23.28258600
C	29.44940100	81.11212800	23.73640800
O	28.46000000	80.31759200	23.83317200
O	30.60348300	80.68947700	24.04677100
H	28.25912700	81.97046000	20.86909100
H	27.09100900	82.58171500	23.51068800
H	30.01678700	82.77861600	22.53489700
H	29.47214800	83.16709500	24.15694900
C	32.11266900	77.99294000	19.42312200
C	31.15920400	78.02828800	20.57209300
N	29.80306100	77.75851100	20.44517800
C	31.33491500	78.30120900	21.90678400
C	29.23121400	77.86905100	21.66532600
N	30.13012600	78.19718500	22.57532100
H	31.83135900	78.70066900	18.63265000
H	33.11549200	78.26153000	19.76614700
H	32.23578300	78.57347800	22.43539600
H	28.17837800	77.72289600	21.82449400
H	29.27638600	77.53576900	19.60995000
Fe	29.64878500	78.74606900	24.67573400
C	31.75495900	81.40480800	28.08622800
C	30.49673700	82.10851500	28.48037800
N	31.61979800	80.81983300	26.71410900
S	29.52447500	82.99585500	27.28892500
O	28.48689300	82.00925100	26.84429100
O	28.99288000	84.17928200	27.98095600
O	30.52478000	83.27699600	26.19824600
H	31.98342800	80.59198300	28.78226000

H	28.82472800	80.14757100	26.52810800
H	30.13245000	82.06313300	29.50045000
H	31.40698300	81.59324800	26.05083800
H	32.43147800	80.28966300	26.38932400
C	31.45398400	77.08041200	25.40833700
O	31.76716900	78.28896200	25.15566200
O	30.25841100	76.69970200	25.23629900
C	32.51444100	76.12310000	25.92168100
C	32.05300000	74.67144400	26.04990100
H	31.74790800	74.26816700	25.07876800
H	32.86090900	74.04385400	26.44169400
H	31.19605600	74.59255200	26.72535600
H	33.38217900	76.21056000	25.25573300
H	32.84939600	76.51195800	26.89317800
O	29.41983600	79.36793400	26.41926900
H	32.17195100	76.99574700	18.96818200
H	26.16382600	80.95501500	19.32392000
H	25.05883200	76.02934700	20.78624300
H	32.61603100	82.08802200	28.06284300
H	30.77313000	80.15615800	26.65314300
H	27.75025300	83.91587200	22.55107300

^{5}IM1$_{HA,C2S,A}$

C	24.66520700	76.93080800	20.49825500
C	25.80029100	77.81637900	19.98027700
O	26.89863500	77.35102500	19.66956600
C	24.23606600	77.24866300	21.95990200
C	25.36523200	77.23515200	22.94394800
N	25.93866300	76.07664000	23.45611800
C	26.13295100	78.25598900	23.44672200
C	27.00479300	76.42611600	24.21996100
N	27.15275300	77.73856400	24.22091900
H	23.78914200	77.03286200	19.84491300
H	23.45606300	76.53265900	22.25005700
H	23.76696400	78.23728000	22.00699700
H	26.03460000	79.31889300	23.28915200
H	27.65118100	75.73261000	24.73578500
H	25.60819300	75.13298800	23.31157200
N	25.50337600	79.14399900	19.88549300
C	26.53266200	80.15883400	19.77108500
C	26.38412600	81.13115000	20.95668400
O	25.33044400	81.17102800	21.59075200
H	24.67535900	79.48925800	20.36160300
H	27.50755500	79.66491500	19.76383700
N	27.45329800	81.92264800	21.20565500
C	27.53458900	82.78954000	22.38119200
C	28.84658700	82.61021200	23.14725700
C	29.05803200	81.23710100	23.78283300
O	28.11505000	80.39055400	23.75373700
O	30.17422500	80.97887000	24.31373200
H	28.30370600	81.75373600	20.68424700
H	26.68036300	82.53606300	23.01054900
H	29.71510700	82.82187000	22.50887000
H	28.89108100	83.35442700	23.95296100
C	32.27502500	77.72364000	20.03332300
C	31.15560400	77.87092500	21.01108100
N	29.82523200	77.64393600	20.68652900
C	31.13266200	78.22168900	22.33933700
C	29.07671000	77.85172500	21.79440600
N	29.83558500	78.20480000	22.81537900
H	32.15876000	78.39023100	19.16890600
H	33.22375500	77.97092200	20.51734500
H	31.95314300	78.48133200	22.99055800
H	28.00767500	77.74116800	21.79675100
H	29.42961700	77.35862900	19.79964200
Fe	29.01887300	78.78303500	24.83430400
C	31.11676900	81.81623100	27.84376400
C	29.96908200	81.56003700	28.71966600
N	31.35582100	80.79324400	26.82083400
S	30.25920200	79.95567900	29.68047500
O	29.07049400	79.76982400	30.52498000
O	30.34137800	78.96181000	28.51689700
O	31.56427100	80.13918200	30.34332300
H	28.97712700	79.07361300	27.27020800
H	29.83719300	82.35205400	29.45862300
H	29.03769500	81.38651500	28.17088600
H	30.77337700	80.90736800	25.95787000
H	32.32935000	80.75399400	26.51610700
C	30.73506000	77.13182200	25.75355700
O	31.08225100	78.32524400	25.46742300
O	29.54708200	76.76400700	25.49797900
C	31.71840600	76.15423000	26.36281300
C	32.85153700	76.81521000	27.15153800
H	32.44862700	77.40003400	27.98380400
H	33.52842900	76.05508400	27.55634700
H	33.43455700	77.48407400	26.50988100
H	31.14448500	75.46518800	26.99103500
H	32.12145600	75.55219500	25.53458600
O	28.55793900	79.44234800	26.45599300
H	32.35456600	76.69781700	19.65092100
H	26.43201600	80.71874000	18.83082900
H	25.02008800	75.89998600	20.41812300
H	32.01448500	82.29354300	28.21757700
H	31.09855700	79.84910900	27.23895000
H	27.43049900	83.83845200	22.07438200

^{5}IM1$_{HA,C2R,A}$

C	24.51116000	78.36207200	23.21743300
C	25.30398700	78.39993100	21.90907400
O	25.91265700	77.41345100	21.48851700
C	25.04220900	79.32955400	24.31425900
C	26.51429300	79.19808600	24.54949700
N	27.09508800	78.24837800	25.37449100
C	27.55558100	79.83984700	23.93148200
C	28.43371500	78.36067200	25.25394700
N	28.75123700	79.30240500	24.37466300
H	23.45752200	78.59634700	23.01697500
H	24.48489500	79.14627600	25.24006400
H	24.83273200	80.36800900	24.03376900
H	27.53359600	80.64045800	23.20926500
H	29.14072800	77.77355200	25.82057200
H	26.69513100	77.87003200	26.24518900
N	25.28574900	79.58833800	21.23693500
C	26.18983000	79.86627000	20.13865000
C	26.92893800	81.18616300	20.41717300
O	26.44885200	82.01238900	21.18822600
H	24.94354700	80.42230400	21.70249100
H	26.87309400	79.01984700	20.03632300
N	28.08915400	81.36362000	19.73103000
C	28.96173800	82.51861900	19.92884600
C	30.40202200	82.12532800	20.28146800
C	30.53557300	81.38199700	21.60022000
O	29.56740500	81.36275700	22.41664400
O	31.60704400	80.75511500	21.87666700
H	28.43311800	80.59763000	19.16692700
H	28.51309400	83.10123300	20.73449800

H	30.85683700	81.50677100	19.49732700
H	31.01782500	83.03301200	20.33606400
C	30.68724100	76.25961400	18.92481500
C	30.27377800	77.02425900	20.13952700
N	29.02266500	76.90206000	20.72793800
C	30.93609000	77.93465200	20.92556700
C	28.97920700	77.70896900	21.81452000
N	30.12223500	78.35513100	21.96258500
H	30.03182300	76.45880300	18.06694400
H	31.70435300	76.54084200	18.63850000
H	31.93830700	78.32084800	20.81725100
H	28.10163100	77.79150800	22.43077900
H	28.23705000	76.34737600	20.41399500
Fe	30.67101200	79.86764300	23.61185900
C	30.03740200	80.08143700	28.84997100
C	29.18070300	78.94738400	29.20998300
N	30.16088700	80.29558600	27.41100100
S	27.36519400	79.22695900	28.66292400
O	26.65332700	79.71088000	29.85442700
O	27.50269500	80.24380400	27.55722500
O	26.93098100	77.89279300	28.13849600
H	30.11435300	80.97414300	29.45948800
H	29.48294800	78.02748900	28.69911700
H	29.14286900	78.77937200	30.28691200
H	30.71040600	81.10198100	27.09854900
H	30.59956400	79.46227300	26.93399600
C	32.12213400	77.93334400	25.40196800
O	31.88802000	78.51103800	24.26934600
O	31.43370000	78.07604900	26.43076800
C	33.33223400	77.00688000	25.40175600
C	33.61800600	76.33917500	26.74547000
H	32.76623500	75.73682600	27.07554200
H	34.49381400	75.68535800	26.66974300
H	33.81299700	77.08456100	27.52268500
H	33.16628200	76.26076700	24.61336500
H	34.18915600	77.60217900	25.06021100
O	31.03474000	81.18850200	24.88510900
H	30.67943400	75.17634000	19.10075600
H	25.63973900	79.96758000	19.19151300
H	24.56183300	77.32927900	23.57055200
H	30.61734700	82.01539100	24.58859200
H	29.16935800	80.40089700	27.05937200
H	28.96825400	83.13755900	19.02217100

Model B structures:

[1]Re$_B$

C	29.64428900	89.00739100	27.72603600
C	28.35258100	88.25312800	27.63380100
N	27.14345800	88.85618500	27.91798500
C	28.14974400	86.93491600	27.28741400
C	26.23685400	87.91233500	27.74726800
N	26.78932900	86.72814100	27.36497000
H	29.80983600	89.39013800	28.74075400
H	30.49518900	88.36832300	27.46408400
H	28.82503100	86.13591400	27.02236900
H	25.17125200	88.02938900	27.89604400
H	26.30268300	85.85597200	27.14705600
C	28.85877600	83.04879400	34.86970000
C	28.30706600	82.54551800	33.55482800
C	28.01558500	81.19001300	33.35817800
C	28.04810200	83.42681200	32.49474800
C	27.48222600	80.71601600	32.15744100
C	27.50748600	82.97839600	31.29098600
C	27.21831700	81.61916300	31.12043000
O	26.71151300	81.21259400	29.90871800
H	28.07009500	83.48097800	35.50092100
H	29.61206000	83.83095500	34.71862500
H	28.21537300	80.48078400	34.15934400
H	28.26631600	84.48625500	32.61305400
H	27.28816700	79.65731200	32.00628200
H	27.29964000	83.66989700	30.47951100
H	26.23268500	80.32150600	29.97515800
C	32.77739000	82.62439300	29.14998600
C	31.58516000	82.66523500	28.17828900
C	33.61185500	83.91049600	29.18266900
C	30.49712200	83.68066400	28.54921200
H	32.40801000	82.40535700	30.16218000
H	31.95291800	82.88057800	27.16326300
H	31.12959300	81.66625000	28.13181800
H	29.66383400	83.64093300	27.83906900
H	30.88019600	84.70788500	28.55123000
H	30.09122000	83.47759100	29.54811900
H	34.47186400	83.80691000	29.85451200
H	33.99701500	84.15592000	28.18426300
H	33.02463400	84.76734700	29.53055100
C	26.03683600	75.31612900	29.46675800
C	27.15968800	75.26882600	28.43813600
O	27.03584100	74.71313200	27.33177300
C	25.21811400	76.61829800	29.37382100
C	25.93980700	77.87271900	29.89319400
O	26.99320700	77.75763300	30.55153900
O	25.36077100	78.99268400	29.61932100
H	25.38563300	74.45617700	29.28553000
H	24.89896500	76.79043700	28.33789200
H	24.29656700	76.51859700	29.96215300
N	28.29748100	75.90400900	28.80108000
C	29.35018300	76.19383300	27.83356300
C	29.91951800	77.60689100	28.01195300
C	28.83097800	78.67382200	27.99246200
O	27.88179300	78.58421000	27.19642000
N	28.98034300	79.70445500	28.84311700
H	28.92197300	76.07622000	26.83596600
H	30.50666700	77.67574100	28.93594900
H	30.59902000	77.81401900	27.17465000
H	29.63616500	79.62311700	29.60713400
H	28.19098900	80.33390900	29.01442900
H	28.20275900	76.49003600	29.63386300
C	22.40434000	74.71407400	25.27315600
C	23.89235800	74.88860700	25.47631800
O	24.50655300	75.82189200	24.90599800
N	24.51736900	74.03127300	26.29089100
H	21.87796600	75.51341400	25.81321700
H	22.03695300	73.74721500	25.62899600
H	23.98539900	73.31728000	26.76727700
H	25.47975800	74.22288700	26.62144900
C	26.63046200	75.44502900	20.19957400
C	26.65456000	76.73127000	19.36883800
O	27.59640100	76.99187600	18.62687800
C	26.24300400	75.59424500	21.68214300
C	27.10427400	76.54757800	22.45726800
N	26.98024500	76.67947200	23.82985800
C	28.07633400	77.44534300	22.09230400
C	27.84115200	77.62647100	24.25249500
N	28.52418700	78.10506300	23.21972900
H	25.93126500	74.74305100	19.72706300
H	26.29314800	74.59964100	22.14543200
H	25.18968400	75.89612600	21.77938000
H	28.46821500	77.67150600	21.11492100
H	27.95131900	77.94173700	25.28119700
H	26.27228700	76.21623300	24.40955100
N	25.54361700	77.53145800	19.47555100
C	25.61080500	78.91755300	19.04554300
C	25.76615400	79.82191200	20.28053600
O	25.29534500	79.46820700	21.36367300
H	24.98058700	77.41658800	20.31108300
H	26.45107900	79.01292400	18.35571400
N	26.45133600	80.96934700	20.06982500
C	26.55595400	82.02652000	21.06611700
C	25.60687700	83.17056900	20.64231100
O	25.36083600	83.34965800	19.44626800
C	27.98481000	82.58818400	21.12506700
C	29.12360100	81.64723400	21.53994200
O	28.76324500	80.54324100	22.11172100
O	30.28559200	82.02307800	21.32311800
H	26.53472300	81.29353200	19.11210600
H	26.27832400	81.60161700	22.03080300
H	28.25533500	83.02929900	20.15844700
H	27.99679200	83.41151300	21.85099100
N	25.13314500	83.94599600	21.64137300
C	24.27237700	85.09530800	21.37442400
C	25.02882600	86.37611100	20.96760500
C	24.02108800	87.47290500	20.59446300
C	25.98508200	86.85339600	22.06893000
H	23.68016000	85.28029500	22.27715100
H	25.61625500	86.12667800	20.07354400
H	24.53592300	88.38298700	20.26411000
H	23.36099400	87.14849600	19.78070400
H	23.39188100	87.74001000	21.45345500
H	26.71485700	86.07757600	22.32864800
H	26.53943700	87.74345300	21.74586400
H	25.43279600	87.11666600	22.97988600
H	25.38862000	83.73084100	22.60381600
C	19.42576000	86.27783600	23.40025300
C	20.73936200	86.67079000	24.03783300
C	21.10888800	88.01965200	24.14284000
C	21.62786100	85.70011200	24.52042400
C	22.33167800	88.38749100	24.70671900
C	22.85464800	86.05984400	25.08807700
C	23.20883700	87.40856900	25.18074000

H	19.15089000	85.24817300	23.65140900
H	18.60968400	86.93469900	23.72344300
H	20.42976800	88.78894100	23.78025800
H	21.35842400	84.64863200	24.44325800
H	22.59787300	89.43896400	24.78070000
H	23.53542800	85.29603900	25.45539700
H	24.16178000	87.69201200	25.61922200
C	20.84690300	77.61379400	29.02208400
C	21.57484600	77.92684700	30.32878500
O	22.18975400	77.06770300	30.95824700
C	21.70965500	78.08634500	27.84808400
O	21.19253900	77.58185000	26.62717400
H	20.71369700	76.53082700	28.96452800
H	21.73941400	79.18754200	27.82767100
H	22.74101300	77.73939800	28.00869900
H	21.81280300	77.89928900	25.93564000
N	21.55050300	79.23820100	30.69966900
C	22.49447300	79.77626900	31.67677200
C	22.13944000	81.22875300	32.03762700
C	22.05360300	82.15522500	30.83719300
C	20.81317700	82.63702800	30.39438800
C	23.20653400	82.53674600	30.13029000
C	20.72124300	83.48006500	29.28226600
C	23.12025400	83.38253500	29.02268900
C	21.87474300	83.85683700	28.59412300
H	23.51309700	79.71487300	31.27208900
H	21.18417100	81.24246200	32.57654200
H	22.90527100	81.59017800	32.73504800
H	19.91084400	82.36256100	30.93780800
H	24.18154900	82.17495300	30.44781300
H	19.74931200	83.84917100	28.96421500
H	24.02142300	83.68251700	28.49427100
H	21.81274200	84.52633000	27.73988200
H	21.14776400	79.90731300	30.05495800
C	19.06089700	77.54857000	23.93088400
C	18.88723800	79.00363500	23.45916400
C	20.15090200	79.83087700	23.58648500
C	20.30759700	80.76162300	24.62270800
C	21.20713000	79.67392600	22.67558500
C	21.46998000	81.52919400	24.73642200
C	22.37564100	80.43535500	22.77962500
C	22.50527400	81.37667200	23.80941500
H	19.38674200	77.51056500	24.97508600
H	18.55395100	79.00758800	22.41255000
H	18.08649900	79.47710900	24.04038700
H	19.50452600	80.89215400	25.34469500
H	21.10528900	78.95850000	21.86125600
H	21.56491400	82.24608300	25.54865200
H	23.18284700	80.30037200	22.06488300
H	23.39927400	81.99149000	23.87439200
C	32.71363300	75.93703600	19.99530300
C	32.05793200	77.14721400	20.57394600
N	32.02601800	78.37636500	19.92849700
C	31.37803900	77.35620500	21.74459200
C	31.34411100	79.25914900	20.69905600
N	30.93555700	78.66501300	21.80748300
H	33.78228900	76.09888800	19.80433900
H	32.62459000	75.09936400	20.69224600
H	31.17310000	76.65887000	22.54223900
H	31.17481300	80.30239900	20.46797300
H	32.41525500	78.57773600	19.01853600
C	33.62672200	84.58244200	22.02564600
C	32.52610000	83.83367100	22.77037400
N	31.41192000	84.74866700	23.06028600
C	30.33749100	84.40672800	23.79907100
N	30.26520400	83.23304700	24.41778800
N	29.32680500	85.29074000	23.93350100
H	34.00226800	85.42976400	22.60971200
H	33.26799300	84.95727600	21.05899600
H	32.15911400	82.98146500	22.19030700
H	32.90475400	83.45823500	23.72717500
H	31.25663300	85.48818800	22.38748400
H	30.80217900	82.42347400	24.10420000
H	29.43174300	83.05659000	24.98282000
H	29.51618300	86.27709100	23.82547700
H	28.56546100	85.02108200	24.56891400
Fe	29.83625300	79.56426500	23.36335000
C	25.14663700	81.03316400	26.50686000
C	26.49199300	81.74760700	26.55826800
N	25.27142900	79.60286300	26.95317400
S	26.40041000	83.36019900	25.73756600
O	27.82333300	83.86161200	25.77893300
O	25.91318100	83.09424900	24.35519300
O	25.48405100	84.20931300	26.55329400
H	24.77574100	81.01633000	25.48167800
H	26.81294700	81.92890000	27.58715400
H	27.26278900	81.18584500	26.02020700
H	25.07991800	79.44355300	27.97903200
H	24.63174200	79.01848800	26.37598500
C	31.96019100	79.73257000	24.47101100
O	31.52142600	80.68803100	23.74379400
O	31.24321900	78.68746200	24.56021200
C	33.27523000	79.86185000	25.20333700
C	33.94056900	78.52355300	25.53476600
H	34.17141500	77.96188600	24.62346400
H	34.87523600	78.68787400	26.08073000
O	23.45839100	78.31801000	25.14700800
H	23.80060300	77.43430700	24.86081100
H	23.29069100	78.84971800	24.34652200
H	33.28597100	77.90131500	26.15126500
H	33.92995900	80.49825200	24.59769200
H	33.06761300	80.42620300	26.12385400
O	29.08256200	80.22928400	24.66439800
H	32.24686200	75.63354100	19.04965000
H	24.69056400	79.19615200	18.51545900
H	34.46403600	83.90716600	21.82482600
H	33.43170700	81.78374300	28.87638900
H	29.32566000	82.24030000	35.44291500
H	29.64947000	89.87535600	27.05439700
H	18.11880500	76.99574400	23.83570400
H	19.81463100	77.02938500	23.32574000
H	27.63114900	75.01440700	20.11043800
H	22.46040300	79.14459900	32.56889500
H	30.16816800	75.46747400	27.92482200
H	26.44544200	75.22911500	30.47793900
H	23.58648900	84.81404600	20.56884200
H	19.47786500	86.34423900	22.30522700
H	22.16986500	74.81903500	24.21006100
H	19.85941400	78.08897000	28.97558800
H	24.40533000	81.52532000	27.13900900
H	26.24834500	79.26176700	26.85361900

$^{3}Re_{B}$

C	29.64014900	89.00568200	27.74212500
C	28.34948600	88.25020100	27.64532600
N	27.13943800	88.84926300	27.93421100
C	28.14873700	86.93402600	27.29025100
C	26.23441200	87.90493900	27.75768900
N	26.78879400	86.72429000	27.36722000
H	29.80770300	89.37844700	28.76026000
H	30.49126800	88.37044200	27.47167500
H	28.82506600	86.13815200	27.01852600
H	25.16867100	88.01911300	27.90779900
H	26.30392600	85.85239000	27.14391500
C	28.84554700	83.05400900	34.86415100
C	28.29708800	82.54951700	33.54838500
C	28.00562500	81.19393600	33.35239700
C	28.04120500	83.42987000	32.48672500
C	27.47499900	80.71893300	32.15079700
C	27.50353700	82.98045400	31.28203200
C	27.21406900	81.62119000	31.11230700
O	26.71000500	81.21379500	29.89970400
H	28.05573500	83.48910300	35.49193900
H	29.60105300	83.83423700	34.71415100
H	28.20303300	80.48544500	34.15480400
H	28.25920300	84.48939500	32.60479100
H	27.28079100	79.66015000	32.00030500
H	27.29817800	83.67103100	30.46916000
H	26.23177200	80.32258300	29.96562500
C	32.78434100	82.63159300	29.15564900
C	31.59363300	82.66677500	28.18187700
C	33.61302400	83.92141700	29.18944100
C	30.50125700	83.67886600	28.54914400
H	32.41425800	82.41122300	30.16730600
H	31.96231400	82.88201800	27.16718200
H	31.14178300	81.66606500	28.13603500
H	29.66964300	83.63472700	27.83733500
H	30.88071200	84.70741200	28.54953600
H	30.09445300	83.47655800	29.54782300
H	34.47309900	83.82128100	29.86174800
H	33.99768000	84.16912700	28.19141000
H	33.02182300	84.77544700	29.53754900
C	26.03980200	75.31497500	29.46172700
C	27.16589300	75.26768200	28.43667200
O	27.04578300	74.71155100	27.32997900
C	25.22089800	76.61671900	29.36526700
C	25.94091300	77.87184700	29.88529900
O	26.99202500	77.75754400	30.54748500
O	25.36266400	78.99135700	29.60823100
H	25.38960200	74.45453300	29.27925500
H	24.90433600	76.78772000	28.32835800
H	24.29801100	76.51741600	29.95157000
N	28.30210800	75.90332700	28.80330700
C	29.35907700	76.19242800	27.84041900
C	29.92881200	77.60490700	28.02177900
C	28.84176100	78.67328500	27.99669100
O	27.89823500	78.58699100	27.19342800
N	28.98654300	79.70205000	28.85019100
H	28.93529900	76.07527100	26.84087600
H	30.51149300	77.67297500	28.94864600
H	30.61245300	77.81122500	27.18771900
H	29.63660500	79.61861800	29.61888200
H	28.19737300	80.33313100	29.01609000
H	28.20438900	76.48935700	29.63579100
C	22.41092300	74.71116200	25.27736500
C	23.89960000	74.88424000	25.47659100
O	24.51344300	75.81557500	24.90260100
N	24.52572000	74.02797200	26.29145200
H	21.88694000	75.51362200	25.81512900
H	22.04275000	73.74639300	25.63802800
H	23.99371700	73.31641100	26.77146300
H	25.48855400	74.22038800	26.62053700
C	26.63517800	75.45020300	20.19618700
C	26.65631600	76.73763100	19.36711900
O	27.60020500	77.00429400	18.62993500
C	26.24650300	75.59660700	21.67875400
C	27.10645900	76.54895600	22.45654400
N	26.98563000	76.67261200	23.83051800
C	28.07308200	77.45304800	22.09364400
C	27.84255000	77.62095900	24.25660100
N	28.52054200	78.10772900	23.22440400
H	25.93789300	74.74713400	19.72251200
H	26.29728500	74.60134200	22.14046800
H	25.19290300	75.89750300	21.77593500
H	28.46170400	77.68653700	21.11679100
H	27.95254900	77.93356800	25.28614100
H	26.27863200	76.20586900	24.40894700
N	25.54048100	77.53158300	19.46963800
C	25.60178400	78.91820900	19.04055100
C	25.75714800	79.82276600	20.27552900
O	25.29078300	79.46663700	21.35974700
H	24.97497200	77.41278400	20.30290000
H	26.43986700	79.01695900	18.34856700
N	26.43757100	80.97262400	20.06278800
C	26.54281500	82.03091000	21.05796000
C	25.59215600	83.17372800	20.63510100
O	25.34157300	83.35131300	19.43978800
C	27.97158200	82.59281800	21.11419100
C	29.10752100	81.65557300	21.54589700
O	28.74080300	80.55707300	22.12180100
O	30.27163400	82.03213800	21.33953300
H	26.51686300	81.29699000	19.10474300
H	26.26755000	81.60706600	22.02377300
H	28.24544600	83.02243500	20.14327800
H	27.98285000	83.42455400	21.83042700
N	25.12295200	83.95055100	21.63531700
C	24.26226300	85.10072200	21.37220700
C	25.01881400	86.38191400	20.96683700
C	24.01118600	87.48073000	20.59940200
C	25.97875400	86.85530900	22.06666200
H	23.67191000	85.28390400	22.27653700
H	25.60359300	86.13433100	20.07046700
H	24.52602800	88.39107700	20.26979700
H	23.34833300	87.15905200	19.78679800
H	23.38482000	87.74633300	21.46092300
H	26.70834300	86.07797800	22.32238400
H	26.53329200	87.74547100	21.74421500
H	25.42958200	87.11694300	22.97996800
H	25.38179400	83.73502400	22.59675400
C	19.42078900	86.28253400	23.41578700
C	20.73684000	86.67371200	24.04939400
C	21.10713100	88.02226300	24.15593200
C	21.62679400	85.70179800	24.52673800
C	22.33197800	88.38860500	24.71629200
C	22.85573300	86.06002900	25.09071400
C	23.21060500	87.40844500	25.18507700
H	19.14845800	85.25115000	23.66257800
H	18.60535300	86.93651400	23.74640200

H	20.42695200	88.79249700	23.79734700
H	21.35686400	84.65051800	24.44834500
H	22.59867300	89.43985800	24.79160200
H	23.53769400	85.29531300	25.45391800
H	24.16517000	87.69063300	25.62086000
C	20.85481600	77.61786300	29.01876900
C	21.58150400	77.93272600	30.32570900
O	22.19665500	77.07459200	30.95638100
C	21.71753600	78.09072600	27.84482500
O	21.20282200	77.58250500	26.62440300
H	20.72319700	76.53466500	28.96187600
H	21.74435800	79.19195100	27.82247300
H	22.74965900	77.74695400	28.00697500
H	21.82297500	77.89944000	25.93266600
N	21.55615800	79.24440900	30.69521400
C	22.49987300	79.78416800	31.67161500
C	22.14386000	81.23671400	32.03110200
C	22.05575600	82.16175200	30.82970800
C	20.81458600	82.64419900	30.38975000
C	23.20712000	82.54142100	30.11922700
C	20.72045500	83.48635300	29.27715800
C	23.11866100	83.38621300	29.01102500
C	21.87242900	83.86144500	28.58555900
H	23.51844700	79.72303800	31.26679500
H	21.18915700	81.25015500	32.57102100
H	22.91009500	81.59966100	32.72730700
H	19.91346300	82.37098300	30.93579500
H	24.18267700	82.17907300	30.43447400
H	19.74801700	83.85616300	28.96146900
H	24.01873300	83.68461800	28.47983500
H	21.80866400	84.53050100	27.73109300
H	21.15344200	79.91256200	30.04948100
C	19.06291900	77.54368700	23.93561800
C	18.88718400	78.99956600	23.46719900
C	20.15097500	79.82710600	23.59139700
C	20.30966100	80.75859700	24.62664100
C	21.20515400	79.67010800	22.67815000
C	21.47193600	81.52681900	24.73719000
C	22.37354100	80.43210900	22.77907800
C	22.50520300	81.37406200	23.80800500
H	19.39278700	77.50399000	24.97848400
H	18.55006100	79.00532900	22.42180800
H	18.08840800	79.47172600	24.05217400
H	19.50812300	80.88938600	25.35028500
H	21.10171600	78.95424900	21.86439000
H	21.56823300	82.24446100	25.54859300
H	23.17912900	80.29716600	22.06252900
H	23.39896500	81.98949700	23.87042000
C	32.71555300	75.95879100	19.99674200
C	32.05398000	77.16497500	20.57702700
N	32.01522100	78.39460900	19.93269500
C	31.37382900	77.36949700	21.74831200
C	31.32946800	79.27317100	20.70453300
N	30.92467300	78.67599000	21.81265800
H	33.78336500	76.12612800	19.80581000
H	32.63074600	75.11977100	20.69259600
H	31.17268300	76.67024000	22.54529200
H	31.15573000	80.31608700	20.47506600
H	32.40297100	78.59880700	19.02269300
C	33.64567000	84.55141500	22.03173400
C	32.53831800	83.80811200	22.77195400
N	31.42570000	84.72731300	23.05382900
C	30.34936700	84.39181400	23.79312100
N	30.27495200	83.22331100	24.42152500
N	29.34014700	85.27858500	23.91941700
H	34.02081300	85.39869000	22.61608600
H	33.29372900	84.92562600	21.06231400
H	32.17120400	82.95568400	22.19244600
H	32.90997700	83.43376600	23.73180000
H	31.26971000	85.45799200	22.37149300
H	30.80551200	82.40931500	24.10884500
H	29.43991600	83.05244300	24.98585900
H	29.53156200	86.26368600	23.80399000
H	28.57610100	85.01449100	24.55396600
Fe	29.81956500	79.57390500	23.37815300
C	25.15568600	81.03239300	26.49817100
C	26.50043000	81.74789800	26.54650400
N	25.28386300	79.60148600	26.94211000
S	26.40535700	83.36155600	25.72848000
O	27.82756900	83.86512800	25.76927400
O	25.91636300	83.09745200	24.34643300
O	25.48874500	84.20815000	26.54672100
H	24.78174400	81.01659200	25.47407000
H	26.82352300	81.92866400	27.57481000
H	27.26955100	81.18678300	26.00515000
H	25.09137900	79.44042900	27.96756300
H	24.64554600	79.01674600	26.36367400
C	31.94935400	79.71815200	24.47979900
O	31.52710300	80.67093500	23.73869500
O	31.21744000	78.68459900	24.58158500
C	33.26343200	79.83927200	25.21483100
C	33.91795800	78.49690000	25.55176700
H	34.14680700	77.93083700	24.64262500
H	34.85262900	78.65584300	26.09926900
O	23.46914700	78.31429800	25.13964700
H	23.80979600	77.42996100	24.85408000
H	23.30083100	78.84526900	24.33882900
H	33.25764500	77.88126200	26.16880200
H	33.92443200	80.46875900	24.60884900
H	33.05782700	80.40745200	26.13355800
O	29.07143000	80.24768400	24.65249500
H	32.25013300	75.65425700	19.05076100
H	24.67906700	79.19349800	18.51312200
H	34.48171100	83.87276700	21.83690900
H	33.44284200	81.79379200	28.88337900
H	29.30875300	82.24554600	35.44038300
H	29.64238100	89.88031500	27.07920900
H	18.12057000	76.99085800	23.84301200
H	19.81443000	77.02570500	23.32669500
H	27.63699300	75.02219900	20.10720800
H	22.46654200	79.15330500	32.56432700
H	30.17607200	75.46538600	27.93520300
H	26.44537800	75.22896300	30.47420100
H	23.57490500	84.82145200	20.56722000
H	19.46766600	86.35507000	22.32091700
H	22.17433200	74.81206400	24.21433700
H	19.86664700	78.09154300	28.97137500
H	24.41558500	81.52233200	27.13343600
H	26.26149300	79.26294200	26.84244800

^{7}Re$_{B}$

C	29.67607900	89.06001400	27.66795600
C	28.38392700	88.30572000	27.58244800
N	27.17453000	88.91347200	27.85486100
C	28.18105400	86.98293300	27.25367300
C	26.26774500	87.96793100	27.69473100
N	26.82021900	86.77820000	27.33031700
H	29.83146300	89.46856000	28.67407800
H	30.52828700	88.41276000	27.43166000
H	28.85695200	86.17920200	27.00504000
H	25.20202300	88.08753300	27.84046700
H	26.33298600	85.90448100	27.12022800
C	29.02276300	82.82107900	34.90132400
C	28.43912600	82.37014400	33.58126500
C	28.13221700	81.02483400	33.34232100
C	28.16394900	83.29089300	32.55965400
C	27.56848300	80.59841600	32.13766600
C	27.59351300	82.89002700	31.35274800
C	27.28960500	81.54010200	31.13942800
O	26.75206800	81.18213300	29.92608500
H	28.24810300	83.21676800	35.57264000
H	29.76421500	83.61689300	34.76410200
H	28.34316600	80.28567700	34.11301900
H	28.39316500	84.34377700	32.71120200
H	27.36040600	79.54748700	31.95423700
H	27.37346800	83.61190300	30.57152600
H	26.26591600	80.29413700	29.97117500
C	32.75622900	82.72591600	29.20264100
C	31.57314700	82.75544300	28.21946300
C	33.57722900	84.02045800	29.24388000
C	30.47371600	83.76315700	28.57666700
H	32.37867500	82.50265700	30.21079800
H	31.94979100	82.97170800	27.20764000
H	31.12657700	81.75248000	28.17042200
H	29.64683600	83.71236400	27.85984900
H	30.84845700	84.79346600	28.57736100
H	30.06230800	83.56046200	29.57326300
H	34.43245300	83.92463000	29.92295600
H	33.96863300	84.27106800	28.24919500
H	32.97842700	84.87087400	29.58792000
C	25.96990500	75.29939100	29.36992100
C	27.07955400	75.24700900	28.32691800
O	26.93119900	74.71065900	27.21371600
C	25.17581800	76.61840000	29.30812600
C	25.93057800	77.84934300	29.83662500
O	26.98811900	77.70062800	30.48159200
O	25.37381200	78.98571900	29.58621600
H	25.29997100	74.45501300	29.18383600
H	24.84483000	76.81289200	28.27987500
H	24.26113100	76.52775600	29.90856900
N	28.23232000	75.85545400	28.68709300
C	29.28677800	76.13851500	27.71925100
C	29.88021300	77.53952600	27.91388100
C	28.81187400	78.62588800	27.90365600
O	27.87055900	78.57015700	27.09436800
N	28.97218100	79.63794100	28.77462200
H	28.85420900	76.03891700	26.72170600
H	30.46555500	77.58672400	28.84045800
H	30.56427800	77.74876200	27.08091700
H	29.61678400	79.52883400	29.54472600
H	28.19832600	80.28398400	28.95016400
H	28.15826000	76.42709000	29.53192300
C	22.23823900	74.77576600	25.29544700
C	23.73522400	74.92183100	25.44615100
O	24.34704200	75.83710500	24.84411300
N	24.37223500	74.06074300	26.24692800
H	21.74717200	75.59919600	25.83264300
H	21.86175400	73.82575100	25.68542400
H	23.84360400	73.36174200	26.74872100
H	25.34819600	74.23897400	26.54509900
C	26.58671600	75.31765200	20.10326500
C	26.63982400	76.64479900	19.33860400
O	27.53989500	76.87290100	18.53682300
C	26.21241300	75.38337500	21.59434300
C	27.08742300	76.29627300	22.40088600
N	26.86166900	76.52433300	23.74863700
C	28.15255100	77.09599600	22.07287000
C	27.74728000	77.44051200	24.18212600
N	28.55224800	77.80864500	23.18932200
H	25.87145600	74.66078000	19.59167500
H	26.25848000	74.36355800	22.00094700
H	25.16300000	75.68923300	21.71837300
H	28.63832400	77.22665000	21.11851100
H	27.79659500	77.81111700	25.19764800
H	26.09218400	76.13157300	24.30409300
N	25.59907000	77.51246700	19.55955500
C	25.70188300	78.89438700	19.12765100
C	25.85815900	79.80933800	20.35357500
O	25.46186700	79.43894100	21.46007500
H	25.09280700	77.41447500	20.43261400
H	26.55517400	78.97042800	18.45043500
N	26.45634000	81.00086600	20.10484100
C	26.53389000	82.06644500	21.09519300
C	25.53168100	83.17073900	20.68969600
O	25.23756800	83.31953300	19.50063200
C	27.94331400	82.69189200	21.12701900
C	29.10545200	81.71109100	21.25973600
O	28.90048300	80.68766700	22.04534700
O	30.16120200	81.92150300	20.65774000
H	26.46693900	81.32818900	19.14403200
H	26.29501100	81.63292300	22.06628600
H	28.11624600	83.28351800	20.22211900
H	27.98635100	83.37766200	21.98277600
N	25.07528500	83.94993100	21.69275700
C	24.18862800	85.08290900	21.43852600
C	24.91707900	86.36978400	21.00039000
C	23.88559400	87.44936200	20.64206500
C	25.89484900	86.87078300	22.07192500
H	23.61852100	85.26797400	22.35521600
H	25.48502400	86.11950300	20.09389200
H	24.38004300	88.36262700	20.29021300
H	23.20990100	87.10823600	19.84820000
H	23.27459800	87.71689600	21.51392100
H	26.63866400	86.10615500	22.32496100
H	26.43215500	87.76155000	21.72337200
H	25.36235200	87.14166100	22.99229300
H	25.37120100	83.75539500	22.64850500
C	19.36334400	86.29523700	23.51820000
C	20.68708700	86.69728100	24.12850900
C	21.05107600	88.04846900	24.22253800
C	21.59044500	85.73308700	24.59618100
C	22.28280400	88.42465000	24.76078600
C	22.82623400	86.10103300	25.13843200
C	23.17471200	87.45196300	25.21988700
H	19.10378800	85.26172900	23.76983700
H	18.54854600	86.94258500	23.86309100

H	20.36068200	88.81297100	23.87134600
H	21.32560800	84.67997600	24.52637800
H	22.54464800	89.47775900	24.82623400
H	23.51871500	85.34224600	25.49445600
H	24.13509100	87.74184300	25.63721800
C	20.81610600	77.68965200	29.03328300
C	21.56787400	77.96932300	30.33406700
O	22.17949100	77.09045700	30.93908400
C	21.66619800	78.17690200	27.85574000
O	21.13202500	77.69399500	26.63333100
H	20.67137000	76.60937800	28.95658500
H	21.70120000	79.27831000	27.85264900
H	22.69782900	77.82280500	27.99746400
H	21.75910200	77.99815400	25.94196700
N	21.57002700	79.27440500	30.72680600
C	22.54233000	79.78254300	31.69238100
C	22.22571000	81.23936100	32.07152800
C	22.13314300	82.17598600	30.87946900
C	20.89225600	82.67945000	30.46259300
C	23.27856500	82.54370100	30.15337300
C	20.79251700	83.52952800	29.35660200
C	23.18444700	83.39724000	29.05228400
C	21.93866200	83.89238300	28.64909700
H	23.55207900	79.70273600	31.26911600
H	21.28266100	81.26882200	32.63093500
H	23.01375900	81.57933400	32.75474500
H	19.99612800	82.41598000	31.02151500
H	24.25357400	82.16472500	30.45019800
H	19.82059000	83.91492700	29.05851200
H	24.08012100	83.68842900	28.50964400
H	21.87084200	84.56694100	27.79931700
H	21.16684500	79.95978100	30.09970600
C	19.04933600	77.60980300	23.89525100
C	18.89885000	79.05041300	23.37360500
C	20.16526500	79.87165200	23.51047700
C	20.31577500	80.80502700	24.54544100
C	21.23148700	79.70329400	22.61361100
C	21.48209300	81.56429400	24.67084900
C	22.40426000	80.45638800	22.73062500
C	22.52831500	81.40070200	23.75805900
H	19.34744300	77.60231300	24.94841100
H	18.59432100	79.02398200	22.31875100
H	18.08638400	79.54707300	23.91805000
H	19.50519100	80.94302700	25.25744700
H	21.13474000	78.98522400	21.80108800
H	21.57255000	82.28332900	25.48155200
H	23.22002600	80.31165100	22.02780700
H	23.42582700	82.00902400	23.83278900
C	33.24257400	75.91012700	20.25908900
C	32.43336600	77.07734600	20.71991200
N	32.30824900	78.25198900	19.98889000
C	31.67742500	77.29015600	21.84295000
C	31.50343400	79.10671700	20.66728400
N	31.10353800	78.54673000	21.79677800
H	34.29886900	76.17184100	20.11610000
H	33.19717800	75.11172900	21.00465300
H	31.50395000	76.62746600	22.67770000
H	31.23304300	80.10688600	20.34798100
H	32.72273000	78.43451300	19.08589900
C	33.48881800	84.52520700	21.75897600
C	32.37241200	83.77295900	22.47596100
N	31.34197300	84.72303800	22.92504000
C	30.30214100	84.38885800	23.71764600
N	30.21765900	83.19471200	24.29065300
N	29.32455300	85.29459200	23.93197200
H	33.95832800	85.26619000	22.41494200
H	33.11300900	85.03895800	20.86535800
H	31.92221300	83.01728200	21.82204700
H	32.76567200	83.25942000	23.35759300
H	31.18507500	85.52047900	22.32226200
H	30.79881000	82.39812800	24.01914500
H	29.41883100	83.01364700	24.90202900
H	29.52535100	86.27769200	23.81707500
H	28.59817300	85.03265500	24.61116500
Fe	29.78551600	79.52628500	23.27657200
C	25.15517400	81.06353100	26.48474900
C	26.49196600	81.78767100	26.59388600
N	25.27377800	79.63215700	26.92663900
S	26.43434900	83.38718100	25.74681500
O	27.86078500	83.87705100	25.80973000
O	25.98013400	83.10182000	24.35506300
O	25.50542600	84.26039300	26.52214100
H	24.82925400	81.04745900	25.44425300
H	26.76470100	81.98333900	27.63423400
H	27.28943000	81.22386000	26.09737300
H	25.09483400	79.46994100	27.95396200
H	24.61911300	79.05559400	26.35860800
C	31.96116300	79.88925300	24.58067800
O	31.65720800	80.75780200	23.70195100
O	31.19752500	78.88678700	24.76539100
C	33.22067400	80.06047200	25.40849400
C	33.78956900	78.75902500	25.97779500
H	34.05378300	78.05987400	25.17710700
H	34.69196200	78.96078200	26.56475300
O	23.41258800	78.37233000	25.14688000
H	23.71311700	77.47614700	24.85338700
H	23.25419600	78.90904100	24.34780000
H	33.06267700	78.26180400	26.62575000
H	33.95713400	80.57972800	24.78541400
H	32.96831800	80.75363100	26.22393000
O	28.93017600	80.38148500	24.73058700
H	32.86889200	75.50133800	19.31159600
H	24.79929600	79.19478500	18.57753300
H	34.25807400	83.81951700	21.43117000
H	33.42123800	81.89200600	28.93494900
H	29.51296300	81.99322300	35.42540200
H	29.69032500	89.91033200	26.97412100
H	18.10599400	77.06049700	23.79283700
H	19.81491200	77.06727900	23.32650600
H	27.57552400	74.86785800	19.97968300
H	22.51015000	79.14407900	32.57976400
H	30.09267200	75.39759800	27.80060000
H	26.38888500	75.18862400	30.37448100
H	23.48626800	84.78312300	20.65439400
H	19.39039900	86.36795300	22.42270800
H	21.97178000	74.86227700	24.23820600
H	19.83275300	78.17506800	29.01174200
H	24.38187400	81.54401100	27.08718300
H	26.24639100	79.28225700	26.80757300

$^{5}Re_B$

C	31.21815100	87.72462700	29.51396600
C	29.82255300	87.49410700	29.01913400
N	28.74578500	88.18304600	29.54563400
C	29.39086400	86.63213300	28.03547100
C	27.69290800	87.74006500	28.88273000
N	28.02560600	86.80104900	27.95520000
H	31.29661000	87.52767700	30.59032100
H	31.93110900	87.07212700	28.99818600
H	29.90422100	85.92232600	27.40523600
H	26.67171900	88.06419200	29.03642000
H	27.39547900	86.27393200	27.34646500
C	29.01374800	85.25666700	33.03488000
C	28.26361700	84.23975300	32.20393100
C	28.03383000	82.93954200	32.67868500
C	27.77730800	84.56511900	30.93224900
C	27.34659100	81.99216100	31.92026500
C	27.07580700	83.63459400	30.16118200
C	26.85627300	82.34575000	30.65529500
O	26.18978600	81.42623600	29.87560800
H	29.07013100	86.22111100	32.52022700
H	30.04071400	84.92929400	33.24455000
H	28.40401800	82.65639500	33.66253800
H	27.94111200	85.56407600	30.53800500
H	27.19457300	80.98029000	32.28467100
H	26.69445000	83.91532500	29.18270400
H	25.69343800	80.69163600	30.44936800
C	31.27775900	80.98003800	30.29829800
C	31.48098700	81.49545800	28.86300000
C	32.41807200	81.32352900	31.26442500
C	31.49181900	83.02401500	28.73665000
H	30.33398000	81.37952700	30.69536500
H	32.42451000	81.08801400	28.46689000
H	30.68126900	81.09076500	28.22855000
H	31.60192900	83.32724900	27.68848900
H	32.31886000	83.47461100	29.29762100
H	30.55878300	83.45945000	29.11413700
H	32.23672400	80.89510400	32.25693400
H	33.37671200	80.93066500	30.90094000
H	32.52898600	82.40613900	31.39017000
C	25.39519800	75.93247200	31.54715000
C	26.08032300	75.45618500	30.26867700
O	25.65573000	74.48172400	29.62617000
C	24.64318100	77.26948900	31.38850400
C	25.51866800	78.53575700	31.26828600
O	26.76233600	78.45237900	31.43619000
O	24.87435700	79.60189900	31.00430500
H	24.69494000	75.14407600	31.83583000
H	23.97249600	77.23757400	30.52159200
H	23.99384400	77.41585800	32.26158700
N	27.16119700	76.17886300	29.90262400
C	27.83011200	75.98660900	28.62567400
C	28.67761400	77.20882600	28.24928200
C	27.83704500	78.47486700	28.13003200
O	26.92430000	78.55965600	27.28249400
N	28.14804900	79.47460100	28.97084500
H	27.08070900	75.80717200	27.85235700
H	29.48577000	77.35050800	28.97582400
H	29.14327600	77.02751800	27.27488500
H	28.72885900	79.27332000	29.77344800
H	27.47467900	80.22855200	29.14042000
H	27.32168700	77.02478100	30.46643400
C	26.99104100	71.85076500	25.33267000
C	26.63030600	73.04333500	26.19951300
O	26.67463300	74.19661700	25.72157000
N	26.27539000	72.79915900	27.46497900
H	26.33151800	71.83392900	24.45930300
H	26.91492000	70.89280000	25.85444600
H	26.24387600	71.85071400	27.80876300
H	26.05941700	73.54977400	28.14284300
C	25.41951700	75.99515200	21.49388100
C	25.66022700	77.15178100	20.52350100
O	26.61110500	77.16794600	19.73609800
C	25.35100600	76.38673600	22.99580100
C	26.61212800	77.01708400	23.50261000
N	27.64183700	76.27885100	24.06558400
C	27.08752100	78.30160500	23.42449200
C	28.68063800	77.11280800	24.30574600
N	28.38036600	78.33931000	23.90912400
H	24.47713700	75.50236000	21.21932000
H	25.14587500	75.47567800	23.56851000
H	24.49965400	77.04614300	23.19484500
H	26.59736200	79.18842600	23.05521600
H	29.62663200	76.81721500	24.73013200
H	27.52548400	75.33703500	24.44498600
N	24.71654700	78.13787600	20.55011400
C	24.90747600	79.40617200	19.86993300
C	25.30011500	80.49337900	20.88748900
O	24.91669200	80.40521600	22.05465400
H	24.10911000	78.17924100	21.35997300
H	25.67216600	79.26173100	19.10476500
N	26.09587300	81.48007500	20.40703700
C	26.45522300	82.65247400	21.19640100
C	25.79887500	83.88486200	20.54382700
O	25.60012700	83.90348600	19.32630800
C	27.97849400	82.87354300	21.23204000
C	28.78684700	81.84094200	22.00271200
O	28.17951600	80.84411600	22.53433800
O	30.02378200	81.97802300	22.12609200
H	26.11561300	81.63952800	19.40387300
H	26.08919500	82.49445300	22.20988700
H	28.37727600	82.93288100	20.21107900
H	28.18523100	83.83977900	21.70416000
N	25.52059400	84.89794800	21.39398300
C	24.97993500	86.17570400	20.94451100
C	26.02552400	87.30605500	20.86691900
C	25.38318200	88.56238900	20.26154500
C	26.64522000	87.60705300	22.23912600
H	24.17161800	86.46448200	21.62745400
H	26.82151900	86.96300100	20.18970400
H	26.11533100	89.37275000	20.16595300
H	24.97145800	88.36213400	19.26504100
H	24.56530400	88.92846900	20.89558200
H	27.12682200	86.72201600	22.67012900
H	27.39986400	88.39967400	22.16334300
H	25.87885500	87.94276300	22.94952000
H	25.74114700	84.76974200	22.37987800
C	19.70604900	86.98884600	23.11322000
C	21.04201600	86.89440900	23.81530600
C	21.51721000	87.95042900	24.60470900
C	21.84936200	85.75571400	23.67479100
C	22.76309300	87.87662300	25.23177800
C	23.09579600	85.67716000	24.30126000
C	23.56071900	86.73938900	25.08309200
H	19.24368900	86.00261900	22.99647400
H	19.00735800	87.62797800	23.66419400

H	20.90199600	88.83899600	24.73209500
H	21.49552000	84.92124800	23.07266300
H	23.10758600	88.70711600	25.84321000
H	23.71325200	84.79055800	24.18396700
H	24.52745100	86.65993100	25.57131500
C	22.08200000	77.85393900	27.14115100
C	22.12239500	79.00582500	28.13174000
O	22.92886800	79.93668300	28.03113400
C	22.41051800	78.26879100	25.69812800
O	22.76248500	77.14668200	24.90618800
H	22.86072000	77.14936600	27.46658900
H	21.54832500	78.74282000	25.22221600
H	23.22050200	79.01021800	25.71608200
H	23.58352200	76.78846800	25.31356400
N	21.27567200	78.93188100	29.18685400
C	21.41959000	79.75449400	30.39383900
C	20.66081600	81.09884100	30.37330600
C	21.29863200	82.20296800	29.55305800
C	20.64792700	82.75149900	28.44226400
C	22.55203400	82.71919500	29.91491700
C	21.23317500	83.78395800	27.70360500
C	23.14050000	83.74879300	29.18166300
C	22.48317500	84.28631100	28.06948700
H	22.48918500	79.90761800	30.56987600
H	19.63166700	80.92521300	30.03457300
H	20.59056600	81.42943500	31.41957400
H	19.67246600	82.36645300	28.15157400
H	23.07887800	82.30594100	30.77180800
H	20.71102300	84.20436200	26.84764700
H	24.10799000	84.13907000	29.48650300
H	22.93683800	85.09138500	27.49850300
H	20.63859900	78.14889900	29.21936900
C	19.70088800	77.07150900	22.85558700
C	19.65945200	78.30771500	21.93680700
C	20.07514600	79.58048900	22.64591900
C	19.17654000	80.27042700	23.47388500
C	21.37968800	80.07685800	22.53225800
C	19.57566200	81.41012000	24.17372500
C	21.78761900	81.21624300	23.22859800
C	20.88499900	81.88593100	24.05704600
H	19.00090600	77.18714000	23.69162000
H	20.31850200	78.13998100	21.07515800
H	18.64471500	78.42309100	21.53272500
H	18.15437300	79.90870800	23.57019200
H	22.09570500	79.56221600	21.89615200
H	18.86405300	81.92885100	24.81159500
H	22.81135800	81.56253500	23.11976000
H	21.19826600	82.76566200	24.61212500
C	31.41368100	76.09299000	19.64002200
C	30.72757400	77.19500600	20.37797900
N	29.44896500	77.63563800	20.06981900
C	31.12862600	77.98676700	21.42504500
C	29.12688300	78.63285000	20.91590500
N	30.12197600	78.87498700	21.75630500
H	31.57211900	76.34355300	18.58344000
H	32.39242100	75.89850300	20.08726200
H	32.06091800	77.96327800	21.96646300
H	28.17608200	79.13541700	20.91523900
H	28.75484400	77.20423400	19.46726800
C	34.38888900	83.45568600	21.75021400
C	33.25053600	83.06781300	22.68879100
N	32.30818000	84.18697600	22.81730900
C	31.26270200	84.18525100	23.67105200

N	31.11276500	83.21630000	24.57843500
N	30.40494900	85.21611600	23.65020100
H	34.92897400	84.33447200	22.11856400
H	34.01595300	83.67411500	20.74172000
H	32.72826800	82.17432400	22.33163900
H	33.64127800	82.84744600	23.68762700
H	32.17109000	84.73840000	21.98006800
H	31.40873200	82.26884200	24.33789800
H	30.25521200	83.26426400	25.13030500
H	30.67011100	86.09057700	23.22304200
H	29.58808600	85.17751200	24.27573100
Fe	29.74746700	79.94199100	23.52891800
C	25.46541100	82.01659400	26.23415100
C	26.91809300	82.48738400	26.19142800
N	25.31549000	80.93514600	27.25520500
S	27.08621900	84.13647700	25.45708000
O	28.57067400	84.39306800	25.51090700
O	26.55092000	84.04582700	24.06930800
O	26.32138900	85.06423100	26.33982400
H	25.14650600	81.62568300	25.26566800
H	27.32906500	82.58831800	27.20082900
H	27.55867300	81.81071600	25.61791800
H	25.54058100	81.29310900	28.20868300
H	24.34258000	80.56008400	27.32413000
C	32.09722400	79.29942000	24.49005000
O	31.73347700	80.32638300	23.79199000
O	31.31324200	78.34808400	24.66973700
C	33.49838100	79.32281500	25.08072600
C	33.96472500	77.97511600	25.63086800
H	34.00140100	77.21662300	24.84160100
H	34.96582900	78.06311000	26.06637400
O	25.06433100	76.48123700	26.35965400
H	25.58254800	75.66443300	26.21620900
H	25.72167600	77.17869100	26.55670600
H	33.28090400	77.61368400	26.40385800
H	34.18359600	79.70266700	24.31292900
H	33.48987200	80.08218000	25.87533500
O	29.40686700	80.72174800	24.91152600
H	30.83910000	75.15871700	19.67572600
H	23.97516900	79.70645500	19.37462300
H	35.09847000	82.62712800	21.66430500
H	31.16416700	79.88572900	30.26535600
H	28.52760200	85.42341800	34.00486600
H	31.53202600	88.76434500	29.35588400
H	19.41689300	76.16676200	22.30410300
H	20.70008300	76.92230400	23.27955700
H	26.22881300	75.27970400	21.32501700
H	21.03389300	79.14901800	31.22176100
H	28.47840500	75.10034100	28.65866900
H	26.14298400	76.04150900	32.34068300
H	24.54693800	86.00108500	19.95619800
H	19.81258400	87.41682500	22.10698600
H	28.01567300	71.97400000	24.96712900
H	21.12596600	77.31788700	27.18144600
H	24.79490500	82.82616200	26.53267300
H	25.94654900	80.11942500	27.09863800

[5]TS1$_{HA,C1S,B}$

C	29.05690800	88.44997800	29.56030700
C	27.94672400	87.53447500	29.14334400
N	26.80936600	87.37998300	29.91449900
C	27.85844000	86.74864500	28.01682100
C	26.05608000	86.51914900	29.25685400
N	26.63985500	86.10656900	28.09686800
H	29.45402400	88.16974000	30.54373200
H	29.88225100	88.42228000	28.84040800
H	28.53311600	86.58095600	27.19097200
H	25.08702300	86.15625000	29.57389400
H	26.28299200	85.40491800	27.44680100
C	27.49035100	83.93277800	34.29194500
C	27.34450600	83.18424500	32.98602200
C	27.21414800	81.78927500	32.95792900
C	27.35461300	83.86538300	31.75984300
C	27.10562300	81.08361400	31.75740000
C	27.24046200	83.17979400	30.55066400
C	27.11202900	81.78682400	30.54663100
O	27.00408400	81.13800000	29.33627100
H	28.54394400	84.14431900	34.52175700
H	27.08494900	83.35714300	35.13151300
H	27.20183100	81.23601900	33.89527300
H	27.43088300	84.95048000	31.74018300
H	27.02676400	79.99970300	31.74407200
H	27.22589600	83.71884600	29.60857500
H	26.44921300	80.29263700	29.43365200
C	32.68282500	82.62995300	29.31125000
C	31.64480500	82.81119800	28.19013000
C	33.64453900	83.81227800	29.48157200
C	30.63634500	83.94051600	28.43453300
H	32.15744400	82.45009600	30.26025900
H	32.17010600	82.98917300	27.23909600
H	31.09469400	81.86812300	28.06391300
H	29.89629100	83.98992200	27.62859100
H	31.12505300	84.91986100	28.49445800
H	30.08895000	83.78562100	29.37210100
H	34.38561700	83.61033500	30.26382000
H	34.19010900	84.01391300	28.55036300
H	33.11416100	84.72941600	29.76007100
C	26.06960800	75.33165400	29.45369900
C	27.22347500	75.16589500	28.47375600
O	27.10630400	74.56571100	27.38958100
C	25.28294300	76.63418200	29.21724600
C	26.03130100	77.91855000	29.60723500
O	27.05529800	77.85422300	30.31371000
O	25.49438400	79.01639800	29.18363500
H	25.40322800	74.47242500	29.33193600
H	24.96089400	76.70424300	28.17091300
H	24.36919400	76.61548300	29.82541000
N	28.38449300	75.74478100	28.85766400
C	29.49690100	75.91760300	27.92922800
C	30.14576300	77.29950800	28.06608500
C	29.12987900	78.42792800	27.93335500
O	28.19149800	78.32887500	27.12848900
N	29.32860900	79.51616600	28.70036300
H	29.10918800	75.77357400	26.91865500
H	30.69033900	77.38137500	29.01494200
H	30.87457100	77.42454400	27.25542900
H	29.96839100	79.45246300	29.47950400
H	28.56106100	80.18782000	28.81465900
H	28.28275900	76.39664600	29.63792800
C	22.36090300	74.52605800	25.61175900
C	23.86576800	74.65448700	25.65906900
O	24.45116600	75.51587300	24.95999400
N	24.53942600	73.84073400	26.48008800
H	21.92658000	75.38179700	26.14704000
H	21.99779000	73.60065700	26.06808700
H	24.02980300	73.19322900	27.06420200
H	25.52332400	74.04233600	26.73338000
C	26.40506200	75.51895600	20.07350200
C	26.45801900	76.82745000	19.27824100
O	27.33696700	77.02133300	18.44532000
C	25.99530700	75.59543000	21.55473500
C	26.88696600	76.45502500	22.40274900
N	26.84243000	76.40889800	23.78763800
C	27.81592100	77.42041200	22.10579600
C	27.70390000	77.32395300	24.27509700
N	28.31093400	77.95757400	23.27845300
H	25.70675500	74.84889400	19.55494100
H	25.99229300	74.57352000	21.95612200
H	24.95235500	75.93484500	21.64807500
H	28.15970200	77.76958200	21.14540600
H	27.89098600	77.49429900	25.32696100
H	26.17335400	75.87043500	24.34915700
N	25.43525600	77.71588100	19.50374600
C	25.54114400	79.08151600	19.02282400
C	25.76045800	80.02827100	20.21419400
O	25.36669600	79.70798300	21.33831500
H	24.95478200	77.65424600	20.39427000
H	26.36662400	79.12098100	18.30913800
N	26.40537000	81.18089300	19.92033400
C	26.56862100	82.26873300	20.87512500
C	25.64824300	83.42987200	20.44379700
O	25.42305800	83.62331900	19.24680100
C	28.01502400	82.79080800	20.87832000
C	29.11876800	81.79754700	21.25735300
O	28.75877900	80.79994100	21.99917000
O	30.27133400	82.03010700	20.86660100
H	26.45572000	81.46424200	18.94755800
H	26.31162200	81.88454600	21.86141000
H	28.26642500	83.21865300	19.90172300
H	28.07280800	83.61059800	21.60676400
N	25.18299900	84.20677200	21.44792100
C	24.39810600	85.41064100	21.19913100
C	25.24492200	86.68816000	21.02287300
C	24.33091500	87.86985100	20.66960900
C	26.08615500	86.99396200	22.27028600
H	23.70338300	85.53762100	22.03714300
H	25.92281600	86.50682300	20.17678300
H	24.91371300	88.78367500	20.50373800
H	23.75531000	87.66989600	19.75784100
H	23.61933600	88.07138100	21.48073900
H	26.73947700	86.15066600	22.52651800
H	26.71505000	87.87915700	22.11072000
H	25.44437300	87.19223500	23.13798400
H	25.42994500	83.96476700	22.40510600
C	19.73749200	86.45781200	23.10916900
C	20.99703500	86.69852100	23.91031800
C	21.36194700	87.99319200	24.30553600
C	21.81769400	85.62995600	24.30064900
C	22.50447400	88.21498800	25.07792300
C	22.96505100	85.84481800	25.06992300
C	23.30921000	87.14110900	25.46488500
H	18.87079400	86.30506200	23.76673300
H	19.50455200	87.30947800	22.46054100

H	20.74131200	88.83609000	24.00795000
H	21.55572900	84.61797000	23.99719500
H	22.76500100	89.22712200	25.37724000
H	23.59999600	85.01086900	25.35583500
H	24.19652800	87.30606100	26.07036700
C	21.20791300	77.70101200	29.22581100
C	21.92886200	78.14551800	30.49690000
O	22.58944500	77.36608500	31.18144600
C	21.89930500	78.29580400	27.99150600
O	21.51405900	77.56538500	26.83479200
H	21.27326400	76.61145300	29.18205500
H	21.62897500	79.35882600	27.88055200
H	22.98799500	78.25800900	28.13533600
H	22.12787800	77.84038800	26.12063400
N	21.83643900	79.47575200	30.78259600
C	22.77317600	80.12218400	31.69933700
C	22.35508400	81.57624300	31.97532500
C	22.17273100	82.40177800	30.71425800
C	20.90035000	82.84558900	30.32660200
C	23.26602500	82.71067900	29.88852900
C	20.72113000	83.58543500	29.15385900
C	23.09161300	83.44430400	28.71406800
C	21.81654100	83.88837200	28.34439200
H	23.78521500	80.08744500	31.27415800
H	21.42225600	81.58111700	32.55215300
H	23.12888700	82.02536700	32.61040500
H	20.04295900	82.62029900	30.95795100
H	24.26288400	82.37910300	30.16788300
H	19.72697600	83.92997000	28.87991300
H	23.94943500	83.66973400	28.08633400
H	21.68658400	84.47643800	27.43951900
H	21.40637900	80.07903500	30.09205800
C	19.28851000	77.32490300	24.21333500
C	19.03732300	78.76879400	23.74202500
C	20.27767700	79.63873500	23.78059900
C	20.46254200	80.59517000	24.78767400
C	21.28111000	79.50326500	22.80734600
C	21.59879200	81.40945000	24.81449100
C	22.42281900	80.31080900	22.82470200
C	22.57890000	81.27621100	23.82796000
H	19.68482500	77.31087400	25.23361400
H	18.63704900	78.75182500	22.71925500
H	18.26080200	79.22177900	24.37042900
H	19.70050600	80.71242900	25.55497000
H	21.15382500	78.77215000	22.01048200
H	21.71150500	82.14874100	25.60387400
H	23.18888700	80.19877700	22.06246700
H	23.45210000	81.92327000	23.82501600
C	32.78091200	75.82044700	20.53774600
C	32.05206900	77.05439800	20.95718200
N	32.05048500	78.22640000	20.21184100
C	31.27291000	77.34112700	22.04747900
C	31.28996600	79.15014000	20.85265300
N	30.80295200	78.63892700	21.96982200
H	33.85801600	75.99714700	20.42070600
H	32.65384600	75.04092400	21.29389100
H	31.01782200	76.70559300	22.88184300
H	31.12271900	80.16774900	20.52030400
H	32.51136800	78.36446900	19.32382500
C	32.94776700	84.01971200	20.83636500
C	32.47997600	83.71912400	22.25667000
N	31.46274900	84.69552700	22.66357400
C	30.50960600	84.49924800	23.58832300

N	30.41864000	83.37443900	24.28440400
N	29.63986400	85.50399400	23.86157600
H	33.35106000	85.03686600	20.74500400
H	32.11575700	83.89221600	20.13875700
H	32.02746700	82.72662200	22.27592200
H	33.32017100	83.74444100	22.96464100
H	31.56302600	85.63655900	22.30826100
H	30.98376300	82.54167000	24.10099400
H	29.57258300	83.24547600	24.84396900
H	29.47220500	86.20422500	23.15323700
H	28.84048700	85.24813700	24.44843000
Fe	29.49270700	79.71367900	23.45916700
C	25.59078600	80.82920600	25.87642500
C	26.82175600	81.67746100	26.07949700
N	25.72306200	79.47360400	26.49339100
S	26.61768200	83.34252600	25.43869700
O	27.97361600	83.97919100	25.56167000
O	26.14832200	83.19419800	24.03630400
O	25.61657100	83.98113700	26.34385000
H	25.40071600	80.69495400	24.80963400
H	27.20156700	81.70580800	27.10402400
H	27.80364200	81.10534600	25.34221000
H	25.49114700	79.42095200	27.52386500
H	25.07433000	78.83048000	25.99344700
C	31.72500800	79.82248900	24.69011200
O	31.34039700	80.75816200	23.90199500
O	30.98644000	78.81343600	24.85159000
C	33.05182000	79.96027000	25.41160700
C	33.74065800	78.62595900	25.70960200
H	33.94938400	78.07599800	24.78548900
H	34.69022100	78.79284700	26.22912300
O	23.65697900	78.10815100	25.08717200
H	23.86444100	77.16932200	24.85106200
H	23.36992900	78.57078300	24.27804500
H	33.11222800	77.98987400	26.33896300
H	33.69333900	80.61374100	24.81095600
H	32.84870600	80.50193400	26.34637700
O	28.58989200	80.45001200	24.77575500
H	32.40338400	75.42427100	19.58620600
H	24.62033700	79.37822400	18.50201000
H	33.74466800	83.32374000	20.55595700
H	33.26971100	81.72177500	29.11027300
H	26.97011300	84.89686900	34.26162700
H	28.70995700	89.48799900	29.63978100
H	18.36025900	76.74199900	24.18654900
H	20.01572200	76.82304400	23.56263600
H	27.40006900	75.07606700	19.97901700
H	22.79402700	79.54821100	32.62956800
H	30.26012000	75.14698600	28.09870600
H	26.44270000	75.32928700	30.48195300
H	23.81269700	85.23591300	20.29176900
H	19.82633800	85.56627200	22.47912000
H	22.02399500	74.57325000	24.57259300
H	20.14709200	77.98082800	29.23856600
H	24.71440100	81.31268700	26.32155200
H	26.69991400	79.12250800	26.45899800

^{5}TS1$_{HA,C1R,B}$

C	28.23655800	88.29615300	29.41786600
C	27.15433800	87.58360700	28.66554400
N	25.87144400	88.09161900	28.59135300
C	27.23009100	86.39556900	27.97334600
C	25.19309300	87.22160900	27.86929100
N	25.96601400	86.17352600	27.46650700
H	27.96997200	88.42028800	30.47476200
H	29.18087000	87.74301600	29.36875600
H	28.03745400	85.70047300	27.80033900
H	24.14405300	87.29500300	27.61431600
H	25.68454700	85.38387300	26.88633100
C	27.90699900	83.03245800	34.86436200
C	27.72264500	82.42865100	33.49021800
C	27.52657200	81.05178400	33.32002400
C	27.76161700	83.22745400	32.33863400
C	27.38498400	80.47931700	32.05472200
C	27.61306500	82.67849700	31.06497600
C	27.42411400	81.29870700	30.91872100
O	27.29750800	80.78007500	29.65400900
H	28.96959400	83.17219800	35.10713200
H	27.47866700	82.39254300	35.64360400
H	27.48868800	80.40655100	34.19568700
H	27.89897700	84.30236000	32.43687100
H	27.25608200	79.40700400	31.93272700
H	27.62513400	83.30956700	30.18095400
H	26.72249800	79.94428500	29.67436200
C	32.89438800	82.71394000	29.25229500
C	31.69914400	82.70799400	28.28385200
C	33.69828400	84.01992700	29.25943600
C	30.59109400	83.70769300	28.63670100
H	32.53259200	82.50391800	30.26904300
H	32.05934500	82.91217200	27.26399300
H	31.26846100	81.69753300	28.25995000
H	29.74992200	83.62079900	27.93953200
H	30.94921700	84.74325700	28.59877200
H	30.20354800	83.52419300	29.64611900
H	34.56213100	83.94868300	29.93057600
H	34.07549200	84.25642000	28.25585400
H	33.09235800	84.86937800	29.59366800
C	26.03734100	75.00890200	29.32328700
C	27.16936700	74.85407700	28.31564500
O	27.01507700	74.32145300	27.20151500
C	25.33509600	76.37375700	29.19963200
C	26.17360100	77.57503400	29.66221200
O	27.19390400	77.39860600	30.35679900
O	25.71477500	78.73017800	29.31085200
H	25.31751600	74.20477900	29.14442900
H	25.00072800	76.54311000	28.16903500
H	24.43360700	76.37505100	29.82654300
N	28.35343100	75.37253100	28.71352400
C	29.46359500	75.56354000	27.78733600
C	30.21716100	76.86520200	28.07718900
C	29.31242000	78.09108500	27.99826500
O	28.41274400	78.14806500	27.14890600
N	29.57411800	79.09797200	28.85793200
H	29.06100800	75.57268800	26.77180700
H	30.71628500	76.81514000	29.05293100
H	31.00033100	76.99084500	27.31952700
H	30.13790600	78.90133800	29.67326200
H	28.85922000	79.82107000	28.99237000
H	28.28657500	75.97329400	29.53849200
C	22.29752000	74.87569500	25.42227700
C	23.80473800	74.86702300	25.52063700
O	24.48381000	75.74412600	24.93684400
N	24.38331300	73.91041700	26.25716900
H	21.92582500	75.73063800	26.00307300
H	21.83853700	73.95719800	25.79914700
H	23.80700500	73.24595800	26.75329500
H	25.37786200	73.99096300	26.53384400
C	26.35953500	75.45218000	20.09050700
C	26.37954600	76.74090700	19.26292600
O	27.19386000	76.89154500	18.35850300
C	26.00266900	75.55769000	21.58244400
C	26.91824300	76.43933700	22.38106000
N	26.91046100	76.42579900	23.76691600
C	27.83928500	77.39727500	22.03725600
C	27.77985600	77.35400800	24.21112500
N	28.36228700	77.96272600	23.18450300
H	25.64653000	74.76926600	19.61011500
H	26.02136100	74.54447900	22.00501300
H	24.96185600	75.89306000	21.70800600
H	28.16032300	77.72292500	21.06049800
H	27.99296100	77.54406400	25.25440900
H	26.23649200	75.92158800	24.35343800
N	25.40194800	77.66186200	19.54644500
C	25.49061000	79.00700700	19.00827100
C	25.76092800	80.00073300	20.14802100
O	25.37136700	79.75263300	21.29208600
H	24.97883900	77.63378700	20.46709300
H	26.28341400	79.01375800	18.25741500
N	26.44548300	81.11152500	19.78596400
C	26.61886200	82.25829800	20.66722700
C	25.68396400	83.38371600	20.17301300
O	25.42953500	83.48691400	18.97104800
C	28.06391200	82.77801000	20.64071900
C	29.15943600	81.84238200	21.16156300
O	28.77120400	80.86353100	21.91890500
O	30.33275800	82.09896100	20.86070400
H	26.47880700	81.34019600	18.79785500
H	26.37016600	81.93672900	21.67676000
H	28.34059500	83.08990300	19.62745600
H	28.10989700	83.67640600	21.26887600
N	25.22771900	84.21985500	21.13288600
C	24.42797300	85.39977300	20.82367300
C	25.25566800	86.68836800	20.63814500
C	24.33714300	87.83362000	20.18848600
C	26.02291700	87.06589000	21.91370900
H	23.69929500	85.53730200	21.63146200
H	25.98135300	86.48847100	19.83686200
H	24.90898800	88.75351600	20.01830900
H	23.81576500	87.58556400	19.25618100
H	23.57889400	88.05002000	20.95203700
H	26.66423300	86.24545800	22.25698000
H	26.65463400	87.94668000	21.74308600
H	25.32745200	87.30658800	22.72764000
H	25.53641800	84.06633900	22.09066200
C	19.25031300	85.95919500	22.98419700
C	20.53999000	86.48737100	23.57058000
C	20.74100200	87.86529800	23.73753300
C	21.55655800	85.61396000	23.98081800
C	21.91816100	88.35663000	24.30375200
C	22.73940500	86.09824400	24.54786500
C	22.92212400	87.47446700	24.71171000
H	18.87912200	86.60572700	22.18085800
H	19.38000000	84.95170800	22.57541000

H	19.96585200	88.55991500	23.41989500
H	21.42346200	84.54153400	23.85378100
H	22.05254500	89.42849900	24.42561600
H	23.51798800	85.40259800	24.85079400
H	23.83902200	87.85459300	25.15427500
C	21.50430700	77.76882100	29.21959300
C	22.14633000	78.15086300	30.55266200
O	22.75404800	77.34126800	31.24955300
C	22.22444300	78.45999200	28.05583400
O	21.83106500	77.85168900	26.83296000
H	21.59973900	76.68592100	29.11127200
H	21.97913500	79.53470300	28.04199900
H	23.30944800	78.38083200	28.20791300
H	22.45008400	78.17972100	26.14575800
N	22.02746700	79.46808200	30.88633200
C	22.87207000	80.08484000	31.90489900
C	22.44950500	81.54703600	32.14235200
C	22.43019500	82.37340000	30.86966200
C	21.21825300	82.68540900	30.23577200
C	23.62511600	82.79943900	30.26823200
C	21.19655500	83.39512000	29.03062700
C	23.60702700	83.50838400	29.06672100
C	22.39218800	83.80577100	28.44007700
H	23.92466200	80.03642700	31.59389200
H	21.45564200	81.56297900	32.60612400
H	23.15022400	81.98138100	32.86539900
H	20.28113500	82.38040700	30.69806000
H	24.57691800	82.57940600	30.74621700
H	20.24485700	83.63577600	28.56315500
H	24.54007700	83.83996700	28.62139800
H	22.38094200	84.36383400	27.50754900
H	21.65441100	80.09684400	30.18568400
C	18.89060600	78.49814000	25.10523500
C	19.05589100	79.98305900	24.73801900
C	20.44011400	80.34005800	24.23052400
C	21.23611600	81.28165800	24.89883200
C	20.95680400	79.74386700	23.06943200
C	22.49962800	81.63741700	24.41729900
C	22.22267400	80.08297000	22.58735100
C	22.99646300	81.04031700	23.25444800
H	19.63518400	78.19670300	25.84876300
H	18.31118200	80.25215100	23.97518600
H	18.82901300	80.60052100	25.61567300
H	20.86057800	81.74959300	25.80634600
H	20.35618000	79.01426600	22.53040600
H	23.09200200	82.38198800	24.94388500
H	22.61246200	79.62212300	21.68409800
H	23.96406800	81.31659400	22.84745000
C	32.85971700	75.92164700	20.42893100
C	32.13305200	77.14977700	20.86875100
N	32.21664500	78.36355900	20.19819700
C	31.28239100	77.39462900	21.91466400
C	31.43533800	79.27033000	20.83771000
N	30.85270500	78.70827500	21.88276200
H	33.94743900	76.06892800	20.41305700
H	32.64717100	75.09974200	21.11799200
H	30.94862100	76.71787900	22.68646400
H	31.30948900	80.30815400	20.55267000
H	32.74778500	78.54074300	19.35756100
C	32.72400300	84.40133400	21.19550200
C	32.26927300	83.92979100	22.57282600
N	31.24159900	84.83762600	23.09880700
C	30.33528700	84.53518800	24.04467900

N	30.26427300	83.33118200	24.59693800
N	29.50266300	85.50757700	24.49032300
H	33.09882900	85.43318400	21.21791400
H	31.89701600	84.32707600	20.48453200
H	31.83571400	82.93335800	22.47600800
H	33.11425900	83.88304700	23.27437200
H	31.35629400	85.82026200	22.88860900
H	30.81864600	82.52964100	24.28321000
H	29.42792000	83.12530100	25.14733300
H	29.29853000	86.27735800	23.86794400
H	28.71302400	85.17624400	25.05109100
Fe	29.49836000	79.75157900	23.35645700
C	26.05727400	81.08372900	26.64375500
C	26.38368800	81.54019100	25.23934600
N	26.06873600	79.59562800	26.74735300
S	26.50997200	83.33093300	25.08905600
O	27.72991000	83.71729100	25.88064600
O	26.65430600	83.62285400	23.63888200
O	25.25351800	83.85234200	25.70773500
H	25.05842000	81.43107500	26.92772600
H	27.54538200	81.05696400	24.87004700
H	25.71504400	81.15035700	24.46763700
H	25.79182800	79.27274800	27.71725800
H	25.41551900	79.15705200	26.06546500
C	31.68387100	79.88865900	24.65193600
O	31.30178600	80.82596900	23.86000300
O	30.95946100	78.86806900	24.78667900
C	33.01436200	80.03434300	25.36402700
C	33.27402300	78.98944200	26.44818700
H	33.24395600	77.97758000	26.03276500
H	34.25776700	79.14596700	26.90375300
O	23.99326200	78.44203700	25.11942800
H	24.12340300	77.48699200	24.89651600
H	23.71550600	78.90136300	24.30512200
H	32.51798200	79.05372400	27.23694000
H	33.79136200	79.99570300	24.58776500
H	33.05535000	81.04974800	25.77602800
O	28.54822900	80.42244700	24.70404100
H	32.55245800	75.60043400	19.42529100
H	24.54889300	79.28944000	18.51726400
H	33.53846400	83.76232200	20.84032600
H	33.56626900	81.88416000	28.99059600
H	27.42924700	84.01619900	34.93883300
H	28.40864400	89.30118600	29.01235300
H	17.88816900	78.31110600	25.50799000
H	19.01635800	77.85605400	24.22508300
H	27.35153100	75.00878000	19.96866500
H	22.78142100	79.50724000	32.82928900
H	30.16600500	74.72244100	27.85261400
H	26.42119200	74.90195900	30.34218900
H	23.87991900	85.18155000	19.90302700
H	18.45954500	85.90433700	23.74476700
H	22.00358500	75.02507200	24.37943700
H	20.43612600	78.02016400	29.20260400
H	26.76742600	81.47057300	27.37709200
H	27.03182500	79.22307300	26.63366200

^{5}TS1$_{HA,C2S,B}$

C	29.37974000	89.91162900	26.46328600
C	28.44451800	88.75383100	26.29815200
N	28.31472200	88.08960500	25.08847000
C	27.60661000	88.18586300	27.23056100
C	27.40194200	87.15478500	25.30143600
N	26.93844200	87.17100000	26.57705300
H	29.10475000	90.74373900	25.80351500
H	29.37083200	90.27968200	27.49435700
H	27.42786400	88.41142000	28.27137200
H	27.06337700	86.42354000	24.58055800
H	26.39270500	86.40576800	26.98106200
C	28.15138000	83.21360500	34.99822100
C	27.95743000	82.60058400	33.62964400
C	27.49639300	81.28569100	33.48089300
C	28.21009700	83.33614700	32.46329000
C	27.28666200	80.71569800	32.22373000
C	27.99894700	82.79135500	31.19711000
C	27.53014300	81.47829700	31.07350300
O	27.35891600	80.96510600	29.81290400
H	28.94582300	83.96847000	34.99103000
H	28.41620900	82.45565300	35.74408500
H	27.30456800	80.68507200	34.36823300
H	28.57429600	84.35843000	32.54455800
H	26.96505900	79.68330400	32.11711400
H	28.19342300	83.36887800	30.29800000
H	26.67303900	80.21725000	29.79623800
C	33.31988900	82.23176200	29.09945400
C	31.96897000	82.38908900	28.38030600
C	34.30476800	83.38255000	28.85846800
C	31.12554100	83.57624600	28.85990200
H	33.14133500	82.12739700	30.17960000
H	32.14345900	82.48685200	27.29825900
H	31.38980600	81.46481600	28.51052300
H	30.16453300	83.60707500	28.33510400
H	31.63043200	84.53324200	28.68302500
H	30.91809500	83.50126000	29.93494400
H	35.26191900	83.19679100	29.35993900
H	34.50983000	83.50859000	27.78719600
H	33.91611400	84.33560700	29.23406700
C	25.53413700	75.37450300	29.69970900
C	26.78610200	74.96902300	28.93335400
O	26.74786700	74.28492900	27.89440900
C	25.03487200	76.77040800	29.28150100
C	25.88788400	77.94078700	29.79638300
O	26.76061100	77.73925600	30.66724200
O	25.60338200	79.09428000	29.29658900
H	24.75966000	74.62960900	29.49749400
H	24.95705800	76.83792600	28.18979300
H	24.01793300	76.91648100	29.66709500
N	27.94276100	75.44664800	29.44668000
C	29.19219100	75.41010800	28.69580200
C	30.02198900	76.67630500	28.93355500
C	29.25798100	77.94521100	28.56020900
O	28.50725600	77.95058900	27.57144600
N	29.47155700	79.02669100	29.33075400
H	28.94698400	75.30639900	27.63604300
H	30.36050800	76.72606600	29.97583300
H	30.92331800	76.62748400	28.30903200
H	29.91206900	78.90338700	30.23163900
H	28.82408700	79.81927200	29.27479400
H	27.81196800	76.16488100	30.16370100
C	22.43577100	74.73691400	25.22224900
C	23.88612600	74.67978400	25.65070300
O	24.75103600	75.35411800	25.04657700
N	24.21169900	73.89001200	26.68093100
H	22.07597200	75.76901900	25.32542100
H	21.78876400	74.07208700	25.80123300
H	23.49273700	73.39013900	27.18351700
H	25.15908500	73.94299400	27.09417500
C	26.01336200	75.67696900	20.58769700
C	25.60833300	77.11674000	20.26413700
O	26.15199000	77.74422900	19.36206800
C	26.15936900	75.32579600	22.07760000
C	27.08015600	76.24496300	22.82378100
N	27.28355900	76.13236100	24.19126100
C	27.83592500	77.32250200	22.43688000
C	28.10980200	77.12554300	24.58545700
N	28.46433100	77.86319100	23.54156300
H	25.27452200	75.00148500	20.13722200
H	26.52302500	74.29123300	22.14968500
H	25.18283500	75.31419700	22.58056500
H	27.95606000	77.75908500	21.45793700
H	28.42325400	77.28460400	25.60875500
H	26.72025800	75.54601200	24.80797100
N	24.57970000	77.64537500	20.99943500
C	24.22195100	79.04381700	20.85593500
C	25.07509200	79.97825800	21.72764700
O	25.29984800	79.71687000	22.91986400
H	24.27610700	77.16872800	21.83601300
H	24.31682800	79.30085400	19.79966400
N	25.50997400	81.08882200	21.10818100
C	26.03712000	82.27368500	21.77617100
C	25.05172700	83.41961700	21.45017600
O	24.44922900	83.41889800	20.37013100
C	27.42162900	82.68751700	21.24450200
C	28.64002100	81.82569700	21.59832800
O	28.43401000	80.79863800	22.35908500
O	29.73513800	82.18153800	21.14294900
H	25.12833800	81.29665700	20.18998400
H	26.09255100	82.07827600	22.84631400
H	27.38666100	82.78311000	20.15268500
H	27.64839800	83.69120300	21.62419900
N	24.95309000	84.39887700	22.37128800
C	24.11614700	85.57876600	22.14863100
C	24.71733900	86.60643100	21.16913700
C	23.69916300	87.72909300	20.92261800
C	26.05424700	87.16955900	21.66712400
H	23.94286200	86.04082900	23.12501800
H	24.88927400	86.08159100	20.22068300
H	24.09251000	88.46766700	20.21435800
H	22.76163300	87.33594600	20.51144400
H	23.45976400	88.25529700	21.85595400
H	26.78379000	86.37118500	21.84653500
H	26.48946800	87.85546400	20.93067900
H	25.92965700	87.72833000	22.60365000
H	25.41365700	84.27024400	23.27117300
C	19.47785500	84.90632100	24.17838700
C	20.65957200	85.69757200	24.69200300
C	20.68194800	87.09687200	24.62271900
C	21.76263400	85.04533000	25.26232600
C	21.77085600	87.82495500	25.10958400
C	22.85724900	85.76418600	25.74699400
C	22.86272100	87.16096900	25.67226900
H	18.83747300	84.56437400	25.00271500
H	18.85439100	85.50742900	23.50811700

H	19.83844200	87.62202700	24.17918800
H	21.76627600	83.95869100	25.31764100
H	21.76594000	88.91036500	25.04474200
H	23.71004300	85.23858400	26.16828200
H	23.71647700	87.72291400	26.04248600
C	21.01149900	78.48369900	28.02237200
C	21.40539600	78.43085000	29.49516700
O	21.63537300	77.35927800	30.05894100
C	22.21971100	78.02892800	27.19926500
O	21.92609200	78.08361900	25.80979800
H	20.17257000	77.80097200	27.85113700
H	23.08065100	78.66915700	27.44029500
H	22.48014800	77.00704100	27.50996800
H	22.79493000	78.16471300	25.35568800
N	21.54797400	79.63569200	30.11003100
C	22.21762100	79.76876900	31.39918900
C	21.94388900	81.14961200	32.02162900
C	22.37545400	82.28881000	31.11858600
C	21.43238800	83.01727800	30.37900500
C	23.73603600	82.59028300	30.94967200
C	21.83578900	84.01337300	29.48429200
C	24.14168500	83.58127300	30.05618100
C	23.19206200	84.29303900	29.31703900
H	23.29947400	79.62479400	31.26796400
H	20.87461600	81.23637600	32.24959200
H	22.48292900	81.19552000	32.97599700
H	20.37272600	82.80516800	30.51016300
H	24.48417900	82.04112300	31.51693700
H	21.09255600	84.56597000	28.91536400
H	25.19836700	83.79542200	29.92721700
H	23.51272900	85.05427500	28.61391100
H	21.50007900	80.47191600	29.54146200
C	19.91426700	77.45469000	20.57530900
C	19.21401800	78.69193400	21.16620900
C	20.18606700	79.71816300	21.71274200
C	20.68167200	79.61095000	23.02105700
C	20.63784400	80.78029600	20.91509800
C	21.59379300	80.54294500	23.52050600
C	21.55517800	81.71329100	21.40616900
C	22.03214800	81.59820200	22.71571500
H	20.52380000	76.95084400	21.33469600
H	18.58925500	79.15431400	20.39159400
H	18.53372800	78.37017500	21.96502700
H	20.35670700	78.79769400	23.66590300
H	20.26335100	80.87935400	19.89790700
H	21.95329900	80.42879300	24.53866400
H	21.91354700	82.51853400	20.77102800
H	22.74244100	82.32467300	23.10116500
C	32.65048100	76.26824600	20.03589900
C	31.88899900	77.41216800	20.62025400
N	31.74009400	78.63441300	19.97719600
C	31.20735200	77.56098900	21.79972600
C	30.99438900	79.45246700	20.76336500
N	30.65376300	78.82540800	21.87575300
H	33.70316500	76.52175700	19.85457400
H	32.62932700	75.41971300	20.72526000
H	31.07901500	76.84863900	22.60058400
H	30.72842500	80.47731100	20.53284200
H	32.10611100	78.87511900	19.06721300
C	32.54108500	84.84025600	21.31554100
C	32.07075500	84.05999900	22.53951300
N	31.43435000	84.96803000	23.49931700
C	30.60776600	84.58868300	24.49264900

N	30.35215100	83.30908900	24.73250100
N	30.05555400	85.53323800	25.28254400
H	33.26701900	85.61921700	21.58250100
H	31.69440800	85.31019700	20.80504800
H	31.33836100	83.30701000	22.23457500
H	32.91731900	83.54279700	23.01315100
H	31.74771900	85.92889900	23.51965700
H	30.85007800	82.53157800	24.29592100
H	29.57803100	83.08594000	25.36275400
H	29.84641500	86.45590400	24.90510000
H	29.34902300	85.18357700	25.93268100
Fe	29.49864000	79.73146700	23.61680800
C	26.49632200	80.79697100	25.99575100
C	26.23897900	81.94633800	26.93628900
N	26.34181600	79.47043600	26.65243100
S	26.70918100	83.58060700	26.28433100
O	28.18717400	83.68748900	26.52690100
O	26.35434600	83.58710600	24.83576600
O	25.91840000	84.54566900	27.10343600
H	27.71054200	80.75970800	25.53269800
H	25.16228900	82.01877400	27.14305100
H	26.75744500	81.83230700	27.89331500
H	25.87283300	79.47821900	27.59734300
H	25.78544400	78.83479400	26.03266500
C	31.84416000	79.85543600	24.55886600
O	31.33793300	80.81846300	23.87722500
O	31.16375300	78.81139700	24.74212100
C	33.25414200	79.99429500	25.09696800
C	33.71445800	78.82497200	25.96708300
H	33.67731500	77.88342900	25.41100600
H	34.74227200	78.98434600	26.31087000
O	24.57128100	78.11337500	24.93296900
H	24.67889900	77.14041200	24.83013800
H	24.78545600	78.55780900	24.07120600
H	33.07109700	78.71672200	26.84588300
H	33.91550100	80.12559900	24.22962700
H	33.29851200	80.94071200	25.65024900
O	28.77349000	80.34114200	25.12280600
H	32.22141900	75.93200100	19.08300700
H	23.17850200	79.18069500	21.15791900
H	33.02818900	84.16018300	20.61022900
H	33.78679700	81.29036300	28.77628300
H	27.23758500	83.71032800	35.35295700
H	30.41057000	89.63072200	26.21351400
H	19.18293800	76.73231000	20.19397900
H	20.57691900	77.73554500	19.74851500
H	26.95720100	75.51355700	20.06097200
H	21.85517300	78.96901700	32.05030700
H	29.78766900	74.53328700	28.98114100
H	25.73123200	75.37824800	30.77578300
H	23.14670000	85.24301100	21.76442000
H	19.80206100	84.01365900	23.63157800
H	22.36657200	74.46608800	24.16350300
H	20.70539600	79.48703800	27.70482800
H	25.93868300	80.82257300	25.05931900
H	27.26941500	79.03416500	26.86021000

^{5}TS1$_{HA,C2R,B}$

C	29.69072400	89.86524700	25.98160600
C	28.80191000	88.71590300	25.61825500
N	29.02954500	87.94824700	24.48631000
C	27.68450000	88.25161700	26.27449000
C	28.04978600	87.05654400	24.46710000
N	27.20969500	87.19700100	25.52233300
H	29.67792100	90.63910300	25.20429700
H	29.37228300	90.32459200	26.92290400
H	27.19191600	88.57761500	27.17841100
H	27.92928300	86.26318400	23.74274700
H	26.52364700	86.48901100	25.79269100
C	28.99351800	81.36340800	35.60470400
C	28.70387800	81.09407300	34.14528000
C	28.06580600	79.91582600	33.73617000
C	29.04204000	82.02743800	33.15510100
C	27.76724000	79.66695000	32.39509400
C	28.74527100	81.80448400	31.81150200
C	28.10106800	80.62359700	31.42656500
O	27.85048000	80.42015600	30.09083800
H	29.90505000	81.95875100	35.73059100
H	29.12125900	80.43169800	36.16710100
H	27.80038400	79.16733900	34.48054300
H	29.54273500	82.95078100	33.43944900
H	27.29977000	78.73559200	32.08555900
H	29.00041300	82.53813100	31.05225900
H	27.08168200	79.77178400	29.96708600
C	30.36446700	85.18218200	29.60168400
C	31.58566000	84.33405900	29.99852000
C	30.71496400	86.56660200	29.04170000
C	32.49587600	83.94456400	28.82670400
H	29.76149300	84.63181500	28.86576000
H	32.17507300	84.87867200	30.75098500
H	31.23411900	83.41824500	30.49300000
H	33.30848900	83.28849700	29.16152900
H	32.95451800	84.82226900	28.35754200
H	31.93262100	83.41262700	28.04947300
H	29.80902200	87.13462700	28.80028800
H	31.29499200	87.15175800	29.76741600
H	31.30503900	86.49297300	28.12155600
C	25.25594300	75.25628200	28.71653800
C	26.49305900	74.95436500	27.88109100
O	26.43052200	74.69926700	26.65824900
C	24.93609600	76.76224300	28.76047200
C	25.94666300	77.61261500	29.54913500
O	26.75232500	77.05833300	30.32677600
O	25.85646800	78.88372700	29.36141200
H	24.40993500	74.71557100	28.28438700
H	24.83719900	77.16472500	27.74553300
H	23.96042200	76.89771600	29.24546000
N	27.64674800	75.00855800	28.57700200
C	28.97285300	74.96644100	27.97436400
C	29.91097100	75.98447600	28.63349700
C	29.38624800	77.41372800	28.51612200
O	28.71145900	77.74473800	27.52926800
N	29.72780400	78.27686900	29.48852000
H	28.87463700	75.16999500	26.90689000
H	30.07958200	75.72589700	29.68626900
H	30.88835600	75.93792300	28.13629600
H	30.07401700	77.91716600	30.36724000
H	29.20853000	79.15965900	29.55032100
H	27.54438500	75.46652700	29.48756700
C	21.87603400	75.85648600	24.47109700
C	23.34881300	75.61027000	24.69802900
O	24.21893000	76.27415500	24.09276800
N	23.69796800	74.67724800	25.59827200
H	21.61222700	76.78408500	24.99781300
H	21.24297100	75.04364600	24.83902800
H	22.98993800	74.19268200	26.13090300
H	24.67622000	74.60978400	25.91033000
C	26.22417400	75.74150100	20.62108400
C	25.68606800	77.17282800	20.53219200
O	26.04259400	77.92737100	19.62381200
C	26.52674100	75.20439900	22.03278100
C	27.43260100	76.11090700	22.80879900
N	27.61216300	76.02600000	24.18255800
C	28.19554500	77.18186400	22.41475700
C	28.44277800	77.02078300	24.56638900
N	28.81288600	77.73693000	23.51591800
H	25.49766900	75.07581500	20.13722100
H	26.97436700	74.20563500	21.92946000
H	25.60005800	75.06305900	22.60014900
H	28.31331100	77.61574500	21.43409800
H	28.73233000	77.20653200	25.59086700
H	27.14754600	75.40202100	24.84321600
N	24.77149800	77.54843000	21.46447200
C	24.17402500	78.87280300	21.41065200
C	25.05053400	80.00040300	21.98031600
O	24.88981600	80.41325300	23.14895600
H	24.55791100	76.97001000	22.27351100
H	23.92364400	79.10391500	20.37152100
N	25.94946600	80.54605700	21.15449600
C	26.58472500	81.81890700	21.47404200
C	25.56147500	82.95037100	21.18436000
O	24.71654900	82.83832300	20.29547900
C	27.88444000	82.03903400	20.68044800
C	29.15237700	81.44344200	21.30970200
O	28.96305800	80.43185900	22.09891600
O	30.25038000	81.93303200	21.01907000
H	26.00212200	80.17325100	20.21260800
H	26.82036200	81.81131100	22.53898100
H	27.78473700	81.64245500	19.66040000
H	28.06902800	83.11107000	20.56755200
N	25.70491000	84.04483300	21.96701300
C	24.79016500	85.17912900	21.86426200
C	25.09236900	86.13286000	20.69131500
C	24.00410900	87.21224700	20.61083200
C	26.48958500	86.75601800	20.80006600
H	24.84061600	85.72231100	22.81513000
H	25.04805200	85.52860100	19.77579900
H	24.17405300	87.87843300	19.75688800
H	23.00767100	86.76885400	20.49781300
H	23.99308500	87.83167500	21.51749800
H	27.26669500	85.98479000	20.85038500
H	26.70532100	87.39161100	19.93284800
H	26.57698500	87.38180400	21.69833600
H	26.31328700	83.97615900	22.78254600
C	19.17487400	84.61164300	23.95828300
C	20.26072000	85.30359700	24.75058300
C	20.00757500	86.49781800	25.43791800
C	21.55797400	84.77122100	24.80013700
C	21.01902100	87.14733300	26.14949900
C	22.57502800	85.41415000	25.50814100
C	22.30613900	86.60824500	26.18547500
H	19.19779500	83.52641500	24.10549300
H	18.18072300	84.97610800	24.23919900

H	19.00634600	86.92363800	25.41559300
H	21.76819100	83.84106400	24.27669200
H	20.79954100	88.07337600	26.67566100
H	23.57437100	84.98701000	25.53709900
H	23.09614000	87.11283000	26.73622700
C	21.15379400	78.59132600	28.30446200
C	21.59596700	78.56348700	29.76414300
O	21.91989500	77.51966600	30.33109700
C	22.22316900	79.25900100	27.42501200
O	22.04688600	78.86201500	26.07195900
H	21.03587200	77.55240900	27.98851400
H	22.15788200	80.35520400	27.51715200
H	23.22011900	78.96969700	27.78319600
H	22.93689800	78.91596500	25.65819400
N	21.66507000	79.77459500	30.38502000
C	22.37021800	79.92389400	31.65569900
C	22.42892100	81.40163900	32.07634000
C	23.12317500	82.28106200	31.05375000
C	22.39351100	83.16843200	30.24915000
C	24.50906100	82.18611600	30.85726700
C	23.02638500	83.93663700	29.26727900
C	25.14584500	82.95680600	29.88470800
C	24.40675900	83.83419800	29.08280100
H	23.38434700	79.51468800	31.55841700
H	21.40996000	81.76845400	32.25354000
H	22.95509400	81.44947400	33.03778200
H	21.31940600	83.26634900	30.39938900
H	25.09550500	81.50440500	31.46854400
H	22.44489700	84.61272000	28.64614600
H	26.22084200	82.86639200	29.75270600
H	24.90270000	84.42391000	28.31683100
H	21.56692900	80.60895400	29.81935900
C	17.57621600	79.85962500	19.87493800
C	17.70919200	81.08128100	20.80262700
C	19.06196000	81.15991000	21.48086700
C	19.27592900	80.56179500	22.73139800
C	20.14300800	81.79619300	20.85323800
C	20.53221000	80.59643400	23.34183300
C	21.40386300	81.83662800	21.45242900
C	21.59765600	81.23793800	22.70198500
H	17.70851500	78.92676900	20.43510100
H	17.53677600	81.99442000	20.21839000
H	16.91766200	81.04317500	21.56203700
H	18.44658900	80.06473000	23.23207500
H	19.99367000	82.26695100	19.88310200
H	20.69021000	80.12203200	24.30801100
H	22.23551400	82.32656900	20.95064400
H	22.57818900	81.26229100	23.16807400
C	33.52730400	76.06357800	20.60221100
C	32.67446700	77.20417700	21.05165600
N	32.61367800	78.41897100	20.38022600
C	31.82020000	77.35665600	22.11275500
C	31.74994300	79.23632800	21.03579100
N	31.25172700	78.61497500	22.09009100
H	34.59212600	76.32941300	20.57637500
H	33.41357100	75.22303700	21.29228100
H	31.57844600	76.65147600	22.89349100
H	31.50601300	80.25311800	20.74977100
H	33.11616400	78.65638600	19.53686800
C	33.06176800	84.38854100	21.66450000
C	32.46202900	83.67772400	22.87339500
N	31.87032100	84.66265200	23.78714500
C	31.01447400	84.38356300	24.78396300
N	30.73024700	83.13165100	25.13004000
N	30.47755800	85.40100400	25.49210900
H	33.84411400	85.10038900	21.95734300
H	32.28870800	84.92447700	21.10427800
H	31.68473800	82.98409100	22.53970700
H	33.23087100	83.09701700	23.39980400
H	32.14532500	85.62994300	23.68480200
H	31.13721400	82.31296900	24.67100700
H	29.88689100	82.99303500	25.68761400
H	30.32855200	86.29657300	25.02569900
H	29.70671800	85.12827900	26.10359500
Fe	29.87413200	79.56833200	23.59358900
C	27.29555800	80.99568500	26.62227300
C	26.28054700	82.01505200	26.18544900
N	26.75377600	79.65577300	26.90370500
S	27.06072700	83.61845700	25.74766500
O	28.27930100	83.71875900	26.62358200
O	27.41920800	83.54979600	24.30153400
O	26.01334300	84.63593600	26.05643500
H	27.95140500	81.31494500	27.43144500
H	25.70806100	81.69354600	25.30929100
H	25.58773200	82.23859300	27.00879000
H	26.23885800	79.56504800	27.82633100
H	26.09588100	79.30944800	26.16336900
C	32.02519000	79.69757700	24.93710500
O	31.65733600	80.63434600	24.13550000
O	31.34332000	78.64325100	25.00802100
C	33.28109900	79.89371500	25.76498300
C	33.49486100	78.82865100	26.84096200
H	33.55516400	77.83089500	26.39592800
H	34.42309200	79.01976100	27.39035000
O	24.70602900	78.77968100	25.20841900
H	24.71281800	77.86193100	24.85830600
H	24.70775300	79.36725600	24.40020100
H	32.66694600	78.82516900	27.55738600
H	34.12727100	79.91977800	25.06424100
H	33.22908700	80.89760700	26.20437600
O	28.95751500	80.28838600	24.90733900
H	33.24924900	75.71007600	19.60074800
H	23.25293900	78.86375600	21.99593200
H	33.51339400	83.65225800	20.99336000
H	29.72326000	85.30823300	30.48521600
H	28.17622100	81.92043900	36.08339000
H	30.73274300	89.54262500	26.09855400
H	16.58972200	79.83298000	19.39680500
H	18.33663300	79.88328900	19.08613500
H	27.12462000	75.72473800	20.00081700
H	21.85543500	79.33133700	32.41939800
H	29.40518000	73.96297800	28.07917400
H	25.39733100	74.89640900	29.73947000
H	23.76969900	84.79352700	21.75738900
H	19.29795900	84.78384200	22.88097800
H	21.69141500	76.00889700	23.40461600
H	20.18881300	79.09849600	28.18196400
H	28.16814800	80.74833800	25.61489100
H	27.54501200	78.98716500	27.01858200

^{5}IM1$_{HA,C1S,B}$

C	29.70329900	89.39979000	27.11057800
C	28.69604200	88.45708800	26.52747500
N	28.60502000	88.24419700	25.16019200
C	27.75040100	87.69785200	27.17796400
C	27.60866700	87.38647300	25.00786200
N	27.05315900	87.02699500	26.19397500
H	29.54320100	90.42382300	26.75176400
H	29.64432100	89.41031700	28.20365900
H	27.51317000	87.58204800	28.22495200
H	27.26807700	86.97967400	24.06589400
H	26.41588600	86.23480200	26.31805900
C	27.37147400	82.59562800	35.12675600
C	27.36205700	82.11531300	33.69290900
C	27.10216400	80.77534300	33.37592100
C	27.59169200	82.99988700	32.63000000
C	27.06307400	80.32216600	32.05609200
C	27.54767400	82.57212500	31.30374300
C	27.27706400	81.22959900	31.00888000
O	27.25732500	80.84549300	29.69457400
H	26.38745900	82.97633500	35.43443100
H	28.09060500	83.40968400	35.27400300
H	26.93613700	80.06101200	34.18036400
H	27.80408100	84.04624000	32.84136100
H	26.90122200	79.27281000	31.82541600
H	27.72361200	83.26570200	30.48649600
H	26.71287700	79.99699900	29.55572500
C	33.00123000	82.72872100	29.55502800
C	31.73461900	82.78820100	28.68441000
C	33.86274100	83.99657800	29.50805900
C	30.70154100	83.82909800	29.13335900
H	32.71179700	82.52389200	30.59592800
H	32.02326500	82.99044600	27.64160500
H	31.26024600	81.79750900	28.68166500
H	29.80611200	83.78642100	28.50383800
H	31.09884300	84.84955300	29.07796200
H	30.38921100	83.64701400	30.16939900
H	34.77138200	83.88077300	30.11054700
H	34.17207900	84.22494400	28.47973000
H	33.32303300	84.86946900	29.89170300
C	25.95211700	75.07852200	29.18502300
C	27.18369000	74.83656700	28.32447900
O	27.13688100	74.24364300	27.22904000
C	25.33431000	76.45908600	28.89082400
C	26.15194400	77.64852400	29.42086000
O	27.04432700	77.45427000	30.27379300
O	25.81192000	78.80028600	28.95573900
H	25.22338700	74.29135000	28.97452400
H	25.16999600	76.58564600	27.81390900
H	24.34805400	76.51855500	29.36866300
N	28.32242500	75.36679100	28.82152200
C	29.52422100	75.53840300	28.01358000
C	30.24183800	76.84402500	28.36735700
C	29.36087700	78.07905200	28.15984800
O	28.51550200	78.12113300	27.26418100
N	29.60709300	79.13023800	28.98342900
H	29.23418200	75.53008500	26.96004200
H	30.61494500	76.80749200	29.39884700
H	31.11896800	76.94897900	27.71674400
H	30.08610400	78.95364600	29.85587800
H	28.89581600	79.86357000	29.03265800
H	28.16751500	76.00666300	29.60527600
C	22.49342700	74.51005500	25.27523000
C	23.99139100	74.50130900	25.47365400
O	24.71736400	75.31266900	24.85529300
N	24.52100100	73.60621100	26.31782500
H	22.10934900	75.49185100	25.58214500
H	21.97898300	73.72552600	25.83721900
H	23.91864400	72.99907800	26.85445700
H	25.50700600	73.71578000	26.61395700
C	26.10549100	75.46015900	20.40257000
C	25.64517300	76.87914200	20.06164400
O	26.12985900	77.49592700	19.12033100
C	26.26223000	75.12394800	21.89466100
C	27.14896600	76.08057800	22.63533700
N	27.24670000	76.06815900	24.01772500
C	27.95421600	77.11422300	22.22757300
C	28.05838500	77.07250500	24.40323000
N	28.50779500	77.72469700	23.33663300
H	25.39383700	74.75459200	19.95438500
H	26.65829200	74.10170200	21.97349600
H	25.28572400	75.08651400	22.39619000
H	28.15257100	77.47855300	21.23134600
H	28.28817800	77.31449600	25.43492400
H	26.64291800	75.52030000	24.63443900
N	24.62359900	77.39634700	20.82063500
C	24.24278300	78.78850400	20.66074700
C	25.12809000	79.74190700	21.47822800
O	25.33858800	79.53393500	22.68179000
H	24.40747500	76.96077000	21.70621900
H	24.28376700	79.02044000	19.59531800
N	25.62193900	80.79500000	20.79808100
C	26.20703200	81.97648900	21.41984600
C	25.20426600	83.13232700	21.21705400
O	24.46701900	83.14747500	20.22620400
C	27.54655000	82.38234600	20.77136900
C	28.80603600	81.61685200	21.19444100
O	28.61990400	80.59159300	21.96878000
O	29.89958200	82.01942000	20.78514200
H	25.23905700	80.97261500	19.87563700
H	26.35029300	81.76146200	22.47766600
H	27.46673300	82.32452600	19.67867600
H	27.74710200	83.43593900	20.99656400
N	25.24150800	84.10792200	22.15105000
C	24.35109600	85.26784100	22.07537800
C	24.74565900	86.30586700	21.00592000
C	23.64263100	87.36812800	20.89410500
C	26.10660500	86.94911600	21.29869300
H	24.33582600	85.73065100	23.06676400
H	24.80805800	85.76949900	20.05100600
H	23.89352900	88.11373300	20.13051800
H	22.68190600	86.91722500	20.61921100
H	23.50302100	87.89687900	21.84587900
H	26.89020800	86.19257300	21.42617700
H	26.41396700	87.61098100	20.48044600
H	26.06390700	87.55738100	22.21196900
H	25.78457400	83.93929500	22.99727900
C	19.25711600	86.52894800	23.93025900
C	20.61800400	86.93845800	24.44619100
C	20.97214600	88.29125800	24.54657800
C	21.55230200	85.97658300	24.85424500
C	22.21900500	88.67371500	25.04510800
C	22.80258900	86.35161600	25.35517500
C	23.13890000	87.70480900	25.45262800
H	19.27634000	85.51851900	23.50865600
H	18.50844500	86.53231500	24.73422200

H	20.26346900	89.05302800	24.22764200
H	21.30247800	84.92116200	24.76760300
H	22.47279400	89.72873500	25.11231500
H	23.51901800	85.59021100	25.65428400
H	24.11248500	87.99950200	25.83540800
C	21.24842100	77.84163700	28.28917000
C	21.87754800	78.05400700	29.66484900
O	22.33025300	77.12015200	30.32563300
C	22.20348100	78.34597300	27.19768500
O	21.82815800	77.80143800	25.94199500
H	21.09449000	76.76725100	28.16513400
H	22.17752500	79.44832300	27.16207700
H	23.23276800	78.06135300	27.45615300
H	22.56656200	78.01158800	25.32744700
N	21.95973200	79.35290400	30.07069400
C	22.86329000	79.77254500	31.13866200
C	22.47833600	81.15902800	31.68520500
C	22.36263000	82.21562600	30.60238800
C	21.10454600	82.66759300	30.17857700
C	23.50476900	82.72677200	29.96402300
C	20.98548900	83.60554100	29.14905500
C	23.38978400	83.66067700	28.93280800
C	22.12792200	84.10372900	28.52199100
H	23.89553000	79.78377700	30.76233400
H	21.52662500	81.08230500	32.22452300
H	23.24420300	81.44736800	32.41572500
H	20.21033700	82.28644700	30.66777700
H	24.49052500	82.39380700	30.28165900
H	20.00063200	83.94877700	28.84213000
H	24.28077900	84.04414100	28.44292200
H	22.04011000	84.83785300	27.72608800
H	21.67559900	80.07465300	29.41989000
C	19.64394600	77.72665500	23.09511100
C	19.08942200	79.15764000	22.96669800
C	20.15999400	80.21809200	23.12271200
C	20.56511400	80.64290600	24.39624900
C	20.79218600	80.77907300	22.00362900
C	21.57138200	81.59861800	24.54797100
C	21.80654500	81.73023800	22.14605600
C	22.19934900	82.14110200	23.42327400
H	20.12948300	77.58466300	24.06627900
H	18.59681900	79.27357300	21.99279000
H	18.31317500	79.30696400	23.72838000
H	20.08978200	80.21463200	25.27497000
H	20.47598500	80.47526000	21.00698100
H	21.85996000	81.92685200	25.54414500
H	22.28618200	82.15720100	21.26907200
H	22.98744700	82.87946000	23.54014100
C	33.22107000	76.17360700	20.41846400
C	32.34184100	77.29609800	20.86250300
N	32.26401000	78.51176600	20.19390100
C	31.47331600	77.42808200	21.91455100
C	31.37714100	79.30982800	20.84183500
N	30.88014300	78.67537600	21.88897100
H	34.28088200	76.45960300	20.40525200
H	33.11557400	75.32850600	21.10420400
H	31.23469100	76.71396200	22.68833000
H	31.11718400	80.32362500	20.55830700
H	32.76728600	78.75971500	19.35403900
C	32.66998400	84.71722100	21.40064900
C	32.07625800	83.95890200	22.58349000
N	31.46903000	84.90343800	23.52890900
C	30.64124200	84.56346500	24.53483300
N	30.34831100	83.29468800	24.79169100
N	30.12755700	85.53153300	25.32159500
H	33.44978200	85.42109200	21.71853600
H	31.89434500	85.27217300	20.86290000
H	31.30704200	83.26742400	22.22666700
H	32.85145200	83.36690100	23.08880100
H	31.80212800	85.85776900	23.52107300
H	30.78762600	82.49414300	24.32780900
H	29.57421900	83.11391600	25.43413300
H	29.99507700	86.47142500	24.95495400
H	29.38766400	85.22806000	25.95560700
Fe	29.47624300	79.60328500	23.40787900
C	25.42553700	80.91186700	26.25539800
C	26.25135100	81.99723500	26.84990300
N	26.15325000	79.59457500	26.27426700
S	26.75157600	83.40132200	25.91973800
O	28.09262900	83.77299200	26.47924400
O	26.82026500	82.95586500	24.48784000
O	25.72596100	84.46881300	26.13638600
H	25.16619200	81.12245800	25.21626800
H	26.59032600	81.94839400	27.88219600
H	27.93544400	81.16167300	24.40291500
H	26.23314300	79.22946300	27.25161500
H	25.58998400	78.90941500	25.72356300
C	31.63692900	79.92753700	24.68867000
O	31.24090500	80.80961600	23.84389200
O	30.96656500	78.86728300	24.82525900
C	32.90716900	80.17774100	25.47610700
C	33.16410200	79.17511800	26.60068900
H	33.21895100	78.15442500	26.20986100
H	34.10835000	79.40197400	27.10764700
O	24.32196400	78.06382800	24.72185800
H	24.48042200	77.09370000	24.67151400
H	24.59855800	78.47379700	23.86469400
H	32.35968100	79.21123900	27.34179400
H	33.73408600	80.17754200	24.75199700
H	32.85297100	81.20157100	25.86609300
O	28.41039000	80.37473000	24.73852000
H	32.96037700	75.81895200	19.41277200
H	23.21383100	78.92233900	21.01318800
H	33.12413600	84.00905400	20.70160700
H	33.61183800	81.87222900	29.23541300
H	27.63472200	81.78739000	35.81827200
H	30.72523500	89.11647500	26.82939100
H	18.84085200	76.98654800	22.99433600
H	20.38621500	77.52671200	22.31224800
H	27.05475300	75.32715900	19.87661600
H	22.81394400	79.02032500	31.92938700
H	30.21261900	74.69724900	28.16755500
H	26.21145700	75.03035600	30.24649500
H	23.33673100	84.91022700	21.86469000
H	18.89903700	87.21408000	23.15365800
H	22.27199000	74.38952900	24.20978400
H	20.27520300	78.34067600	28.20157300
H	24.50655000	80.76340300	26.83365200
H	27.09969200	79.70359900	25.84039700

$^{5}IM1_{HA,C1R,B}$

C	29.38070800	87.32236100	30.13277600
C	28.11682500	86.76138400	29.55588500
N	26.92366000	86.80060700	30.25370100
C	27.92407000	86.15685600	28.33414700
C	26.03554400	86.23299600	29.45843100
N	26.58639200	85.82565000	28.28173000
H	29.63858400	86.83102900	31.07921300
H	30.22110300	87.18889400	29.44276100
H	28.59276700	85.92548100	27.51977200
H	24.98850700	86.08555800	29.68833400
H	26.12668900	85.32313200	27.52077100
C	27.04861200	83.81383200	34.80460400
C	27.02661500	83.06174800	33.49275700
C	26.88966000	81.66787200	33.45595800
C	27.16127600	83.74045200	32.27212100
C	26.88879800	80.96175300	32.25054800
C	27.16079700	83.05368000	31.05864700
C	27.01629800	81.66242800	31.04588400
O	27.00767900	81.01099100	29.83169700
H	26.49458100	84.75700700	34.73528000
H	28.07378200	84.06699700	35.10824600
H	26.78443200	81.11672900	34.38854400
H	27.24367700	84.82490000	32.25236600
H	26.80059000	79.87861400	32.23249600
H	27.25394100	83.59143700	30.11988700
H	26.45352700	80.16402000	29.88552700
C	32.90906200	82.40707000	29.02906400
C	31.70673200	82.40971700	28.06880500
C	33.76435500	83.67885300	28.97785200
C	30.63954800	83.46295500	28.38930700
H	32.54721000	82.25340000	30.05590800
H	32.06711200	82.55675600	27.03931000
H	31.23784100	81.41618400	28.09232800
H	29.80072200	83.40659300	27.68735600
H	31.04344500	84.48047100	28.33461900
H	30.23459100	83.32422100	29.39938400
H	34.63004200	83.60024000	29.64590100
H	34.14222100	83.85880200	27.96290500
H	33.19490100	84.56415400	29.28117100
C	26.14138700	75.22413700	29.39089000
C	27.22657900	75.20374300	28.32089700
O	27.08507700	74.64031100	27.22102200
C	25.31110500	76.52089200	29.35252700
C	26.06227300	77.77474400	29.82625500
O	27.11811300	77.66404600	30.48170200
O	25.50734800	78.89710900	29.51330300
H	25.49305600	74.36001700	29.21993800
H	24.91867100	76.69596500	28.34324500
H	24.43497100	76.42334800	30.00719600
N	28.35481100	75.87921600	28.63977800
C	29.40560400	76.13013000	27.66072600
C	30.15782000	77.43458100	27.95605100
C	29.22979200	78.64174400	27.98198900
O	28.49407800	78.88373600	27.00032800
N	29.26915300	79.42407300	29.06679800
H	28.95090700	76.16928800	26.66771900
H	30.71859100	77.35334500	28.89471700
H	30.88075700	77.60007500	27.14792300
H	29.71443900	79.08006100	29.90631700
H	28.53787700	80.13189600	29.22081000
H	28.28557900	76.45261100	29.48404200
C	22.35865400	74.95797500	25.40471900
C	23.86285700	75.01993800	25.52521900
O	24.50997200	75.92327100	24.94565000
N	24.47196500	74.09507900	26.27830500
H	21.93600500	75.78989000	25.98458000
H	21.93918100	74.01614700	25.77003500
H	23.91927200	73.40708500	26.76902400
H	25.45931400	74.21575600	26.56007500
C	26.62065600	75.24076800	20.17127200
C	26.66982600	76.52527900	19.33711900
O	27.57922400	76.72182200	18.53639100
C	26.25051200	75.38712800	21.65762700
C	27.13646200	76.32864200	22.41830100
N	26.97288300	76.54830700	23.77496000
C	28.16335200	77.15815600	22.04107000
C	27.86005900	77.48571900	24.16291600
N	28.60524300	77.87573400	23.13626200
H	25.90348400	74.55721700	19.69870300
H	26.28921700	74.38927200	22.11584200
H	25.20362200	75.70701100	21.76713100
H	28.59761000	77.29850100	21.06364300
H	27.95600100	77.85024200	25.17524900
H	26.23212300	76.14738400	24.36235100
N	25.61838700	77.39063400	19.49982700
C	25.70969700	78.74961400	18.99827400
C	25.82175300	79.72730500	20.17984600
O	25.37501900	79.41597600	21.28518400
H	25.09594800	77.33137300	20.36703600
H	26.57600000	78.80420200	18.33593000
N	26.44024100	80.89851400	19.89079600
C	26.46047100	82.03111000	20.80725000
C	25.46671900	83.08914800	20.27274200
O	25.23074800	83.15078300	19.06308400
C	27.85693500	82.67133900	20.87432600
C	29.02381200	81.78277800	21.32088200
O	28.71107400	80.78204500	22.07348200
O	30.17065100	82.11674100	20.97873500
H	26.49050500	81.16567100	18.91271700
H	26.17676300	81.66151000	21.79214800
H	28.12280600	83.10500900	19.90394500
H	27.81081500	83.49950800	21.59320900
N	24.94322900	83.92238900	21.19799200
C	24.07490000	85.03759500	20.83692700
C	24.82712600	86.34670800	20.52075500
C	23.83263100	87.40976500	20.03260000
C	25.62701600	86.85374100	21.72937700
H	23.37931100	85.20143000	21.66822200
H	25.52582600	86.12228000	19.70247000
H	24.34912200	88.33999800	19.76757900
H	23.28328900	87.06807300	19.14688000
H	23.09998400	87.64879500	20.81442900
H	26.32373800	86.09314300	22.10005400
H	26.20496200	87.74876800	21.46644100
H	24.95744000	87.11846100	22.55747500
H	25.23237700	83.82400400	22.16866700
C	19.62824200	85.92820500	22.80867700
C	20.76518700	86.54036100	23.59580800
C	20.85776700	87.92943200	23.76325200
C	21.76584000	85.73596400	24.15950900
C	21.91887700	88.49805200	24.47028000
C	22.83382500	86.29763800	24.86640700
C	22.90982900	87.68413700	25.02441500
H	19.45793000	84.88578800	23.09775000
H	18.69300100	86.47976700	22.95786600

H	20.08759800	88.57101500	23.33939500
H	21.70871700	84.65555400	24.04528400
H	21.96782000	89.57727600	24.59329900
H	23.60306500	85.65464800	25.28688400
H	23.73310600	88.12499900	25.58041400
C	21.32477300	77.80127600	29.20943100
C	21.96265800	78.20129400	30.53967000
O	22.60378400	77.41169500	31.22946800
C	22.06329500	78.47662300	28.04837900
O	21.67294600	77.87877500	26.82079800
H	21.41495500	76.71652900	29.11527900
H	21.84011400	79.55566000	28.03375300
H	23.14518700	78.37598300	28.21099000
H	22.29084800	78.22641400	26.14148400
N	21.80578000	79.51471000	30.87400600
C	22.65540800	80.16256800	31.86959800
C	22.19043100	81.60862600	32.12403100
C	22.08013400	82.43818000	30.85721000
C	20.82508700	82.76765200	30.32447700
C	23.22768100	82.85896600	30.16532700
C	20.71332000	83.49806100	29.13693600
C	23.12028800	83.58085700	28.97533000
C	21.86147900	83.90551700	28.45677100
H	23.69928100	80.14904300	31.52857800
H	21.21904000	81.59020500	32.63294300
H	22.90832900	82.06794900	32.81430700
H	19.92513500	82.46061000	30.85410200
H	24.21214600	82.62590800	30.56421300
H	19.72955700	83.75431800	28.75156300
H	24.01699000	83.88825000	28.44492000
H	21.78256700	84.48219200	27.53872300
H	21.39309700	80.12840000	30.18225800
C	19.02826200	78.38960100	24.61553500
C	19.06914200	79.89905300	24.31906000
C	20.47222900	80.44405800	24.13477900
C	21.02264900	81.35574000	25.04678700
C	21.25871300	80.04841900	23.04149000
C	22.30899400	81.87224700	24.86648000
C	22.54882600	80.55362400	22.85448600
C	23.07589100	81.47727600	23.76647300
H	19.61721700	78.15090900	25.50682200
H	18.47803200	80.10616500	23.41586100
H	18.57931600	80.44178200	25.13680500
H	20.43528600	81.67345400	25.90569800
H	20.85011200	79.34588800	22.31744800
H	22.71003300	82.59092800	25.57673400
H	23.14739800	80.23767300	22.00451300
H	24.06591200	81.89600000	23.61413400
C	32.95768500	76.20323100	20.02588100
C	32.19695000	77.38183600	20.53661600
N	32.06384400	78.56798500	19.82405200
C	31.49549400	77.59339500	21.69228300
C	31.30670300	79.43173800	20.53823600
N	30.94285600	78.86341900	21.67833500
H	34.01006900	76.44602500	19.83117200
H	32.93388400	75.39918700	20.76618400
H	31.34219800	76.93003300	22.52874100
H	31.05670800	80.44761900	20.25969300
H	32.44868900	78.75545800	18.90905100
C	32.83778200	84.28369400	20.94451500
C	32.40368100	83.82971100	22.33495300
N	31.39432400	84.75024500	22.86872500
C	30.40919200	84.44171200	23.72992200

N	30.29745200	83.23779300	24.27604200
N	29.53383300	85.41067300	24.08786200
H	33.23205600	85.30858100	20.95068500
H	31.99220500	84.22706900	20.25311800
H	31.95492700	82.83963700	22.26034800
H	33.26052900	83.77839900	23.02049600
H	31.52318500	85.73405100	22.67469400
H	30.93058600	82.46600700	24.06438400
H	29.43715700	83.01049500	24.77343800
H	29.36437600	86.16146000	23.43357500
H	28.73143200	85.10113800	24.65172700
Fe	29.83302500	79.64906000	23.19427500
C	25.70770300	81.28497000	26.88065900
C	26.33853300	81.78141900	25.62704200
N	25.78435500	79.79122500	26.95308900
S	26.38215200	83.50588900	25.29987100
O	27.78249600	83.93189800	25.67054200
O	26.13051800	83.66220100	23.84142000
O	25.35184700	84.13068300	26.18182300
H	24.65561200	81.57669500	26.95679200
H	29.19234200	79.91354200	25.38544700
H	27.04516600	81.18646500	25.04714100
H	25.49447500	79.43761300	27.90579200
H	25.18462300	79.33936600	26.22900600
C	32.06498300	79.83066600	24.47323000
O	31.87174700	80.84595800	23.75435700
O	31.19514400	78.88798000	24.51378300
C	33.32323300	79.69621100	25.31455700
C	33.91973100	78.28407400	25.30993000
H	34.20378500	77.98086500	24.29578400
H	34.81585700	78.24240300	25.93819200
O	23.87454300	78.57801000	25.21353700
H	24.06599200	77.64898800	24.93619500
H	23.65353800	79.09064200	24.41292900
H	33.19915000	77.55227000	25.68610500
H	34.04564300	80.43458400	24.95421400
H	33.05762100	79.98818300	26.34037900
O	29.00305000	80.43080100	24.57790300
H	32.52622300	75.81187300	19.09594600
H	24.81443100	79.00620800	18.41507400
H	33.63285100	83.62831200	20.57439700
H	33.54703900	81.54149700	28.79820400
H	26.60685200	83.22186900	35.61379800
H	29.28157200	88.39442500	30.34472000
H	17.99603600	78.05453500	24.77206200
H	19.43982900	77.81341500	23.77776800
H	27.60963100	74.78600100	20.07002300
H	22.61156200	79.58173200	32.79527100
H	30.12496400	75.30104200	27.65077200
H	26.59077900	75.12427500	30.38354000
H	23.49693200	84.72882000	19.96103300
H	19.83941300	85.93588100	21.73077000
H	22.07341100	75.09930200	24.35845400
H	20.25900800	78.06071900	29.17757000
H	26.21302600	81.66401700	27.77957800
H	26.77313900	79.48786200	26.85659300

^{5}IM1$_{HA,C2S,B}$

C	30.62583400	88.66487600	27.68362100
C	29.33808100	88.05428700	27.22192600
N	28.84879000	88.26996200	25.94352300
C	28.46800500	87.24831500	27.92102100
C	27.69698900	87.61790900	25.89765100
N	27.41998000	86.98107500	27.06557200
H	30.56854000	89.76036900	27.68643000
H	30.87532900	88.33640300	28.69787900
H	28.49820000	86.85435800	28.92587700
H	27.04050600	87.56065600	25.03989200
H	26.68352700	86.27870200	27.19744700
C	28.08954100	82.99621200	34.98711100
C	27.89750100	82.48022100	33.57869200
C	27.71186700	81.11337600	33.32659900
C	27.91902100	83.34528200	32.47713100
C	27.56368100	80.61599000	32.03193300
C	27.76356500	82.87121300	31.17369400
C	27.58683800	81.50149900	30.94367500
O	27.45672300	81.06435900	29.65267700
H	27.86698500	84.06678800	35.05481900
H	29.12297700	82.85687100	35.33404200
H	27.68711300	80.41637900	34.16260600
H	28.05008200	84.41336400	32.64006000
H	27.44116200	79.55177000	31.84695700
H	27.76649400	83.54935900	30.32543000
H	26.90112700	80.21370000	29.61256800
C	33.03792400	83.33393200	29.17139300
C	31.81259500	83.01549000	28.29776500
C	33.58040700	84.75860800	29.00524600
C	30.55764500	83.82633800	28.64111000
H	32.77845400	83.16398400	30.22618800
H	32.07451400	83.17998400	27.24090500
H	31.57912700	81.94602700	28.39195500
H	29.70148800	83.50503200	28.03814400
H	30.70169800	84.89795500	28.46143800
H	30.28666400	83.69359900	29.69576900
H	34.47440200	84.91602900	29.61991700
H	33.85613000	84.95612800	27.96099500
H	32.84173000	85.51241300	29.29971800
C	25.94660100	75.30254000	29.42346400
C	27.08746300	75.01181900	28.45927100
O	26.91412900	74.44119000	27.36513100
C	25.36431100	76.70609200	29.17471200
C	26.27526300	77.86217100	29.61793200
O	27.22838400	77.64071200	30.39368400
O	25.94194100	79.01976800	29.16166700
H	25.17151500	74.54460900	29.27824900
H	25.11827600	76.83468100	28.11488100
H	24.41848000	76.81094900	29.72164600
N	28.29194200	75.47036000	28.86542300
C	29.43458800	75.57862800	27.96594700
C	30.20291000	76.88370700	28.19731400
C	29.33151400	78.12726200	28.00862800
O	28.42876300	78.15357800	27.16914700
N	29.65553900	79.19901200	28.77728900
H	29.06379100	75.52356800	26.94022500
H	30.66475100	76.88394500	29.19287600
H	31.02031500	76.94385300	27.46764200
H	30.18630000	79.03707100	29.62214900
H	28.97909200	79.96249300	28.84905900
H	28.24065200	76.10694100	29.66438500
C	22.27219500	74.62554300	25.26905900
C	23.76514400	74.60998700	25.50659700
O	24.53365300	75.23361300	24.73657300
N	24.23439600	73.92968400	26.55826500
H	21.90696500	75.64757700	25.44024400
H	21.72558600	73.93542600	25.91794900
H	23.59540500	73.47158400	27.19197600
H	25.22690500	74.02618700	26.83801800
C	25.80450200	75.46282500	20.41087700
C	25.36540100	76.92560500	20.30070900
O	25.71510000	77.62202900	19.35472600
C	26.13313200	74.93823100	21.82057000
C	27.07550700	75.83415100	22.56858700
N	27.11957000	75.87631600	23.95334800
C	27.95240100	76.80417600	22.15336800
C	27.96288800	76.85836200	24.33088100
N	28.49038900	77.43578600	23.25710600
H	25.01682200	74.83675900	19.97148100
H	26.54979900	73.92605800	21.72037500
H	25.22090400	74.81748700	22.41781800
H	28.20062400	77.12348600	21.15263200
H	28.14863700	77.15237100	25.35767300
H	26.47267600	75.37713700	24.56721600
N	24.52045900	77.40381400	21.26934500
C	24.10528900	78.79419900	21.24136600
C	25.13376600	79.76092800	21.84804100
O	25.55582600	79.58760200	23.01085000
H	24.35283200	76.86684600	22.10850400
H	23.88934900	79.06014200	20.20514800
N	25.48324000	80.79597600	21.07757100
C	26.17050200	82.00834600	21.51995400
C	25.21977400	83.16940600	21.14824600
O	24.68529500	83.16213800	20.03427700
C	27.49771600	82.24905900	20.77419200
C	28.70619600	81.36358600	21.10093900
O	28.47575800	80.26733300	21.76071300
O	29.81488800	81.73644900	20.70298400
H	25.03241300	80.90283600	20.17413600
H	26.32930700	81.94228700	22.59724400
H	27.31308800	82.18378400	19.69479100
H	27.81721300	83.28098000	20.95805200
N	25.06138200	84.14055000	22.07026700
C	24.20142500	85.29733400	21.82073100
C	24.96069600	86.54154900	21.32036600
C	23.95793600	87.62883000	20.90817600
C	25.94426900	87.06576600	22.37476800
H	23.67483500	85.52923300	22.75287100
H	25.52872200	86.23676300	20.42990800
H	24.47681900	88.52139600	20.53931900
H	23.29108800	87.27558700	20.11244400
H	23.33474900	87.93238900	21.75918600
H	26.65521300	86.28949400	22.68112000
H	26.51948300	87.91684000	21.99132300
H	25.40086200	87.40178700	23.26794600
H	25.40709700	83.99263400	23.01859100
C	19.76799000	85.27849000	23.79457000
C	20.84543000	86.17101400	24.36803700
C	20.77955400	87.56456900	24.23218400
C	21.93080700	85.62489000	25.06899600
C	21.76186000	88.38905000	24.78640100
C	22.91973500	86.44131300	25.62437100
C	22.83372500	87.83008300	25.48599100
H	19.02454200	85.01218800	24.55823300
H	19.23122100	85.77218000	22.97723100

H	19.94926800	88.00828000	23.68644200
H	22.00910700	84.54502700	25.17497100
H	21.68872800	89.46768400	24.67033500
H	23.75670800	85.98758000	26.14861100
H	23.59861300	88.47042200	25.91818000
C	21.22832000	78.35242400	28.27756000
C	21.79806700	78.39906700	29.69255900
O	22.11025500	77.36997700	30.29281100
C	22.35866300	77.92844000	27.33489600
O	21.91131400	77.94118200	25.98488500
H	20.41791300	77.61732200	28.23974700
H	23.21123700	78.60978000	27.46694800
H	22.69702000	76.92540500	27.63079900
H	22.71988000	78.04189800	25.43569400
N	21.99938300	79.64810900	30.19399800
C	22.82150800	79.90611100	31.37044800
C	22.40480200	81.21556600	32.06785500
C	22.40670300	82.40727200	31.12957000
C	21.20544100	82.90305400	30.60112100
C	23.61125100	82.99695300	30.71422200
C	21.20409400	83.95035100	29.67532000
C	23.61397700	84.04025100	29.78647900
C	22.40930300	84.51730300	29.25952100
H	23.87956500	79.95186400	31.07594900
H	21.40564600	81.08765100	32.50104200
H	23.10092100	81.38446900	32.89872700
H	20.26246000	82.46409500	30.92106200
H	24.55536300	82.63411300	31.11519200
H	20.26161500	84.31947200	29.27868400
H	24.55390400	84.47217400	29.45616800
H	22.41438900	85.32119500	28.52873200
H	21.82010600	80.43998600	29.59038800
C	19.84291700	77.64125300	21.02873600
C	19.23422500	78.91871100	21.63520600
C	20.28149000	79.88525500	22.15051700
C	20.78971900	79.76619000	23.45286900
C	20.79394600	80.89856700	21.32710600
C	21.77263100	80.64212900	23.92000500
C	21.78232200	81.77309900	21.78670600
C	22.27239900	81.65106800	23.09161700
H	20.44030000	77.10165600	21.77294700
H	18.61688900	79.41665000	20.87680300
H	18.55906000	78.63930900	22.45387400
H	20.42224300	78.98796700	24.11731800
H	20.41184800	81.00624700	20.31367200
H	22.14072600	80.52496200	24.93520800
H	22.17887500	82.53722600	21.12375000
H	23.03743600	82.33170700	23.45670900
C	33.33908000	76.01541700	20.52155600
C	32.40194900	77.11132200	20.91009200
N	32.30371100	78.31068700	20.21462000
C	31.48951900	77.22921900	21.92596100
C	31.36418900	79.08665300	20.81211100
N	30.85221800	78.45279000	21.85297500
H	34.38730000	76.34058600	20.54581700
H	33.23674600	75.17800700	21.21711700
H	31.24690000	76.52021400	22.70321400
H	31.07222100	80.08389600	20.50051000
H	32.83011400	78.56274900	19.39024100
C	32.46375500	84.83894900	21.45990300
C	31.70930700	83.95102400	22.44528100
N	31.06241700	84.77763800	23.47089700
C	30.06115700	84.36965800	24.27667300
N	29.59358400	83.12216900	24.21009800
N	29.51946400	85.23177100	25.14554300
H	33.24899100	85.42240800	21.95781300
H	31.78342900	85.53279800	20.95506400
H	30.94158600	83.37430800	21.92093000
H	32.39253800	83.23047800	22.91447900
H	31.49528500	85.66123500	23.70259300
H	30.17723300	82.34974200	23.88319600
H	28.81333200	82.88299800	24.82706800
H	29.61458900	86.23808800	25.05478700
H	28.76625200	84.87882100	25.73966100
Fe	29.36033200	79.37239700	23.26890200
C	25.31910900	80.90452800	26.27407500
C	25.44430700	82.09317100	27.13408200
N	26.13955400	79.72409500	26.47021600
S	26.10183500	83.56859000	26.22898800
O	27.59852800	83.42571500	26.22156500
O	25.50186800	83.50712000	24.86540600
O	25.64894300	84.74070000	27.04030300
H	27.28255200	80.16363900	23.91017500
H	24.46171400	82.43709500	27.47979400
H	26.09101600	81.93856900	28.00354900
H	26.22343800	79.45169600	27.48346700
H	25.66711300	78.92892500	25.98748100
C	31.43835900	79.92964200	24.59078300
O	30.98500100	80.72212500	23.68659400
O	30.83701000	78.84028100	24.79910800
C	32.69071100	80.31557900	25.34824200
C	33.08438100	79.34875700	26.46434000
H	33.24835700	78.34055000	26.07121300
H	34.00721100	79.68323000	26.95047600
O	24.49571600	78.01346200	24.91971500
H	24.61298800	77.04539700	24.80306000
H	24.82315800	78.46855600	24.10072900
H	32.29746100	79.29316900	27.22246800
H	33.49349400	80.40977900	24.60385500
H	32.53171600	81.32644900	25.74363600
O	28.08468000	80.05374500	24.46392600
H	33.13267200	75.63540100	19.51254600
H	23.18577700	78.90342600	21.82525700
H	32.94375500	84.21780000	20.69785600
H	33.83890200	82.61883500	28.93601400
H	27.43981400	82.47450700	35.69964100
H	31.45827900	88.39034000	27.02377400
H	19.05926500	76.96467400	20.66799800
H	20.49908800	77.88145000	20.18416300
H	26.67363300	75.37391800	19.75328800
H	22.70424600	79.05346900	32.04342600
H	30.11750600	74.73138800	28.11139700
H	26.29398100	75.24141000	30.45885300
H	23.46240800	84.99852200	21.07212500
H	20.18447900	84.34064300	23.41076600
H	22.07372700	74.37093200	24.22362000
H	20.82828600	79.32045000	27.95431700
H	24.79149600	80.93824600	25.33046300
H	27.09142000	79.80607800	26.02396100

^{5}IM1$_{HA,C2R,B}$

C	30.28644400	89.23516100	27.27396500
C	29.18935300	88.43183800	26.64589800
N	29.02599800	88.37373700	25.27001800
C	28.21985400	87.67178500	27.25865800
C	27.96396000	87.60716300	25.07739500
N	27.43518800	87.15808200	26.24609800
H	30.18881600	90.30003300	27.03005000
H	30.27183200	89.13392700	28.36397400
H	28.01835000	87.45961500	28.29810500
H	27.54897900	87.33715600	24.11587700
H	26.74489200	86.40448100	26.33853600
C	28.58994600	82.09405300	35.50206600
C	28.25496300	81.70217800	34.08065400
C	27.53074200	80.53629400	33.79901100
C	28.63938100	82.50407100	32.99684000
C	27.19051500	80.17454800	32.49390300
C	28.30348500	82.16752400	31.68602000
C	27.56954000	81.00247500	31.42764200
O	27.28308100	80.69603100	30.12469400
H	27.81565700	82.74045700	35.93816500
H	29.53587100	82.64493600	35.55504500
H	27.23159800	79.88473800	34.61801800
H	29.21035900	83.41228100	33.17953900
H	26.66765400	79.24503200	32.28591800
H	28.60514300	82.79459400	30.85165100
H	26.53010000	80.02478400	30.05144400
C	31.77362700	84.47452100	29.80707500
C	31.77594000	82.94322900	29.66113000
C	32.60625600	85.21616500	28.75394000
C	31.14753300	82.42813600	28.36004600
H	30.73629500	84.83734100	29.76779100
H	32.81046100	82.57702300	29.73901500
H	31.23419500	82.51008100	30.51264900
H	31.10331300	81.33236700	28.35262100
H	31.71812500	82.74130000	27.47755500
H	30.12450100	82.80365300	28.23301300
H	32.63223400	86.29268300	28.96054600
H	33.64299000	84.85471400	28.74481900
H	32.19534500	85.08762700	27.74651700
C	24.93837800	75.29808000	29.41363500
C	26.18802100	74.83055300	28.67838800
O	26.14516400	74.22619500	27.59238500
C	24.61528500	76.77482100	29.11546200
C	25.53371600	77.79081100	29.81184000
O	26.33292600	77.41619900	30.69447100
O	25.38187200	79.01606700	29.43429800
H	24.10689100	74.66484600	29.09288500
H	24.63955700	76.95921500	28.03418200
H	23.59097300	76.99822900	29.44037000
N	27.35566200	75.16053300	29.27698100
C	28.63460400	75.05029100	28.58619100
C	29.57645800	76.19352900	28.97860300
C	28.97962200	77.56092500	28.65404200
O	28.38030900	77.73850900	27.58031500
N	29.17225200	78.53335300	29.56339700
H	28.44249200	75.07161300	27.51076500
H	29.84177000	76.13014700	30.04098800
H	30.50941300	76.08837100	28.41054900
H	29.46410500	78.27871600	30.49676700
H	28.61969100	79.39415800	29.51526300
H	27.25865000	75.81093900	30.06030600
C	22.30035000	76.02696400	24.86335300
C	23.65600400	75.61650600	25.39418700
O	24.67050400	76.30672800	25.14736700
N	23.74312600	74.49316900	26.11914900
H	22.08674100	77.05247900	25.18945700
H	21.49274300	75.36427500	25.18696700
H	22.90876000	73.97844700	26.35959300
H	24.62327700	74.28141300	26.61991100
C	26.33625300	75.25945100	20.58086500
C	26.19104500	76.60416500	19.86408200
O	27.00182200	76.96872300	19.01857200
C	26.25862600	75.30572800	22.11814700
C	27.24215400	76.24191200	22.75414100
N	27.29192500	76.42155200	24.12786100
C	28.18295400	77.10164200	22.24601800
C	28.20967500	77.37361500	24.40041600
N	28.77226400	77.80464400	23.27988200
H	25.55668500	74.58011600	20.21277700
H	26.41254600	74.28547000	22.49627200
H	25.24645700	75.58150700	22.44752700
H	28.45436200	77.27939000	21.21733800
H	28.45357400	77.70831000	25.39694200
H	26.60028900	76.06104100	24.78677400
N	25.07895900	77.34047400	20.19178100
C	25.00939400	78.74355600	19.83606900
C	25.42969500	79.63559700	21.01485300
O	25.37706500	79.19999300	22.16898600
H	24.61712300	77.11512500	21.06382500
H	25.65848500	78.89767400	18.97212700
N	25.83554300	80.87451600	20.66579900
C	26.06675300	81.95984600	21.60975100
C	24.92393800	82.98360900	21.42774800
O	24.34148400	83.07220700	20.34056700
C	27.41236500	82.65689700	21.34415600
C	28.67517700	81.79631300	21.47719600
O	28.56031100	80.77375700	22.26663900
O	29.69946100	82.15629400	20.88362700
H	25.59836800	81.19287900	19.73162200
H	26.06706100	81.53894000	22.61401900
H	27.41867200	83.11453900	20.34904500
H	27.52070900	83.46998000	22.07292600
N	24.65311000	83.75882000	22.49762700
C	23.63730300	84.80866200	22.44247800
C	24.09325600	86.09463700	21.72362500
C	22.91053000	87.06485200	21.59266900
C	25.27694800	86.75592900	22.44064100
H	23.34511900	85.03878400	23.47281100
H	24.41142000	85.79787600	20.71584300
H	23.20573800	87.96879100	21.04686400
H	22.07560700	86.60521000	21.04992800
H	22.54261600	87.37737100	22.57828800
H	26.11588400	86.05746300	22.54454400
H	25.63347100	87.63171500	21.88523000
H	24.98468400	87.09047200	23.44433500
H	25.20166000	83.62546900	23.34820000
C	19.14537900	86.68746000	25.58711100
C	20.60220400	87.06405800	25.73446900
C	21.01473700	88.39944300	25.63048600
C	21.57472900	86.08918800	26.00054000
C	22.35591600	88.75272600	25.79527200
C	22.91947300	86.43313900	26.16447500
C	23.31219400	87.77141200	26.06401100
H	18.63154000	86.69337900	26.55812100
H	18.61332000	87.38895800	24.93546200

H	20.27721400	89.17045700	25.41718500
H	21.27539500	85.04531400	26.07340500
H	22.65333300	89.79488900	25.70966600
H	23.66225900	85.66353100	26.35678300
H	24.35696900	88.04386300	26.18953400
C	21.13628900	79.26189600	27.85151900
C	21.43818900	79.11289000	29.34092200
O	21.46583400	78.01364700	29.89338600
C	22.41827500	79.56157300	27.06481600
O	22.17756800	79.35554000	25.68268900
H	20.72485800	78.30930500	27.50971900
H	22.74040400	80.59980000	27.25637300
H	23.22308500	78.90755500	27.42850800
H	23.05559500	79.37825200	25.23747000
N	21.70962100	80.27441200	30.00112200
C	22.28481900	80.27555400	31.34194600
C	22.13767200	81.65559600	32.00746000
C	22.84367000	82.75513700	31.23875300
C	22.13596100	83.60561200	30.37604700
C	24.23572300	82.90505400	31.32831500
C	22.79995900	84.56611400	29.60712700
C	24.90087200	83.86776700	30.56867500
C	24.18663000	84.69588100	29.69740700
H	23.34499300	79.99086000	31.29076500
H	21.07149000	81.89177300	32.11030600
H	22.55139100	81.58077900	33.02059200
H	21.05255000	83.51904500	30.31223600
H	24.80582800	82.26049300	31.99342600
H	22.23756500	85.21195900	28.93817500
H	25.97921300	83.96630800	30.66166600
H	24.70640700	85.42827900	29.08708900
H	21.83481000	81.11868600	29.45593200
C	20.93310000	76.30112900	19.36393300
C	20.02954700	77.54694600	19.41807900
C	20.60157700	78.65158300	20.28335600
C	20.52416600	78.57945700	21.68246600
C	21.25303200	79.75360000	19.71142900
C	21.09026200	79.56829700	22.48966100
C	21.82399800	80.75013400	20.50951400
C	21.74757900	80.65242100	21.90087700
H	21.06000800	75.86707100	20.36287200
H	19.87334100	77.92327200	18.39947200
H	19.04098900	77.25379800	19.79598700
H	20.00669400	77.73992400	22.14442600
H	21.30602900	79.83590800	18.62704600
H	21.02756500	79.49670700	23.57232800
H	22.33519700	81.59570000	20.05734700
H	22.19366200	81.42212300	22.52500800
C	32.87983900	76.46625700	19.67339000
C	32.07506400	77.56808900	20.27930000
N	31.74202500	78.73066500	19.59392600
C	31.50463200	77.71943500	21.51427700
C	30.99957300	79.52317000	20.40116200
N	30.83680800	78.93056200	21.57408700
H	33.87010500	76.81126200	19.34951500
H	33.02978300	75.67077500	20.40818300
H	31.52193900	77.05207800	22.36155300
H	30.62421300	80.51095200	20.16220800
H	31.99373100	78.94978400	18.64062500
C	32.76594300	84.95753200	21.34126500
C	32.13065100	84.08584600	22.42005300
N	31.60714200	84.93258800	23.50083400
C	30.73603100	84.52983900	24.44366800
N	30.37104300	83.25629600	24.54141100
N	30.24548400	85.43325900	25.31695800
H	33.60365500	85.54660300	21.73614600
H	32.03253200	85.64399700	20.90455600
H	31.30428200	83.50386900	21.99923100
H	32.86045400	83.37190300	22.82039000
H	31.97913600	85.86750600	23.59532200
H	30.89246000	82.49028500	24.10574100
H	29.61025100	83.02667200	25.18069200
H	30.16822000	86.41012200	25.04405800
H	29.49219300	85.09638600	25.91990700
Fe	29.86528200	79.66093600	23.20769300
C	27.03255900	81.01416600	27.01897300
C	26.17158800	82.20451600	27.16746000
N	26.45662000	79.68443800	27.01462400
S	26.73967500	83.54407500	26.05565300
O	28.19137500	83.71948300	26.40121200
O	26.53408100	83.04433300	24.66375600
O	25.90721100	84.73433700	26.40189100
H	28.11088700	81.06570200	27.03720300
H	25.12411600	82.00485100	26.92002200
H	26.21021300	82.62143300	28.18349400
H	25.86221100	79.48757800	27.87375000
H	25.88236700	79.48229500	26.15174300
C	32.13170900	80.01862800	24.27504000
O	31.74866700	80.97810000	23.53395500
O	31.41915000	78.97195300	24.37309800
C	33.42715700	80.13457400	25.05800400
C	33.96305700	78.81175200	25.60658300
H	34.19146300	78.11180800	24.79566600
H	34.88039400	78.97765800	26.18206900
O	24.81033800	79.03903200	24.84134300
H	24.73446700	78.05245200	24.84298900
H	25.07310100	79.27345500	23.92035500
H	33.22574200	78.33421800	26.25770800
H	34.16100300	80.62963400	24.41125200
H	33.22832700	80.83754300	25.87957100
O	29.10806700	80.31988700	24.69442400
H	32.37840900	76.02458600	18.80277000
H	23.98138400	79.00971900	19.56203800
H	33.15529900	84.32333300	20.53931200
H	32.14995100	84.73196000	30.80685400
H	28.67791600	81.21485600	36.15016400
H	31.27264900	88.91283300	26.91727100
H	20.50335600	75.52995900	18.71372800
H	21.92984700	76.55300300	18.98349900
H	27.29885300	74.85296400	20.26018100
H	21.76569600	79.50964900	31.92374100
H	29.11732200	74.09162900	28.81622500
H	25.06485000	75.17539900	30.49366800
H	22.76261400	84.40014700	21.92509800
H	19.03106000	85.68239300	25.16664500
H	22.33201500	76.03962700	23.76829600
H	20.39128100	80.04478700	27.66354500
H	28.37134900	80.86600500	24.35639000
H	27.22594000	78.98271800	27.08888700

^{5}TS2$_{reb,C1S,B}$

C	30.82105500	88.39407800	25.01384900
C	29.45443300	87.79533600	25.15337900
N	28.41967200	88.14165700	24.30158700
C	29.01000500	86.87046500	26.07136700
C	27.37577900	87.44238300	24.71746400
N	27.67817800	86.65882300	25.78803100
H	30.78231900	89.48792900	25.07890300
H	31.49165900	88.02990500	25.79905400
H	29.50715200	86.36243300	26.88429300
H	26.38608400	87.46708100	24.28072800
H	27.08650400	85.92326400	26.18455800
C	28.27479600	83.94351800	34.07202400
C	27.97234900	83.22259300	32.77764200
C	27.68877400	81.85005300	32.76073800
C	27.93372500	83.90975300	31.55583700
C	27.37204400	81.17755900	31.57852000
C	27.61257400	83.26111900	30.36417600
C	27.32433300	81.89245400	30.37393100
O	27.04113300	81.28276900	29.17843900
H	28.90814600	84.82184500	33.90441900
H	28.78972200	83.29012000	34.78565600
H	27.71766800	81.28840300	33.69277300
H	28.14748800	84.97655500	31.53695000
H	27.17491200	80.10828700	31.57228000
H	27.56705000	83.79974000	29.42181000
H	26.51560700	80.42353000	29.29875000
C	31.69193800	80.52945200	31.61334800
C	32.30746400	81.60216700	30.69782100
C	32.30371500	80.46717100	33.01845300
C	32.05815800	83.04295200	31.15968400
H	30.60983400	80.70546700	31.70127200
H	33.38931000	81.42319900	30.61199100
H	31.89640500	81.47898100	29.68573400
H	32.48136800	83.76076200	30.44724900
H	32.51600500	83.24204200	32.13532900
H	30.98441300	83.24814000	31.24536400
H	31.85995400	79.65534500	33.60589800
H	33.38611600	80.29254400	32.96987200
H	32.14081200	81.39885200	33.57083900
C	26.00457100	75.42503800	29.41245000
C	27.27617500	75.20134400	28.60624000
O	27.29566400	74.53903400	27.55214600
C	25.31925300	76.74819000	29.02483400
C	26.03024600	78.01582300	29.52501400
O	26.93159200	77.93513600	30.38287400
O	25.59423400	79.11882200	29.01433500
H	25.32805500	74.58849200	29.21621200
H	25.20342100	76.80622000	27.93509700
H	24.29875100	76.76685200	29.42618900
N	28.37427900	75.81693800	29.10038000
C	29.59610000	75.96174300	28.31816000
C	30.21792200	77.34990800	28.49464900
C	29.26513300	78.48085500	28.11258400
O	28.41434200	78.32350900	27.22656400
N	29.43185300	79.64927200	28.76388800
H	29.34526100	75.78084800	27.27058900
H	30.57256100	77.48521700	29.52428300
H	31.09835500	77.42442700	27.84347000
H	29.95685800	79.66408100	29.62768300
H	28.68417500	80.34386700	28.69141300
H	28.17296100	76.49892900	29.83592600
C	23.06984100	73.63537500	24.80006500
C	24.45093100	74.02310100	25.27597600
O	25.14525600	74.82565400	24.61021200
N	24.90330000	73.48939400	26.41718000
H	22.44980500	74.54142400	24.76908900
H	22.58831900	72.88702800	25.43596100
H	24.31855700	72.86615500	26.95448500
H	25.79323000	73.81684500	26.82988900
C	26.80402700	75.37973000	20.26316000
C	26.18051600	76.71815500	19.86611500
O	26.67530100	77.41957800	18.99132500
C	26.83162500	75.05559200	21.76518800
C	27.47769300	76.12081900	22.60086600
N	27.54876700	76.01822000	23.98113100
C	28.06488000	77.32413600	22.29972700
C	28.13762400	77.13121700	24.46442400
N	28.46429100	77.94578200	23.46860900
H	26.26274300	74.58170800	19.73857400
H	27.36638400	74.10475400	21.89760100
H	25.82104500	74.86194200	22.15107600
H	28.21421700	77.79481400	21.34034300
H	28.31455200	77.32225500	25.51500800
H	27.01868600	75.34191900	24.53430000
N	25.01534800	77.06395400	20.50365800
C	24.44773100	78.38379800	20.30592700
C	25.04232100	79.44065700	21.25190400
O	25.07040400	79.24863300	22.47904100
H	24.75010200	76.56781900	21.34244300
H	24.60345400	78.65287200	19.26014700
N	25.47949200	80.55849000	20.64635100
C	25.78701100	81.82508000	21.30828700
C	24.66147300	82.80152400	20.89434500
O	24.25155500	82.78511600	19.72982800
C	27.12258700	82.42105000	20.82759800
C	28.43149700	81.75741000	21.27800600
O	28.32743300	80.75477600	22.09047100
O	29.49204600	82.23892300	20.85563600
H	25.25571400	80.67499200	19.66331800
H	25.80864800	81.65080800	22.38279500
H	27.12718300	82.47657600	19.73272600
H	27.18152100	83.45881800	21.17694000
N	24.22000400	83.64692200	21.84763300
C	23.18770000	84.64334500	21.57004900
C	23.70394100	85.91207500	20.86200000
C	22.52108400	86.83603100	20.53946000
C	24.76566600	86.64196900	21.69498800
H	22.73388900	84.91152300	22.53038500
H	24.15852800	85.58716600	19.91680300
H	22.85626600	87.72676300	19.99544400
H	21.77203400	86.32753100	19.92030100
H	22.02711100	87.17617300	21.45916700
H	25.61656100	85.98718900	21.91521700
H	25.14769700	87.51990200	21.16048300
H	24.34456500	86.98635800	22.64863500
H	24.54966200	83.54314000	22.80786800
C	19.71895000	86.86920000	24.37371300
C	21.09818500	87.25147100	24.86283600
C	21.47515800	88.60018500	24.95948900
C	22.03291000	86.27384500	25.22744900
C	22.74452400	88.96032900	25.41071000
C	23.30984200	86.62716700	25.67705800
C	23.66685900	87.97401400	25.77411000
H	19.58907300	87.11121400	23.31069700

H	19.53657600	85.79669900	24.49350900
H	20.76258100	89.37489800	24.68264500
H	21.75877600	85.22316400	25.16991600
H	23.01445300	90.01101600	25.48271000
H	24.01709300	85.84827500	25.94936100
H	24.65382000	88.25487600	26.13244200
C	21.67935400	78.17897000	27.09229400
C	21.86204900	78.22398100	28.60880300
O	22.02531800	77.20085300	29.27781900
C	21.69710300	76.74701300	26.55188800
O	21.76845700	76.70530600	25.12927000
H	20.76001400	78.69706100	26.79458100
H	22.53228800	76.19823800	27.00797100
H	20.78017200	76.22203700	26.83506100
H	22.62063800	77.11951100	24.87312700
N	21.85307200	79.46730900	29.16405100
C	22.31852300	79.68290500	30.53161700
C	21.78381900	80.98640300	31.13191800
C	22.13410700	82.26026600	30.37778700
C	21.21814100	83.32298300	30.35473000
C	23.36245000	82.42959900	29.72013100
C	21.51690600	84.51989900	29.70146600
C	23.66292800	83.62343300	29.05766500
C	22.74090400	84.67243300	29.04588000
H	23.41636700	79.66361600	30.55338700
H	20.69285300	80.91740900	31.22602200
H	22.17651200	81.05284800	32.15727600
H	20.25995100	83.20880300	30.85822000
H	24.09751900	81.62924700	29.71627900
H	20.79079600	85.32905900	29.69679000
H	24.61540700	83.73168500	28.54639500
H	22.97017600	85.59632300	28.52280800
H	21.83766300	80.26843900	28.54374800
C	21.19037900	79.05223800	22.59320100
C	20.33393200	80.32664600	22.70446400
C	20.41921100	80.97853500	24.06935100
C	19.37268400	80.85607600	24.99363400
C	21.55919300	81.70307600	24.44961000
C	19.45663900	81.44173200	26.25979100
C	21.65144700	82.28733000	25.71489200
C	20.59668000	82.16113800	26.62631700
H	20.91314500	78.31357800	23.35253300
H	20.65216400	81.04222700	21.93437400
H	19.28775600	80.07914900	22.48530600
H	18.47882900	80.30175300	24.71447100
H	22.38381400	81.82212600	23.75029800
H	18.62712500	81.34282700	26.95590300
H	22.54249100	82.84956000	25.97835300
H	20.66045700	82.63489600	27.60171400
C	33.46134300	76.81716500	20.77677000
C	32.45021100	77.85196100	21.14738000
N	32.15858500	78.95020600	20.34903900
C	31.62700700	77.99197300	22.23510800
C	31.19684100	79.69017500	20.96108600
N	30.85532300	79.13070100	22.10709400
H	34.46621700	77.24394200	20.66073500
H	33.51452300	76.05516300	21.55927200
H	31.53790700	77.35437100	23.10210000
H	30.77829300	80.61588700	20.58287400
H	32.57221000	79.15706100	19.45121300
C	33.23379100	84.70446600	21.18570600
C	32.17988800	84.10890400	22.11335200
N	30.98540800	84.96179200	22.13466400
C	29.92343400	84.68922900	22.92072300
N	29.98192600	83.76488100	23.86844000
N	28.78811500	85.40462400	22.74213900
H	33.52383000	85.71020300	21.50780200
H	32.86672300	84.76279100	20.15372500
H	31.90160300	83.09681100	21.79515700
H	32.56794000	84.04720000	23.13549100
H	30.73945700	85.39054700	21.25093000
H	30.62270100	82.96597400	23.84266400
H	29.13097500	83.62246000	24.41264600
H	28.86813700	86.40695500	22.59763000
H	28.01444300	85.13409600	23.34770100
Fe	29.30161600	79.89242400	23.58299900
C	25.49399700	80.59200700	25.67595600
C	26.46351000	81.67236800	26.01182100
N	25.90447400	79.29591500	26.29720600
S	26.13416900	83.28786300	25.33404300
O	27.22070200	83.53650500	24.31097600
O	24.79766800	83.19871100	24.68184500
O	26.21395000	84.24422900	26.47475700
H	25.42026800	80.43762700	24.59631500
H	26.95633600	81.68692200	26.97840900
H	27.99666000	81.73000900	24.31748700
H	25.64483200	79.22600700	27.32238400
H	25.45009500	78.51949600	25.76737800
C	31.40029700	80.37721100	24.97181500
O	31.09543700	81.24688100	24.09845400
O	30.71683700	79.30819600	25.06321100
C	32.56824500	80.59953900	25.91401000
C	33.51195900	79.39199900	25.98706500
H	33.95776400	79.18005500	25.00833800
H	34.32521800	79.57769500	26.69674100
O	24.45768700	77.55027900	24.58121100
H	24.74629800	76.61448500	24.48824700
H	24.63771100	78.01080100	23.72634400
H	32.96920100	78.49789200	26.30739700
H	33.10307100	81.50184800	25.60233000
H	32.14185800	80.79506900	26.90732100
O	28.28601500	80.95526600	24.83568100
H	33.20625200	76.30980800	19.83721000
H	23.37119400	78.34052900	20.50296900
H	34.12766700	84.07309800	21.18346000
H	31.81737000	79.54476600	31.13629700
H	27.35678800	84.29606900	34.56225900
H	31.27159700	88.14594200	24.04391700
H	21.06733300	78.59265600	21.60354200
H	22.25311000	79.28280200	22.73423800
H	27.81833800	75.39691200	19.85560900
H	21.97459200	78.83290400	31.12525500
H	30.33196000	75.20204600	28.61277200
H	26.22917400	75.44189300	30.48317300
H	22.41944500	84.17232500	20.94711400
H	18.93616500	87.40736000	24.92212200
H	23.14260700	73.24442800	23.78003500
H	22.50167300	78.74429700	26.63096500
H	24.48450400	80.82582500	26.03848700
H	26.93074000	79.17118600	26.28335800

$^{5}TS2_{reb,C1R,B}$

C	28.66060800	87.91226000	29.97753500
C	27.57088800	87.32080100	29.13612800
N	26.30514900	87.87286100	29.10105400
C	27.62549900	86.21720800	28.31318700
C	25.61624100	87.11130100	28.27318300
N	26.36613100	86.09407500	27.76380800
H	28.37546200	87.93739600	31.03673300
H	29.58621000	87.33308100	29.88797300
H	28.41759700	85.52675400	28.06657600
H	24.57436200	87.24504800	28.01317700
H	26.07173900	85.37178900	27.10463700
C	28.58317400	83.35325200	34.90145000
C	28.22366300	82.76919100	33.55377700
C	27.95096200	81.40490700	33.39934000
C	28.13187900	83.58484000	32.41568100
C	27.60002500	80.85985100	32.16160900
C	27.77562400	83.06545900	31.17276500
C	27.50772800	81.69759700	31.04414800
O	27.18982300	81.20750100	29.79697700
H	27.75253600	83.93384000	35.32500700
H	29.44309700	84.03052700	34.83227800
H	28.01722300	80.74690000	34.26380600
H	28.33233000	84.65077200	32.50202800
H	27.40722200	79.79562200	32.05022600
H	27.69005800	83.71066600	30.30314400
H	26.63166300	80.36661500	29.86184900
C	33.15282500	82.69850800	29.30996900
C	31.95510600	82.65798600	28.34543900
C	33.95198700	84.00690800	29.27324900
C	30.84239600	83.66262400	28.66733400
H	32.79402500	82.51993200	30.33381800
H	32.31263200	82.82989800	27.31862500
H	31.52842000	81.64531300	28.35768300
H	30.00399400	83.55308700	27.97042800
H	31.19795200	84.69762300	28.59995900
H	30.45228000	83.50846900	29.68084400
H	34.81717300	83.96056600	29.94493200
H	34.32649800	84.21223000	28.26192600
H	33.34326100	84.86432200	29.58076400
C	26.01270000	75.38285400	29.55373500
C	27.16348400	75.24942900	28.56508000
O	27.03717200	74.70062200	27.45460900
C	25.30056600	76.73820300	29.39921000
C	26.11551800	77.94371500	29.89153900
O	27.09930700	77.77219500	30.64041300
O	25.68364600	79.09009800	29.48976600
H	25.30853300	74.56782200	29.36532700
H	25.01781200	76.90085300	28.35233700
H	24.35719300	76.73033600	29.96054000
N	28.32224800	75.81781500	28.96986100
C	29.47419900	75.97823100	28.09209900
C	30.23193800	77.28082000	28.38060800
C	29.37125200	78.52510000	28.19594400
O	28.69876500	78.67376500	27.15649900
N	29.41560100	79.44891400	29.16710000
H	29.12364700	75.95625100	27.05800300
H	30.66663800	77.25557000	29.38706500
H	31.06136900	77.35804500	27.66748800
H	29.79290800	79.19974800	30.07092600
H	28.71581000	80.19995200	29.18491800
H	28.23873900	76.39978100	29.80641400
C	22.43091800	74.67084600	25.24800700
C	23.90485900	74.78646000	25.56080700
O	24.63495100	75.56473200	24.90495900
N	24.40308300	74.03671400	26.55309000
H	21.96131600	75.64650800	25.43046500
H	21.92247400	73.90623600	25.84205900
H	23.79538300	73.44177800	27.09710200
H	25.37379300	74.18437000	26.87395100
C	25.98889700	75.54723000	20.54712700
C	25.37902100	76.92884400	20.29983100
O	25.64817300	77.56634100	19.28814100
C	26.27637500	75.15932500	22.00591800
C	27.14387400	76.14920600	22.72565300
N	27.23002200	76.18301000	24.10772300
C	27.96545000	77.15967000	22.29128900
C	28.05573200	77.19163000	24.45903600
N	28.52650000	77.79750800	23.37937800
H	25.31347000	74.80184100	20.10720900
H	26.75207300	74.16806600	22.00681300
H	25.34376400	75.02907500	22.56943600
H	28.16953800	77.49089600	21.28481000
H	28.28759100	77.46963100	25.47725400
H	26.59924900	75.68310700	24.73569300
N	24.46993500	77.37971900	21.22239400
C	23.92453300	78.72266800	21.10183200
C	24.85566500	79.79100300	21.69252800
O	25.13147000	79.77887200	22.90506600
H	24.41829300	76.93386700	22.12778400
H	23.72541900	78.90577100	20.04487400
N	25.32315000	80.70353400	20.82716700
C	25.99798700	81.94127700	21.19757200
C	25.08423700	83.10241200	20.75280500
O	24.39593400	82.99777700	19.73380800
C	27.37046700	82.11194500	20.51136000
C	28.58590700	81.48510400	21.21152100
O	28.33694500	80.58101300	22.08685100
O	29.71202800	81.89478600	20.86825300
H	24.93539200	80.71080200	19.89021000
H	26.13583800	81.93950900	22.27615500
H	27.32498100	81.71849000	19.48768300
H	27.59550500	83.17875800	20.41487600
N	25.15318700	84.20338100	21.53655500
C	24.46573500	85.44974200	21.21822900
C	25.41823700	86.60527100	20.85167900
C	24.59871000	87.82340100	20.40099600
C	26.35178100	86.97354100	22.01560900
H	23.84513900	85.74268300	22.07469200
H	26.03322900	86.26510400	20.00558300
H	25.25303400	88.65352000	20.10971600
H	23.96149300	87.58027500	19.54234500
H	23.94957900	88.17876900	21.21148100
H	26.94298200	86.11265200	22.35008600
H	27.04426100	87.77172100	21.71902000
H	25.77364000	87.33458500	22.87567700
H	25.71565400	84.14591300	22.38535500
C	19.48832800	86.49978700	23.46930500
C	20.87516600	86.78501600	23.99956800
C	21.32506500	88.10227000	24.16658700
C	21.73724100	85.73860600	24.35770800
C	22.59477200	88.36876700	24.68307900
C	23.01108900	85.99615200	24.87279300
C	23.44216600	87.31645200	25.03832200
H	19.44617500	85.53203200	22.95847100
H	18.74962600	86.47075600	24.28192200

H	20.67274800	88.92796100	23.88919500
H	21.41047800	84.70963500	24.22284300
H	22.92194000	89.39802500	24.80776800
H	23.67290700	85.17397300	25.13311300
H	24.43056500	87.51959200	25.44189200
C	20.98770300	78.00825500	28.31354100
C	21.57948800	78.13163800	29.71505100
O	22.03011000	77.15545800	30.31556300
C	22.16059100	77.85487000	27.33951800
O	21.70571800	77.86681700	25.99351400
H	20.34039500	77.12726200	28.26785100
H	22.87444100	78.67430500	27.50674300
H	22.68564400	76.91694700	27.57456000
H	22.48317000	78.14085000	25.45746900
N	21.65402600	79.40257000	30.19457200
C	22.53605500	79.76888400	31.29964400
C	22.26958800	81.21781300	31.74779500
C	22.32491900	82.21439500	30.60379900
C	21.14780700	82.79307700	30.10683100
C	23.54175400	82.54234200	29.98371600
C	21.17927700	83.67222800	29.02020100
C	23.57639100	83.42099600	28.89991800
C	22.39396400	83.98657200	28.41133400
H	23.58211900	79.65317600	30.98532400
H	21.28633500	81.27144500	32.23046700
H	23.01659600	81.47139800	32.50994300
H	20.19760800	82.55902300	30.58328300
H	24.46963100	82.10820400	30.34779200
H	20.25493800	84.11235000	28.65475700
H	24.52596600	83.67202900	28.43672300
H	22.42301200	84.66959500	27.56671500
H	21.38890500	80.15736500	29.57395700
C	19.76002700	77.51469200	22.78358300
C	19.16952000	78.91791900	22.55527700
C	20.20585200	80.02291400	22.62039000
C	20.80046200	80.36981600	23.84456800
C	20.60761600	80.71114100	21.46673000
C	21.76192300	81.37911100	23.91013500
C	21.57515000	81.71919100	21.52558800
C	22.15156700	82.05850000	22.75205500
H	20.23269100	77.44407000	23.76930700
H	18.66603600	78.94819800	21.58095100
H	18.39322600	79.09980300	23.31074300
H	20.52470000	79.83159100	24.74794500
H	20.15423500	80.45647400	20.51049800
H	22.20755700	81.63828200	24.86752500
H	21.89116000	82.23396500	20.62238100
H	22.90153100	82.84235100	22.80370800
C	33.03660800	76.18061100	20.43221900
C	32.16951300	77.32618100	20.83946300
N	31.97657900	78.45306600	20.04942300
C	31.41302800	77.55711900	21.95763700
C	31.13577100	79.30153000	20.69107200
N	30.77517200	78.78015000	21.85210000
H	34.07853900	76.48788200	20.27469400
H	33.03144100	75.41711000	21.21477400
H	31.27863400	76.93433900	22.82847700
H	30.81630000	80.27474200	20.33740500
H	32.38521100	78.61682400	19.14035300
C	32.06869600	84.28571400	20.52615800
C	31.91780800	83.84446900	21.97858400
N	31.08656400	84.80501600	22.71247900
C	30.37408100	84.53758800	23.81890800

N	30.41901800	83.36149300	24.42567300
N	29.63612300	85.53662500	24.37447300
H	32.48402500	85.29895500	20.44435300
H	31.10015400	84.24617800	20.02051200
H	31.41601700	82.87527400	21.99662700
H	32.89833000	83.75281900	22.46678100
H	31.17137300	85.78003400	22.45806700
H	30.93925200	82.55915300	24.06177900
H	29.65311400	83.15471500	25.07475700
H	29.22946400	86.20446000	23.73150900
H	28.98215200	85.21190100	25.08988400
Fe	29.58679000	79.66406500	23.35636700
C	26.05657100	81.14981900	26.68369800
C	26.55965700	81.59541800	25.34975000
N	26.05370100	79.66234400	26.83629400
S	26.83844800	83.34526200	25.22031000
O	28.08658100	83.64806400	26.00652000
O	26.99436900	83.66605400	23.77535700
O	25.63144900	83.95626700	25.85898600
H	25.02405700	81.49363600	26.83227200
H	28.93625200	80.05740900	25.57215100
H	26.25221800	81.09054900	24.43952700
H	25.73221700	79.40598400	27.80981900
H	25.44295600	79.18314200	26.13269600
C	31.78878900	79.94619400	24.61587000
O	31.40581600	80.82699900	23.77181600
O	31.04858500	78.95229800	24.86079400
C	33.15073200	80.10455000	25.26992100
C	33.44837700	79.10859900	26.38974000
H	33.36720900	78.07823400	26.03015300
H	34.46098800	79.25975500	26.77930700
O	24.28997400	78.36332200	25.01630000
H	24.48522400	77.40648700	24.90782600
H	24.51459300	78.81176300	24.15537500
H	32.74548800	79.23204900	27.22005400
H	33.89403800	80.01908400	24.46519600
H	33.22359400	81.13770400	25.63024100
O	28.60462800	80.43453400	24.73062100
H	32.68528700	75.70861400	19.50562400
H	22.97942500	78.77655000	21.65024600
H	32.75172700	83.60658100	20.00621600
H	33.82695700	81.86315300	29.07288600
H	28.83373400	82.56791800	35.62274400
H	28.87645000	88.94673900	29.68153200
H	18.97735700	76.74865800	22.72596300
H	20.51640500	77.28111300	22.02397600
H	26.90637700	75.52093800	19.95236400
H	22.36202000	79.07310100	32.12527500
H	30.16759400	75.13568900	28.21401200
H	26.37870700	75.28910300	30.58036700
H	23.80144300	85.23095900	20.37848900
H	19.16148300	87.27140100	22.76340100
H	22.30855400	74.43870500	24.18529700
H	20.39603000	78.88715100	28.03257900
H	26.64823300	81.56286300	27.50610700
H	27.02310500	79.30432100	26.77230400

^{5}TS2$_{reb,C2S,B}$

C	29.89038300	89.48274000	26.86111000
C	28.77064800	88.50695900	26.66623300
N	28.17463600	88.32126200	25.42884900
C	28.16647300	87.69335900	27.59864500
C	27.22147700	87.42538300	25.63488300
N	27.17244900	87.01325900	26.92771700
H	29.56685500	90.50824100	26.64431300
H	30.26077300	89.45528700	27.89104700
H	28.34664700	87.54712000	28.65303300
H	26.55420500	87.02907100	24.88191600
H	26.64379000	86.19471900	27.24930400
C	28.80484700	82.40553100	35.13245100
C	28.46097800	81.90853400	33.74621200
C	27.91508200	80.63534600	33.54214700
C	28.69645700	82.70351800	32.61489500
C	27.61468600	80.15782500	32.26436000
C	28.39666100	82.25184900	31.33184500
C	27.84922800	80.97543400	31.15125500
O	27.58485000	80.55374500	29.87359600
H	28.47279000	83.43958100	35.28479700
H	29.88829100	82.38709200	35.31420700
H	27.72471500	79.99412000	34.40078800
H	29.11554400	83.70015900	32.74016000
H	27.22320000	79.15519900	32.11459300
H	28.57342200	82.87794200	30.46182500
H	26.86585400	79.83691000	29.86449800
C	33.25321800	84.60556000	28.85695400
C	31.99165900	84.01930400	28.20069000
C	33.57827500	86.04204600	28.42985800
C	30.68405200	84.71963000	28.58877800
H	33.13930400	84.56725500	29.94995000
H	32.10715400	84.05064600	27.10673000
H	31.91785100	82.95661000	28.47028800
H	29.82332600	84.22851700	28.12237100
H	30.67179600	85.76791800	28.26910800
H	30.53954200	84.69975800	29.67708200
H	34.50527600	86.39412900	28.89744100
H	33.70805400	86.10937500	27.34182900
H	32.78199700	86.74015700	28.71065600
C	25.40620100	75.07196000	29.42223600
C	26.56526800	74.71040200	28.50310100
O	26.40026400	74.18854600	27.38459400
C	24.96575300	76.53493900	29.23192300
C	25.93948400	77.58275800	29.79570900
O	26.86015000	77.23193200	30.56251000
O	25.69512900	78.79803300	29.44230000
H	24.57184800	74.40247200	29.19421000
H	24.79331100	76.74377900	28.16982100
H	23.99802000	76.68675900	29.72668400
N	27.78596100	75.03790000	28.98289600
C	28.98190100	75.02167600	28.14883100
C	29.89885100	76.20659900	28.46844500
C	29.19893900	77.55144500	28.28113600
O	28.38244500	77.71122700	27.36405700
N	29.54626200	78.53413500	29.13712200
H	28.66646400	75.04958100	27.10343400
H	30.29615500	76.11985700	29.48739300
H	30.76046200	76.17977300	27.78911800
H	30.01841200	78.28481100	29.99503100
H	28.94732300	79.36360600	29.20197700
H	27.75776900	75.65652900	29.79754300
C	21.98008400	74.75910100	24.88385200
C	23.44423600	74.65095700	25.24648000
O	24.31116500	75.24345400	24.56200000
N	23.77519300	73.92778400	26.32276200
H	21.64893800	75.78362300	25.10322200
H	21.34795300	74.05443100	25.43166100
H	23.05540800	73.49315800	26.88199400
H	24.74203400	73.94816400	26.69113700
C	25.85460800	75.60835400	20.30132400
C	25.39148200	77.06546500	20.22456400
O	25.76242600	77.79924700	19.31567700
C	26.08303800	75.01721200	21.70300900
C	26.98807400	75.85741100	22.55460600
N	26.98436600	75.77714000	23.93817100
C	27.88365600	76.85430400	22.25733200
C	27.82211500	76.71726900	24.42582300
N	28.38816800	77.38393700	23.42880300
H	25.11590100	74.99299200	19.77109500
H	26.49281300	74.00418500	21.58291200
H	25.13098300	74.88191400	22.23200500
H	28.17067400	77.25477300	21.29680600
H	27.98914000	76.90305700	25.47904800
H	26.29889900	75.25343100	24.48353400
N	24.49511600	77.49128700	21.17200900
C	24.07361800	78.88084700	21.18687700
C	25.08091600	79.81134000	21.87669100
O	25.44125700	79.58443300	23.05061400
H	24.33295500	76.93388000	21.99909000
H	23.89940500	79.19046800	20.15511200
N	25.49424600	80.86240100	21.16249400
C	26.21060900	82.02706100	21.67678700
C	25.32302800	83.24620700	21.34195700
O	24.73896500	83.28495700	20.25334200
C	27.56783300	82.24475000	20.97764100
C	28.73752200	81.33041000	21.36184500
O	28.44820600	80.26208700	22.03335700
O	29.87193200	81.66863100	20.99744400
H	25.07509900	81.02252200	20.25174500
H	26.34379000	81.90693400	22.75240200
H	27.42718200	82.18148800	19.89143700
H	27.90146100	83.26939800	21.17598000
N	25.28984500	84.23241300	22.26104700
C	24.51828400	85.45548700	22.03358900
C	25.20627600	86.46422000	21.09241600
C	24.24586200	87.62482500	20.79656800
C	26.53584700	86.97549200	21.66214200
H	24.33400900	85.90924200	23.01234000
H	25.40844900	85.93429200	20.15258000
H	24.70798300	88.35590100	20.12289900
H	23.32361900	87.26831600	20.32271900
H	23.96603300	88.14983000	21.71913600
H	27.20698200	86.14796100	21.92052300
H	27.05421800	87.61226300	20.93530800
H	26.37565200	87.57823600	22.56581100
H	25.65282300	84.04705900	23.19640500
C	19.84876800	85.26018100	23.83880700
C	21.01649500	86.01022100	24.43923600
C	21.09985900	87.40676900	24.36067600
C	22.04241000	85.32193700	25.10398600
C	22.17173700	88.09738300	24.93158000
C	23.11887600	86.00379500	25.67617800
C	23.18405900	87.39857000	25.59210800
H	19.13668600	84.94545000	24.61364500
H	19.29937400	85.88108300	23.12314200

H	20.31715300	87.95916200	23.84473300
H	22.00094000	84.23666500	25.16741800
H	22.21462000	89.18142800	24.85942500
H	23.90810400	85.44634400	26.17363300
H	24.01947400	87.93331600	26.03685600
C	20.96805200	78.39317500	28.11461700
C	21.43748300	78.30592900	29.56383200
O	21.62672300	77.21831100	30.11071500
C	22.11282600	77.89182700	27.22906000
O	21.77590500	78.01280300	25.85373900
H	20.08858000	77.75467500	27.98115400
H	23.01837900	78.47293300	27.45424700
H	22.32403400	76.84669800	27.49666400
H	22.63201100	78.08832100	25.37619600
N	21.70019900	79.49710100	30.16644300
C	22.45612800	79.58953300	31.41101000
C	22.22409800	80.94687400	32.09951600
C	22.58317300	82.11989500	31.20806900
C	21.58484200	82.86044700	30.55819400
C	23.92579000	82.44567400	30.95838800
C	21.91506700	83.89028500	29.67164900
C	24.25806800	83.47201200	30.07387200
C	23.25330000	84.19388500	29.42164200
H	23.52628400	79.45185900	31.20099900
H	21.17393300	81.01914900	32.40723100
H	22.83128600	80.96360600	33.01306300
H	20.53883500	82.62955700	30.75212500
H	24.71670300	81.88779200	31.45484800
H	21.12708000	84.45088900	29.17507000
H	25.29980300	83.70885500	29.88066800
H	23.51919600	84.97851800	28.72067600
H	21.65470600	80.33860100	29.60570400
C	19.67619300	77.91268000	20.80933900
C	19.13188900	79.18231300	21.48915900
C	20.22709500	80.08006500	22.02878800
C	20.73918800	79.89224900	23.32155500
C	20.78073800	81.09627900	21.23584100
C	21.76787600	80.70281300	23.80850100
C	21.81418700	81.90579200	21.71475900
C	22.30885200	81.71348100	23.00932300
H	20.26013000	77.31051400	21.51531100
H	18.52169700	79.74072900	20.76782400
H	18.46066400	78.89072200	22.30686800
H	20.33950700	79.11062300	23.96315500
H	20.39595100	81.25685800	20.23033900
H	22.13815000	80.53258400	24.81513300
H	22.24593300	82.67125500	21.07598900
H	23.11044600	82.34123500	23.38952200
C	33.44878200	75.98951600	21.19293000
C	32.47741200	77.08457600	21.48856000
N	32.42532700	78.27087800	20.76666500
C	31.49000100	77.21444600	22.43044300
C	31.43772200	79.05000500	21.27958600
N	30.85204900	78.43120000	22.28941500
H	34.49016200	76.32175400	21.29560900
H	33.29606200	75.16197100	21.89117400
H	31.19239700	76.51566700	23.19803800
H	31.16025700	80.03699700	20.92505000
H	33.01088700	78.51225100	19.97996300
C	31.96866000	85.21270400	20.77111900
C	31.55581100	84.34394500	21.95662300
N	30.90463200	85.16603400	22.98155800
C	30.09703900	84.69741400	23.95456300

N	29.80414500	83.40402700	24.05630200
N	29.57101900	85.55706300	24.84283600
H	32.67791000	85.99591500	21.06836600
H	31.09662800	85.69061900	20.31305300
H	30.85581100	83.56970000	21.62771900
H	32.43613300	83.83832500	22.38026500
H	31.24528400	86.11020800	23.10490100
H	30.38663200	82.65433500	23.68934000
H	29.13431100	83.13809800	24.78609100
H	29.42421800	86.53848000	24.62452900
H	28.93962400	85.15204500	25.53738900
Fe	29.24148700	79.36460300	23.60508800
C	26.11037700	80.69926600	26.20751300
C	26.20589000	81.84086300	27.15348900
N	26.18313300	79.35447100	26.77010800
S	26.55116600	83.43047100	26.31916500
O	28.04994800	83.55165600	26.24235300
O	25.90089100	83.35640300	24.97934300
O	25.95496700	84.46241200	27.22179000
H	27.05617300	79.98461700	24.06910800
H	25.24885700	82.01555000	27.67140900
H	26.96760600	81.70042400	27.92680100
H	25.82840200	79.24735900	27.75932600
H	25.63617700	78.70867300	26.16094700
C	31.20408200	80.04162800	25.01278600
O	30.78336000	80.79943300	24.07067000
O	30.62343700	78.93395400	25.20806400
C	32.38542400	80.47998200	25.85222300
C	32.80690500	79.48032900	26.92930600
H	33.09170300	78.52136000	26.48438000
H	33.66405600	79.86566300	27.49244800
O	24.43038600	78.00523200	24.97901300
H	24.50055900	77.04410900	24.78871100
H	24.73118300	78.49248600	24.16770000
H	31.98775700	79.29382900	27.63015300
H	33.21059300	80.68786000	25.15766700
H	32.12430200	81.44916400	26.29671500
O	27.80360700	79.88367700	24.69405500
H	33.32622800	75.59228400	20.17676900
H	23.13191000	78.96309600	21.73862800
H	32.45695000	84.59319900	20.01280100
H	34.11045900	83.96062900	28.61830200
H	28.33473300	81.78784800	35.90517600
H	30.73263700	89.26475500	26.19250900
H	18.85898000	77.28940900	20.42748800
H	20.33038100	78.16779900	19.96762200
H	26.77358800	75.56583000	19.71001400
H	22.13572700	78.76627500	32.05441800
H	29.53971700	74.08778200	28.29705900
H	25.68874200	74.91976000	30.46812200
H	23.54723400	85.17745400	21.60962700
H	20.17925600	84.35319200	23.32002100
H	21.86261300	74.58972100	23.80964100
H	20.69794600	79.41400000	27.82075800
H	25.47439600	80.78816600	25.33829100
H	27.16183800	78.99996600	26.79481300

$^{5}TS2_{reb,C2R,B}$

C	29.86081400	89.32322500	27.41353800
C	28.83509600	88.36343000	26.89254400
N	28.66627200	88.14828000	25.53414600
C	27.96007200	87.57018100	27.59935200
C	27.69890300	87.25204700	25.43770400
N	27.23454400	86.87052100	26.65591100
H	29.84176600	90.25784400	26.84257200
H	29.68098700	89.56042200	28.46716600
H	27.79586900	87.44139600	28.65884100
H	27.30779000	86.83654800	24.51975000
H	26.60927000	86.08275000	26.80922400
C	28.14728000	82.38512300	35.11990400
C	28.03813200	81.87206900	33.70177100
C	27.39862200	80.65998200	33.41138700
C	28.55723500	82.60583900	32.62506500
C	27.26894200	80.18868400	32.10290300
C	28.42839600	82.16004400	31.31087200
C	27.77712500	80.95029200	31.04235700
O	27.69343100	80.52984300	29.73793000
H	27.39575700	83.15844400	35.33022900
H	29.12950500	82.83234400	35.31333100
H	26.99798500	80.06090300	34.22699800
H	29.06674800	83.54827900	32.81682300
H	26.80628400	79.22804300	31.89297900
H	28.82851600	82.73672100	30.48154100
H	26.90448400	79.91159800	29.60627200
C	32.65291900	85.45457600	29.35138900
C	32.69299000	83.94530500	29.64447900
C	33.31780200	85.86415300	28.03097900
C	31.91154500	83.07978100	28.64812500
H	31.60690700	85.79347500	29.34914400
H	33.74117800	83.61315600	29.66865500
H	32.29612300	83.77241800	30.65391800
H	31.93910200	82.02469800	28.94759600
H	32.32561800	83.14906500	27.63543700
H	30.85870200	83.38495600	28.59088800
H	33.39469500	86.95521300	27.95285000
H	34.33369200	85.45416300	27.95676300
H	32.74833300	85.51467100	27.16313300
C	24.97126100	75.27250800	29.23515200
C	26.20655200	74.73459600	28.52433800
O	26.14971900	74.09792200	27.45647600
C	24.75209600	76.75399500	28.86644000
C	25.79987600	77.72317900	29.42956400
O	26.53024300	77.37293800	30.37916500
O	25.77121600	78.91770600	28.93524500
H	24.11453700	74.68472800	28.89704400
H	24.68993000	76.86715900	27.77844700
H	23.78526700	77.08824900	29.26492800
N	27.37562400	75.00892000	29.14467900
C	28.66837500	74.75162000	28.52519100
C	29.71069300	75.76349700	29.01507300
C	29.29793600	77.19746500	28.68772300
O	28.88994600	77.49677400	27.55711400
N	29.43319600	78.10853400	29.67218400
H	28.55152600	74.83111600	27.44080800
H	29.89414100	75.65033600	30.09035200
H	30.65839500	75.55438400	28.50403400
H	29.54844800	77.79637000	30.62585900
H	28.96728500	79.01165600	29.56278500
H	27.30628200	75.69573300	29.89834400
C	22.33617700	76.01504700	24.79978600
C	23.66045500	75.54037400	25.35696000
O	24.71231500	76.17511600	25.13470800
N	23.67869100	74.41287700	26.08435300
H	22.19550500	77.06469600	25.08411800
H	21.48441900	75.42102900	25.14335700
H	22.82096100	73.92311300	26.29124500
H	24.55416300	74.13913800	26.56144600
C	25.89203600	75.30763600	20.49670100
C	25.80467800	76.64822600	19.76701700
O	26.72486400	77.07967800	19.08189500
C	25.90447100	75.40203700	22.03859200
C	27.01090600	76.21258400	22.64975800
N	27.32274200	76.08366400	23.99697000
C	27.83030100	77.21517400	22.19397200
C	28.26330100	77.00435500	24.30748700
N	28.59910900	77.70387800	23.23311100
H	25.02132900	74.70111000	20.21556100
H	25.93936100	74.38173700	22.44229000
H	24.94591300	75.81653000	22.38313900
H	27.92168400	77.61165800	21.19578300
H	28.68138900	77.12547300	25.29660000
H	26.75461700	75.58058600	24.67355400
N	24.62080000	77.33365700	19.92263600
C	24.58855200	78.75179900	19.62197300
C	25.17897300	79.57981400	20.77881800
O	25.23595600	79.08194100	21.90591300
H	24.04190300	77.06221900	20.70732800
H	25.15397000	78.91864900	18.70315100
N	25.61427600	80.80428800	20.41543500
C	25.96081500	81.88593000	21.32894700
C	24.82272600	82.92868300	21.26983300
O	24.07721900	82.99487000	20.28689600
C	27.30715000	82.52845900	20.94313000
C	28.57826000	81.76192000	21.35984900
O	28.38834200	80.78103400	22.17600400
O	29.66349700	82.16714100	20.91996500
H	25.30392300	81.13746800	19.50829000
H	26.04663200	81.46353100	22.32769800
H	27.34787600	82.71069500	19.86307500
H	27.38667400	83.51059500	21.42470600
N	24.73411500	83.74797800	22.34113700
C	23.70148900	84.77644100	22.44563900
C	23.92237900	86.01388900	21.55235200
C	22.73473500	86.97544000	21.70465700
C	25.24760600	86.71876500	21.86424900
H	23.65165000	85.07798800	23.49786600
H	23.94861700	85.65666600	20.51545400
H	22.81537100	87.80995200	20.99828400
H	21.78126000	86.46820000	21.51289600
H	22.69255800	87.39759800	22.71692500
H	26.09973900	86.04210700	21.73357200
H	25.40095200	87.57946300	21.20256300
H	25.25854600	87.08887900	22.89832600
H	25.38551800	83.60216000	23.11033100
C	19.20938900	86.69455100	25.79788900
C	20.65852600	86.99559700	26.10700500
C	21.08978000	88.31395200	26.31378300
C	21.60319300	85.96566200	26.21226500
C	22.42250600	88.59432900	26.61818800
C	22.93962400	86.23751900	26.51988300
C	23.35242500	87.55710600	26.72202900
H	18.60813900	86.63802700	26.71556600
H	18.76314800	87.47250500	25.16838000

H	20.37357200	89.12887600	26.22980100
H	21.29121100	84.93759500	26.03997000
H	22.73690600	89.62444500	26.76616000
H	23.65655600	85.42400300	26.59332200
H	24.39236100	87.77687400	26.94863100
C	21.44497200	79.14783700	27.71528500
C	21.71033800	79.00281200	29.21000600
O	21.76715600	77.90172700	29.75643600
C	22.69942400	79.61075200	26.95818800
O	22.54061000	79.31872500	25.57861000
H	21.17118100	78.15674300	27.34700300
H	22.85968700	80.69256300	27.10839500
H	23.57697800	79.09689500	27.37372600
H	23.43732900	79.29810500	25.17082500
N	21.88320400	80.17196600	29.89777600
C	22.45619500	80.17472600	31.24125200
C	22.11273500	81.46895400	31.99645300
C	22.64720700	82.71717800	31.32333700
C	21.80197100	83.53392200	30.55796000
C	24.00746200	83.05330500	31.40201500
C	22.29946100	84.64826400	29.87760800
C	24.50693300	84.16719100	30.72639600
C	23.65605500	84.96457900	29.95629400
H	23.54642500	80.04205100	31.18321800
H	21.02258000	81.54182500	32.09096600
H	22.51949500	81.37817000	33.01157800
H	20.74177300	83.29537200	30.49927900
H	24.68171100	82.43771500	31.99340800
H	21.63114500	85.26832800	29.28713000
H	25.56299700	84.40978400	30.80553300
H	24.04394800	85.82304000	29.41503800
H	22.02407800	81.01871000	29.35894500
C	20.64428900	76.25892600	19.43292800
C	19.81443900	77.55448400	19.45058700
C	20.43869700	78.66952000	20.26846500
C	20.66716200	78.51583400	21.64537000
C	20.81107800	79.88138000	19.66955300
C	21.25502400	79.53322300	22.40026800
C	21.40894400	80.90351500	20.41414400
C	21.63252900	80.72783400	21.78064500
H	20.80343300	75.87514000	20.44751400
H	19.67474600	77.90262800	18.41981300
H	18.80970000	77.32944600	19.83561700
H	20.36557900	77.59263500	22.13774000
H	20.63166200	80.02560000	18.60566400
H	21.41492700	79.39880500	23.46700400
H	21.72218100	81.82562400	19.93383300
H	22.10405900	81.51652000	22.35997000
C	32.80187500	76.37960000	19.88722400
C	32.07378800	77.52495900	20.51033300
N	31.97758500	78.77781200	19.91677400
C	31.35952800	77.64169900	21.67358400
C	31.23024400	79.58207800	20.71467000
N	30.84263200	78.91925600	21.79024500
H	33.86552000	76.60102100	19.72820800
H	32.74041300	75.50449400	20.54006600
H	31.18229500	76.89993300	22.43727200
H	30.97327400	80.61666900	20.51675100
H	32.36648100	79.04218800	19.02311000
C	33.27359500	84.77003900	21.53140300
C	32.49153900	84.01888600	22.60414600
N	31.90609300	84.97224300	23.55564000
C	30.97333600	84.64678200	24.46832500

N	30.59174900	83.39523400	24.64050400
N	30.42933500	85.62452400	25.23738400
H	34.08700400	85.36599900	21.96486400
H	32.61842700	85.43627600	20.95975000
H	31.67742400	83.44263100	22.15189500
H	33.14395000	83.31219200	23.13271700
H	32.28094300	85.90972000	23.59211000
H	31.13814100	82.58049600	24.33353400
H	29.77340800	83.21064700	25.23487600
H	30.29403700	86.55750800	24.85819600
H	29.64789500	85.31845000	25.81259400
Fe	29.57114300	79.65813900	23.34090500
C	27.57809800	80.70774800	26.62616600
C	26.55492500	81.78323000	26.69501400
N	27.04705800	79.36400700	26.62239200
S	26.91634700	83.27651100	25.69132500
O	28.30118500	83.67735800	26.09850900
O	26.81594300	82.81180000	24.27779400
O	25.89615300	84.29288100	26.04815100
H	28.53881600	80.83044800	27.10174200
H	25.57911700	81.41884400	26.36263900
H	26.47401700	82.14304400	27.72873200
H	26.41140700	79.19625200	27.46360200
H	26.49022200	79.16803000	25.74509800
C	31.80866900	79.97883100	24.73971600
O	31.60447000	80.93060600	23.91961100
O	30.98182200	79.02756800	24.85174000
C	33.11729500	79.93501800	25.51854300
C	32.98841500	79.33427400	26.92075900
H	32.51527000	78.34966200	26.88141200
H	33.97211200	79.23456400	27.39333100
O	25.12504500	78.86054200	24.68580600
H	24.96891600	77.88272900	24.72454600
H	25.25646500	79.05157800	23.72837800
H	32.37555400	79.98778100	27.55191000
H	33.81207700	79.33675300	24.91076700
H	33.53471500	80.94661600	25.55947100
O	28.45241800	80.48825900	24.72446000
H	32.37139000	76.09959200	18.91686300
H	23.55137600	79.06478200	19.46234700
H	33.72130300	84.05619400	20.83365500
H	33.14333600	85.98499400	30.17910600
H	27.99720700	81.58079000	35.84811000
H	30.87671500	88.91357100	27.33328600
H	20.13545300	75.47607300	18.85850600
H	21.62929000	76.42810500	18.98237400
H	26.79115300	74.80629300	20.13106500
H	22.04876800	79.31025700	31.76952800
H	29.01480900	73.73253900	28.73872500
H	25.05276900	75.16335400	30.32032400
H	22.74102300	84.31858000	22.18011500
H	19.10121800	85.73663900	25.27831500
H	22.37368000	75.97823000	23.70523000
H	20.60414200	79.82534300	27.52176200
H	27.87065000	81.07482100	24.19266200
H	27.82088600	78.67959200	26.72540200

^{5}Prod$_{hydro,C1S,B}$

C	31.11521000	87.35099600	27.61370900
C	29.67874400	87.11734000	27.25690300
N	29.01157300	87.93677600	26.36063200
C	28.82326300	86.14351800	27.72262400
C	27.77432400	87.46486400	26.31542800
N	27.60813100	86.38364300	27.11917700
H	31.25351700	88.33095800	28.08637900
H	31.47745100	86.58597700	28.30814200
H	28.96056600	85.32865200	28.41869200
H	26.97166500	87.86141600	25.70718400
H	26.79596300	85.75801100	27.10927500
C	30.33158800	83.59815500	33.01283500
C	29.26487300	83.11087000	32.05774400
C	28.94562500	81.75018100	31.95232800
C	28.51513800	84.01068400	31.28450200
C	27.91681700	81.28957500	31.12555600
C	27.48551700	83.57581600	30.45232200
C	27.17252000	82.21016300	30.36986300
O	26.15438000	81.84224900	29.55125700
H	29.90828600	83.85516700	33.99387500
H	30.83295200	84.49682800	32.63545500
H	29.50066300	81.02706400	32.54808800
H	28.72991600	85.07623900	31.34566000
H	27.66564400	80.23178900	31.07668500
H	26.89871200	84.28023800	29.86934500
H	25.97554800	80.85303000	29.55750300
C	32.77614500	81.60157400	30.00824100
C	33.25544100	82.81392500	29.19115500
C	33.62514600	81.30217700	31.24997600
C	33.12045200	84.15748300	29.91783100
H	31.73175900	81.76258400	30.31196900
H	34.30423000	82.65849400	28.89798300
H	32.67949600	82.85690500	28.25587100
H	33.44280900	84.98715800	29.27735400
H	33.72933800	84.19268100	30.82811300
H	32.07852800	84.34164700	30.20765900
H	33.26890900	80.40152500	31.76307100
H	34.67623500	81.13782500	30.98008900
H	33.59278100	82.12492200	31.97285700
C	25.87200000	75.68752100	29.48786100
C	27.15949700	75.50624000	28.69247900
O	27.23240400	74.79175800	27.67613200
C	25.15014400	76.99691600	29.11912400
C	25.83169000	78.28492300	29.61266800
O	26.74533100	78.22495000	30.46173300
O	25.36456700	79.37282900	29.10964400
H	25.21947400	74.83615300	29.27559500
H	25.01703200	77.05692600	28.03133000
H	24.13693900	76.98844700	29.54026800
N	28.20674100	76.21654700	29.16504600
C	29.43149800	76.41880200	28.40767400
C	29.86188200	77.88715100	28.42550900
C	28.90208600	78.82885900	27.70338800
O	28.10563100	78.41446400	26.84579700
N	29.02510300	80.13925200	27.99383900
H	29.25442800	76.07774200	27.38553800
H	30.01122800	78.23426100	29.45627300
H	30.83435400	77.97558900	27.92312700
H	29.45940600	80.44938000	28.85284600
H	28.42764200	80.79514200	27.48891600
H	27.95988600	76.89569500	29.89115700
C	23.02322700	73.62388900	25.01874200
C	24.38120500	74.13237400	25.44341700
O	24.94846400	75.03513500	24.78092200
N	24.94721000	73.60154500	26.53161100
H	22.30808000	74.45480500	25.09110200
H	22.66824600	72.78467400	25.62374700
H	24.45723900	72.89563600	27.06183600
H	25.81460600	74.00562900	26.93052800
C	26.29667000	75.10034600	20.24774400
C	25.76472000	76.48750200	19.88068000
O	26.14213200	77.06037300	18.86530200
C	26.50750100	74.80554900	21.74149000
C	27.38641900	75.81258100	22.42205500
N	27.37019800	75.99295000	23.79346500
C	28.29249900	76.73096000	21.95089200
C	28.22139800	76.99266300	24.10363200
N	28.80115600	77.46492600	23.00630300
H	25.60403600	74.35742900	19.83066800
H	26.93663700	73.79738200	21.83177700
H	25.54912700	74.75319800	22.27484700
H	28.58967700	76.93431700	20.93282800
H	28.36946600	77.36278600	25.10911600
H	26.71426600	75.53429500	24.43346300
N	24.79246400	77.00600500	20.70094000
C	24.27872400	78.34407200	20.48568400
C	24.83622100	79.36261400	21.48723100
O	24.85296600	79.12035700	22.70417700
H	24.65755200	76.60528000	21.61854600
H	24.52142400	78.61293000	19.45649900
N	25.25718300	80.52210600	20.95340400
C	25.57352200	81.73181000	21.70516000
C	24.81834700	82.87746500	20.99078600
O	24.69273300	82.83731400	19.76272300
C	27.08232300	82.07205000	21.68351500
C	27.99705300	81.02718700	22.30323700
O	27.94661300	80.73955200	23.53330100
O	28.86010800	80.46788900	21.54555000
H	25.13005200	80.69751900	19.96005200
H	25.22731400	81.60626100	22.73017600
H	27.39233500	82.23682500	20.64847200
H	27.21159400	83.00206000	22.24703400
N	24.37098500	83.86941200	21.78171400
C	23.70760800	85.05135800	21.23664500
C	24.67616800	86.16936800	20.80139700
C	23.88489200	87.30443300	20.13625300
C	25.51494900	86.69035900	21.97648300
H	23.02761900	85.42838400	22.00874700
H	25.35118800	85.73214200	20.05252000
H	24.55570100	88.09622400	19.78293000
H	23.31109500	86.94256600	19.27439900
H	23.18001200	87.75906100	20.84468100
H	26.07855900	85.88305300	22.45712100
H	26.23294700	87.44685600	21.63688200
H	24.87492700	87.15289500	22.73905400
H	24.47185800	83.78295800	22.79499000
C	19.99595100	85.75170400	23.45934100
C	20.98409100	86.68990600	24.11594400
C	20.78487800	88.07850500	24.08749200
C	22.13056700	86.19786600	24.75584100
C	21.70081200	88.94901800	24.67966900
C	23.05038200	87.06532300	25.35410400
C	22.83713300	88.44495700	25.31823500
H	20.08050800	84.73650600	23.85967600
H	18.96391900	86.09071600	23.60608200

H	19.89829400	88.48031400	23.60061300
H	22.31466300	85.12726900	24.79308100
H	21.52329900	90.02133800	24.64922400
H	23.92295200	86.64755200	25.84940100
H	23.54490100	89.12287900	25.78875500
C	21.23034700	78.10757900	27.37752000
C	21.57898900	78.33715400	28.84730300
O	21.80912200	77.39885900	29.61363800
C	21.52851000	76.67090100	26.93978100
O	21.44650600	76.51539500	25.52553300
H	20.17524900	78.34710500	27.19677600
H	22.51850500	76.37192100	27.31021000
H	20.80295900	75.98132900	27.37883500
H	22.19089100	77.03279500	25.14912100
N	21.62145600	79.64054500	29.23839400
C	22.17093000	80.04948900	30.52713300
C	21.47849000	81.30244500	31.08398200
C	21.51008000	82.50449400	30.15556100
C	20.32209500	83.16563400	29.81253200
C	22.71696400	82.98188300	29.61760100
C	20.33282800	84.27558000	28.96356100
C	22.73030300	84.08247400	28.75752200
C	21.53796500	84.73512600	28.42894900
H	23.25119900	80.21489800	30.42496800
H	20.43661800	81.06046100	31.32600300
H	21.97319600	81.55246500	32.03314200
H	19.37888700	82.80981900	30.22263200
H	23.65942800	82.49778200	29.86393100
H	19.39962100	84.77596900	28.71674200
H	23.67094600	84.42082700	28.33185600
H	21.55176200	85.58852500	27.75670500
H	21.52935700	80.35677000	28.52857400
C	19.45899000	77.91670700	22.92382700
C	18.69906900	79.22314300	23.21460800
C	19.49189600	80.21583700	24.04279300
C	19.05712300	80.60450400	25.31710000
C	20.68565400	80.76977300	23.55542500
C	19.78974900	81.51410300	26.08521100
C	21.42788700	81.67238100	24.31798800
C	20.98237900	82.04812000	25.59034100
H	19.78954100	77.43062600	23.84768500
H	18.40864300	79.68901500	22.26202000
H	17.76348600	78.98833700	23.73716000
H	18.12850600	80.19400000	25.70888300
H	21.03598700	80.49480400	22.56229700
H	19.42965600	81.81498000	27.06580500
H	22.34979500	82.09446200	23.92967700
H	21.55203200	82.76042000	26.18025600
C	34.11466100	77.13054400	19.53008500
C	32.93245000	77.87109800	20.06298300
N	32.14383100	78.70487300	19.28283100
C	32.36462300	77.92929500	21.31050200
C	31.15805100	79.22140900	20.06097200
N	31.26729400	78.76763400	21.29631300
H	34.88240800	77.80938800	19.13725200
H	34.57168700	76.54052000	20.32899000
H	32.67101100	77.42568100	22.21596300
H	30.39381400	79.90251400	19.71696600
H	32.27222400	78.89185700	18.29842300
C	33.74344900	84.91812900	23.84679000
C	32.48966000	84.25962200	24.41546600
N	31.31270200	85.07984800	24.11423000
C	30.02963900	84.68618800	24.24403600
N	29.70323400	83.40722600	24.39031700
N	29.05833800	85.61816500	24.16622200
H	33.90590800	85.91394900	24.27775700
H	33.67608000	85.01778100	22.75829900
H	32.35387900	83.26747000	23.97648100
H	32.58522900	84.12947200	25.50292700
H	31.45796300	86.07427700	24.00140800
H	30.35300000	82.62786300	24.24216500
H	28.72287200	83.19347400	24.57159000
H	29.23916400	86.53930000	24.56040000
H	28.10834900	85.26965900	24.31481100
Fe	29.88049600	79.30158000	22.89836000
C	25.13061100	80.81864300	25.76139200
C	26.06970500	81.91815200	26.26109200
N	25.47643900	79.50892700	26.39913600
S	25.65322400	83.55876800	25.43630900
O	26.82460500	83.85006400	24.53541900
O	24.39692600	83.33943300	24.65595000
O	25.50560900	84.54589100	26.54593800
H	25.22126000	80.67325700	24.68377300
H	25.94196100	82.10553300	27.33050000
H	27.55232000	81.32816900	25.10388100
H	25.26759200	79.47343000	27.43994300
H	24.94909700	78.74718300	25.91426000
C	31.54707900	80.16987300	24.70908200
O	31.29396200	81.09354200	23.88292800
O	31.01029000	79.01602300	24.58761000
C	32.47892500	80.40683500	25.88709200
C	33.42891300	79.23472400	26.16034300
H	34.10943700	79.07309700	25.31633900
H	34.03747200	79.42845900	27.05036400
O	23.97070500	77.64626800	24.84412700
H	24.35451700	76.74078600	24.79098000
H	24.16333800	78.07689500	23.97821000
H	32.86613600	78.30993500	26.31484900
H	33.03506600	81.33331000	25.71442600
H	31.83743900	80.57559100	26.76350900
O	27.41406400	81.59523500	26.05042000
H	33.83716500	76.43918100	18.72418000
H	23.18647200	78.34372200	20.59944200
H	34.62209200	84.30976600	24.08155600
H	32.77825200	80.71599900	29.35643900
H	31.09874500	82.83437700	33.18420100
H	31.75978200	87.33543000	26.72455200
H	18.82449600	77.21662400	22.36710200
H	20.35086000	78.10868500	22.31435700
H	27.23560500	74.99166600	19.69786500
H	22.03624000	79.20682300	31.20857300
H	30.24442500	75.80894000	28.82420500
H	26.08987100	75.69338300	30.56045500
H	23.11055700	84.73066400	20.37715000
H	20.16247100	85.69075800	22.37511600
H	23.07075900	73.31444000	23.96980700
H	21.80350800	78.80359600	26.75086600
H	24.09002300	81.04971000	25.99241000
H	26.49219100	79.29520100	26.33222200

^{5}Prod$_{hydro,C1R,B}$

C	28.98328500	87.87166600	29.89992100
C	27.84887400	87.24321500	29.14911400
N	26.57522200	87.77656200	29.18592000
C	27.86531800	86.12047800	28.35045700
C	25.84420100	86.98580900	28.42407800
N	26.57393900	85.96663100	27.89009100
H	28.76653100	87.92760600	30.97392300
H	29.90802900	87.29906700	29.76932500
H	28.64923900	85.43422100	28.06776900
H	24.78499000	87.09794400	28.23297900
H	26.24951500	85.22723400	27.26517500
C	27.95038400	83.62791500	34.95042200
C	27.70461900	82.97802900	33.60735800
C	27.44973100	81.60653700	33.49520900
C	27.71832900	83.73730900	32.42694600
C	27.21590600	80.99955400	32.25825400
C	27.48015200	83.15587600	31.18386200
C	27.22399200	81.78220000	31.09882100
O	27.01321100	81.23453900	29.85213600
H	27.17603000	84.36872900	35.18850000
H	28.91327200	84.15396400	34.97608800
H	27.43793500	80.99136700	34.39291300
H	27.90773800	84.80746400	32.47790300
H	27.04490100	79.92901700	32.18107000
H	27.47546500	83.75767800	30.27971900
H	26.47389300	80.38116200	29.90693500
C	33.05032700	82.69397800	29.98419900
C	32.06495000	82.51356800	28.81667800
C	33.78434600	84.04041800	29.99076900
C	30.86746200	83.47138600	28.84319900
H	32.50808100	82.57056000	30.93268900
H	32.60916600	82.63149000	27.86716400
H	31.68955600	81.48106700	28.82918800
H	30.17700000	83.26834900	28.01659500
H	31.18365300	84.51796400	28.75946900
H	30.30185100	83.36969500	29.77759700
H	34.50283000	84.09423000	30.81696500
H	34.34013200	84.19106800	29.05617700
H	33.09123100	84.88137900	30.10342600
C	26.13137800	75.38481400	29.70899200
C	27.30359900	75.27022500	28.74355600
O	27.24828600	74.61662800	27.68571300
C	25.34570000	76.68881600	29.47613300
C	26.06399500	77.95825000	29.95896800
O	27.03932300	77.87100200	30.73339900
O	25.56869200	79.06639900	29.52359600
H	25.47970200	74.52143600	29.55208500
H	25.10114800	76.79730800	28.41184100
H	24.38047600	76.64608300	29.99700300
N	28.39789700	75.98388400	29.09105000
C	29.51957900	76.18089700	28.18164800
C	30.24655500	77.50048200	28.46550600
C	29.34814600	78.72228700	28.30064400
O	28.70310100	78.89830100	27.24709400
N	29.32801900	79.60167400	29.31310600
H	29.14412300	76.16294000	27.15515600
H	30.69498700	77.48117400	29.46595600
H	31.06547700	77.60008300	27.74341000
H	29.69229100	79.32840200	30.21528000
H	28.59462900	80.32081300	29.34084800
H	28.26801000	76.60814800	29.89039700
C	22.67000000	74.74714100	25.55843700
C	24.13417500	74.87075000	25.91579000
O	24.83226600	75.79508800	25.43832900
N	24.66956700	73.95177000	26.72870600
H	22.18235900	75.72034900	25.69192800
H	22.14751100	73.99097000	26.15107400
H	24.08820100	73.24024100	27.14654100
H	25.63704400	74.07874500	27.07178300
C	25.76588900	75.28981000	20.89488700
C	25.37094700	76.68363400	20.39951500
O	25.80260600	77.12715700	19.34203800
C	26.06411200	75.15271400	22.39715700
C	27.09889200	76.12193500	22.88586100
N	27.30602500	76.37279800	24.23254500
C	27.98092900	76.94403900	22.22990000
C	28.26265500	77.32256800	24.34583700
N	28.69621100	77.68719800	23.14742600
H	24.96146400	74.59198800	20.62785800
H	26.38596600	74.11899800	22.58860500
H	25.14967300	75.27823500	22.99124200
H	28.12738400	77.07443200	21.16807400
H	28.60215200	77.73477700	25.28655500
H	26.72672400	75.99279700	24.98311800
N	24.46041800	77.37032900	21.16344600
C	24.11418800	78.74195900	20.83804700
C	25.02418000	79.77115600	21.52446400
O	25.24803000	79.70665600	22.74415300
H	24.28032400	77.06192000	22.10796900
H	24.15616200	78.83561000	19.75184400
N	25.51177300	80.72861100	20.71540500
C	26.14446400	81.96465600	21.16085400
C	25.18786300	83.12019700	20.79468600
O	24.50666700	83.05270800	19.76693600
C	27.48495100	82.23183300	20.45182700
C	28.70549400	81.40896400	20.88233700
O	28.52496100	80.55016200	21.82706200
O	29.78009800	81.64718600	20.30579800
H	25.13235500	80.78602200	19.77645400
H	26.30821200	81.89726400	22.23416800
H	27.36426900	82.12188200	19.36732700
H	27.75600200	83.28186000	20.61462200
N	25.21749000	84.17713400	21.63815000
C	24.50521900	85.42256800	21.37299100
C	25.43420900	86.60262700	21.02296800
C	24.58987000	87.82360000	20.62981500
C	26.38849700	86.94551900	22.17772900
H	23.89916400	85.67933700	22.25061400
H	26.03418300	86.29714900	20.15321800
H	25.22717400	88.67129800	20.35173200
H	23.93780900	87.59959200	19.77717400
H	23.95404300	88.14378600	21.46503500
H	26.99070600	86.07840600	22.47497200
H	27.06916400	87.75712900	21.89003100
H	25.82600300	87.27677900	23.05965400
H	25.77311100	84.09065300	22.48918600
C	19.68240200	86.44768300	23.60751200
C	21.05466200	86.74960400	24.16566600
C	21.47942100	88.07217200	24.35564400
C	21.92806400	85.71385900	24.52754100
C	22.73436400	88.35368800	24.89944200
C	23.18817300	85.98683700	25.06870300
C	23.59316100	87.31187400	25.25867600
H	19.66071700	85.47716900	23.10085400
H	18.92706000	86.41569300	24.40455800

H	20.81844200	88.89004300	24.07572100
H	21.62022600	84.68153300	24.37476800
H	23.04135800	89.38669500	25.04269100
H	23.85833100	85.17147100	25.32994100
H	24.56930900	87.52707900	25.68504500
C	21.01599100	77.88881300	28.31137500
C	21.58987200	78.03550700	29.71962200
O	22.09935000	77.08888900	30.31862700
C	22.12086400	78.22190100	27.30422400
O	21.66912300	77.97110000	25.98155500
H	20.69335100	76.85305600	28.17683900
H	22.41565300	79.27664200	27.42024900
H	23.00555300	77.61157700	27.53890000
H	22.40885600	78.24722400	25.39581600
N	21.56709100	79.30270600	30.21638400
C	22.41857600	79.71012800	31.33192600
C	22.10836000	81.15897000	31.74884000
C	22.16759900	82.13970700	30.59118400
C	20.99416900	82.71788900	30.08571200
C	23.38724200	82.45585700	29.97044500
C	21.03246900	83.58730600	28.99141600
C	23.42871600	83.32229000	28.87729600
C	22.24993800	83.89025800	28.38207200
H	23.47261200	79.61285100	31.03977200
H	21.11338000	81.19817400	32.20856000
H	22.83147600	81.44200800	32.52366500
H	20.04165300	82.49253000	30.56177200
H	24.31183600	82.02233700	30.34218200
H	20.11109400	84.02904700	28.62038100
H	24.38136300	83.55960300	28.41211600
H	22.28421400	84.56846000	27.53382300
H	21.26149300	80.04608800	29.60012200
C	19.83520800	77.53421900	22.73087700
C	19.24979300	78.93268800	22.46165400
C	20.27935100	80.04079000	22.56518400
C	20.79689000	80.41588400	23.81513000
C	20.75422800	80.70115000	21.42301800
C	21.76047100	81.41993800	23.91682300
C	21.72341900	81.70537100	21.51808700
C	22.22784100	82.06767200	22.76953100
H	20.25709600	77.47421600	23.74003200
H	18.79261100	78.95167200	21.46445800
H	18.43867700	79.11829500	23.17842400
H	20.46130000	79.90008100	24.71108300
H	20.35607200	80.42893200	20.44711700
H	22.14646100	81.69968800	24.89439200
H	22.09570700	82.19958400	20.62477900
H	22.98153200	82.84562400	22.84799800
C	32.97496000	75.86639200	19.33132700
C	32.24748700	77.03567300	19.90890700
N	32.15899900	78.26645500	19.27130700
C	31.55063500	77.20431000	21.07734300
C	31.43701000	79.11153300	20.05021200
N	31.05344900	78.49189400	21.15321600
H	34.04144000	76.07790200	19.18014800
H	32.90048600	75.01262300	20.01033900
H	31.37119600	76.48804900	21.86589200
H	31.17269000	80.13390700	19.80854700
H	32.54846800	78.49457500	18.36775500
C	31.84418700	84.45948500	20.18910800
C	31.72356300	83.91174600	21.60711300
N	30.91646000	84.82256100	22.43134900
C	30.31810800	84.49748900	23.59050700
N	30.41387200	83.28877200	24.12011900
N	29.63978600	85.46327100	24.26721700
H	32.28209500	85.46632700	20.17283700
H	30.86466800	84.48448600	19.70317000
H	31.22632400	82.94047800	21.56799900
H	32.71430100	83.77809400	22.06245700
H	31.00369300	85.81193100	22.23941300
H	30.93975300	82.51786200	23.68724200
H	29.73266100	83.05646100	24.84820400
H	29.17105600	86.16268200	23.70488200
H	29.06448600	85.10339500	25.03117900
Fe	29.86753500	79.42590300	22.71218900
C	26.02601700	81.03331900	26.70525500
C	26.71624700	81.37902600	25.38350200
N	25.91427800	79.55044100	26.86732000
S	26.94810600	83.23009300	25.31658300
O	28.25839600	83.48932400	26.01672100
O	27.01497100	83.60107500	23.87329600
O	25.77663600	83.80939100	26.04105100
H	25.02436500	81.46081500	26.74121200
H	28.40807700	80.60747200	26.03714200
H	26.08855400	81.13316300	24.52255100
H	25.56794300	79.31977900	27.84043400
H	25.29316900	79.12172500	26.13734400
C	31.86564500	80.11087200	24.18219100
O	31.59265000	80.92310300	23.24192000
O	31.12900400	79.09582000	24.38677800
C	33.09765400	80.35878100	25.03863700
C	33.29220300	79.37955600	26.19480800
H	33.33350600	78.34822500	25.83141300
H	34.22233100	79.59604900	26.73185400
O	24.21126700	78.43237600	24.90940200
H	24.43194300	77.47261500	24.93969000
H	24.52697800	78.80453300	24.04464000
H	32.46529000	79.45060200	26.90863500
H	33.96065700	80.33857900	24.35939500
H	33.03933300	81.39247000	25.40389800
O	27.93120800	80.71152900	25.19203500
H	32.55642500	75.55866700	18.36437700
H	23.09021900	78.94709000	21.16970300
H	32.49401300	83.80503500	19.60069900
H	33.79274000	81.88432600	29.94897100
H	27.95611000	82.88649400	35.75645200
H	29.16753000	88.89827800	29.55866100
H	19.06053100	76.76343600	22.63948500
H	20.63080000	77.29916500	22.01293400
H	26.63918800	75.00483700	20.30212400
H	22.24625700	79.02624500	32.16809400
H	30.23628700	75.35469300	28.27302200
H	26.48633600	75.36579900	30.74378300
H	23.82721800	85.22194500	20.53976900
H	19.36287300	87.21268700	22.89113900
H	22.58434700	74.47892000	24.49881500
H	20.15420600	78.54613500	28.14452100
H	26.58587600	81.41285200	27.56446800
H	26.86120100	79.14022000	26.81806700

[5]Prod$_{hydro,C2S,B}$

C	29.52221000	89.81673900	23.07394500
C	28.63497700	88.99922300	23.96013000
N	28.82196300	87.63653100	24.12901700
C	27.55888700	89.40501100	24.71087800
C	27.87437500	87.23598000	24.96750700
N	27.08775100	88.27234500	25.34318000
H	29.47745900	89.47251300	22.03300200
H	29.22742700	90.87068400	23.09076100
H	27.09747700	90.37163200	24.84491600
H	27.73897100	86.22822100	25.34243800
H	26.31524600	88.21452300	25.99872700
C	29.75882400	82.01238000	35.35643200
C	29.39091300	81.59989600	33.94892400
C	28.59806600	80.47084300	33.70600800
C	29.81051500	82.34977400	32.84098100
C	28.22735500	80.09620100	32.41356000
C	29.44533700	81.99884600	31.54264700
C	28.64613500	80.87168900	31.32409200
O	28.33766800	80.53073200	30.03111400
H	29.03923400	82.73323400	35.76901700
H	30.74582500	82.48793700	35.39177800
H	28.26492000	79.86287500	34.54520800
H	30.43207900	83.22977900	32.99564800
H	27.63416600	79.20241300	32.23734200
H	29.76537400	82.59057400	30.68979700
H	27.51283200	79.94617600	30.00350100
C	32.81653700	85.12463100	29.49028700
C	31.58102800	84.70815400	28.67424000
C	33.26378900	86.57417200	29.26421200
C	30.28753900	85.44010400	29.05383100
H	32.61123600	84.96930300	30.55971200
H	31.78585300	84.85957600	27.60446000
H	31.42580300	83.62725100	28.80086600
H	29.44355900	85.07238000	28.46085400
H	30.36895500	86.52094500	28.88590600
H	30.04738700	85.28944500	30.11477100
H	34.17050900	86.80316800	29.83680300
H	33.48426300	86.75659400	28.20455900
H	32.49277600	87.29055900	29.56942900
C	25.10267000	75.55525300	29.18469100
C	26.29416900	75.05607900	28.37200700
O	26.19418000	74.69607200	27.17854000
C	24.97045400	77.09076900	29.16889100
C	26.11047700	77.85533000	29.86827900
O	26.83942800	77.26675200	30.69467700
O	26.20484400	79.09570400	29.54422800
H	24.19398700	75.11361200	28.76648400
H	24.86468200	77.45155500	28.14143200
H	24.03495100	77.36156700	29.67619300
N	27.45429100	75.04936400	29.05799400
C	28.76119600	74.80180500	28.46346600
C	29.81183700	75.75587300	29.04066400
C	29.46780100	77.22035800	28.77182400
O	28.86358700	77.53566800	27.73700400
N	29.89510800	78.12345100	29.67273100
H	28.68261200	74.93131100	27.38281100
H	29.93599000	75.58415400	30.11704600
H	30.78122100	75.54000100	28.57314900
H	30.16479200	77.80933400	30.59472200
H	29.49401300	79.06746000	29.63273800
H	27.41348700	75.58168800	29.93329200
C	21.67484100	74.72342700	24.61177100
C	23.12854400	74.81147600	25.01254400
O	23.94005800	75.47383800	24.33430600
N	23.51284400	74.18597200	26.13785600
H	21.22836800	75.69727600	24.84501100
H	21.12713200	73.93498000	25.13624000
H	22.84025200	73.69863100	26.71128000
H	24.46835300	74.31767100	26.49717900
C	26.03591000	74.99013000	21.07518000
C	25.52302000	76.41717300	20.86267200
O	25.95724100	77.11804200	19.95107300
C	26.44188500	74.61449600	22.51362800
C	27.37402400	75.60435500	23.14390400
N	27.62196400	75.63878200	24.50913300
C	28.10944900	76.64261000	22.62561900
C	28.46607500	76.66935200	24.76137900
N	28.77747800	77.30092900	23.63979000
H	25.26516900	74.28602100	20.73512500
H	26.90292000	73.61636100	22.48404600
H	25.55236600	74.52169700	23.14518800
H	28.16788400	76.98325800	21.60279400
H	28.81406200	76.92723000	25.75167100
H	27.17744600	75.07965100	25.23570400
N	24.54039600	76.86530200	21.69979200
C	24.05685500	78.22260600	21.54654000
C	25.02502100	79.27335800	22.10678600
O	25.48475800	79.14035500	23.26356400
H	24.25023300	76.33242900	22.51652400
H	23.85878300	78.41746300	20.48961200
N	25.29625600	80.32273400	21.32972700
C	25.99302300	81.53747800	21.74800900
C	24.99361400	82.69889300	21.57777800
O	24.17145000	82.68056800	20.65435100
C	27.25758900	81.82214200	20.90486000
C	28.56860200	81.22719000	21.43809900
O	28.47497400	80.17960100	22.16614700
O	29.63109600	81.79974900	21.10644400
H	24.78454200	80.41886400	20.45875300
H	26.27580500	81.43114400	22.79344100
H	27.10791900	81.46183800	19.87906300
H	27.40965500	82.90286500	20.83073600
N	25.12120400	83.69673900	22.47400700
C	24.25810700	84.87256200	22.43541400
C	24.65599100	85.91918300	21.37491700
C	23.59635700	87.02830500	21.32008000
C	26.05236700	86.49538000	21.63947700
H	24.28268000	85.32400100	23.43383200
H	24.66278100	85.40103300	20.40709600
H	23.84165600	87.76944500	20.55044700
H	22.60344300	86.62319600	21.09059800
H	23.53034900	87.55841100	22.28006100
H	26.80679200	85.70345000	21.70551400
H	26.35057600	87.18275700	20.83802900
H	26.07665800	87.05480100	22.58311700
H	25.80540400	83.59665400	23.23059600
C	22.23209000	89.41918700	26.00222900
C	23.22005000	88.39958100	26.52254000
C	24.12658300	88.72391600	27.54114000
C	23.23719500	87.09185100	26.01192000
C	25.02041400	87.76880000	28.04218500
C	24.13123500	86.13725000	26.50006900
C	25.02900100	86.47223800	27.52026100
H	21.22427400	89.23210700	26.39586900
H	22.51291600	90.43669100	26.29377900

H	24.12659900	89.73072600	27.95414400
H	22.54082800	86.82199600	25.22009700
H	25.70817800	88.04025700	28.83946600
H	24.15386700	85.12931300	26.09691100
H	25.72457700	85.72235600	27.88369800
C	20.44182800	78.11932300	27.69075700
C	20.37076400	78.27673200	29.20735700
O	21.02760900	77.55595500	29.95690500
C	21.90541200	78.07347800	27.25756900
O	21.98087100	77.84626600	25.85612600
H	19.95756000	77.17437400	27.41299400
H	22.39770700	79.01866500	27.53076700
H	22.40092900	77.27045600	27.81639300
H	22.93585700	77.88922400	25.62822100
N	19.54091200	79.26276700	29.66181800
C	19.55889500	79.71090200	31.04843100
C	19.92686700	81.20242400	31.18509000
C	21.16916200	81.62410000	30.42071600
C	21.13313400	82.74783800	29.58462800
C	22.37054800	80.90568000	30.51457900
C	22.26024600	83.14371100	28.86012500
C	23.49746900	81.29104700	29.78526500
C	23.44638400	82.41342100	28.95155800
H	20.28211000	79.07097200	31.55786500
H	19.07895600	81.81530200	30.84955000
H	20.03910800	81.41266000	32.25875000
H	20.21057900	83.32002700	29.49980700
H	22.42543900	80.02234800	31.14626400
H	22.21205500	84.02099000	28.21978500
H	24.41002200	80.70510600	29.86113600
H	24.31532900	82.71132200	28.37045900
H	19.14331200	79.88884400	28.97486400
C	18.87282800	77.60031200	19.52659200
C	18.36301000	78.79215200	20.35728200
C	19.46277600	79.47835800	21.14172100
C	19.78851900	79.06078600	22.43968200
C	20.20845400	80.52104500	20.57337400
C	20.82252400	79.66779700	23.15798300
C	21.24616900	81.13473000	21.27823700
C	21.54997600	80.71017500	22.57517700
H	19.33140400	76.84136600	20.17100400
H	17.88267400	79.51645100	19.68683700
H	17.58377000	78.44183100	21.04651300
H	19.22078100	78.25251500	22.89766200
H	19.97008200	80.85821100	19.56608100
H	21.06373300	79.31638100	24.15867000
H	21.82665500	81.93271000	20.82419500
H	22.35028700	81.19405400	23.12994000
C	34.41134300	77.01358100	21.34078500
C	33.21578900	77.87221400	21.59308100
N	32.86265000	78.94028400	20.77922900
C	32.25445100	77.85322800	22.57082100
C	31.73490400	79.51250100	21.27778600
N	31.34382400	78.87080900	22.36358600
H	35.34555000	77.58998600	21.36123800
H	34.48229400	76.24281700	22.11317100
H	32.15624500	77.18479400	23.41339000
H	31.22944000	80.37597200	20.86165900
H	33.35664200	79.24482900	19.95283500
C	30.27483200	86.44835900	20.34735700
C	30.49732400	85.18739600	21.17730500
N	30.26389900	85.46721400	22.59365000
C	30.42437800	84.56726000	23.57333000
N	30.72188300	83.29780600	23.30581900
N	30.37187400	84.99616600	24.86150100
H	30.94887000	87.25339900	20.66110000
H	29.24280300	86.80477400	20.44184100
H	29.84023300	84.38001800	20.83076800
H	31.52785100	84.82770400	21.05784800
H	29.81383300	86.34368300	22.88053200
H	30.43137400	82.86883400	22.42219700
H	30.82595000	82.62860400	24.07328800
H	30.16302600	85.98676500	24.95123700
H	29.80508700	84.43257200	25.50533300
Fe	29.58938300	79.32304700	23.61203200
C	26.95172200	80.42240100	25.92560000
C	27.77378200	81.55956100	26.52327500
N	26.82969300	79.25724600	26.87919900
S	27.16585100	83.17582600	25.92676700
O	28.15139600	84.16072900	26.47878300
O	27.20218700	83.11780600	24.42904000
O	25.79288700	83.30159100	26.47898100
H	26.85924900	79.71161300	24.08683200
H	27.71377100	81.59616500	27.61523200
H	28.81555400	81.46154000	26.20991200
H	26.41859600	79.45869500	27.82560000
H	26.21169400	78.54684800	26.42960600
C	31.12038800	80.01837500	25.55497500
O	30.66846600	80.94469600	24.80699900
O	30.73631000	78.82223700	25.37762300
C	32.09398400	80.36717700	26.66837000
C	32.89214000	79.18053000	27.20904900
H	33.48192500	78.70893400	26.41538300
H	33.57941900	79.50744100	27.99735800
O	24.81516700	77.79332000	25.48105800
H	24.79675300	76.83552300	25.26486500
H	24.96052400	78.22096500	24.59877000
H	32.22462400	78.42243200	27.62664700
H	32.75401500	81.15808500	26.29386700
H	31.50511500	80.82896800	27.47406500
O	27.54895300	79.93887900	24.76095800
H	34.35625400	76.50485700	20.36944100
H	23.11640100	78.32675400	22.09642700
H	30.46287000	86.23809800	19.28993300
H	33.64990300	84.45325300	29.24031600
H	29.77751400	81.15187800	36.03472600
H	30.56985100	89.75392400	23.39289400
H	18.05335800	77.12676300	18.97290600
H	19.63064400	77.92217000	18.80321400
H	26.88534900	74.88614300	20.39486700
H	18.58140100	79.53498200	31.51746400
H	29.07345700	73.76618000	28.65066400
H	25.19799800	75.21878700	30.22142500
H	23.23344200	84.53644900	22.24050200
H	22.15969100	89.38832900	24.90878900
H	21.60426100	74.56259900	23.53287900
H	19.91726300	78.92495900	27.16385600
H	25.92418100	80.74727000	25.73500400
H	27.74821700	78.81342200	27.07812800

^{5}Prod$_{hydro,C2R,B}$

C	29.17612300	89.47296700	27.47050000
C	28.21761700	88.50019900	26.85694700
N	28.57674200	87.70582200	25.77719200
C	26.91586700	88.23539200	27.21102100
C	27.49876100	86.99439700	25.49215500
N	26.46346600	87.28160400	26.31996500
H	29.48111000	90.24263600	26.75058700
H	28.72702600	89.97548700	28.33314200
H	26.28621700	88.63598100	27.99122500
H	27.42529500	86.22669400	24.73558700
H	25.64451500	86.68180800	26.40792200
C	29.74663700	82.10496700	35.08241000
C	29.21489700	81.69829800	33.72650500
C	28.57705500	80.46608900	33.53511300
C	29.32021300	82.55495200	32.62100100
C	28.05602900	80.09034600	32.29521500
C	28.80090500	82.20454800	31.37594200
C	28.16076600	80.97068400	31.21055800
O	27.68607800	80.64728000	29.96394800
H	29.01540800	82.70609300	35.64046200
H	30.65719900	82.70867700	34.99332300
H	28.48969700	79.77609200	34.37236700
H	29.81078900	83.51945300	32.73698400
H	27.59043700	79.11915400	32.14863600
H	28.87714500	82.87687600	30.52596100
H	26.94364700	79.95520600	30.00597200
C	32.03509700	85.09708700	29.38950000
C	32.58177900	83.72775900	29.82763300
C	32.93240200	85.85358400	28.40216800
C	32.72521900	82.70945000	28.68930300
H	31.04230800	84.96286400	28.93906600
H	33.55749700	83.86718900	30.31606800
H	31.91284000	83.31098400	30.59234300
H	33.05500500	81.73627600	29.07155900
H	33.45847600	83.03520000	27.94216800
H	31.76830800	82.55812100	28.17395100
H	32.52712400	86.85022200	28.18950700
H	33.94523300	85.98615000	28.80448800
H	33.01962500	85.32360300	27.44649600
C	25.33587700	75.25616500	29.42828800
C	26.46624200	74.82358200	28.50362900
O	26.26800700	74.23475200	27.42603000
C	24.96977500	76.74017500	29.22948900
C	25.95614700	77.74269000	29.84996100
O	26.89456500	77.34021400	30.57022700
O	25.71539900	78.98101100	29.58567200
H	24.46793700	74.62828400	29.21005400
H	24.86956500	76.96420700	28.15955300
H	23.98345200	76.93676600	29.66779100
N	27.70558900	75.17039000	28.91930400
C	28.85630700	75.11463000	28.02504000
C	29.83876600	76.25464200	28.31338900
C	29.18401800	77.62746000	28.18678300
O	28.38661400	77.86060100	27.26366200
N	29.54317500	78.55435700	29.09525500
H	28.49238100	75.17629100	26.99637900
H	30.28901200	76.13482100	29.30638900
H	30.65681800	76.20515900	27.58374800
H	30.00605000	78.25598700	29.94241500
H	28.97745400	79.40327800	29.19134600
H	27.71640600	75.81054400	29.71829500
C	22.07913600	75.84203600	25.09554800
C	23.49527900	75.50693100	25.51080900
O	24.45410900	76.21248000	25.12321600
N	23.69414800	74.42475400	26.27365200
H	21.87950800	76.89935300	25.30804200
H	21.32832700	75.22093800	25.59221400
H	22.90677700	73.89976000	26.62492600
H	24.63194300	74.25040400	26.67523800
C	25.81382600	75.22043800	20.41027700
C	25.73254200	76.61111500	19.77717200
O	26.54306500	76.98449400	18.93628400
C	25.83449100	75.19338400	21.95057400
C	26.92977800	76.01678600	22.55880800
N	27.00447300	76.23219700	23.92384900
C	27.97992800	76.72329900	22.02636800
C	28.04607600	77.05612700	24.16868700
N	28.66388900	77.37283900	23.03742300
H	24.96148800	74.62339500	20.06142800
H	25.92435400	74.14543300	22.26981300
H	24.87275600	75.53573400	22.35841900
H	28.26514000	76.83384600	20.99122500
H	28.31957100	77.39130900	25.16039900
H	26.29014300	75.95678900	24.60200300
N	24.66526600	77.38315100	20.17300900
C	24.66096000	78.80561200	19.90246500
C	25.14707100	79.60593600	21.12007500
O	25.17829000	79.08240300	22.23831600
H	24.21329300	77.12639700	21.04151700
H	25.30527000	78.97960100	19.03827300
N	25.51126700	80.87483800	20.84977000
C	25.82381700	81.88862100	21.84489300
C	25.03225300	83.15660400	21.43501100
O	24.51568100	83.22233900	20.31246000
C	27.34017200	82.18767900	21.90284900
C	28.16502800	80.98846900	22.33846700
O	28.03335100	80.50655800	23.50153000
O	29.01240400	80.48814200	21.52943200
H	25.24336400	81.27891200	19.95498800
H	25.49185800	81.53360200	22.82333100
H	27.68387300	82.52199500	20.92003500
H	27.50774100	82.98285500	22.63613600
N	24.96046300	84.13237100	22.35744800
C	24.21055600	85.36349700	22.10719600
C	24.93554100	86.38948900	21.21325900
C	23.99230300	87.56535200	20.92064900
C	26.25040100	86.87132700	21.83800600
H	23.98130800	85.80429900	23.08298400
H	25.16115700	85.88289700	20.26589700
H	24.47665600	88.30503200	20.27241000
H	23.07757700	87.22787300	20.41884100
H	23.69542800	88.07439200	21.84673000
H	26.92305600	86.03292600	22.05235900
H	26.77684900	87.55769700	21.16404600
H	26.06913400	87.40629600	22.77922700
H	25.45581000	84.02490300	23.24676200
C	18.47764200	86.79169600	24.51475600
C	19.92787900	87.16379700	24.72598300
C	20.37760300	88.47043500	24.49373400
C	20.85404200	86.21452100	25.18061700
C	21.71090800	88.82124400	24.71543400
C	22.18933000	86.55535000	25.40588100
C	22.62023500	87.86523500	25.17317700
H	17.90798500	86.85937300	25.45167400
H	17.99355200	87.45700700	23.79173300

H	19.67589800	89.22004200	24.13356400
H	20.52500500	85.19252500	25.35807900
H	22.03883900	89.84099200	24.52885100
H	22.89197700	85.80238300	25.75351300
H	23.65890600	88.13794700	25.34347400
C	21.11831500	79.08748900	28.06946500
C	21.55897900	78.82749100	29.50806200
O	21.73422500	77.68587600	29.93379000
C	22.36706700	79.13993300	27.18367500
O	21.99166800	79.17848300	25.81615700
H	20.48374200	78.25564000	27.75178800
H	22.96653900	80.02487100	27.45247400
H	22.98303600	78.25457500	27.39780200
H	22.83392400	79.22713600	25.30963800
N	21.81642500	79.93992800	30.24964100
C	22.59396400	79.86986100	31.48458800
C	22.58819900	81.22572800	32.21195600
C	23.07345300	82.36430100	31.33411200
C	22.17687900	83.32722600	30.84915700
C	24.41775500	82.44256800	30.93551200
C	22.60469500	84.33666600	29.98149200
C	24.84825000	83.44925400	30.07051000
C	23.94070000	84.39746900	29.58546000
H	23.62424400	79.57062300	31.24951000
H	21.57412900	81.44174100	32.56973000
H	23.22770000	81.12808400	33.09813100
H	21.13355300	83.28631700	31.15690500
H	25.13087600	81.70556500	31.29600300
H	21.89219200	85.07003000	29.61237900
H	25.89048000	83.49549000	29.76616100
H	24.27537300	85.15781400	28.88800200
H	21.80277800	80.83815800	29.78198900
C	20.63572000	76.73301100	19.91917800
C	19.78553400	78.01658700	19.89240500
C	20.42129600	79.16649400	20.64793700
C	20.42121500	79.18776800	22.05065800
C	21.04819200	80.22150000	19.96948800
C	21.03114800	80.22498300	22.75843400
C	21.66480600	81.26458000	20.66803000
C	21.65766200	81.26464100	22.06486500
H	20.78297500	76.38409600	20.94848000
H	19.61386400	78.31363700	18.85038000
H	18.79774000	77.79633200	20.31879400
H	19.93120600	78.38435800	22.59785000
H	21.04439000	80.23113100	18.88076800
H	21.02181800	80.21350200	23.84513300
H	22.15875000	82.06981200	20.13139500
H	22.13191600	82.07703600	22.60971800
C	33.75374800	76.49514300	19.38774500
C	32.66764800	77.35939600	19.93858200
N	31.89064000	78.19681100	19.15048100
C	32.19254400	77.54649500	21.21185800
C	30.99987400	78.83925400	19.94965000
N	31.16108400	78.46424500	21.20495600
H	34.53804100	77.08379200	18.89463100
H	34.22328200	75.93111100	20.19813300
H	32.52000500	77.08586100	22.13263300
H	30.26620600	79.55316800	19.60569500
H	31.96105900	78.30142100	18.14830300
C	33.37175700	84.78062500	23.10107900
C	32.08629800	84.04372100	23.46640900
N	31.46204600	84.67543100	24.63382400
C	30.42405500	84.15285500	25.33155500
N	30.10962400	82.86631800	25.22128700
N	29.76098000	84.94444200	26.18730700
H	34.09062000	84.75125900	23.92671400
H	33.17587200	85.83087900	22.85089500
H	31.38977100	84.04414100	22.61574300
H	32.30531800	82.99839800	23.69892700
H	31.57438700	85.67769400	24.71688300
H	30.64385300	82.19788700	24.65612400
H	29.27622400	82.49860600	25.68523000
H	29.74292400	85.95379600	26.02716000
H	28.88861300	84.58516000	26.59996800
Fe	29.88759500	79.11890700	22.85096200
C	26.79378900	81.21160200	26.88373500
C	25.59692000	82.09514900	26.51208900
N	26.34041400	79.77188800	27.01640000
S	26.14306900	83.80110900	26.14511300
O	27.22565100	84.07177300	27.13868100
O	26.63646700	83.76196700	24.72792100
O	24.94782700	84.67581700	26.30900200
H	27.17289900	81.49832400	27.86403300
H	25.06162700	81.72697600	25.63192800
H	24.90306700	82.15675600	27.35558200
H	25.84804200	79.57025100	27.92335100
H	25.75044100	79.46494300	26.20711300
C	31.71265800	79.75322600	24.57476400
O	31.39109900	80.72604600	23.82420300
O	31.14113700	78.62204900	24.44719000
C	32.81287900	79.93677400	25.60899600
C	32.92402600	78.81708800	26.64394100
H	33.05289200	77.84651800	26.15574400
H	33.78041800	78.99041500	27.30535500
O	24.62855500	78.95842500	24.90293300
H	24.54191000	77.97355600	24.92084900
H	24.89802000	79.16192100	23.97618400
H	32.02265500	78.76638500	27.26340000
H	33.75311200	80.03861300	25.04807300
H	32.65325600	80.90678100	26.09491500
O	27.87398300	81.24924800	26.01260100
H	33.37188100	75.77047200	18.65722700
H	23.64551500	79.14130200	19.65932000
H	33.83246100	84.31261400	22.22574400
H	31.88735300	85.71721800	30.28424100
H	29.98319000	81.23042100	35.69862400
H	30.08965000	88.96918300	27.81092900
H	20.14885100	75.92693600	19.35763400
H	21.62606100	76.90368100	19.48106300
H	26.72006700	74.75946100	20.00925800
H	22.16073400	79.09064200	32.11790900
H	29.38049200	74.15586000	28.12870800
H	25.61934600	75.09829800	30.47354900
H	23.26168400	85.08716800	21.63540700
H	18.37599100	85.76381800	24.14927400
H	21.98750400	75.70529400	24.01180200
H	20.54837500	80.01844300	27.96647000
H	27.64848700	81.07464900	25.06062900
H	27.19389600	79.16894900	27.05214900

Model B2 structures:

$^{5}Re_{B2}$

C	28.23655800	88.29615300	29.41786600
C	27.15433800	87.58360700	28.66554400
N	25.87144400	88.09161900	28.59135300
C	27.23009100	86.39556900	27.97334600
C	25.19309300	87.22160900	27.86929100
N	25.96601400	86.17352600	27.46650700
H	27.96997200	88.42028800	30.47476200
H	29.18087000	87.74301600	29.36875600
H	28.03745400	85.70047300	27.80033900
H	24.14405300	87.29500300	27.61431600
H	25.68454700	85.38197400	26.88443200
C	27.90699900	83.03245800	34.86436200
C	27.72264500	82.42865100	33.49021800
C	27.52657200	81.05178400	33.32002400
C	27.76161700	83.22745400	32.33863400
C	27.38498400	80.47931700	32.05472200
C	27.61306500	82.67849700	31.06497600
C	27.42411400	81.29870700	30.91872100
O	27.29750800	80.78007500	29.65400900
H	28.96959400	83.17219800	35.10713200
H	27.47866700	82.39254300	35.64360400
H	27.48868800	80.40655100	34.19568700
H	27.89897700	84.30236000	32.43687100
H	27.25608200	79.40700400	31.93272700
H	27.62513400	83.30956700	30.18095400
H	26.72249800	79.94428500	29.67436200
C	32.89438800	82.71394000	29.25229500
C	31.69914400	82.70799400	28.28385200
C	33.69828400	84.01992700	29.25943600
C	30.59109400	83.70769300	28.63670100
H	32.53259200	82.50391800	30.26904300
H	32.05934500	82.91217200	27.26399300
H	31.26846100	81.69753300	28.25995000
H	29.74992200	83.62079900	27.93953200
H	30.94921700	84.74325700	28.59877200
H	30.20354800	83.52419300	29.64611900
H	34.56213100	83.94868300	29.93057600
H	34.07549200	84.25642000	28.25585400
H	33.09235800	84.86937800	29.59366800
C	22.29752000	74.87569500	25.42227700
C	23.80473800	74.86702300	25.52063700
O	24.48381000	75.74412600	24.93684400
N	24.38331300	73.91041700	26.25716900
H	21.92582500	75.73063800	26.00307300
H	21.83853700	73.95719800	25.79914700
H	23.80700500	73.24595800	26.75329500
H	25.37786200	73.99096300	26.53384400
C	26.35953500	75.45218000	20.09050700
C	26.37954600	76.74090700	19.26292600
O	27.19386000	76.89154500	18.35850300
C	26.00266900	75.55769000	21.58244400
C	26.91824300	76.43933700	22.38106000
N	26.91046100	76.42579900	23.76691600
C	27.83928500	77.39727500	22.03725600
C	27.77985600	77.35400800	24.21112500
N	28.36228700	77.96272600	23.18450300
H	25.64653000	74.76926600	19.61011500
H	26.02136100	74.54447900	22.00501300
H	24.96185600	75.89306000	21.70800600
H	28.16032300	77.72292500	21.06049800
H	27.99296100	77.54406400	25.25440900
H	26.23649200	75.92158800	24.35343800
N	25.40194800	77.66186200	19.54644500
C	25.49061000	79.00700700	19.00827100
C	25.76092800	80.00073300	20.14802100
O	25.37136700	79.75263300	21.29208600
H	24.97883900	77.63378700	20.46709300
H	26.28341400	79.01375800	18.25741500
N	26.44548300	81.11152500	19.78596400
C	26.61886200	82.25829800	20.66722700
C	25.68396400	83.38371600	20.17301300
O	25.42953500	83.48691400	18.97104800
C	28.06391200	82.77801000	20.64071900
C	29.15943600	81.84238200	21.16156300
O	28.77120400	80.86353100	21.91890500
O	30.33275800	82.09896100	20.86070400
H	26.47880700	81.34019600	18.79785500
H	26.37016600	81.93672900	21.67676000
H	28.34059500	83.08990300	19.62745600
H	28.10989700	83.67640600	21.26887600
N	25.22771900	84.21985500	21.13288600
C	24.42797300	85.39977300	20.82367300
C	25.25566800	86.68836800	20.63814500
C	24.33714300	87.83362000	20.18848600
C	26.02291700	87.06589000	21.91370900
H	23.69929500	85.53730200	21.63146200
H	25.98135300	86.48847100	19.83686200
H	24.90898800	88.75351600	20.01830900
H	23.81576500	87.58556400	19.25618100
H	23.57889400	88.05002000	20.95203700
H	26.66423300	86.24545800	22.25698000
H	26.65463400	87.94668000	21.74308600
H	25.32745200	87.30658800	22.72764000
H	25.53641800	84.06633900	22.09066200
C	19.25031300	85.95919500	22.98419700
C	20.53999000	86.48737100	23.57058000
C	20.74100200	87.86529800	23.73753300
C	21.55655800	85.61396000	23.98081800
C	21.91816100	88.35663000	24.30375200
C	22.73940500	86.09824400	24.54786500
C	22.92212400	87.47446700	24.71171000
H	18.87912200	86.60572700	22.18085800
H	19.38000000	84.95170800	22.57541000
H	19.96585200	88.55991500	23.41989500
H	21.42346200	84.54153400	23.85378100
H	22.05254500	89.42849900	24.42561600
H	23.51798800	85.40259800	24.85079400
H	23.83902200	87.85459300	25.15427500
C	18.89060600	78.49814000	25.10523500
C	19.05589100	79.98305900	24.73801900
C	20.44011400	80.34005800	24.23052400
C	21.23611600	81.28165800	24.89883200
C	20.95680400	79.74386700	23.06943200
C	22.49962800	81.63741700	24.41729900
C	22.22267400	80.08297000	22.58735100
C	22.99646300	81.04031700	23.25444800
H	19.63518400	78.19670300	25.84876300
H	18.31118200	80.25215100	23.97518600
H	18.82901300	80.60052100	25.61567300
H	20.86057800	81.74959300	25.80634600
H	20.35618000	79.01426600	22.53040600
H	23.09200200	82.38198800	24.94388500
H	22.61246200	79.62212300	21.68409800

H	23.96406800	81.31659400	22.84745000
C	32.85971700	75.92164700	20.42893100
C	32.13305200	77.14977700	20.86875100
N	32.21664500	78.36355900	20.19819700
C	31.28239100	77.39462900	21.91466400
C	31.43533800	79.27033000	20.83771000
N	30.85270500	78.70827500	21.88276200
H	33.94743900	76.06892800	20.41305700
H	32.64717100	75.09974200	21.11799200
H	30.94862100	76.71787900	22.68646400
H	31.30948900	80.30815400	20.55267000
H	32.74778500	78.54074300	19.35756100
C	32.72400300	84.40133400	21.19550200
C	32.26927300	83.92979100	22.57282600
N	31.24159900	84.83762600	23.09880700
C	30.33528700	84.53518800	24.04467900
N	30.26427300	83.33118200	24.59693800
N	29.50266300	85.50757700	24.49032300
H	33.09882900	85.43318400	21.21791400
H	31.89701600	84.32707600	20.48453200
H	31.83571400	82.93335800	22.47600800
H	33.11425900	83.88304700	23.27437200
H	31.35629400	85.82026200	22.88860900
H	30.81864600	82.53154000	24.28321000
H	29.42602100	83.12530100	25.14733300
H	29.29853000	86.27735800	23.86794400
H	28.71302400	85.17434500	25.05109100
Fe	29.49836000	79.75157900	23.35835600
C	25.70021100	81.41972700	26.86040600
C	26.03801800	81.86859300	25.45219900
N	25.71357200	79.92972700	26.96400400
S	26.15290900	83.66503200	25.30570700
O	27.37474600	84.05139000	26.09729700
O	26.29914200	83.95695300	23.85553300
O	24.89835400	84.18834000	25.92438600
H	24.70325600	81.76327500	27.14627600
H	27.03071100	81.47461400	25.13986700
H	25.33899200	81.49964700	24.69568100
H	25.43666400	79.60684700	27.93390900
H	25.06035500	79.49305000	26.28211600
C	31.68387100	79.88865900	24.65193600
O	31.30178600	80.82596900	23.86000300
O	30.95946100	78.86806900	24.78667900
C	33.01436200	80.03434300	25.36402700
C	33.27402300	78.98944200	26.44818700
H	33.24395600	77.97758000	26.03276500
H	34.25776700	79.14596700	26.90375300
O	23.99326200	78.44203700	25.11942800
H	24.12340300	77.48699200	24.89651600
H	23.71550600	78.90136300	24.30512200
H	32.51798200	79.05372400	27.23694000
H	33.79136200	79.99570300	24.58776500
H	33.05535000	81.04974800	25.77602800
O	28.55772300	80.41675000	24.69644500
H	32.55245800	75.60043400	19.42529100
H	24.54889300	79.28944000	18.51726400
H	33.53846400	83.76232200	20.84032600
H	33.56626900	81.88416000	28.99059600
H	27.42924700	84.01619900	34.93883300
H	28.40864400	89.30118600	29.01235300
H	17.88816900	78.31110600	25.50799000
H	19.01635800	77.85605400	24.22508300
H	27.35153100	75.00878000	19.96866500
H	23.87991900	85.18155000	19.90302700
H	18.45954500	85.90433700	23.74476700
H	22.00358500	75.02507200	24.37943700
H	26.41226200	81.80277300	27.59374300
H	26.67666100	79.55717200	26.85031300

^{5}TS1$_{HA,C1S,B2}$

C	29.05690800	88.44997800	29.56030700
C	27.94672400	87.53447500	29.14334400
N	26.80936600	87.37998300	29.91449900
C	27.85844000	86.74864500	28.01682100
C	26.05608000	86.51914900	29.25685400
N	26.63985500	86.10656900	28.09686800
H	29.45402400	88.16974000	30.54373200
H	29.88225100	88.42228000	28.84040800
H	28.53311600	86.58095600	27.19097200
H	25.08702300	86.15625000	29.57389400
H	26.28299200	85.40491800	27.44680100
C	27.49035100	83.93277800	34.29194500
C	27.34450600	83.18424500	32.98602200
C	27.21414800	81.78927500	32.95792900
C	27.35461300	83.86538300	31.75984300
C	27.10562300	81.08361400	31.75740000
C	27.24046200	83.17979400	30.55066400
C	27.11202900	81.78682400	30.54663100
O	27.00408400	81.13800000	29.33627100
H	28.54394400	84.14431900	34.52175700
H	27.08494900	83.35714300	35.13151300
H	27.20183100	81.23601900	33.89527300
H	27.43088300	84.95048000	31.74018300
H	27.02676400	79.99970300	31.74407200
H	27.22589600	83.71884600	29.60857500
H	26.44921300	80.29263700	29.43365200
C	32.68282500	82.62995300	29.31125000
C	31.64480500	82.81119800	28.19013000
C	33.64453900	83.81227800	29.48157200
C	30.63634500	83.94051600	28.43453300
H	32.15744400	82.45009600	30.26025900
H	32.17010600	82.98917300	27.23909600
H	31.09469400	81.86812300	28.06391300
H	29.89629100	83.98992200	27.62859100
H	31.12505300	84.91986100	28.49445800
H	30.08895000	83.78562100	29.37210100
H	34.38561700	83.61033500	30.26382000
H	34.19010900	84.01391300	28.55036300
H	33.11416100	84.72941600	29.76007100
C	22.36090300	74.52605800	25.61175900
C	23.86576800	74.65448700	25.65906900
O	24.45116600	75.51587300	24.95999400
N	24.53942600	73.84073400	26.48008800
H	21.92658000	75.38179700	26.14704000
H	21.99779000	73.60065700	26.06808700
H	24.02980300	73.19322900	27.06420200
H	25.52332400	74.04233600	26.73338000
C	26.40506200	75.51895600	20.07350200
C	26.45801900	76.82745000	19.27824100
O	27.33696700	77.02133300	18.44532000
C	25.99530700	75.59543000	21.55473500
C	26.88696600	76.45502500	22.40274900
N	26.84243000	76.40889800	23.78763800
C	27.81592100	77.42041200	22.10579600
C	27.70390000	77.32395300	24.27509700
N	28.31093400	77.95757400	23.27845300

H	25.70675500	74.84889400	19.55494100
H	25.99229300	74.57352000	21.95612200
H	24.95235500	75.93484500	21.64807500
H	28.15970200	77.76958200	21.14540600
H	27.89098600	77.49429900	25.32696100
H	26.17335400	75.87043500	24.34915700
N	25.43525600	77.71588100	19.50374600
C	25.54114400	79.08151600	19.02282400
C	25.76045800	80.02827100	20.21419400
O	25.36669600	79.70798300	21.33831500
H	24.95478200	77.65424600	20.39427000
H	26.36662400	79.12098100	18.30913800
N	26.40537000	81.18089300	19.92033400
C	26.56862100	82.26873300	20.87512500
C	25.64824300	83.42987200	20.44379700
O	25.42305800	83.62331900	19.24680100
C	28.01502400	82.79080800	20.87832000
C	29.11876800	81.79754700	21.25735300
O	28.75877900	80.79994100	21.99917000
O	30.27133400	82.03010700	20.86660100
H	26.45572000	81.46424200	18.94755800
H	26.31162200	81.88454600	21.86141000
H	28.26642500	83.21865300	19.90172300
H	28.07280800	83.61059800	21.60676400
N	25.18299900	84.20677200	21.44792100
C	24.39810600	85.41064100	21.19913100
C	25.24492200	86.68816000	21.02287300
C	24.33091500	87.86985100	20.66960900
C	26.08615500	86.99396200	22.27028600
H	23.70338300	85.53762100	22.03714300
H	25.92281600	86.50682300	20.17678300
H	24.91371300	88.78367500	20.50373800
H	23.75531000	87.66989600	19.75784100
H	23.61933600	88.07138100	21.48073900
H	26.73947700	86.15066600	22.52651800
H	26.71505000	87.87915700	22.11072000
H	25.44437300	87.19223500	23.13798400
H	25.42994500	83.96476700	22.40510600
C	19.73749200	86.45781200	23.10916900
C	20.99703500	86.69852100	23.91031800
C	21.36194700	87.99319200	24.30553600
C	21.81769400	85.62995600	24.30064900
C	22.50447400	88.21498800	25.07792300
C	22.96505100	85.84481800	25.06992300
C	23.30921000	87.14110900	25.46488500
H	18.87079400	86.30506200	23.76673300
H	19.50455200	87.30947800	22.46054100
H	20.74131200	88.83609000	24.00795000
H	21.55572900	84.61797000	23.99719500
H	22.76500100	89.22712200	25.37724000
H	23.59999600	85.01086900	25.35583500
H	24.19652800	87.30606100	26.07036700
C	19.28851000	77.32490300	24.21333500
C	19.03732300	78.76879400	23.74202500
C	20.27767700	79.63873500	23.78059900
C	20.46254200	80.59517000	24.78767400
C	21.28111000	79.50326500	22.80734600
C	21.59879200	81.40945000	24.81449100
C	22.42281900	80.31080900	22.82470200
C	22.57890000	81.27621100	23.82796000
H	19.68482500	77.31087400	25.23361400
H	18.63704900	78.75182500	22.71925500
H	18.26080200	79.22177900	24.37042900
H	19.70050600	80.71242900	25.55497000
H	21.15382500	78.77215000	22.01048200
H	21.71150500	82.14874100	25.60387400
H	23.18888700	80.19877700	22.06246700
H	23.45210000	81.92327000	23.82501600
C	32.78091200	75.82044700	20.53774600
C	32.05206900	77.05439800	20.95718200
N	32.05048500	78.22640000	20.21184100
C	31.27291000	77.34112700	22.04747900
C	31.28996600	79.15014000	20.85265300
N	30.80295200	78.63892700	21.96982200
H	33.85801600	75.99714700	20.42070600
H	32.65384600	75.04092400	21.29389100
H	31.01782200	76.70559300	22.88184300
H	31.12271900	80.16774900	20.52030400
H	32.51136800	78.36446900	19.32382500
C	32.94776700	84.01971200	20.83636500
C	32.47997600	83.71912400	22.25667000
N	31.46274900	84.69552700	22.66357400
C	30.50960600	84.49924800	23.58832300
N	30.41864000	83.37443900	24.28440400
N	29.63986400	85.50399400	23.86157600
H	33.35106000	85.03686600	20.74500400
H	32.11575700	83.89221600	20.13875700
H	32.02746700	82.72662200	22.27592200
H	33.32017100	83.74444100	22.96464100
H	31.56302600	85.63655900	22.30826100
H	30.98376300	82.54167000	24.10099400
H	29.57258300	83.24547600	24.84396900
H	29.47220500	86.20422500	23.15323700
H	28.84048700	85.24813700	24.44843000
Fe	29.49270700	79.71367900	23.45916700
C	25.59078600	80.82920600	25.87642500
C	26.82175600	81.67746100	26.07949700
N	25.72306200	79.47360400	26.49339100
S	26.61768200	83.34252600	25.43869700
O	27.97361600	83.97919100	25.56167000
O	26.14832200	83.19419800	24.03630400
O	25.61657100	83.98113700	26.34385000
H	25.40071600	80.69495400	24.80963400
H	27.20156700	81.70580800	27.10402400
H	27.80364200	81.10534600	25.34221000
H	25.49114700	79.42095200	27.52386500
H	25.07433000	78.83048000	25.99344700
C	31.72500800	79.82248900	24.69011200
O	31.34039700	80.75816200	23.90199500
O	30.98644000	78.81343600	24.85159000
C	33.05182000	79.96027000	25.41160700
C	33.74065800	78.62595900	25.70960200
H	33.94938400	78.07599800	24.78548900
H	34.69022100	78.79284700	26.22912300
O	23.65697900	78.10815100	25.08717200
H	23.86444100	77.16932200	24.85106200
H	23.36992900	78.57078300	24.27804500
H	33.11222800	77.98987400	26.33896300
H	33.69333900	80.61374100	24.81095600
H	32.84870600	80.50193400	26.34637700
O	28.58989200	80.45001200	24.77575500
H	32.40338400	75.42427100	19.58620600
H	24.62033700	79.37822400	18.50201000
H	33.74466800	83.32374000	20.55595700
H	33.26971100	81.72177500	29.11027300
H	26.97011300	84.89686900	34.26162700

H	28.70995700	89.48799900	29.63978100
H	18.36025900	76.74199900	24.18654900
H	20.01572200	76.82304400	23.56263600
H	27.40006900	75.07606700	19.97901700
H	23.81269700	85.23591300	20.29176900
H	19.82633800	85.56627200	22.47912000
H	22.02399500	74.57325000	24.57259300
H	24.71440100	81.31268700	26.32155200
H	26.69991400	79.12250800	26.45899800

^{5}TS1$_{HA,C1R,B2}$

C	30.86007900	89.39484100	27.03870800
C	29.74713100	88.49775600	26.59421900
N	29.89309400	87.62978200	25.52248700
C	28.47947400	88.37735400	27.11388600
C	28.72964500	87.01309300	25.40294000
N	27.83861100	87.43158800	26.33747500
H	31.14975900	90.09267000	26.24395300
H	30.56564100	89.98199200	27.91399600
H	27.98835600	88.87382400	27.93710800
H	28.49056700	86.24174000	24.68550200
H	26.94596300	86.98566900	26.51580800
C	24.35170500	84.01684000	29.49671800
C	24.14339500	82.53033500	29.32332500
C	25.06192800	81.60181100	29.83513500
C	23.03586500	82.02086500	28.62883400
C	24.89919300	80.22689400	29.65409200
C	22.85585600	80.64680900	28.43652600
C	23.79534500	79.73700800	28.94451900
O	23.57965700	78.40916100	28.71838200
H	24.97935100	84.42273800	28.69335400
H	24.84513300	84.24096300	30.44855900
H	25.92266200	81.95803400	30.39636700
H	22.29469800	82.70781600	28.22610200
H	25.62256100	79.53680700	30.08071000
H	21.98721800	80.26587800	27.90701900
H	24.39674800	77.89971200	28.89245200
C	29.04806600	79.58295100	29.50447400
C	30.13355400	80.38936300	28.77112800
C	29.29681400	79.39775800	31.00678500
C	30.24856200	81.85288700	29.21426600
H	28.07552900	80.07360200	29.34897000
H	31.10269800	79.88738700	28.91274800
H	29.91531800	80.36063100	27.69567700
H	31.04549400	82.36256700	28.66122400
H	30.48082700	81.94361000	30.28130700
H	29.31758900	82.39918100	29.02003500
H	28.52055700	78.77539800	31.46967900
H	30.26241700	78.91003500	31.18924300
H	29.30475900	80.35474000	31.53940200
C	25.93098700	74.54275200	28.32806900
C	26.11864400	75.91361300	27.71158000
O	26.08736800	76.09333800	26.49359200
N	26.25095900	76.96508400	28.59570400
H	24.90269100	74.44315600	28.69547200
H	26.60517800	74.37570300	29.17472600
H	26.60123600	76.76347000	29.52438100
H	26.56243300	77.84420000	28.17038600
C	24.74201600	76.75491400	20.25015500
C	25.16471400	78.14513700	19.77767700
O	26.31023300	78.38749400	19.40109700
C	24.60542200	76.64169100	21.79499500
C	25.83281500	77.04995900	22.55232700
N	26.78026000	76.16046700	23.03914700
C	26.34041700	78.29294900	22.83652900
C	27.79585000	76.86926800	23.58849800
N	27.56222000	78.16615700	23.46741900
H	23.78531900	76.47201400	19.79340400
H	24.33464500	75.60773100	22.04091400
H	23.76384500	77.25762700	22.13069200
H	25.92836600	79.26053400	22.59296000
H	28.66527100	76.43602800	24.05713200
H	26.69336500	75.15387500	23.06307400
N	24.19253000	79.10438400	19.79874300
C	24.49247900	80.49164400	19.50420700
C	25.03232200	81.25689400	20.73123200
O	24.65780300	80.96805100	21.87198800
H	23.29722500	78.89244300	20.21530600
H	25.21789600	80.51396900	18.68800600
N	25.91214100	82.23543000	20.42630900
C	26.47464000	83.18022900	21.37784700
C	26.15875000	84.60269700	20.86931700
O	25.87296300	84.79646100	19.68793000
C	28.00467200	83.01786700	21.53407000
C	28.41768300	81.83695900	22.38412000
O	27.61346800	80.86821200	22.57617600
O	29.56921300	81.78825600	22.90315700
H	25.97287400	82.55124300	19.46240300
H	25.99625600	83.01574800	22.34210700
H	28.46578100	82.91135600	20.54308500
H	28.43529800	83.91024700	21.99300700
N	26.24786500	85.57223700	21.80943300
C	26.05046500	86.98667100	21.48608800
C	27.23997700	87.65691500	20.76982800
C	26.86480200	89.09962900	20.40227400
C	28.52500200	87.61142000	21.60580200
H	25.83436900	87.50488600	22.42737000
H	27.40564400	87.09666300	19.84034400
H	27.67574800	89.58468900	19.84784200
H	25.96498000	89.13426800	19.77683300
H	26.67378400	89.70052400	21.30121200
H	28.81167900	86.58170600	21.85035000
H	29.36011000	88.06507000	21.05977000
H	28.41191400	88.16020200	22.54947500
H	26.52792400	85.30341100	22.74946900
C	19.05747200	85.80848800	23.43101900
C	20.40586400	85.61587800	24.08700800
C	20.54034000	85.64369100	25.48312500
C	21.56353900	85.43797900	23.31465300
C	21.79205400	85.51001500	26.08843800
C	22.81710400	85.30509900	23.91625000
C	22.93981800	85.34203300	25.30877700
H	18.97684700	85.23647200	22.50036600
H	18.24040400	85.49949200	24.09135900
H	19.65498800	85.78148300	26.10022100
H	21.48165500	85.41580300	22.22985800
H	21.87211100	85.54879100	27.17234400
H	23.70212600	85.18523100	23.29556200
H	23.91754400	85.25194000	25.77507600
C	22.84811200	77.01974900	25.37164500
C	21.56058400	77.42023800	24.62755900
C	21.32999000	78.91775200	24.59483200
C	20.40869700	79.52365900	25.46070300
C	22.04686300	79.74494000	23.71199400
C	20.21248400	80.90804800	25.45287300
C	21.85323500	81.13135900	23.70020500

C	20.93789500	81.72010500	24.57787400
H	22.84182000	77.38491900	26.40442400
H	21.60046600	77.03228500	23.60043400
H	20.70219100	76.93343300	25.10522700
H	19.83199100	78.90203800	26.14208700
H	22.75783400	79.31373400	23.00931500
H	19.48524500	81.35147800	26.12819900
H	22.42095600	81.73977100	23.00234700
H	20.78595200	82.79510000	24.56970000
C	32.48121400	78.45325400	19.24501800
C	31.31149500	78.67101100	20.14760200
N	29.99548400	78.50159700	19.74121000
C	31.21768700	79.05181600	21.46149400
C	29.17673600	78.77365600	20.78491600
N	29.89077400	79.10721500	21.84877900
H	32.45403500	79.11608500	18.37094100
H	33.40987100	78.65503300	19.78548500
H	32.00691600	79.30410300	22.15231700
H	28.10055000	78.73253600	20.70879200
H	29.67878800	78.22900900	18.82065700
C	33.81906800	83.51822300	22.35447200
C	32.92817600	82.94356800	23.45200400
N	32.47160200	84.01500900	24.34257400
C	31.49490500	83.88304600	25.25702500
N	30.95386600	82.69455200	25.52268800
N	31.09811700	84.95930400	25.96374300
H	34.71074300	84.00139800	22.77175300
H	33.27624100	84.25194300	21.74907200
H	32.04597600	82.46332100	23.01729300
H	33.47602600	82.18447800	24.02725800
H	32.97999500	84.88871500	24.33835500
H	31.19716400	81.84947900	25.00904800
H	30.02864500	82.70249000	25.95160200
H	31.14376500	85.89669200	25.55762100
H	30.24195900	84.82085100	26.49808600
Fe	29.05596300	79.80796100	23.78187700
C	25.34198300	81.84488800	25.13611900
C	26.55642700	82.15771800	25.97469400
N	24.60757400	80.65727000	25.68858700
S	27.19276500	83.83525100	25.82596900
O	28.49213500	83.78148500	26.55578200
O	27.30944400	84.10186100	24.36259200
O	26.16572300	84.68423100	26.49441600
H	25.60471600	81.61351100	24.10085000
H	26.48569600	81.88667600	27.03093400
H	27.59073900	81.34041000	25.54800700
H	24.13024400	80.88453200	26.57693200
H	23.87344600	80.35304900	25.03087700
C	31.09580500	78.83737700	24.98718600
O	31.13077400	79.99332000	24.44065500
O	30.04559800	78.14479700	24.86076800
C	32.29281000	78.33685500	25.76774400
C	32.19532800	76.87948200	26.21899300
H	32.11134400	76.20393800	25.36156100
H	33.08618300	76.59684000	26.78901800
O	26.47495900	78.84597900	26.45471200
H	26.41260400	77.92566100	26.11368100
H	27.29480100	79.26691100	26.06474700
H	31.31748800	76.72408800	26.85294800
H	33.18249300	78.50617100	25.14819600
H	32.40338700	79.00686500	26.63106100
O	28.32718700	80.41947900	25.35027600
H	32.52926400	77.42050300	18.87748400
H	23.57465800	80.98348900	19.16336200
H	34.15752000	82.71523900	21.69301800
H	28.97214800	78.59364200	29.02912300
H	23.39792800	84.55542400	29.48026200
H	31.75384500	88.81698500	27.30421400
H	22.96087200	75.92999900	25.39817800
H	23.73867500	77.42636300	24.87727400
H	25.50518400	76.06104000	19.88886500
H	25.16096300	87.06052900	20.85219700
H	18.88872700	86.86353700	23.17792100
H	26.10350500	73.78113600	27.56671500
H	24.64065800	82.68739500	25.13524100
H	25.27451600	79.85176900	25.89844500

^{5}TS1$_{HA,C2S,B2}$

C	29.37974000	89.91162900	26.46328600
C	28.44451800	88.75383100	26.29815200
N	28.31472200	88.08960500	25.08847000
C	27.60661000	88.18586300	27.23056100
C	27.40194200	87.15478500	25.30143600
N	26.93844200	87.17100000	26.57705300
H	29.10475000	90.74373900	25.80351500
H	29.37083200	90.27968200	27.49435700
H	27.42786400	88.41142000	28.27137200
H	27.06337700	86.42354000	24.58055800
H	26.39270500	86.40576800	26.98106200
C	28.15138000	83.21360500	34.99822100
C	27.95743000	82.60058400	33.62964400
C	27.49639300	81.28569100	33.48089300
C	28.21009700	83.33614700	32.46329000
C	27.28666200	80.71569800	32.22373000
C	27.99894700	82.79135500	31.19711000
C	27.53014300	81.47829700	31.07350300
O	27.35891600	80.96510600	29.81290400
H	28.94582300	83.96847000	34.99103000
H	28.41620900	82.45565300	35.74408500
H	27.30456800	80.68507200	34.36823300
H	28.57429600	84.35843000	32.54455800
H	26.96505900	79.68330400	32.11711400
H	28.19342300	83.36887800	30.29800000
H	26.67303900	80.21725000	29.79623800
C	33.31988900	82.23176200	29.09945400
C	31.96897000	82.38908900	28.38030600
C	34.30476800	83.38255000	28.85846800
C	31.12554100	83.57624600	28.85990200
H	33.14133500	82.12739700	30.17960000
H	32.14345900	82.48685200	27.29825900
H	31.38980600	81.46481600	28.51052300
H	30.16453300	83.60707500	28.33510400
H	31.63043200	84.53324200	28.68302500
H	30.91809500	83.50126000	29.93494400
H	35.26191900	83.19679100	29.35993900
H	34.50983000	83.50859000	27.78719600
H	33.91611400	84.33560700	29.23406700
C	22.43577100	74.73691400	25.22224900
C	23.88612600	74.67978400	25.65070300
O	24.75103600	75.35411800	25.04657700
N	24.21169900	73.89001200	26.68093100
H	22.07597200	75.76901900	25.32542100
H	21.78876400	74.07208700	25.80123300
H	23.49273700	73.39013900	27.18351700
H	25.15908500	73.94299400	27.09417500
C	26.01336200	75.67696900	20.58769700

C	25.60833300	77.11674000	20.26413700
O	26.15199000	77.74422900	19.36206800
C	26.15936900	75.32579600	22.07760000
C	27.08015600	76.24496300	22.82378100
N	27.28355900	76.13236100	24.19126100
C	27.83592500	77.32250200	22.43688000
C	28.10980200	77.12554300	24.58545700
N	28.46433100	77.86319100	23.54156300
H	25.27452200	75.00148500	20.13722200
H	26.52302500	74.29123300	22.14968500
H	25.18283500	75.31419700	22.58056500
H	27.95606000	77.75908500	21.45793700
H	28.42325400	77.28460400	25.60875500
H	26.72025800	75.54601200	24.80797100
N	24.57970000	77.64537500	20.99943500
C	24.22195100	79.04381700	20.85593500
C	25.07509200	79.97825800	21.72764700
O	25.29984800	79.71687000	22.91986400
H	24.27610700	77.16872800	21.83601300
H	24.31682800	79.30085400	19.79966400
N	25.50997400	81.08882200	21.10818100
C	26.03712000	82.27368500	21.77617100
C	25.05172700	83.41961700	21.45017600
O	24.44922900	83.41889800	20.37013100
C	27.42162900	82.68751700	21.24450200
C	28.64002100	81.82569700	21.59832800
O	28.43401000	80.79863800	22.35908500
O	29.73513800	82.18153800	21.14294900
H	25.12833800	81.29665700	20.18998400
H	26.09255100	82.07827600	22.84631400
H	27.38666100	82.78311000	20.15268500
H	27.64839800	83.69120300	21.62419900
N	24.95309000	84.39887700	22.37128800
C	24.11614700	85.57876600	22.14863100
C	24.71733900	86.60643100	21.16913700
C	23.69916300	87.72909300	20.92261800
C	26.05424700	87.16955900	21.66712400
H	23.94286200	86.04082900	23.12501800
H	24.88927400	86.08159100	20.22068300
H	24.09251000	88.46766700	20.21435800
H	22.76163300	87.33594600	20.51144400
H	23.45976400	88.25529700	21.85595400
H	26.78379000	86.37118500	21.84653500
H	26.48946800	87.85546400	20.93067900
H	25.92965700	87.72833000	22.60365000
H	25.41365700	84.27024400	23.27117300
C	19.47785500	84.90632100	24.17838700
C	20.65957200	85.69757200	24.69200300
C	20.68194800	87.09687200	24.62271900
C	21.76263400	85.04533000	25.26232600
C	21.77085600	87.82495500	25.10958400
C	22.85724900	85.76418600	25.74699400
C	22.86272100	87.16096900	25.67226900
H	18.83747300	84.56437400	25.00271500
H	18.85439100	85.50742900	23.50811700
H	19.83844200	87.62202700	24.17918800
H	21.76627600	83.95869100	25.31764100
H	21.76594000	88.91036500	25.04474200
H	23.71004300	85.23858400	26.16828200
H	23.71647700	87.72291400	26.04248600
C	19.91426700	77.45469000	20.57530900
C	19.21401800	78.69193400	21.16620900
C	20.18606700	79.71816300	21.71274200
C	20.68167200	79.61095000	23.02105700
C	20.63784400	80.78029600	20.91509800
C	21.59379300	80.54294500	23.52050600
C	21.55517800	81.71329100	21.40616900
C	22.03214800	81.59820200	22.71571500
H	20.52380000	76.95084400	21.33469600
H	18.58925500	79.15431400	20.39159400
H	18.53372800	78.37017500	21.96502700
H	20.35670700	78.79769400	23.66590300
H	20.26335100	80.87935400	19.89790700
H	21.95329900	80.42879300	24.53866400
H	21.91354700	82.51853400	20.77102800
H	22.74244100	82.32467300	23.10116500
C	32.65048100	76.26824600	20.03589900
C	31.88899900	77.41216800	20.62025400
N	31.74009400	78.63441300	19.97719600
C	31.20735200	77.56098900	21.79972600
C	30.99438900	79.45246700	20.76336500
N	30.65376300	78.82540800	21.87575300
H	33.70316500	76.52175700	19.85457400
H	32.62932700	75.41971300	20.72526000
H	31.07901500	76.84863900	22.60058400
H	30.72842500	80.47731100	20.53284200
H	32.10611100	78.87511900	19.06721300
C	32.54108500	84.84025600	21.31554100
C	32.07075500	84.05999900	22.53951300
N	31.43435000	84.96803000	23.49931700
C	30.60776600	84.58868300	24.49264900
N	30.35215100	83.30908900	24.73250100
N	30.05555400	85.53323800	25.28254400
H	33.26701900	85.61921700	21.58250100
H	31.69440800	85.31019700	20.80504800
H	31.33836100	83.30701000	22.23457500
H	32.91731900	83.54279700	23.01315100
H	31.74771900	85.92889900	23.51965700
H	30.85007800	82.53157800	24.29592100
H	29.57803100	83.08594000	25.36275400
H	29.84641500	86.45590400	24.90510000
H	29.34902300	85.18357700	25.93268100
Fe	29.49864000	79.73146700	23.61680800
C	26.49632200	80.79697100	25.99575100
C	26.23897900	81.94633800	26.93628900
N	26.34181600	79.47043600	26.65243100
S	26.70918100	83.58060700	26.28433100
O	28.18717400	83.68748900	26.52690100
O	26.35434600	83.58710600	24.83576600
O	25.91840000	84.54566900	27.10343600
H	27.71054200	80.75970800	25.53269800
H	25.16228900	82.01877400	27.14305100
H	26.75744500	81.83230700	27.89331500
H	25.87283300	79.47821900	27.59734300
H	25.78544400	78.83479400	26.03266500
C	31.84416000	79.85543600	24.55886600
O	31.33793300	80.81846300	23.87722500
O	31.16375300	78.81139700	24.74212100
C	33.25414200	79.99429500	25.09696800
C	33.71445800	78.82497200	25.96708300
H	33.67731500	77.88342900	25.41100600
H	34.74227200	78.98434600	26.31087000
O	24.57128100	78.11337500	24.93296900
H	24.67889900	77.14041200	24.83013800
H	24.78545600	78.55780900	24.07120600
H	33.07109700	78.71672200	26.84588300

H	33.91550100	80.12559900	24.22962700
H	33.29851200	80.94071200	25.65024900
O	28.77349000	80.34114200	25.12280600
H	32.22141900	75.93200100	19.08300700
H	23.17850200	79.18069500	21.15791900
H	33.02818900	84.16018300	20.61022900
H	33.78679700	81.29036300	28.77628300
H	27.23758500	83.71032800	35.35295700
H	30.41057000	89.63072200	26.21351400
H	19.18293800	76.73231000	20.19397900
H	20.57691900	77.73554500	19.74851500
H	26.95720100	75.51355700	20.06097200
H	23.14670000	85.24301100	21.76442000
H	19.80206100	84.01365900	23.63157800
H	22.36657200	74.46608800	24.16350300
H	25.93868300	80.82257300	25.05931900
H	27.26941500	79.03416500	26.86021000

^{5}TS1$_{HA,C2R,B2}$

C	29.69072400	89.86524700	25.98160600
C	28.80191000	88.71590300	25.61825500
N	29.02954500	87.94824700	24.48631000
C	27.68450000	88.25161700	26.27449000
C	28.04978600	87.05654400	24.46710000
N	27.20969500	87.19700100	25.52233300
H	29.67792100	90.63910300	25.20429700
H	29.37228300	90.32459200	26.92290400
H	27.19191600	88.57761500	27.17841100
H	27.92928300	86.26318400	23.74274700
H	26.52364700	86.48901100	25.79269100
C	28.99351800	81.36340800	35.60470400
C	28.70387800	81.09407300	34.14528000
C	28.06580600	79.91582600	33.73617000
C	29.04204000	82.02743800	33.15510100
C	27.76724000	79.66695000	32.39509400
C	28.74527100	81.80448400	31.81150200
C	28.10106800	80.62359700	31.42656500
O	27.85048000	80.42015600	30.09083800
H	29.90505000	81.95875100	35.73059100
H	29.12125900	80.43169800	36.16710100
H	27.80038400	79.16733900	34.48054300
H	29.54273500	82.95078100	33.43944900
H	27.29977000	78.73559200	32.08555900
H	29.00041300	82.53813100	31.05225900
H	27.08168200	79.77178400	29.96708600
C	30.36446700	85.18218200	29.60168400
C	31.58566000	84.33405900	29.99852000
C	30.71496400	86.56660200	29.04170000
C	32.49587600	83.94456400	28.82670400
H	29.76149300	84.63181500	28.86576000
H	32.17507300	84.87867200	30.75098500
H	31.23411900	83.41824500	30.49300000
H	33.30848900	83.28849700	29.16152900
H	32.95451800	84.82226900	28.35754200
H	31.93262100	83.41262700	28.04947300
H	29.80902200	87.13462700	28.80028800
H	31.29499200	87.15175800	29.76741600
H	31.30503900	86.49297300	28.12155600
C	21.87603400	75.85648600	24.47109700
C	23.34881300	75.61027000	24.69802900
O	24.21893000	76.27415500	24.09276800
N	23.69796800	74.67724800	25.59827200
H	21.61222700	76.78408500	24.99781300
H	21.24297100	75.04364600	24.83902800
H	22.98993800	74.19268200	26.13090300
H	24.67622000	74.60978400	25.91033000
C	26.22417400	75.74150100	20.62108400
C	25.68606800	77.17282800	20.53219200
O	26.04259400	77.92737100	19.62381200
C	26.52674100	75.20439900	22.03278100
C	27.43260100	76.11090700	22.80879900
N	27.61216300	76.02600000	24.18255800
C	28.19554500	77.18186400	22.41475700
C	28.44277800	77.02078300	24.56638900
N	28.81288600	77.73693000	23.51591800
H	25.49766900	75.07581500	20.13722100
H	26.97436700	74.20563500	21.92946000
H	25.60005800	75.06305900	22.60014900
H	28.31331100	77.61574500	21.43409800
H	28.73233000	77.20653200	25.59086700
H	27.14754600	75.40202100	24.84321600
N	24.77149800	77.54843000	21.46447200
C	24.17402500	78.87280300	21.41065200
C	25.05053400	80.00040300	21.98031600
O	24.88981600	80.41325300	23.14895600
H	24.55791100	76.97001000	22.27351100
H	23.92364400	79.10391500	20.37152100
N	25.94946600	80.54605700	21.15449600
C	26.58472500	81.81890700	21.47404200
C	25.56147500	82.95037100	21.18436000
O	24.71654900	82.83832300	20.29547900
C	27.88444000	82.03903400	20.68044800
C	29.15237700	81.44344200	21.30970200
O	28.96305800	80.43185900	22.09891600
O	30.25038000	81.93303200	21.01907000
H	26.00212200	80.17325100	20.21260800
H	26.82036200	81.81131100	22.53898100
H	27.78473700	81.64245500	19.66040000
H	28.06902800	83.11107000	20.56755200
N	25.70491000	84.04483300	21.96701300
C	24.79016500	85.17912900	21.86426200
C	25.09236900	86.13286000	20.69131500
C	24.00410900	87.21224700	20.61083200
C	26.48958500	86.75601800	20.80006600
H	24.84061600	85.72231100	22.81513000
H	25.04805200	85.52860100	19.77579900
H	24.17405300	87.87843300	19.75688800
H	23.00767100	86.76885400	20.49781300
H	23.99308500	87.83167500	21.51749800
H	27.26669500	85.98479000	20.85038500
H	26.70532100	87.39161100	19.93284800
H	26.57698500	87.38180400	21.69833600
H	26.31328700	83.97615900	22.78254600
C	19.17487400	84.61164300	23.95828300
C	20.26072000	85.30359700	24.75058300
C	20.00757500	86.49781800	25.43791800
C	21.55797400	84.77122100	24.80013700
C	21.01902100	87.14733300	26.14949900
C	22.57502800	85.41415000	25.50814100
C	22.30613900	86.60824500	26.18547500
H	19.19779500	83.52641500	24.10549300
H	18.18072300	84.97610800	24.23919900
H	19.00634600	86.92363800	25.41559300
H	21.76819100	83.84106400	24.27669200
H	20.79954100	88.07337600	26.67566100
H	23.57437100	84.98701000	25.53709900

H	23.09614000	87.11283000	26.73622700
C	17.57621600	79.85962500	19.87493800
C	17.70919200	81.08128100	20.80262700
C	19.06196000	81.15991000	21.48086700
C	19.27592900	80.56179500	22.73139800
C	20.14300800	81.79619300	20.85323800
C	20.53221000	80.59643400	23.34183300
C	21.40386300	81.83662800	21.45242900
C	21.59765600	81.23793800	22.70198500
H	17.70851500	78.92676900	20.43510100
H	17.53677600	81.99442000	20.21839000
H	16.91766200	81.04317500	21.56203700
H	18.44658900	80.06473000	23.23207500
H	19.99367000	82.26695100	19.88310200
H	20.69021000	80.12203200	24.30801100
H	22.23551400	82.32656900	20.95064400
H	22.57818900	81.26229100	23.16807400
C	33.52730400	76.06357800	20.60221100
C	32.67446700	77.20417700	21.05165600
N	32.61367800	78.41897100	20.38022600
C	31.82020000	77.35665600	22.11275500
C	31.74994300	79.23632800	21.03579100
N	31.25172700	78.61497500	22.09009100
H	34.59212600	76.32941300	20.57637500
H	33.41357100	75.22303700	21.29228100
H	31.57844600	76.65147600	22.89349100
H	31.50601300	80.25311800	20.74977100
H	33.11616400	78.65638600	19.53686800
C	33.06176800	84.38854100	21.66450000
C	32.46202900	83.67772400	22.87339500
N	31.87032100	84.66265200	23.78714500
C	31.01447400	84.38356300	24.78396300
N	30.73024700	83.13165100	25.13004000
N	30.47755800	85.40100400	25.49210900
H	33.84411400	85.10038900	21.95734300
H	32.28870800	84.92447700	21.10427800
H	31.68473800	82.98409100	22.53970700
H	33.23087100	83.09701700	23.39980400
H	32.14532500	85.62994300	23.68480200
H	31.13721400	82.31296900	24.67100700
H	29.88689100	82.99303500	25.68761400
H	30.32855200	86.29657300	25.02569900
H	29.70671800	85.12827900	26.10359500
Fe	29.87413200	79.56833200	23.59358900
C	27.29555800	80.99568500	26.62227300
C	26.28054700	82.01505200	26.18544900
N	26.75377600	79.65577300	26.90370500
S	27.06072700	83.61845700	25.74766500
O	28.27930100	83.71875900	26.62358200
O	27.41920800	83.54979600	24.30153400
O	26.01334300	84.63593600	26.05643500
H	27.95140500	81.31494500	27.43144500
H	25.70806100	81.69354600	25.30929100
H	25.58773200	82.23859300	27.00879000
H	26.23885800	79.56504800	27.82633100
H	26.09588100	79.30944800	26.16336900
C	32.02519000	79.69757700	24.93710500
O	31.65733600	80.63434600	24.13550000
O	31.34332000	78.64325100	25.00802100
C	33.28109900	79.89371500	25.76498300
C	33.49486100	78.82865100	26.84096200
H	33.55516400	77.83089500	26.39592800
H	34.42309200	79.01976100	27.39035000
O	24.70602900	78.77968100	25.20841900
H	24.71281800	77.86193100	24.85830600
H	24.70775300	79.36725600	24.40020100
H	32.66694600	78.82516900	27.55738600
H	34.12727100	79.91977800	25.06424100
H	33.22908700	80.89760700	26.20437600
O	28.95751500	80.28838600	24.90733900
H	33.24924900	75.71007600	19.60074800
H	23.25293900	78.86375600	21.99593200
H	33.51339400	83.65225800	20.99336000
H	29.72326000	85.30823300	30.48521600
H	28.17622100	81.92043900	36.08339000
H	30.73274300	89.54262500	26.09855400
H	16.58972200	79.83298000	19.39680500
H	18.33663300	79.88328900	19.08613500
H	27.12462000	75.72473800	20.00081700
H	23.76969900	84.79352700	21.75738900
H	19.29795900	84.78384200	22.88097800
H	21.69141500	76.00889700	23.40461600
H	28.16814800	80.74833800	25.61489100
H	27.54501200	78.98716500	27.01858200

Model C structures:

$^{5}Re_{C}$

C	-0.82106400	11.80574000	-4.85426800
H	-0.73266000	11.60148200	-5.92396000
H	0.17990900	11.75722800	-4.41085200
C	-1.73223500	10.81150000	-4.21622200
N	-1.95690100	10.77036500	-2.84660700
C	-2.79537700	9.76876800	-2.54572300
H	-3.11646700	9.48982200	-1.54484200
N	-3.13339700	9.17243800	-3.68651200
H	-3.69252900	8.28652400	-3.73517800
C	-2.48488100	9.79388800	-4.73561300
H	-2.60410300	9.46141100	-5.75426500
C	2.35144000	12.96375700	-1.68591400
H	2.67672900	12.91172200	-2.73139100
H	3.26037000	13.01732100	-1.07183800
C	1.49998700	11.77424300	-1.30307100
C	0.41750800	11.90396500	-0.42136800
H	0.17763600	12.88384000	-0.01268700
C	-0.34735000	10.80038800	-0.02107900
H	-1.14739800	10.90820700	0.70723000
C	-0.04398300	9.52495600	-0.52739300
O	-0.77709300	8.41933200	-0.21629300
H	-1.67638400	8.67210500	0.16319900
C	1.04344800	9.37376200	-1.40007900
H	1.27748100	8.38318900	-1.77944600
C	1.79708300	10.48438400	-1.77530800
H	2.63777600	10.34635100	-2.45227900
C	6.20963500	11.68551000	0.72916300
H	5.30036000	12.06503000	0.23885000
C	7.28058600	11.40177800	-0.33114600
H	6.94882300	10.65090500	-1.05728000
H	7.53333400	12.31032100	-0.88963100
H	8.20329300	11.02996400	0.13169600
C	5.84196700	10.47334600	1.60156800
H	6.74914600	10.10362100	2.10077200
H	5.16893900	10.80792200	2.40278700
C	5.17506600	9.32218000	0.83815900
H	4.90047200	8.50653700	1.51793700
H	4.25884800	9.65674100	0.33516800
H	5.83871000	8.90091800	0.07456000
C	-5.30383500	9.98019300	3.35518500
H	-6.37468400	10.15653400	3.48121100
C	-5.02745600	9.73500100	1.86012800
H	-5.61325400	8.88373200	1.50247400
H	-5.38605300	10.60481600	1.29436600
C	-3.54736300	9.53166900	1.51112600
O	-2.66340300	9.99924900	2.25083100
O	-3.31823700	8.87318100	0.41565500
C	-4.92574900	8.75373800	4.16698200
O	-5.69157900	7.79390300	4.29457100
N	-3.66982300	8.76785600	4.71713700
H	-3.07123100	9.48696000	4.31522900
C	-2.99002400	7.53263800	5.11952000
H	-3.45645600	6.70086400	4.57606600
C	-1.51111500	7.56845600	4.71707500
H	-1.06572000	8.54951100	4.92323100
H	-0.98387300	6.83734000	5.34004900
C	-1.32488800	7.15311800	3.26046900
O	-2.09494500	6.33773500	2.74897900
N	-0.23925900	7.64608600	2.61650100
H	-0.20993300	7.52326100	1.60456800
H	0.18193700	8.49928400	2.95877400
C	-3.12504400	7.12839800	6.59474300
O	-2.42771800	6.21190000	7.04141400
N	-4.07303300	7.74165400	7.33803300
H	-4.66107500	8.43615200	6.89940200
C	-4.36484600	7.29634400	8.69741400
H	-3.41441500	7.07143200	9.19039600
C	-5.29180200	6.06511300	8.73831300
O	-6.45072900	6.15034100	9.14522300
N	-4.74438500	4.91094000	8.27481400
H	-3.81386300	4.92929500	7.85480500
C	-5.52253900	3.69068500	8.14607200
H	-5.87154700	3.37947400	9.13875300
C	-6.79626600	3.84475800	7.24644800
H	-7.12262300	2.85879100	6.90012200
H	-7.58424000	4.28893900	7.85555600
C	-6.51683500	4.70404500	6.01968000
O	-5.86053900	4.26933100	5.06019500
N	-7.02832000	5.94984200	6.05434000
H	-6.70960800	6.61991900	5.35475000
H	-7.39220800	6.30559800	6.92946900
C	-4.62527800	2.59594600	7.56007400
O	-3.64476700	2.85246300	6.86181200
N	-5.01505700	1.31858900	7.84143100
H	-5.93499300	1.20840100	8.25050100
C	-4.61249100	0.23746300	6.95129100
H	-3.54289800	0.32992200	6.75395500
C	-5.45071500	0.32353900	5.66032500
O	-6.62302900	0.69121200	5.70876300
N	-4.81831600	0.01914400	4.50201800
H	-3.84739400	-0.26972700	4.47308900
C	-5.42844100	0.31541000	3.21808500
H	-6.47790200	0.00516400	3.25684100
C	-5.39650900	1.85410300	2.93006000
H	-5.90328500	2.06089600	1.98232000
H	-5.97148900	2.34618500	3.71783500
C	-4.01586600	2.42528000	2.85541300
N	-3.27632300	2.87042600	3.93920100
H	-3.60228600	2.97497000	4.90179700
C	-2.05786600	3.24990900	3.49530200
H	-1.29134900	3.66242000	4.13246800
N	-1.96636800	3.06594700	2.18513300
C	-3.18482500	2.56394700	1.77380300
H	-3.38048000	2.33034300	0.74073900
C	-4.68046300	-0.47376100	2.13636300
O	-3.55952800	-0.93970200	2.33877300
N	-5.34624100	-0.59176200	0.95272400
H	-6.17752200	-0.02630600	0.84126200
C	-4.67160600	-0.94243600	-0.28425800
H	-3.81380300	-1.56977500	-0.03574100
C	-4.25039700	0.32450300	-1.05213400
O	-4.84361900	1.39146000	-0.85478600
N	-3.21839500	0.16542100	-1.90966200
H	-2.96290300	-0.77847300	-2.18166900
C	-2.77850400	1.20443900	-2.83343800
H	-3.28015100	2.12755900	-2.54463100
C	-1.25025000	1.40061200	-2.78271600
H	-0.73966800	0.44055700	-2.92982300
H	-0.94300500	2.04244900	-3.61725500
C	-0.71585200	2.04884400	-1.51171600
O	-1.55373100	2.31024200	-0.57668300
O	0.49341700	2.34122700	-1.39996100
C	-3.17111500	0.77837200	-4.26120600

O	-3.09436900	-0.39990100	-4.60287800
N	-3.54513300	1.79336300	-5.08179500
H	-3.62712700	2.73066800	-4.68670200
C	-3.99189000	1.57757700	-6.46402700
H	-4.23755400	2.58294200	-6.83174700
C	-2.93084400	0.96838000	-7.42168600
H	-2.95515200	-0.11726600	-7.29195900
C	-3.28245800	1.29772400	-8.88229700
H	-3.24876400	2.38189800	-9.05987500
H	-2.56307400	0.82760600	-9.56309600
H	-4.27827700	0.93047800	-9.13501400
C	-1.51193900	1.46354000	-7.10066100
H	-1.45050100	2.55950300	-7.12953800
H	-1.18650700	1.12202100	-6.11151200
H	-0.81518400	1.06850700	-7.85186600
C	-5.32381600	0.77046200	-6.49754100
O	-5.61037800	-0.00132300	-7.39954700
N	-6.18925100	1.07905500	-5.47689700
H	-5.76442000	1.44174800	-4.63113300
C	-7.36419300	0.25117600	-5.22307500
H	-7.13585400	-0.79388400	-5.46032000
C	-7.74561200	0.38180800	-3.71959700
H	-8.24498700	1.35115000	-3.58216800
C	-8.65530200	-0.74683400	-3.24757400
H	-9.53938100	-0.82293100	-3.88469300
H	-8.11332700	-1.69812900	-3.28921600
H	-8.96725300	-0.57212500	-2.21111000
O	-6.52020800	0.38618600	-2.96544400
H	-6.58348300	1.02423600	-2.23854900
C	-8.58568000	0.63838200	-6.08548600
O	-9.56959800	-0.09345000	-6.14285400
N	-8.50298500	1.85312000	-6.68712700
H	-7.62191100	2.34545400	-6.62208000
C	-9.56444700	2.41275400	-7.50406500
H	-10.43603200	1.76792000	-7.36956300
C	-9.89565400	3.86621900	-7.10333200
H	-10.74278700	4.19776000	-7.71681500
H	-10.22889000	3.87305500	-6.05876500
C	-8.72668400	4.81345900	-7.28517600
C	-8.48153800	5.41505000	-8.52861800
H	-9.16511000	5.23078400	-9.35479500
C	-7.37866200	6.24825500	-8.71839100
H	-7.21110500	6.70805100	-9.68898800
C	-6.49469800	6.49184600	-7.66439700
H	-5.63448400	7.13959800	-7.81316000
C	-6.72164300	5.90155400	-6.41909500
H	-6.03605600	6.08112800	-5.59446400
C	-7.83314300	5.07318400	-6.23436900
H	-8.01158600	4.62506700	-5.25922600
C	-9.63245900	8.06783000	1.87094200
H	-9.98535100	7.93092400	2.89990000
C	-8.30974600	7.30964300	1.69292300
H	-7.88877000	7.51759300	0.69950000
H	-7.59306500	7.64229200	2.44525000
O	-8.46854200	5.90049300	1.88507200
H	-8.82800100	5.51348500	1.06651900
C	-9.41009900	9.55379000	1.60785700
O	-8.45067500	10.15477600	2.09222200
N	-10.31983000	10.15331500	0.78731500
H	-11.04465900	9.57941400	0.37927600
C	-10.14096100	11.50074100	0.26370600
H	-9.25160300	11.89599700	0.75750100
C	-10.00288500	11.53439600	-1.27191600
H	-10.98030000	11.32461900	-1.72655600
H	-9.76521200	12.57069400	-1.55366700
C	-8.97815100	10.57895100	-1.86042500
C	-9.26897700	9.88659300	-3.04453500
H	-10.23055800	10.04167100	-3.52984100
C	-8.34685300	9.00366600	-3.61231500
H	-8.59175400	8.48109900	-4.53361800
C	-7.11409400	8.78494500	-2.99304500
H	-6.40097500	8.08506700	-3.41817400
C	-6.81240800	9.46766700	-1.81130600
H	-5.86250300	9.29722000	-1.30841900
C	-7.73106000	10.36092300	-1.25569500
H	-7.48507600	10.86345800	-0.32494000
C	-10.19446800	2.60944000	0.52070700
H	-9.62850300	1.80671700	1.00344700
H	-11.02217600	2.14606700	-0.03082900
C	-9.31803700	3.42875900	-0.39910700
C	-9.78101400	4.64239100	-0.93815000
H	-10.78391300	4.98582500	-0.68988200
C	-8.98468300	5.39796900	-1.80535800
H	-9.35748600	6.33575400	-2.20860000
C	-7.70954900	4.94743300	-2.15908000
H	-7.08585000	5.53065800	-2.83022200
C	-7.23425200	3.74443900	-1.63047200
H	-6.24110000	3.38956300	-1.89080100
C	-8.03008100	3.00227400	-0.75297800
H	-7.63746200	2.08065800	-0.32889200
C	-0.13935300	-2.26885200	2.32825000
H	0.21250600	-2.54719100	3.32972300
H	0.28630500	-2.98673000	1.61544200
C	0.21589300	-0.85983100	1.98603100
N	1.51407400	-0.36332600	1.99738000
C	1.48135600	0.94628900	1.65516200
N	0.23524800	1.32021700	1.42164700
C	-0.56055500	0.20749200	1.62088600
H	-1.63198400	0.24076400	1.51119900
H	2.34686600	-0.88609300	2.23000400
H	2.34450700	1.58887100	1.59176800
C	3.19661800	3.80359600	-2.56767200
H	2.81571000	3.11799900	-1.80525400
H	3.37407400	4.77881600	-2.10941200
N	2.25845800	3.92978900	-3.67765300
H	2.32822000	3.26470400	-4.44096100
C	1.10176700	4.60884000	-3.61940000
N	0.78820500	5.37494200	-2.56732300
H	-0.11542400	5.83247400	-2.59476900
H	1.18549800	5.21245200	-1.63324800
N	0.28271400	4.57106300	-4.68309200
H	-0.64367500	4.99751500	-4.62014500
H	0.55126600	4.01126500	-5.48669000
Fe	-0.35280900	3.22624100	0.77796600
O	-0.79457600	4.70161300	0.25967100
C	-4.19448400	5.52775600	-0.64588100
C	-2.99934000	5.74976600	-1.56609900
N	-3.88964000	6.06166800	0.71334300
S	-3.39477900	5.66474200	-3.32673600
O	-4.39665500	6.76595400	-3.57357000
O	-2.08894700	5.95072300	-3.99921400
O	-3.95634800	4.31316200	-3.58926800
H	-4.43160100	4.46783900	-0.54880100
H	-5.08054200	6.05409100	-1.00780400
H	-2.55640300	6.73838400	-1.41044500
H	-2.22344700	5.00721000	-1.37016900

H	-3.78486500	7.09845800	0.67523300
H	-2.99714400	5.71455700	1.09967300
H	-4.65047900	5.78691600	1.40125400
C	2.00408800	4.85052400	1.15499800
O	1.29483500	3.80668300	1.52553100
O	2.01736600	5.34433300	0.02883700
C	2.85037600	5.42797900	2.27723400
H	3.61084400	6.09794400	1.87113800
H	3.31686100	4.63379400	2.86743400
O	1.57290600	2.50985200	-6.29232300
H	0.95540400	1.76491100	-6.37823400
H	1.99534600	2.59192600	-7.16163800
O	-5.85323200	5.07010300	2.32277600
H	-6.80218700	5.28875300	2.18066200
H	-5.75335000	4.95534800	3.29297100
H	-1.22711100	-2.37515200	2.31211800
H	-4.79487900	-0.71773000	7.45320700
H	2.19183300	5.99597300	2.94492600
H	4.14147800	3.42940800	-2.96836400
H	6.56139500	12.49509800	1.38264800
H	-1.19487800	12.82942700	-4.73496800
H	1.81184300	13.90656300	-1.54697500
H	-4.74536700	10.85890900	3.69089300
H	-4.87227800	8.09875700	9.23388700
H	-5.35395600	-1.51321500	-0.92467200
H	-9.28200700	2.37841700	-8.56536300
H	-10.98588100	12.13459200	0.56062300
H	-10.63810800	3.22637000	1.31064100
H	-10.39791600	7.65111300	1.20390100
H	-1.45761700	11.30934600	-2.13982000

$^{5}TS1_{HA,C1S,C}$

C	-0.85917600	11.81607700	-4.94611600
H	-0.70468600	11.54799800	-5.99397200
H	0.12125800	11.89652600	-4.46354000
C	-1.69888000	10.78357700	-4.27188900
N	-1.96734800	10.80572600	-2.90985500
C	-2.72439800	9.75293900	-2.57098200
H	-3.05526200	9.51046900	-1.56506600
N	-2.96768500	9.05956000	-3.68065700
H	-3.45206000	8.12843500	-3.69773900
C	-2.34011400	9.67252500	-4.74722000
H	-2.39410800	9.26939200	-5.74569200
C	2.06203100	13.40311600	-1.83236100
H	2.50843300	13.26357600	-2.82319300
H	2.88154600	13.64566600	-1.14301500
C	1.31161500	12.17068300	-1.37922400
C	0.21276400	12.26492100	-0.51161600
H	-0.11218100	13.24507400	-0.16741900
C	-0.45986900	11.12875500	-0.04485700
H	-1.27225100	11.21444100	0.67205300
C	-0.04695500	9.85414700	-0.46928000
O	-0.69211200	8.71301600	-0.09239800
H	-1.61996900	8.91391700	0.25057300
C	1.05752300	9.73892400	-1.32441900
H	1.37255500	8.74949600	-1.64309000
C	1.72061200	10.88356200	-1.76589100
H	2.57654300	10.77404900	-2.42845700
C	5.21597100	11.83619500	0.90338700
H	4.26353000	11.93044200	0.36102900
C	6.35447600	11.60978200	-0.09890500
H	6.20255300	10.70029600	-0.69123800
H	6.43659500	12.44889500	-0.79944100
H	7.31866800	11.51279100	0.41560600
C	5.07705500	10.73234300	1.96550700
H	6.02567500	10.63862200	2.51331200
H	4.32897500	11.04894000	2.70524100
C	4.67406600	9.36322700	1.40317600
H	4.54203600	8.63060900	2.20834800
H	3.72852600	9.42639500	0.84950900
H	5.43188500	8.96393100	0.71958200
C	-5.28005100	9.96358200	3.44546100
H	-6.35539900	10.09907500	3.58311800
C	-5.00529100	9.77962100	1.94146400
H	-5.56185000	8.92049900	1.55750700
H	-5.40047800	10.65444600	1.40923700
C	-3.52083500	9.64576300	1.58070800
O	-2.65356500	10.14508100	2.31963100
O	-3.26771500	9.00492200	0.47961300
C	-4.85520000	8.72583900	4.21705600
O	-5.59133700	7.74214000	4.33598100
N	-3.58995000	8.75912700	4.74468700
H	-3.01427500	9.50454900	4.35727600
C	-2.88149000	7.53754700	5.13801200
H	-3.30159200	6.70032600	4.56633700
C	-1.39031800	7.63571400	4.79100000
H	-0.98121200	8.61072600	5.08286100
H	-0.87048400	6.87497800	5.38334600
C	-1.13764100	7.32474000	3.32025100
O	-1.76870900	6.42150300	2.76347500
N	-0.15125400	8.02115400	2.70950800
H	-0.11818200	7.98196000	1.68960900
H	0.13589800	8.90350700	3.11120200
C	-3.03938100	7.09944700	6.60284500
O	-2.33834400	6.18084700	7.03842200
N	-3.99652500	7.69342800	7.34899300
H	-4.58972600	8.38810700	6.91802700
C	-4.28862300	7.22807700	8.70187500
H	-3.33733400	7.01138300	9.19704300
C	-5.19749600	5.98304300	8.72705500
O	-6.35549700	6.04608700	9.14076000
N	-4.63505800	4.84461300	8.24412400
H	-3.70628700	4.88401300	7.82154200
C	-5.39083000	3.61121000	8.10849100
H	-5.72175300	3.28045300	9.10102700
C	-6.67841800	3.75051700	7.22620300
H	-6.99211400	2.76087600	6.87871300
H	-7.46530700	4.17757200	7.84881100
C	-6.42934900	4.62254400	6.00229600
O	-5.78899500	4.20039500	5.02631600
N	-6.94736500	5.86486000	6.05817200
H	-6.64476700	6.54450900	5.36138800
H	-7.29860400	6.20902300	6.94316300
C	-4.47961200	2.54249600	7.49591600
O	-3.52999100	2.82639600	6.76721600
N	-4.82549100	1.25469000	7.79046900
H	-5.73132900	1.12172100	8.22381200
C	-4.42726700	0.18628600	6.88342500
H	-3.36917100	0.30871400	6.64511200
C	-5.31624900	0.25121300	5.62614600
O	-6.49293800	0.59700100	5.71932600
N	-4.72470300	-0.04714800	4.44575700
H	-3.74971700	-0.31541700	4.37862300
C	-5.39316600	0.22506600	3.18585700
H	-6.43599300	-0.09663600	3.27577700

C	-5.39439100	1.76094100	2.88159300
H	-5.93558600	1.94657400	1.94806000
H	-5.95194200	2.25121100	3.68322200
C	-4.02925900	2.35888800	2.75298100
N	-3.26894700	2.86207400	3.79784300
H	-3.56701500	2.98111500	4.76721300
C	-2.07907600	3.26570300	3.30042700
H	-1.30655000	3.72767700	3.89449400
N	-2.02525300	3.04370400	1.99231400
C	-3.23960000	2.48695300	1.64169600
H	-3.46253000	2.20309800	0.62713900
C	-4.68472500	-0.57672200	2.08575000
O	-3.54705500	-1.01719400	2.24284900
N	-5.40892700	-0.74647200	0.94270600
H	-6.25746000	-0.20330600	0.85530900
C	-4.79121400	-1.13987600	-0.31105000
H	-3.93982300	-1.78312000	-0.08110400
C	-4.36333700	0.09891900	-1.12069300
O	-4.91260200	1.18769800	-0.91585900
N	-3.37605900	-0.10320700	-2.01925000
H	-3.14493200	-1.05466000	-2.28255600
C	-2.88537300	0.93788900	-2.91260600
H	-3.32366000	1.87586000	-2.57414300
C	-1.34689200	1.05641500	-2.85870000
H	-0.87922000	0.06873200	-2.96143600
H	-0.99393800	1.65918400	-3.70296700
C	-0.84770000	1.72822200	-1.58845800
O	-1.67107400	1.87990900	-0.62894400
O	0.32802000	2.16685500	-1.49314900
C	-3.32793200	0.61570600	-4.34979900
O	-3.34136300	-0.53934300	-4.76685200
N	-3.65944300	1.70500100	-5.09450000
H	-3.62746400	2.61992300	-4.64691700
C	-4.15238900	1.61305300	-6.47441200
H	-4.33339200	2.65495900	-6.77159100
C	-3.16109100	0.99295000	-7.49887600
H	-3.26439700	-0.09429300	-7.43955000
C	-3.51726700	1.44511000	-8.92543900
H	-3.40360900	2.53312200	-9.03274100
H	-2.85049600	0.96882600	-9.65391600
H	-4.54371000	1.17263700	-9.17476800
C	-1.70299100	1.35889800	-7.18430700
H	-1.55858100	2.44687100	-7.14916900
H	-1.38288500	0.93473500	-6.22620200
H	-1.05382100	0.96211100	-7.97594800
C	-5.54158800	0.90898600	-6.52589100
O	-5.91818600	0.25813600	-7.48864600
N	-6.34556700	1.17202000	-5.44506400
H	-5.86659800	1.40698500	-4.58353500
C	-7.57580100	0.41671100	-5.23109800
H	-7.43023600	-0.62414200	-5.54144200
C	-7.92610800	0.47167000	-3.71481400
H	-8.33560000	1.46877300	-3.50131700
C	-8.92756400	-0.60420800	-3.30957300
H	-9.82034800	-0.56205800	-3.93767800
H	-8.46999900	-1.59322900	-3.42249000
H	-9.21314700	-0.47606900	-2.25886700
O	-6.70179400	0.30705000	-2.97951100
H	-6.64951800	0.96239300	-2.26684300
C	-8.77160800	0.95078500	-6.04904800
O	-9.79875100	0.28944200	-6.16586900
N	-8.61697400	2.20882100	-6.53897300
H	-7.69790800	2.62451400	-6.46531600

C	-9.62265100	2.87528500	-7.34674900
H	-10.56462000	2.34753900	-7.17942200
C	-9.75776100	4.36605500	-6.97365000
H	-10.56344300	4.79238700	-7.58423600
H	-10.07061900	4.43867100	-5.92529400
C	-8.47730700	5.14755500	-7.19086700
C	-8.16096400	5.66485400	-8.45598100
H	-8.86744600	5.54359100	-9.27441800
C	-6.95588900	6.33204900	-8.67781100
H	-6.73271500	6.72751500	-9.66537800
C	-6.03991800	6.49104400	-7.63496300
H	-5.10002700	7.00856800	-7.81004100
C	-6.33830200	5.98624800	-6.36730300
H	-5.63227700	6.10451600	-5.54866800
C	-7.55152300	5.32432100	-6.15078200
H	-7.78317400	4.93861100	-5.16009500
C	-9.53901600	8.00641900	1.82684000
H	-9.89966100	7.87169900	2.85323700
C	-8.20533500	7.26352200	1.66550200
H	-7.77725300	7.47103800	0.67490500
H	-7.50032400	7.60969200	2.42270300
O	-8.34629000	5.85462300	1.86591300
H	-8.71028900	5.45878400	1.05358900
C	-9.33010300	9.49288500	1.55667000
O	-8.39476100	10.11205400	2.06482300
N	-10.22032600	10.06993000	0.69948000
H	-10.92923600	9.48330000	0.28180100
C	-10.04847600	11.41564700	0.16962800
H	-9.18493500	11.83257300	0.69084400
C	-9.85765900	11.43859600	-1.36057100
H	-10.81284600	11.19887200	-1.84653200
H	-9.63748600	12.47811800	-1.64468700
C	-8.78782900	10.50594500	-1.90387900
C	-9.01366800	9.80267000	-3.09584600
H	-9.95854200	9.93212300	-3.61983900
C	-8.04828800	8.94085600	-3.62287700
H	-8.24303500	8.40809000	-4.55022800
C	-6.83660800	8.75486800	-2.95306800
H	-6.09213500	8.06913100	-3.34754100
C	-6.59885000	9.44876000	-1.76326200
H	-5.66744900	9.30440500	-1.21952300
C	-7.56093000	10.32117800	-1.24956700
H	-7.36394600	10.83326800	-0.31255600
C	-10.14517200	2.54645300	0.32915000
H	-9.63113700	2.03373800	1.14944500
H	-10.60940800	1.77457300	-0.29866000
C	-9.19243900	3.39843200	-0.47771800
C	-9.60186900	4.62718200	-1.02085100
H	-10.61959900	4.97156000	-0.84692700
C	-8.73184900	5.39832800	-1.80220900
H	-9.06594300	6.34753200	-2.21259300
C	-7.43510000	4.94747400	-2.05947200
H	-6.75698100	5.53955100	-2.66770500
C	-7.00940100	3.72918300	-1.51944800
H	-6.00142600	3.36884600	-1.70339400
C	-7.87876200	2.97220600	-0.73119800
H	-7.52477100	2.03740800	-0.30232000
C	-0.04092000	-2.26657600	2.64978400
H	0.19921100	-2.42573000	3.70877400
H	0.50981300	-3.01755800	2.06876600
C	0.27167100	-0.87392900	2.21155100
N	1.51713500	-0.27659100	2.36276700
C	1.45144700	0.98687100	1.87437300

N	0.23854200	1.23481500	1.41399500
C	-0.50339400	0.08631000	1.61618400
H	-1.54352900	0.02379500	1.34055900
H	2.33531800	-0.70578900	2.77164500
H	2.27192700	1.68695900	1.87631600
C	3.21746000	3.23383400	-2.78582400
H	2.75755800	2.62458600	-2.00155700
H	3.50342800	4.19922500	-2.36193500
N	2.30603300	3.42397900	-3.90930500
H	2.31624500	2.73840200	-4.65703500
C	1.20975300	4.19999800	-3.85745600
N	0.97542700	5.02355200	-2.83584000
H	0.07907000	5.50223300	-2.85270100
H	1.39100300	4.87897700	-1.90772500
N	0.37137300	4.20050200	-4.91119200
H	-0.51531300	4.69253800	-4.82028800
H	0.55389200	3.57250700	-5.68694300
Fe	-0.44165900	3.11660400	0.51921200
O	-0.99799600	4.53971500	-0.27682000
C	-3.93027500	5.57802700	-0.51637600
C	-2.73040100	5.84560800	-1.40158200
N	-3.72895800	6.18667700	0.82903200
S	-3.02557900	5.54934700	-3.14720100
O	-4.06940300	6.57788300	-3.53161400
O	-1.71876500	5.83267500	-3.81747800
O	-3.52124500	4.15797100	-3.29678100
H	-4.09148100	4.50688400	-0.38196100
H	-4.84441500	6.01131200	-0.93426400
H	-2.31596500	6.85203000	-1.29716500
H	-1.80099200	5.08025400	-0.96717100
H	-3.68915300	7.22611100	0.75030300
H	-2.83311500	5.91154500	1.26601100
H	-4.50764700	5.88031700	1.48446800
C	1.95978100	4.79345200	0.90728800
O	1.13671900	3.85455600	1.31023700
O	2.16467400	5.10874700	-0.26409300
C	2.68681800	5.49198800	2.04514300
H	3.53017900	6.06715900	1.65809000
H	3.02949600	4.76900600	2.79166100
O	1.49424100	1.98550400	-6.46569400
H	0.83482700	1.27701700	-6.54454000
H	1.90457700	2.05038000	-7.34225000
O	-5.71619300	5.09476900	2.33071200
H	-6.66769800	5.28144900	2.16252800
H	-5.64968500	4.93683600	3.29823600
H	-1.11036300	-2.45026000	2.51722800
H	-4.56377300	-0.77446400	7.38946000
H	1.98730800	6.17418300	2.54285700
H	4.11623400	2.74004500	-3.16201300
H	5.37571100	12.79621800	1.41273400
H	-1.33399100	12.80382900	-4.91601200
H	1.40811500	14.28088300	-1.87646100
H	-4.75005200	10.84972800	3.80675600
H	-4.80986200	8.01731100	9.24455900
H	-5.51273800	-1.70882000	-0.90847000
H	-9.37595800	2.78370200	-8.41414300
H	-10.91525500	12.03558000	0.43069600
H	-10.95497600	3.14352900	0.76054200
H	-10.29257900	7.57626300	1.15480900
H	-1.54229200	11.42925600	-2.22460500

^{5}TS1$_{HA,C1R,C}$

C	-0.63735600	11.29145600	-4.69086800
H	-0.53308700	10.95803700	-5.72608700
H	0.34807800	11.25255600	-4.21358200
C	-1.60978800	10.42127600	-3.96778900
N	-1.85018600	10.53579400	-2.60554200
C	-2.74939100	9.62336900	-2.21392300
H	-3.07870600	9.47681400	-1.18638600
N	-3.11169600	8.92994600	-3.29491200
H	-3.70984300	8.07586200	-3.29811200
C	-2.41502600	9.39968900	-4.39126500
H	-2.54059200	8.96524600	-5.36978300
C	2.33586100	13.26460400	-1.92265100
H	3.19009400	13.55743200	-1.29805100
H	1.69294200	14.14545700	-2.02388800
C	1.58816600	12.09749400	-1.31782000
C	0.46584600	12.29519800	-0.50084500
H	0.11749400	13.30822200	-0.30867600
C	-0.20175000	11.22393300	0.10718700
H	-1.03138800	11.39702600	0.78817000
C	0.24432700	9.90880000	-0.11484600
O	-0.38691900	8.82918500	0.41753700
H	-1.33387100	9.06416400	0.68409200
C	1.36964200	9.69224500	-0.92366200
H	1.70694000	8.67198300	-1.08469900
C	2.02488500	10.77496600	-1.50852800
H	2.89826500	10.58875500	-2.13028200
C	5.91040500	11.47531900	0.39737900
H	4.87501000	11.66856800	0.07949800
C	6.75311900	11.07751200	-0.82091000
H	6.36635600	10.17526600	-1.30863900
H	6.76603500	11.87737600	-1.57041700
H	7.79266300	10.87750500	-0.53253100
C	5.89817500	10.43323500	1.52883900
H	6.93066000	10.24480500	1.85624900
H	5.37793200	10.86271000	2.39569100
C	5.23006800	9.10404000	1.15533000
H	5.20422300	8.42302600	2.01451900
H	4.19640600	9.26225900	0.82235900
H	5.76454300	8.59114900	0.34759000
C	-5.25688500	10.15392000	3.59784600
H	-6.33552700	10.31977600	3.62624600
C	-4.83937400	9.81453200	2.15480000
H	-5.25872600	8.84959900	1.85222800
H	-5.28851200	10.55313700	1.47888100
C	-3.32584800	9.80102600	1.90375500
O	-2.54409500	10.36575200	2.68851100
O	-2.95564500	9.16731200	0.83279100
C	-4.94119000	9.00928000	4.54792200
O	-5.81901800	8.22549300	4.94487000
N	-3.63923900	8.89041600	4.91893500
H	-3.01409200	9.54244400	4.44234500
C	-3.02952600	7.65113600	5.42096400
H	-3.40882100	6.81119700	4.82126900
C	-1.51021500	7.71582000	5.21991700
H	-1.11605100	8.66843100	5.59391800
H	-1.07105700	6.92428100	5.83555200
C	-1.11380600	7.46157400	3.77031200
O	-1.65683200	6.56570800	3.11641700
N	-0.10145100	8.21855300	3.27944400
H	0.02340000	8.22581900	2.26550200
H	0.11900000	9.08999300	3.74260500
C	-3.32596500	7.25430800	6.88331500

O	-2.57237900	6.46812500	7.46640300
N	-4.45512700	7.73589300	7.45299300
H	-5.15850500	8.12563200	6.82980300
C	-4.86476300	7.27141600	8.77429700
H	-3.96356200	7.15308800	9.38178400
C	-5.65453700	5.95233700	8.72427300
O	-6.84193200	5.90325800	9.04734500
N	-4.96897100	4.87267300	8.25964100
H	-4.00600100	4.98233600	7.94241100
C	-5.63274700	3.60194300	8.02852900
H	-6.03262200	3.22695600	8.97894200
C	-6.84652300	3.69967300	7.03878800
H	-7.07785100	2.70392600	6.64757800
H	-7.70416800	4.07073500	7.60040400
C	-6.53597600	4.61806600	5.86470000
O	-5.78183600	4.25874200	4.94396500
N	-7.12662900	5.82726700	5.89814400
H	-6.79369900	6.56059700	5.27440100
H	-7.58017200	6.12387200	6.75379400
C	-4.62369000	2.59632800	7.46745400
O	-3.66128700	2.93708200	6.78222900
N	-4.90975800	1.28876500	7.74840000
H	-5.82544600	1.10939600	8.14288500
C	-4.43255500	0.24644500	6.85076500
H	-3.38242200	0.43724600	6.62264800
C	-5.30963900	0.25323200	5.58384500
O	-6.50059000	0.55442400	5.65697800
N	-4.69825200	-0.03971800	4.41251600
H	-3.71438000	-0.27585000	4.35398500
C	-5.37264100	0.18771500	3.14770100
H	-6.39669900	-0.19162500	3.23035900
C	-5.45742500	1.72025200	2.83656900
H	-6.00280600	1.86550600	1.89739300
H	-6.04781500	2.18248900	3.63146600
C	-4.12463500	2.38906900	2.71559300
N	-3.40675400	2.95291100	3.75975100
H	-3.73965200	3.09963500	4.71375300
C	-2.23073400	3.40274400	3.27171800
H	-1.50043900	3.93590000	3.85935000
N	-2.14209900	3.14789200	1.97028300
C	-3.32208100	2.52337000	1.61508800
H	-3.51465900	2.20290700	0.60559000
C	-4.61244300	-0.57998400	2.05666300
O	-3.46495400	-0.98386300	2.23729500
N	-5.30342100	-0.76581200	0.89611200
H	-6.16925000	-0.25430100	0.79032100
C	-4.64753500	-1.14730500	-0.34166000
H	-3.76722200	-1.74022000	-0.08703900
C	-4.27098300	0.09648000	-1.16956400
O	-4.86457500	1.16529900	-0.98028800
N	-3.27589700	-0.08192100	-2.06439000
H	-3.01483400	-1.02869500	-2.31900600
C	-2.83857800	0.95244600	-2.99241900
H	-3.36681500	1.86582600	-2.72175700
C	-1.31611400	1.19876900	-2.89788200
H	-0.76874900	0.25075300	-2.97771000
H	-0.99024900	1.81946400	-3.74036700
C	-0.90189500	1.91256000	-1.62071600
O	-1.76564900	2.04758900	-0.69590500
O	0.25311600	2.39929100	-1.48041600
C	-3.20482900	0.51682300	-4.42325900
O	-3.16012200	-0.66483000	-4.75373600
N	-3.52994100	1.53869100	-5.26036600

H	-3.58250900	2.47686400	-4.86882700
C	-3.99545500	1.33375300	-6.63916100
H	-4.17538100	2.34802100	-7.02032500
C	-2.97962200	0.64513000	-7.59062700
H	-3.06151600	-0.43425100	-7.43573500
C	-3.32423800	0.96175700	-9.05581900
H	-3.23322200	2.03862700	-9.25718400
H	-2.63576500	0.43935500	-9.73022100
H	-4.34003400	0.64375700	-9.29478400
C	-1.53336800	1.06981400	-7.29140400
H	-1.41782400	2.16070500	-7.34699800
H	-1.21627800	0.73221600	-6.29820500
H	-0.86429400	0.62077500	-8.03679400
C	-5.37951600	0.61674000	-6.65613200
O	-5.71858200	-0.15144900	-7.54227300
N	-6.21773500	1.00022300	-5.63746100
H	-5.76101400	1.34304100	-4.79987400
C	-7.43435400	0.24454700	-5.35147000
H	-7.27616300	-0.81106200	-5.59828300
C	-7.75683400	0.39087400	-3.83567100
H	-8.18762800	1.39039200	-3.67755500
C	-8.72291300	-0.67792100	-3.33596400
H	-9.63322500	-0.69144200	-3.93962000
H	-8.24727100	-1.66275600	-3.39992700
H	-8.98542600	-0.48880500	-2.28803700
O	-6.50652600	0.31007900	-3.13325800
H	-6.51645700	0.89740400	-2.36168000
C	-8.65593200	0.70905300	-6.17331700
O	-9.68504500	0.04049800	-6.19838200
N	-8.51463600	1.91529500	-6.78182600
H	-7.60647000	2.35744700	-6.73059200
C	-9.56793900	2.54681500	-7.55552700
H	-10.45167400	1.91256700	-7.45446000
C	-9.87193500	3.98030500	-7.06719500
H	-10.71196100	4.36445500	-7.65942700
H	-10.20646700	3.92921600	-6.02424700
C	-8.68425800	4.91293800	-7.19098200
C	-8.42860600	5.58934800	-8.39332100
H	-9.11939300	5.47692000	-9.22633500
C	-7.30607200	6.40594000	-8.53357600
H	-7.13107800	6.92510000	-9.47238500
C	-6.41167900	6.55661900	-7.47100800
H	-5.53595500	7.19109600	-7.58261300
C	-6.64955400	5.89204000	-6.26587200
H	-5.95680500	5.99578000	-5.43439400
C	-7.78153900	5.08216100	-6.12964500
H	-7.96690900	4.57626700	-5.18457800
C	-9.34400500	8.06042700	1.70238800
H	-9.74399300	7.93252000	2.71594800
C	-7.96246600	7.40426800	1.61765200
H	-7.49267900	7.64481700	0.65384300
H	-7.32278100	7.79165000	2.41099000
O	-8.01944500	5.98826400	1.81151700
H	-8.39835400	5.58131400	1.01164200
C	-9.23454300	9.54882700	1.37944100
O	-8.24755300	10.20827600	1.70317200
N	-10.29476500	10.07961600	0.70368500
H	-11.03932000	9.45710100	0.42153900
C	-10.28384300	11.42668700	0.15116700
H	-9.36927200	11.89239300	0.52274300
C	-10.33957000	11.43935300	-1.39048100
H	-11.33731600	11.11932300	-1.71850600
H	-10.24098900	12.48551500	-1.71351800

C	-9.29520400	10.57460000	-2.07356100
C	-9.67475200	9.63937000	-3.04621500
H	-10.72583700	9.54302400	-3.31062800
C	-8.72730800	8.83785400	-3.68990400
H	-9.04125400	8.12610200	-4.44895000
C	-7.37542400	8.95399600	-3.36078900
H	-6.63874000	8.32506400	-3.85300000
C	-6.98494500	9.88313700	-2.39282600
H	-5.93517500	9.98717200	-2.12896400
C	-7.93259500	10.68691400	-1.75856000
H	-7.60960500	11.39349600	-0.99910600
C	-10.02785300	2.50379700	0.52000000
H	-9.83820900	2.71062100	1.58167700
H	-9.84029500	1.43721200	0.35645100
C	-9.15664500	3.36075500	-0.36946500
C	-9.59679700	4.61741600	-0.81751800
H	-10.59639500	4.95538200	-0.55096100
C	-8.78119500	5.42679300	-1.61754400
H	-9.14192700	6.39389600	-1.95850900
C	-7.50927000	4.98756300	-1.99614600
H	-6.87201400	5.61257700	-2.61494900
C	-7.06246900	3.73411900	-1.56711900
H	-6.08526900	3.36694100	-1.86762700
C	-7.87697700	2.93734700	-0.75792200
H	-7.50593600	1.97100800	-0.42295900
C	0.08187700	-2.10773500	2.61129600
H	0.35382400	-2.25867100	3.66374500
H	0.64504500	-2.83769700	2.01569900
C	0.33308800	-0.70458200	2.16720700
N	1.56492800	-0.07025800	2.26930900
C	1.44049000	1.19213600	1.78996400
N	0.20246500	1.40573600	1.38121200
C	-0.49435000	0.23438700	1.60923000
H	-1.54308300	0.13911100	1.37982100
H	2.41141400	-0.47484700	2.64450400
H	2.24070400	1.91482600	1.76112600
C	3.11862100	3.74238300	-2.84096400
H	2.72978400	3.06296900	-2.07527000
H	3.36087800	4.69955900	-2.37319900
N	2.15558700	3.93028900	-3.92115900
H	2.19929300	3.30184100	-4.71658200
C	0.98906400	4.58340800	-3.76932000
N	0.73119200	5.32094400	-2.69146500
H	-0.22192800	5.65933400	-2.59172000
H	1.23924700	5.19772000	-1.80716300
N	0.09753100	4.54743900	-4.77841200
H	-0.82003600	4.95992000	-4.62367100
H	0.29127800	3.96785100	-5.58822100
Fe	-0.54926800	3.29064700	0.50340300
O	-1.21839700	4.77586500	-0.28058500
C	-3.64662800	6.44455200	-0.54959100
C	-3.41950300	5.15081500	-1.29633800
N	-3.26479100	6.36088500	0.88522200
S	-3.43607900	5.39706800	-3.08965100
O	-4.46128700	6.48270600	-3.31149800
O	-2.07284400	5.89028500	-3.46012000
O	-3.80481700	4.09231600	-3.68693200
H	-4.68966400	6.76698800	-0.60951000
H	-3.01672500	7.22230000	-0.97942200
H	-2.21704200	4.78441900	-0.95961500
H	-4.04844300	4.30900700	-1.00271300
H	-3.10952500	7.33643700	1.20255000
H	-2.36827200	5.85627400	0.98667900
H	-4.00580200	5.92952000	1.49845500
C	1.83178500	5.05522400	0.94952700
O	1.00410400	4.09259900	1.28325800
O	2.04734800	5.43635900	-0.20084400
C	2.55171700	5.67816500	2.13272500
H	3.34892300	6.33814900	1.78581100
H	2.96221000	4.90016700	2.78443300
O	1.36086500	2.53318700	-6.49517700
H	0.82662700	1.72512200	-6.56535600
H	1.72529700	2.67456100	-7.38301300
O	-5.36129000	5.59164800	2.48053300
H	-6.29532800	5.58634000	2.17368100
H	-5.38952900	5.17841300	3.37135200
H	-0.98295300	-2.33042900	2.50441300
H	-4.51084000	-0.72123400	7.35634900
H	1.83535600	6.25685700	2.72690600
H	4.03174800	3.32785500	-3.27339300
H	6.29013200	12.42492600	0.79779600
H	-0.96597700	12.33727700	-4.70268200
H	2.73443700	13.02141800	-2.91421500
H	-4.73991900	11.06563800	3.91452000
H	-5.51660800	8.01725200	9.23149400
H	-5.32706500	-1.76685100	-0.93843600
H	-9.28889800	2.57075000	-8.61764300
H	-11.12941000	12.00245100	0.54791600
H	-11.09139000	2.68877900	0.33780500
H	-10.04004200	7.56138700	1.01566600
H	-1.33589700	11.12876900	-1.95067100

^{5}TS1$_{HA,C2S,C}$

C	-0.21102400	11.55134900	-4.78014700
H	-0.10594300	11.36010100	-5.85072000
H	0.77203600	11.43463100	-4.31077700
C	-1.19940300	10.60553800	-4.18432500
N	-1.44298100	10.53613500	-2.82035200
C	-2.36057900	9.59508800	-2.56166200
H	-2.71685700	9.32867100	-1.57024800
N	-2.72908800	9.06074600	-3.72577700
H	-3.36185900	8.23127000	-3.83568100
C	-2.02027400	9.66531200	-4.74558600
H	-2.15349100	9.37828100	-5.77636400
C	2.58555100	12.98541700	-1.61176200
H	3.05186200	12.78687200	-2.58356900
H	3.39687900	13.22372100	-0.91135600
C	1.77305000	11.80487900	-1.12853600
C	0.59507100	11.98456400	-0.38880300
H	0.25271000	12.99373100	-0.16734700
C	-0.14010200	10.89768600	0.10436200
H	-1.02237600	11.05048000	0.72105500
C	0.29579500	9.58773700	-0.16027600
O	-0.39291200	8.49053500	0.25631400
H	-1.36006000	8.71071400	0.44085400
C	1.48010500	9.38790900	-0.88519200
H	1.80994400	8.36929800	-1.07035300
C	2.19905200	10.48461700	-1.35734500
H	3.11414000	10.31224000	-1.92017300
C	5.84559000	11.54854600	1.03628900
H	4.87211400	11.62591500	0.52944800
C	6.94727900	11.31275500	-0.00414000
H	6.78119900	10.39183600	-0.57484600
H	6.99659100	12.14001100	-0.72163800
H	7.93061900	11.23186900	0.47568400
C	5.75607300	10.46378700	2.12302800

H	6.72574600	10.38671500	2.63533900
H	5.03442300	10.78816900	2.88500300
C	5.34264000	9.08138800	1.60232000
H	5.25388100	8.36270200	2.42570600
H	4.37228800	9.12748800	1.09121100
H	6.07353900	8.67701800	0.89277200
C	-5.32429700	9.86691000	3.24979200
H	-6.40496100	10.02115900	3.25859800
C	-4.90241100	9.35104800	1.85984700
H	-5.24467200	8.31932900	1.72014900
H	-5.42413400	9.93907600	1.09462900
C	-3.39812400	9.41859200	1.56182000
O	-2.63067100	10.04852900	2.31216800
O	-3.01699300	8.78197400	0.49610800
C	-4.98796300	8.85748800	4.33469600
O	-5.84697200	8.10201300	4.81836800
N	-3.68551100	8.81623300	4.72348600
H	-3.07671300	9.41748400	4.16526400
C	-3.04617000	7.64346600	5.33839600
H	-3.40484800	6.74413400	4.81715600
C	-1.53057000	7.72393300	5.12281400
H	-1.15417000	8.71616500	5.39891200
H	-1.06921800	7.00320700	5.80577000
C	-1.14961900	7.34419200	3.69882400
O	-1.70852900	6.39234300	3.14029800
N	-0.14882100	8.04722000	3.12414800
H	-0.03996200	7.96926400	2.11018500
H	0.09980500	8.94527500	3.51645100
C	-3.33403200	7.35476300	6.82690200
O	-2.54887900	6.65258700	7.47246400
N	-4.49862200	7.80814700	7.34495500
H	-5.20668300	8.11862000	6.68199000
C	-4.91505800	7.38856600	8.67873600
H	-4.02145800	7.33285800	9.30609400
C	-5.65169100	6.03778400	8.67721600
O	-6.83001800	5.95113900	9.02470900
N	-4.93534800	4.97247700	8.22504500
H	-3.97433200	5.10171800	7.91175000
C	-5.55229700	3.67059600	8.03873600
H	-5.90871100	3.29739900	9.00728800
C	-6.79780900	3.69732300	7.08482300
H	-6.99874000	2.68458100	6.72163500
H	-7.65253500	4.04521700	7.66536700
C	-6.56596300	4.59883200	5.87938000
O	-5.83525400	4.24461000	4.93846800
N	-7.20187000	5.78567700	5.90663400
H	-6.91180100	6.51284900	5.25513900
H	-7.63393200	6.08653200	6.77197900
C	-4.51793900	2.69534600	7.46899600
O	-3.57810100	3.06558900	6.76626900
N	-4.74967400	1.38196200	7.76667900
H	-5.64960700	1.17182500	8.18162200
C	-4.24348700	0.34332500	6.88012500
H	-3.20745600	0.57661200	6.62739800
C	-5.14490400	0.28639500	5.63056900
O	-6.34190000	0.55434300	5.72154500
N	-4.55008300	-0.02159200	4.45263500
H	-3.55689700	-0.20902400	4.37947000
C	-5.24596500	0.21303000	3.19940400
H	-6.27220100	-0.15258500	3.30515200
C	-5.31241200	1.74755400	2.89212600
H	-5.85951000	1.90300300	1.95657700
H	-5.88898600	2.21884500	3.69191800
C	-3.97120000	2.39854900	2.76365600
N	-3.24317400	2.95535500	3.80483400
H	-3.55790800	3.09116500	4.76736000
C	-2.07019000	3.40748300	3.30685200
H	-1.33358300	3.93205600	3.89326500
N	-1.99523900	3.17038500	2.00209800
C	-3.17446500	2.53829200	1.65853500
H	-3.37579000	2.22533800	0.64840100
C	-4.52082100	-0.54406700	2.08163600
O	-3.34928000	-0.89988600	2.20079800
N	-5.26361700	-0.75531900	0.95732000
H	-6.15646300	-0.28334700	0.90541400
C	-4.64913900	-1.05979000	-0.32240400
H	-3.77004300	-1.68072500	-0.14051300
C	-4.28226100	0.23425500	-1.07396800
O	-4.86102700	1.29152100	-0.80369400
N	-3.29988900	0.11385200	-1.99580300
H	-3.06250900	-0.81712500	-2.32338900
C	-2.93563600	1.19127100	-2.90937000
H	-3.42382400	2.09750600	-2.55352700
C	-1.41193900	1.41171600	-2.95734900
H	-0.89220000	0.46320200	-3.14080100
H	-1.17053300	2.06427600	-3.80500700
C	-0.80925500	2.07259200	-1.72591700
O	-1.60026500	2.34277400	-0.74727900
O	0.40127000	2.36726700	-1.68384100
C	-3.42323400	0.81557400	-4.31946800
O	-3.35926400	-0.34664100	-4.71483300
N	-3.86877600	1.85777500	-5.06692900
H	-3.93200500	2.77564700	-4.62664800
C	-4.40566300	1.69068100	-6.42297600
H	-4.69888800	2.70385000	-6.72926900
C	-3.39892600	1.14697200	-7.47538900
H	-3.40212600	0.05578000	-7.39957100
C	-3.84839800	1.54223500	-8.89217200
H	-3.83920300	2.63409000	-9.01780300
H	-3.17019500	1.11584700	-9.64073000
H	-4.85480400	1.17371500	-9.09647500
C	-1.96768700	1.64485700	-7.21970800
H	-1.91764400	2.74154300	-7.19443500
H	-1.57371700	1.25792200	-6.27340000
H	-1.31594400	1.30001200	-8.03316300
C	-5.72015500	0.85639500	-6.40113400
O	-6.06862300	0.14432300	-7.33064900
N	-6.50616400	1.07713300	-5.29822800
H	-6.03151100	1.39102700	-4.46026900
C	-7.67068500	0.24147300	-5.02972400
H	-7.45311500	-0.79815000	-5.29936000
C	-8.00842900	0.33614500	-3.51247100
H	-8.50710000	1.29977700	-3.34011100
C	-8.90217800	-0.80761500	-3.04702300
H	-9.79696000	-0.87690800	-3.67008100
H	-8.35514900	-1.75427700	-3.11846500
H	-9.19584100	-0.65721000	-2.00171100
O	-6.76887700	0.32423300	-2.77966900
H	-6.78414000	1.02379500	-2.10901300
C	-8.91591900	0.65301900	-5.84706300
O	-9.89399800	-0.08470400	-5.91880900
N	-8.86041900	1.89901700	-6.38422800
H	-7.97091000	2.37885300	-6.34877500
C	-9.92218300	2.46636600	-7.19577900
H	-10.83803900	1.92228300	-6.95226100
C	-10.09183900	3.97537500	-6.92399300

H	-10.93182100	4.33329300	-7.53207100
H	-10.36954300	4.11389500	-5.87233900
C	-8.84521700	4.77717500	-7.24328600
C	-8.58895400	5.20891700	-8.55320900
H	-9.31754500	5.00476200	-9.33494100
C	-7.41626700	5.89727400	-8.86570400
H	-7.24055300	6.22677200	-9.88646800
C	-6.47263900	6.16345000	-7.87040000
H	-5.55966600	6.70050700	-8.11435500
C	-6.71024300	5.74320100	-6.55975100
H	-5.98089100	5.94755900	-5.77955300
C	-7.89149300	5.05942300	-6.25263900
H	-8.07624100	4.73979900	-5.22912800
C	-9.53220800	8.02772200	1.51415600
H	-10.01563200	7.92253500	2.49335100
C	-8.17454800	7.31827100	1.54129700
H	-7.62927600	7.51671000	0.60876300
H	-7.57664800	7.70099000	2.36855400
O	-8.29801300	5.90912800	1.75972600
H	-8.64200500	5.50108500	0.94513500
C	-9.33469500	9.50900500	1.20066100
O	-8.36403700	10.13303700	1.62934100
N	-10.29247100	10.07743900	0.41358800
H	-11.02237300	9.48271100	0.04622300
C	-10.16636100	11.42130900	-0.13359700
H	-9.28654200	11.85510500	0.34454300
C	-10.03868000	11.43267400	-1.67101900
H	-11.00737700	11.16989000	-2.11615100
H	-9.84470600	12.47145500	-1.97504000
C	-8.97393900	10.50765100	-2.23429200
C	-9.27561900	9.65732900	-3.30715100
H	-10.27709600	9.67212000	-3.73262000
C	-8.31228200	8.79903800	-3.84454500
H	-8.56479400	8.15595100	-4.68399000
C	-7.02450800	8.76724800	-3.30521400
H	-6.27452200	8.09389600	-3.71053800
C	-6.71265100	9.61005200	-2.23471000
H	-5.71595200	9.59226000	-1.79879700
C	-7.67325700	10.47516000	-1.70884200
H	-7.41410500	11.10971100	-0.86615600
C	-10.01380500	2.64166300	0.66486800
H	-9.90517600	3.06203900	1.67352900
H	-9.72250500	1.58746000	0.71766500
C	-9.16941400	3.40026700	-0.33367800
C	-9.67575500	4.53350200	-0.99240200
H	-10.70525600	4.83641600	-0.81193700
C	-8.88508500	5.26208600	-1.88879700
H	-9.29304300	6.13835100	-2.38553900
C	-7.57382300	4.85950800	-2.15402300
H	-6.96078500	5.41333200	-2.85882200
C	-7.05408600	3.73299800	-1.50858900
H	-6.03621100	3.41146300	-1.70672300
C	-7.84516000	3.01952400	-0.60374100
H	-7.41983000	2.15657500	-0.09607200
C	0.12513800	-2.05976100	2.17770000
H	0.47709100	-2.29471600	3.19010500
H	0.60701000	-2.76325500	1.48676500
C	0.39747200	-0.63820400	1.81212000
N	1.65632700	-0.05097800	1.87134000
C	1.55147800	1.24367400	1.49299700
N	0.29483800	1.52142500	1.18824700
C	-0.43219800	0.36129900	1.37981100
H	-1.49628800	0.31766900	1.21943900
H	2.51085900	-0.50800400	2.15787000
H	2.36923800	1.94472200	1.45948600
C	3.18362900	3.81645500	-2.94557400
H	2.81583400	3.10854500	-2.19568600
H	3.41110100	4.76595700	-2.45521300
N	2.20571300	4.01684800	-4.00997700
H	2.23105600	3.38360200	-4.80193400
C	1.03158900	4.64880500	-3.82400900
N	0.77998800	5.35495000	-2.72340900
H	-0.17914500	5.66654100	-2.59740400
H	1.28982300	5.20285200	-1.84522500
N	0.13266400	4.63840200	-4.82855700
H	-0.80703000	4.97117900	-4.62005500
H	0.29986000	4.02751900	-5.62281700
Fe	-0.39714100	3.32961900	0.49483300
O	-0.85778700	4.83969700	-0.17153200
C	-3.25828400	5.25370000	-0.36833100
C	-3.85799100	6.12369400	-1.45258100
N	-3.37046400	5.96447000	0.93900500
S	-3.53926600	5.65542500	-3.17624800
O	-4.21530600	6.76304500	-3.94494400
O	-2.05799100	5.68810200	-3.35919400
O	-4.16077500	4.32396700	-3.40158500
H	-2.00247200	5.02654400	-0.45245700
H	-3.71809700	4.27017900	-0.29491500
H	-4.95229300	6.15833700	-1.37118200
H	-3.48943900	7.14710100	-1.35824200
H	-3.31250800	6.99724600	0.79572000
H	-2.58038600	5.74785400	1.57047700
H	-4.26068700	5.73877400	1.46325900
C	1.91783600	5.05057900	0.94308400
O	1.14873400	4.05975300	1.32818700
O	2.07430100	5.42901600	-0.21825100
C	2.67101500	5.70317900	2.09074000
H	3.42925300	6.38762600	1.70609300
H	3.13697400	4.94294300	2.72584100
O	1.23805700	2.56771200	-6.56721700
H	0.64979400	1.79572400	-6.59919400
H	1.52132500	2.70726300	-7.48444000
O	-5.63394500	5.41667200	2.38625600
H	-6.57812000	5.44675000	2.11434600
H	-5.63271700	5.08426600	3.31203400
H	-0.95340000	-2.23435100	2.14766800
H	-4.26725700	-0.61598100	7.40668300
H	1.96243600	6.25958100	2.71472200
H	4.10071300	3.43350000	-3.39845700
H	6.01629900	12.51901500	1.52156300
H	-0.52455900	12.59393500	-4.65147700
H	1.96941400	13.88561100	-1.70992900
H	-4.82288900	10.82024700	3.44616000
H	-5.60426000	8.12632700	9.09217300
H	-5.35881700	-1.62175300	-0.93938200
H	-9.71554100	2.30462900	-8.26341400
H	-11.03140500	12.02837900	0.16106000
H	-11.07681300	2.68434300	0.40638500
H	-10.19086700	7.55330000	0.77515200
H	-0.94218300	11.04331100	-2.08735900

^{5}TS1$_{HA,C2R,C}$

C	0.78221000	11.16302700	-5.13990600
H	0.95390100	10.92424300	-6.19223400
H	1.75378700	11.30030800	-4.65312400
C	0.00429700	10.06956900	-4.48691800
N	-0.24537000	10.03463700	-3.12128300
C	-0.96415000	8.94861100	-2.80643900
H	-1.31832800	8.67882600	-1.80802500
N	-1.19066800	8.28149600	-3.93893900
H	-1.67420400	7.36293700	-4.00150800
C	-0.59879900	8.95110200	-4.99363400
H	-0.65503900	8.58138600	-6.00521200
C	3.11778700	13.70222500	-2.63127200
H	3.76252300	13.28767400	-3.41497900
H	3.74415700	14.36668500	-2.02180200
C	2.48824000	12.61510800	-1.78950700
C	1.17303100	12.72201100	-1.32013000
H	0.58072000	13.59630000	-1.58262400
C	0.60020300	11.74308500	-0.49636600
H	-0.41117700	11.85880000	-0.11651100
C	1.35427700	10.61677500	-0.11683200
O	0.84795100	9.64333700	0.67706900
H	-0.14894900	9.76985900	0.81576000
C	2.67070400	10.49280700	-0.58820400
H	3.25042000	9.62055600	-0.30099300
C	3.21856600	11.47714200	-1.40603000
H	4.24350500	11.36197100	-1.75242600
C	0.30899900	13.75179900	3.08412200
H	0.88044200	13.36756700	2.22712900
C	1.26354700	14.42810700	4.07569700
H	2.02026300	13.73279200	4.45633300
H	1.79473400	15.26294600	3.60427700
H	0.71639100	14.82781100	4.93884000
C	-0.52761600	12.60999400	3.68662600
H	-1.09468000	12.99680500	4.54565200
H	-1.26900200	12.28456600	2.94429500
C	0.29000200	11.38745900	4.12320300
H	-0.36097900	10.62398000	4.56770800
H	0.80559500	10.93646300	3.26562000
H	1.04585700	11.64308700	4.87393200
C	-4.16028500	9.91648700	2.58074700
H	-5.19401300	10.20377700	2.77716300
C	-4.03671800	9.48668400	1.10676900
H	-4.63426800	8.58978800	0.91571100
H	-4.46970800	10.27820600	0.48262200
C	-2.60012200	9.24203700	0.63594600
O	-1.69465500	9.97615300	1.13774500
O	-2.40720200	8.33683500	-0.23168300
C	-3.79254300	8.76982500	3.50475800
O	-4.64784500	8.07545600	4.07662800
N	-2.46017000	8.52759600	3.63990900
H	-1.85292500	9.13546600	3.09575500
C	-1.87716800	7.26997100	4.12342600
H	-2.45511100	6.43517900	3.70139600
C	-0.42918800	7.14615800	3.61900600
H	0.06427400	8.12378500	3.58219000
H	0.10989200	6.53962600	4.35635800
C	-0.31841900	6.39897900	2.29799200
O	-1.00056300	5.37320100	2.11090100
N	0.58521700	6.83325400	1.40477500
H	0.74336600	6.27548600	0.56701800
H	0.97526100	7.77188000	1.42246800
C	-1.92577600	7.01871100	5.65205500
O	-1.12937500	6.23542500	6.17450800
N	-2.91662000	7.63025000	6.34407900
H	-3.68956100	8.01515900	5.80582200
C	-3.13156400	7.32880100	7.75371700
H	-2.15879000	7.09787200	8.19510100
C	-4.11212000	6.16509700	7.96731700
O	-5.22715600	6.34632400	8.45682600
N	-3.68401700	4.94273100	7.54837300
H	-2.78098100	4.83794000	7.09025700
C	-4.55783600	3.78425900	7.61228000
H	-4.84904200	3.61523500	8.65670900
C	-5.88773600	3.95416500	6.80083100
H	-6.31078100	2.96856800	6.58043600
H	-6.58825600	4.51073400	7.42386200
C	-5.66637900	4.67071900	5.47374900
O	-5.10612800	4.10674500	4.51921800
N	-6.13628600	5.93082100	5.41568200
H	-5.90463600	6.52053300	4.62024200
H	-6.40559900	6.39543400	6.27379500
C	-3.80952500	2.55469500	7.09383600
O	-2.86935000	2.64134700	6.30355100
N	-4.29368000	1.35884500	7.54217500
H	-5.17978800	1.39340500	8.03204800
C	-4.10341000	0.15651900	6.74044000
H	-3.06723500	0.12767900	6.39845400
C	-5.10212000	0.18773800	5.56466700
O	-6.20831600	0.70690700	5.70893000
N	-4.67753700	-0.33253200	4.38993600
H	-3.75408300	-0.73599100	4.27681300
C	-5.40625800	-0.09584800	3.15518300
H	-6.46702500	-0.30817300	3.32545800
C	-5.27495900	1.40341500	2.72032000
H	-5.86294900	1.56851400	1.81157600
H	-5.71381500	2.01545800	3.51154300
C	-3.86034000	1.80790900	2.45526400
N	-2.95244100	2.22960100	3.41570300
H	-3.15560100	2.49767300	4.37937900
C	-1.74490300	2.36532300	2.82807800
H	-0.86847500	2.72776500	3.34140600
N	-1.82097500	2.04400300	1.54337700
C	-3.13621800	1.71156800	1.29747100
H	-3.47234800	1.41658800	0.31870300
C	-4.83702500	-1.04084500	2.08557300
O	-3.75903700	-1.61167000	2.24405800
N	-5.60889900	-1.17548500	0.96815000
H	-6.37752100	-0.52523800	0.85962400
C	-5.07536600	-1.70618400	-0.27149800
H	-4.18025200	-2.28212900	-0.02621200
C	-4.73897000	-0.56363100	-1.25105300
O	-5.14693800	0.58095700	-1.02087700
N	-3.98715700	-0.90434000	-2.31702900
H	-3.83045400	-1.88417100	-2.51673200
C	-3.39933100	0.06060000	-3.23345100
H	-3.63983400	1.04760900	-2.83678400
C	-1.85951300	-0.09493800	-3.29033300
H	-1.58882300	-1.15504900	-3.38895700
H	-1.45963200	0.41648500	-4.16953100
C	-1.17747400	0.49389300	-2.06508500
O	-1.83264900	0.55739900	-0.97802600
O	-0.00758000	0.96585700	-2.12892700
C	-4.02480400	-0.07403900	-4.64057200
O	-4.55554800	-1.11704500	-5.00616800
N	-3.87572200	1.05132900	-5.39462200

H	-3.53362500	1.87716700	-4.90242500
C	-4.58850400	1.32084900	-6.65688900
H	-4.18001900	2.28088900	-7.00107200
C	-4.36851200	0.27495700	-7.76934000
H	-4.96980800	-0.60210700	-7.51685600
C	-4.85614300	0.83357200	-9.11588400
H	-4.28651600	1.72690800	-9.40813400
H	-4.72942000	0.08613800	-9.90704100
H	-5.91572400	1.09592600	-9.07204000
C	-2.89607900	-0.15487300	-7.86499700
H	-2.23610800	0.71354700	-8.00932800
H	-2.58703300	-0.69895400	-6.96448800
H	-2.75501600	-0.82412900	-8.72106000
C	-6.09826900	1.54580300	-6.34323400
O	-6.98805700	0.83683900	-6.79000200
N	-6.32925000	2.61469500	-5.51272700
H	-5.53492800	3.08339100	-5.08869000
C	-7.57500700	2.77003400	-4.77404700
H	-8.33597600	2.21462300	-5.33418000
C	-7.48455700	2.16696000	-3.35389500
H	-8.31541200	2.59108900	-2.78021300
C	-7.61954200	0.64389700	-3.36073100
H	-8.58583100	0.34814700	-3.78303400
H	-6.83513500	0.15613200	-3.94741400
H	-7.57319300	0.26311900	-2.33390700
O	-6.24292900	2.59549200	-2.77435300
H	-5.94237000	1.91839000	-2.13792800
C	-8.04324800	4.24467500	-4.74969300
O	-8.71469300	4.69199100	-3.81786200
N	-7.72498300	4.95217300	-5.85954200
H	-7.13184000	4.50118900	-6.54490700
C	-8.11471000	6.33488300	-6.08124000
H	-8.56025400	6.69332200	-5.15140000
C	-6.91985000	7.22154500	-6.48670600
H	-7.27263900	8.26109100	-6.50483400
H	-6.15061600	7.14395700	-5.71043500
C	-6.33603700	6.85760400	-7.83823600
C	-6.95486100	7.29110700	-9.02084400
H	-7.84019600	7.92129000	-8.96162800
C	-6.44849900	6.93187700	-10.26985700
H	-6.94098300	7.28112600	-11.17359200
C	-5.30836400	6.12896000	-10.35897500
H	-4.91192500	5.84936800	-11.33141500
C	-4.68190000	5.69361800	-9.19052400
H	-3.78968900	5.07443500	-9.25128500
C	-5.18999300	6.05482900	-7.93899300
H	-4.69437300	5.72204200	-7.03040000
C	-8.20782400	8.78320200	2.59917000
H	-7.48390000	8.86121900	3.41745400
C	-7.69147700	7.71926700	1.61995200
H	-8.42407500	7.51594100	0.83052300
H	-6.78443900	8.09839700	1.14548500
O	-7.34257500	6.51081700	2.30730200
H	-8.05786200	5.86015100	2.19878200
C	-8.27566100	10.13130700	1.88937000
O	-7.25907100	10.65471000	1.43220100
N	-9.51475700	10.68813700	1.77121300
H	-10.30440100	10.15340100	2.10608800
C	-9.78306600	11.80952600	0.87978900
H	-8.80873600	12.12904500	0.50469700
C	-10.72655900	11.42592600	-0.28325000
H	-11.73984300	11.27137200	0.11050100
H	-10.78816900	12.29063600	-0.95703900
C	-10.28511800	10.18559900	-1.03268900
C	-10.97453500	8.97595000	-0.87466200
H	-11.86607900	8.94803700	-0.25049400
C	-10.54184300	7.81012600	-1.51093400
H	-11.08836500	6.88187100	-1.37039200
C	-9.40507000	7.83202800	-2.32235600
H	-9.06947400	6.92004000	-2.80972800
C	-8.71678000	9.03696000	-2.49573900
H	-7.83423500	9.07131400	-3.13021700
C	-9.15227200	10.20018400	-1.85892800
H	-8.60188900	11.12813800	-1.99893600
C	-11.83151600	3.63594300	2.59143800
H	-11.51994400	3.88927400	3.61035600
H	-12.21867400	2.60858500	2.61617500
C	-10.68796400	3.76546100	1.61102200
C	-10.93060200	3.96679200	0.24457900
H	-11.95778900	4.05342400	-0.10438300
C	-9.88041300	4.05362600	-0.67284000
H	-10.07561800	4.21160300	-1.72958800
C	-8.55812900	3.94128400	-0.23554300
H	-7.74416000	3.99987200	-0.95234700
C	-8.29724700	3.73652000	1.12282800
H	-7.26905100	3.66067700	1.46776500
C	-9.35509900	3.65539200	2.03716200
H	-9.14525000	3.48672600	3.09213100
C	0.04613100	-3.25480100	2.51708100
H	0.41320600	-3.31131800	3.54979800
H	0.48817600	-4.09132300	1.96086200
C	0.35213000	-1.93610400	1.88715100
N	1.63717700	-1.42102300	1.76504100
C	1.55990800	-0.20904400	1.16189800
N	0.30227700	0.08256100	0.88385500
C	-0.45890000	-0.98284900	1.32909200
H	-1.53410000	-0.99104500	1.23581200
H	2.48730300	-1.86863700	2.07785300
H	2.40579800	0.42660900	0.95259600
C	2.84871000	2.59380500	-3.06442500
H	2.51238200	2.15896000	-2.11701600
H	3.08806200	3.64856000	-2.91194800
N	1.83462400	2.46837100	-4.10333200
H	1.72479800	1.55122700	-4.51807400
C	0.72860800	3.23491200	-4.15993100
N	0.55110600	4.27476600	-3.34095000
H	-0.30100500	4.81351200	-3.49679300
H	0.90625500	4.26782100	-2.37627600
N	-0.15981200	3.01341000	-5.14638600
H	-1.12903300	3.25724900	-4.93452300
H	-0.00423900	2.21509400	-5.76355600
Fe	-0.44673500	1.89717400	-0.08888700
O	-1.15539200	3.27749900	-0.83451400
C	-2.61598200	5.18319300	-1.20379800
C	-3.79306000	5.07076800	-2.14398600
N	-2.94272800	5.65841100	0.15639000
S	-3.23508900	4.77909200	-3.85614100
O	-2.77369800	3.35358100	-3.93619400
O	-4.38191100	5.08863800	-4.73215900
O	-2.04364700	5.69740100	-4.03467300
H	-1.83769700	5.83571600	-1.59746800
H	-1.98137700	4.07928200	-1.06352500
H	-4.49932900	4.26990500	-1.90253900
H	-4.35365400	6.01500500	-2.18760800
H	-3.81338300	5.28394500	0.61470400
H	-2.98215900	6.70885000	0.12345700

H	-2.15509800	5.42928600	0.80872100
C	1.80794700	3.82351600	0.26064700
O	1.30713100	2.62482700	0.36006200
O	1.55207000	4.64289500	-0.63366600
C	2.79788400	4.16466200	1.36538500
H	3.27270800	5.12857000	1.17618400
H	3.56085400	3.38252000	1.43749400
O	0.35123500	0.53197900	-6.58080500
H	-0.49413800	0.07397400	-6.72151000
H	0.77860500	0.53203700	-7.45167900
O	-5.06733800	5.00125400	1.79165300
H	-5.83873700	5.60735400	1.87154900
H	-4.91541600	4.69792800	2.71352100
H	-1.03725600	-3.40056400	2.53920300
H	-4.28872200	-0.72040000	7.36862200
H	2.27421100	4.19791700	2.32692000
H	3.75166400	2.08002300	-3.40307300
H	-0.37545200	14.50963300	2.67843800
H	0.24677600	12.11847500	-5.09263600
H	2.35919100	14.32734300	-3.11402200
H	-3.50792400	10.77709500	2.75715200
H	-3.56100800	8.20086900	8.24998000
H	-5.80232700	-2.38464600	-0.73588100
H	-8.88909500	6.38260800	-6.85934600
H	-10.21571700	12.64513800	1.44424400
H	-12.66940200	4.28776100	2.32058100
H	-9.17260900	8.47963100	3.02335000
H	0.08600600	10.71465700	-2.43075700

^{5}IM1$_{HA,C1,C}$

C	-0.67297100	11.92493000	-4.53804100
H	-0.56056100	11.81591900	-5.61935600
H	0.31477500	11.81682500	-4.07619000
C	-1.61869500	10.89647800	-4.01436700
N	-1.85771900	10.71820400	-2.65885000
C	-2.72969600	9.71856100	-2.46887100
H	-3.07165800	9.36029400	-1.49984900
N	-3.07456500	9.25547700	-3.66977000
H	-3.65451200	8.39825300	-3.82376800
C	-2.39692900	9.96332300	-4.64347200
H	-2.51712200	9.74422100	-5.69234400
C	2.48994800	12.85172400	-1.41585400
H	2.89216800	12.75247400	-2.43061900
H	3.35028000	12.94825700	-0.74005900
C	1.62518400	11.67021500	-1.03751500
C	0.48482200	11.82655400	-0.23673900
H	0.20971500	12.82036400	0.11167900
C	-0.29502600	10.73192500	0.16074700
H	-1.14250000	10.85851700	0.83060400
C	0.05716900	9.43989900	-0.26598300
O	-0.68095700	8.33809600	0.05955600
H	-1.61591200	8.59865400	0.34233700
C	1.20395600	9.26041700	-1.05140300
H	1.48142600	8.25339800	-1.34800300
C	1.96739400	10.36429700	-1.42757200
H	2.85407400	10.20772300	-2.03865300
C	6.46987400	11.60929900	0.73995500
H	5.53565000	11.93942200	0.26095700
C	7.52776200	11.33910800	-0.33683100
H	7.20994800	10.55779100	-1.03672600
H	7.73325500	12.24182400	-0.92336600
H	8.47370100	11.01461500	0.11447300
C	6.16969800	10.40718800	1.65127600
H	7.10288300	10.08054400	2.13203900
H	5.50814200	10.73696900	2.46377100
C	5.52187100	9.21572000	0.93442000
H	5.30572700	8.40449600	1.64005400
H	4.57509100	9.50580500	0.46079000
H	6.17375600	8.80623100	0.15432600
C	-5.40896300	9.85531900	3.35576700
H	-6.48727200	10.01620900	3.41306000
C	-5.05309500	9.32620300	1.95251600
H	-5.39592400	8.29087000	1.84284900
H	-5.61032500	9.90428000	1.20510400
C	-3.56732900	9.38858700	1.57718200
O	-2.75075100	9.98397700	2.30361200
O	-3.25522000	8.78875400	0.46893700
C	-5.03414000	8.84536200	4.42694500
O	-5.88323700	8.11475500	4.96299600
N	-3.71380400	8.77153300	4.74003800
H	-3.12532600	9.35368600	4.14065500
C	-3.07213800	7.56588100	5.28488700
H	-3.49247900	6.69258100	4.76539600
C	-1.57508300	7.60838900	4.96503000
H	-1.14921600	8.58110800	5.24079100
H	-1.08364300	6.85864000	5.59429700
C	-1.30463000	7.26917500	3.50245300
O	-2.03725200	6.49579800	2.88461500
N	-0.17981200	7.80273500	2.95491000
H	-0.12275300	7.76845000	1.93709800
H	0.23381800	8.61674200	3.38907600
C	-3.27195800	7.25421700	6.78187400
O	-2.47714700	6.50431000	7.35946300
N	-4.37556100	7.75076900	7.38762800
H	-5.11338500	8.10362000	6.78078600
C	-4.71684600	7.33776700	8.74454300
H	-3.78558300	7.23864900	9.30862600
C	-5.51032600	6.02032900	8.78501300
O	-6.67561800	5.98350800	9.18103200
N	-4.85491000	4.92330800	8.31607600
H	-3.90920500	5.01457800	7.94638800
C	-5.53421000	3.64929400	8.16099900
H	-5.88247300	3.30406800	9.14271100
C	-6.79968600	3.72217700	7.23651400
H	-7.04402600	2.72047000	6.86872800
H	-7.63000300	4.09320600	7.83786400
C	-6.56329500	4.62603100	6.03317500
O	-5.83088200	4.27380300	5.09284200
N	-7.19868600	5.81264400	6.06371700
H	-6.90700800	6.54203700	5.41460600
H	-7.61926600	6.11542800	6.93375600
C	-4.55670700	2.62283000	7.58284400
O	-3.59438300	2.94467500	6.88654700
N	-4.86225700	1.32123500	7.86535800
H	-5.77332200	1.15651600	8.27648400
C	-4.41139900	0.26752800	6.96610600
H	-3.35887400	0.43667400	6.73188800
C	-5.29623100	0.28912700	5.70383100
O	-6.48336800	0.60073800	5.78687500
N	-4.69294900	-0.00003800	4.52654600
H	-3.71069800	-0.24159600	4.46346300
C	-5.35984000	0.27036800	3.26533200
H	-6.39052900	-0.09294300	3.33471000
C	-5.41126800	1.81219300	2.99396800
H	-5.94887600	1.99683700	2.05751500

H	-5.99032200	2.27014200	3.79922700
C	-4.05920000	2.44346500	2.89238500
N	-3.32504800	2.95828500	3.94921100
H	-3.64652200	3.08938900	4.91005600
C	-2.12933300	3.36716300	3.47441000
H	-1.37569900	3.84038600	4.08377400
N	-2.04274400	3.13422800	2.16949900
C	-3.24610500	2.56565700	1.79854200
H	-3.44882000	2.27559700	0.78239300
C	-4.60756200	-0.47696200	2.15434300
O	-3.46597300	-0.90201300	2.32531800
N	-5.29770600	-0.61190500	0.98606300
H	-6.15428100	-0.08172500	0.89704000
C	-4.64645100	-0.94997000	-0.26690400
H	-3.75445100	-1.53470400	-0.03472700
C	-4.29649000	0.32289800	-1.06299600
O	-4.89250600	1.38024600	-0.82927900
N	-3.31329200	0.18059200	-1.97856600
H	-3.05532100	-0.75538400	-2.27377600
C	-2.89957600	1.24506800	-2.88377500
H	-3.43196500	2.14776400	-2.58604600
C	-1.37788300	1.49652300	-2.81022200
H	-0.83092200	0.55682600	-2.96204100
H	-1.07806200	2.16528500	-3.62535500
C	-0.91976700	2.12914500	-1.50813300
O	-1.75262400	2.23913400	-0.55177000
O	0.25493300	2.56709900	-1.36530400
C	-3.27029900	0.83373300	-4.32052200
O	-3.18490800	-0.33978300	-4.67539500
N	-3.63944600	1.85758300	-5.13331100
H	-3.72695400	2.78938400	-4.72711400
C	-4.09608600	1.65313200	-6.51489800
H	-4.33674500	2.66216400	-6.87570000
C	-3.04736900	1.03910900	-7.48250700
H	-3.08108700	-0.04672000	-7.35679700
C	-3.40806800	1.37727400	-8.93894700
H	-3.36687600	2.46177500	-9.11279800
H	-2.69823400	0.90382400	-9.62742300
H	-4.40878800	1.01909600	-9.18527500
C	-1.62192200	1.51944500	-7.17116300
H	-1.54860400	2.61463200	-7.20484000
H	-1.29276200	1.17975200	-6.18292500
H	-0.93403800	1.11650400	-7.92642600
C	-5.43356200	0.85389600	-6.54170400
O	-5.72563200	0.07802500	-7.43833000
N	-6.29349000	1.17199200	-5.51995700
H	-5.86595000	1.54194400	-4.67861200
C	-7.46678100	0.34640600	-5.25048400
H	-7.24135000	-0.69962800	-5.48629900
C	-7.82979600	0.48428300	-3.74348900
H	-8.33040900	1.45339000	-3.60634500
C	-8.73110900	-0.64356000	-3.25308100
H	-9.62474200	-0.72306700	-3.87628700
H	-8.18886800	-1.59453000	-3.29794500
H	-9.02813400	-0.46445300	-2.21270400
O	-6.59338700	0.49853900	-3.00906800
H	-6.67674300	1.09166600	-2.24695500
C	-8.69804000	0.73056400	-6.10027300
O	-9.68652700	0.00330200	-6.13434600
N	-8.61657000	1.93566300	-6.72047900
H	-7.73325200	2.42586100	-6.67230000
C	-9.68742000	2.49078400	-7.52846600
H	-10.56333700	1.85878800	-7.36440700

C	-9.99398900	3.95560000	-7.15088200
H	-10.85176400	4.28352300	-7.75132500
H	-10.30264600	3.98781100	-6.09921400
C	-8.81988400	4.88625400	-7.38006200
C	-8.59622300	5.45468900	-8.64304700
H	-9.29948000	5.25704100	-9.44935400
C	-7.48987700	6.27172200	-8.87740000
H	-7.33929900	6.70592200	-9.86243200
C	-6.58097800	6.53237700	-7.84908500
H	-5.71846500	7.16822200	-8.03169500
C	-6.78628400	5.97543200	-6.58481500
H	-6.08095700	6.17037500	-5.78063200
C	-7.90130700	5.16270800	-6.35533900
H	-8.06255200	4.73969400	-5.36585700
C	-9.65368000	7.84120900	1.69830400
H	-10.08723200	7.68068400	2.69289400
C	-8.26885900	7.18594000	1.64159400
H	-7.77953800	7.42687800	0.68792500
H	-7.64979000	7.58098100	2.44733700
O	-8.32157400	5.77008700	1.83707500
H	-8.66232700	5.35757500	1.02387700
C	-9.51431300	9.33935800	1.44035800
O	-8.58596700	9.98882200	1.92187000
N	-10.46286400	9.89260800	0.63136200
H	-11.15727000	9.28199000	0.22353700
C	-10.36644700	11.25407100	0.12238300
H	-9.50791800	11.70013300	0.62729400
C	-10.21951200	11.31392500	-1.41168000
H	-11.17893700	11.04981200	-1.87624400
H	-10.04391400	12.36604300	-1.68000500
C	-9.13388900	10.43045500	-2.00364400
C	-9.37357500	9.73861700	-3.19953000
H	-10.33942500	9.84212000	-3.69008900
C	-8.39558300	8.92167700	-3.77274500
H	-8.60155300	8.39811600	-4.70301800
C	-7.15627700	8.76932500	-3.14660200
H	-6.39969500	8.11912900	-3.57589500
C	-6.90567800	9.45284700	-1.95366700
H	-5.95051700	9.33283700	-1.44609700
C	-7.87975700	10.28122700	-1.39250500
H	-7.67011800	10.78655500	-0.45444300
C	-10.23271400	2.63097500	0.41576400
H	-9.68465700	2.17267600	1.24607100
H	-10.71371200	1.81968800	-0.14616800
C	-9.31738300	3.43801600	-0.47654800
C	-9.76305800	4.61811100	-1.09430700
H	-10.78055700	4.95911000	-0.91368200
C	-8.92884600	5.34696600	-1.95018600
H	-9.28973100	6.26168700	-2.41244600
C	-7.63128200	4.90076700	-2.21165700
H	-6.97987700	5.46028300	-2.87711400
C	-7.16833900	3.73203700	-1.59779600
H	-6.15763500	3.38083900	-1.78230400
C	-8.00170200	3.01843800	-0.73301300
H	-7.61928000	2.12467700	-0.24419600
C	0.11910800	-2.11970500	2.60789500
H	0.42862000	-2.31735500	3.64207000
H	0.63449600	-2.84356100	1.96363000
C	0.39537800	-0.70925400	2.20355400
N	1.65115400	-0.11766600	2.26721500
C	1.54620800	1.16476500	1.83921600
N	0.29763100	1.43195700	1.49970800
C	-0.42672000	0.27491100	1.72120200

H	-1.48850200	0.22082100	1.54020900
H	2.49977700	-0.56138800	2.58960900
H	2.36454300	1.86594800	1.79422600
C	3.02201700	3.80865300	-2.72908500
H	2.59509100	3.17988400	-1.94152400
H	3.19850700	4.81135000	-2.33108800
N	2.13781300	3.87409800	-3.88758700
H	2.25234400	3.18500700	-4.62378400
C	0.97550800	4.54629700	-3.90526600
N	0.61969100	5.36688400	-2.91212600
H	-0.31745700	5.75651500	-2.98113500
H	1.00853800	5.27674700	-1.96991700
N	0.18727300	4.44507800	-4.98891000
H	-0.73466300	4.87806900	-4.96459700
H	0.47275900	3.84022600	-5.75285200
Fe	-0.47583500	3.33938700	0.68753700
O	-1.08850300	4.98612400	0.09575500
C	-4.03867000	5.46535200	-0.69011600
C	-3.15303200	6.03114400	-1.74461200
N	-3.61786400	5.96996800	0.64339100
S	-3.42346800	5.74947200	-3.45280900
O	-4.36252700	6.84549100	-3.89505800
O	-2.07184800	5.91270400	-4.07689900
O	-4.02588400	4.39766900	-3.59397800
H	-3.98856100	4.37089200	-0.67481300
H	-5.08914000	5.74080700	-0.84909900
H	-2.36544000	6.73387800	-1.49974900
H	-0.35305800	5.62112000	-0.01807700
H	-3.57801100	7.01283300	0.64228700
H	-2.64942100	5.63948700	0.83977800
H	-4.27991400	5.66761900	1.39917500
C	1.91403000	5.09743900	0.84533500
O	1.29122900	4.04568000	1.28222500
O	1.63819700	5.70030000	-0.20924200
C	3.02328900	5.60614700	1.74908600
H	3.65025900	6.32667300	1.22054200
H	3.62914200	4.77810200	2.12869000
O	1.55801800	2.33190800	-6.44896600
H	0.94380500	1.58339900	-6.52653100
H	1.98989700	2.39804300	-7.31515200
O	-5.62775900	5.40792500	2.48075700
H	-6.57482500	5.38157200	2.22124000
H	-5.60525100	5.10321500	3.41367900
H	-0.95563200	-2.30700000	2.53722700
H	-4.50633900	-0.69673000	7.47512800
H	2.56411300	6.10154900	2.61319500
H	3.97801300	3.39532300	-3.05745100
H	6.80377500	12.44876500	1.36432000
H	-1.03128000	12.94107200	-4.33561900
H	1.93223100	13.79279500	-1.36402500
H	-4.89148000	10.80522100	3.52623000
H	-5.34327000	8.10173300	9.20747000
H	-5.31997100	-1.56420300	-0.87610400
H	-9.42609900	2.43186000	-8.59408900
H	-11.25238700	11.82911500	0.41926600
H	-11.03044300	3.24895800	0.84002700
H	-10.32568400	7.37169300	0.96845800
H	-1.35709700	11.17332900	-1.89387000

^{5}IM1$_{HA,C2,C}$

C	0.84969300	10.74702900	-4.83118500
H	0.98570300	10.64042300	-5.91012500
H	1.82002400	10.59814900	-4.34451200
C	-0.15577000	9.75952000	-4.33976700
N	-0.44622900	9.59447500	-2.99191600
C	-1.37793100	8.64474100	-2.82759600
H	-1.81833000	8.32167200	-1.87991600
N	-1.70183300	8.19647900	-4.03993000
H	-2.40243700	7.44484500	-4.21059500
C	-0.95911600	8.86659000	-4.99412000
H	-1.06049600	8.65725000	-6.04716300
C	3.42273100	13.25117400	-1.88475300
H	4.05431700	12.91452800	-2.71540100
H	4.08949000	13.70619300	-1.14071100
C	2.62748300	12.11198700	-1.28863900
C	1.28174500	12.26170400	-0.93877600
H	0.78707600	13.21509100	-1.11226300
C	0.54951000	11.22052400	-0.35185200
H	-0.48606300	11.36744600	-0.05694400
C	1.17095200	9.98457000	-0.09764400
O	0.50684000	8.93414900	0.44525200
H	-0.47187800	9.14831500	0.56908600
C	2.52014100	9.81757500	-0.45073800
H	2.99710800	8.86056300	-0.26027700
C	3.22652900	10.86604800	-1.03144000
H	4.27237300	10.71537400	-1.29107700
C	0.04330600	13.52054500	3.23321000
H	1.01943500	13.10810900	2.94028300
C	0.24336300	14.60372700	4.29993500
H	0.73356300	14.20856300	5.19664200
H	0.86655800	15.42120000	3.91959700
H	-0.71735100	15.03458900	4.60934200
C	-0.88029200	12.36831300	3.66424900
H	-1.86246200	12.78072600	3.93942500
H	-1.05431300	11.71457400	2.79809500
C	-0.34000100	11.52578700	4.82716400
H	-1.01543600	10.69445400	5.06051400
H	0.64207900	11.10081000	4.58194200
H	-0.22285500	12.11884800	5.74097500
C	-4.29414600	9.94198600	2.56831000
H	-5.27368600	10.33634900	2.84111500
C	-4.38524700	9.30299100	1.16917300
H	-5.01422700	8.40604800	1.20219800
H	-4.91305300	9.99833100	0.50641000
C	-3.04580300	8.94699000	0.51455900
O	-2.00582100	9.55780800	0.91604800
O	-3.06335100	8.07894000	-0.40863800
C	-3.88565500	8.90669300	3.60013600
O	-4.70557000	8.37261800	4.36679800
N	-2.56924300	8.57233600	3.59639000
H	-2.02505300	9.03478400	2.86927600
C	-2.03100600	7.30107400	4.09369500
H	-2.76211900	6.50981200	3.87123600
C	-0.74621200	6.95437100	3.33482200
H	-0.16376000	7.85049000	3.09075800
H	-0.12634500	6.34669600	4.00419300
C	-1.01065300	6.11063300	2.09265500
O	-2.00080800	5.35523700	2.05929100
N	-0.09099600	6.15452700	1.12099000
H	-0.16393700	5.49248100	0.33719700
H	0.54750300	6.93937300	1.04742700
C	-1.81378300	7.17806700	5.61536600

O	-0.97502600	6.39104300	6.06485200
N	-2.65908700	7.87465900	6.41383100
H	-3.48299300	8.28018400	5.97393800
C	-2.68162400	7.62161500	7.85083500
H	-1.65349000	7.43887900	8.17390700
C	-3.58410100	6.43264800	8.22522500
O	-4.61248900	6.58747300	8.88439300
N	-3.19974400	5.22058900	7.73700400
H	-2.37212100	5.14585500	7.14675500
C	-4.05448400	4.05506400	7.87608200
H	-4.21171400	3.85210200	8.94288600
C	-5.47848800	4.23867700	7.24100600
H	-5.91099100	3.25876600	7.01647400
H	-6.10662600	4.74934100	7.97161700
C	-5.42147100	5.02994900	5.94065300
O	-4.98615400	4.52290300	4.89375400
N	-5.87682500	6.29568600	6.01168500
H	-5.67944400	6.93702800	5.24482900
H	-6.03764500	6.70458000	6.92393900
C	-3.37448500	2.84448300	7.23275400
O	-2.53557500	2.95369700	6.33818700
N	-3.79530300	1.63552500	7.70622200
H	-4.61025600	1.64812900	8.30684900
C	-3.66343400	0.44805300	6.87371900
H	-2.63084200	0.37279100	6.52625600
C	-4.65932000	0.54899300	5.69981200
O	-5.74889700	1.09537800	5.85430400
N	-4.24138900	0.05998000	4.50768500
H	-3.34913600	-0.40640400	4.39165300
C	-4.95622300	0.36420300	3.28133400
H	-6.02657400	0.20026300	3.44610500
C	-4.74929000	1.86568300	2.89120700
H	-5.37762400	2.11162500	2.02858600
H	-5.10483800	2.47850400	3.72412400
C	-3.32644100	2.18549900	2.55515300
N	-2.33887900	2.49194700	3.47822400
H	-2.47735800	2.69606800	4.47228700
C	-1.16758100	2.60956700	2.82102100
H	-0.23435800	2.87057700	3.29667200
N	-1.33564900	2.37415700	1.52465100
C	-2.68127000	2.11880700	1.34815400
H	-3.08726000	1.89281500	0.37685100
C	-4.43297200	-0.58207900	2.18941200
O	-3.42784500	-1.27003400	2.36804500
N	-5.14575100	-0.57887600	1.02870500
H	-5.84741800	0.14052300	0.90180400
C	-4.62532900	-1.14858900	-0.19912000
H	-3.69230000	-1.66278900	0.04331600
C	-4.39561300	-0.04025800	-1.24286700
O	-4.91203400	1.07295300	-1.08351000
N	-3.61280500	-0.37444200	-2.28874800
H	-3.36444100	-1.34737300	-2.41760000
C	-3.15720200	0.56706600	-3.30120800
H	-3.46569200	1.55980900	-2.96788200
C	-1.61884600	0.53665400	-3.41337100
H	-1.25815600	-0.49553200	-3.51560400
H	-1.28509500	1.05828600	-4.31457100
C	-0.91999800	1.18438100	-2.22877300
O	-1.62332200	1.58788600	-1.24747600
O	0.32976500	1.33238700	-2.22071500
C	-3.82535000	0.26404300	-4.65775600
O	-4.37537800	-0.80400000	-4.87690300
N	-3.71403000	1.28370000	-5.58574500

H	-3.52173200	2.19788900	-5.17114700
C	-4.62297400	1.33228200	-6.75931500
H	-4.36118000	2.27236600	-7.26438500
C	-4.45094400	0.17632900	-7.77683200
H	-5.00783900	-0.68121300	-7.39095200
C	-5.05286700	0.57927300	-9.13500500
H	-4.53432800	1.45244900	-9.55531000
H	-4.95015500	-0.24366600	-9.85127400
H	-6.11407800	0.81569300	-9.04264700
C	-2.98719100	-0.24429800	-7.96141400
H	-2.37552500	0.57982500	-8.35634900
H	-2.53306900	-0.60445800	-7.03349800
H	-2.92762300	-1.05703000	-8.69324900
C	-6.10610200	1.48150200	-6.30265000
O	-7.02193700	0.85963000	-6.82222100
N	-6.30803900	2.41883200	-5.32277300
H	-5.52328700	2.84915900	-4.84114800
C	-7.61938300	2.68680700	-4.74993700
H	-8.34040200	2.11911400	-5.34906300
C	-7.70108400	2.21942800	-3.28431000
H	-8.63679900	2.61191400	-2.87624900
C	-7.69230400	0.69944800	-3.14977600
H	-8.56103700	0.26200700	-3.65242900
H	-6.79368900	0.25432100	-3.59152400
H	-7.73824300	0.42051800	-2.09086700
O	-6.62320200	2.83340700	-2.56492400
H	-6.05405200	2.14926500	-2.16584400
C	-8.00755400	4.18195800	-4.87960300
O	-8.81595700	4.70623800	-4.11329000
N	-7.41945300	4.82299000	-5.91898600
H	-6.78639400	4.28563000	-6.49640100
C	-7.70689300	6.18901300	-6.32408900
H	-8.19993800	6.68337800	-5.48394900
C	-6.42055400	6.94072100	-6.72194000
H	-6.70966100	7.95833500	-7.01554400
H	-5.77195700	7.02120400	-5.84311700
C	-5.65883200	6.27201200	-7.85284400
C	-6.13243000	6.32133500	-9.17323100
H	-7.04402100	6.87192900	-9.39637100
C	-5.44739000	5.68091400	-10.20635500
H	-5.82958900	5.73465100	-11.22210800
C	-4.27010200	4.97664300	-9.93864600
H	-3.73456900	4.48357700	-10.74556100
C	-3.78622100	4.92328000	-8.63029000
H	-2.86357500	4.39037000	-8.40650700
C	-4.47474900	5.56395300	-7.59498700
H	-4.08519800	5.53500600	-6.58104200
C	-8.38288100	8.94251800	2.64813300
H	-7.49636300	8.93715300	3.29113400
C	-8.32916000	7.71568000	1.73113700
H	-9.24314800	7.64152900	1.13144100
H	-7.48504400	7.81705700	1.03793300
O	-8.16713900	6.54275900	2.54077500
H	-8.70501500	5.82473300	2.16101200
C	-8.35908700	10.21354300	1.80605500
O	-7.32536600	10.60564800	1.26644100
N	-9.55872800	10.84702400	1.65639800
H	-10.37270200	10.43503900	2.09147800
C	-9.76435100	11.92145300	0.69513000
H	-8.77525600	12.15477300	0.29586200
C	-10.73973100	11.53462400	-0.43838300
H	-11.74562200	11.40229200	-0.01797400
H	-10.80442500	12.39285400	-1.12055100

C	-10.34056400	10.28315400	-1.19327100
C	-11.05526600	9.08915500	-1.02863900
H	-11.92766000	9.07566300	-0.37768400
C	-10.67472700	7.92212000	-1.69647900
H	-11.24199700	7.00694600	-1.55162000
C	-9.56692900	7.92777000	-2.54670600
H	-9.27310300	7.01764400	-3.06420200
C	-8.85147600	9.11577400	-2.72341500
H	-7.99005800	9.13807400	-3.38677400
C	-9.23366500	10.27925900	-2.05409400
H	-8.66449100	11.19481700	-2.20028600
C	-12.56410700	3.84973400	2.12009100
H	-12.39076000	4.08092800	3.17629700
H	-13.01807100	2.85088100	2.07534200
C	-11.28146200	3.90180400	1.32192200
C	-11.30492900	4.09578300	-0.06701300
H	-12.26158900	4.23327600	-0.56720000
C	-10.12612500	4.11281100	-0.81682800
H	-10.15702500	4.27172500	-1.89162300
C	-8.89214300	3.93301200	-0.18547600
H	-7.97842200	3.92670000	-0.77262100
C	-8.85009000	3.73880000	1.19900800
H	-7.89233700	3.61314700	1.69982500
C	-10.03590700	3.72894100	1.94461300
H	-9.99518200	3.57305400	3.02101000
C	-0.11628600	-3.25149900	1.76149400
H	0.55626200	-3.53941100	2.57960300
H	-0.11432700	-4.06836600	1.02856400
C	0.27345300	-1.94709500	1.14784000
N	1.47507200	-1.73270300	0.48472700
C	1.50973400	-0.44542800	0.05812800
N	0.40061300	0.18135300	0.40990200
C	-0.37552600	-0.74071000	1.08579400
H	-1.34549800	-0.49160800	1.48728200
H	2.20766700	-2.41545800	0.34899400
H	2.32452400	-0.00780300	-0.50065400
C	2.89395000	1.97011100	-4.59101000
H	2.72433500	1.31044100	-3.73306000
H	3.70623700	2.66367500	-4.35330300
N	1.68579000	2.70575700	-4.94877200
H	1.10881400	2.34622900	-5.70096500
C	1.06277300	3.54195500	-4.09628400
N	1.64294400	3.92039800	-2.94995800
H	0.99486000	4.25791200	-2.22373700
H	2.33358500	3.29291900	-2.52857600
N	-0.11621800	4.06706600	-4.46434900
H	-0.70904000	4.53822900	-3.77795700
H	-0.57533000	3.69176900	-5.29108800
Fe	0.00386400	2.32447600	-0.11945000
O	-0.25527700	4.05619100	-0.83964900
C	-4.51405400	4.76010900	-0.84017300
C	-4.77690400	5.39246100	-2.13905200
N	-3.99336000	5.53941400	0.25840700
S	-3.44749700	5.08626500	-3.40047000
O	-2.13975200	5.15356000	-2.66705500
O	-3.70556300	3.74534100	-4.01056000
O	-3.57901900	6.20778800	-4.39044500
H	-4.67071000	3.70691000	-0.65337900
H	-1.09652600	4.20092100	-1.31846800
H	-5.68346400	4.98668600	-2.59693400
H	-4.82839500	6.48245500	-2.06510200
H	-4.68293300	5.53721000	1.07989200
H	-3.76813000	6.52869500	-0.01926600
H	-3.10971900	5.17622100	0.69128600
C	2.97633700	2.72581700	0.11271100
O	1.75314200	2.70617100	0.57403200
O	3.28540200	2.44689000	-1.05086700
C	4.01970700	3.11855200	1.14371900
H	5.01766900	3.10251500	0.70260100
H	3.97795700	2.43308600	1.99727300
O	-0.96350200	2.30044700	-6.64519100
H	-1.75070300	1.76003700	-6.43102600
H	-0.94391000	2.34447000	-7.61342400
O	-5.72317900	5.25438000	2.30581000
H	-6.54887400	5.78046000	2.42848700
H	-5.37145600	5.08139400	3.21054900
H	-1.12721100	-3.16790900	2.16943700
H	-3.88817600	-0.43170700	7.48424600
H	3.79954200	4.12113400	1.52641500
H	3.19608900	1.37454800	-5.45428600
H	-0.37356900	13.98456800	2.32867600
H	0.53163500	11.77631200	-4.62851500
H	2.76736100	14.04364900	-2.26066700
H	-3.57242500	10.76407300	2.54220200
H	-3.07565800	8.49790200	8.36759300
H	-5.32566200	-1.88819300	-0.61062500
H	-8.41490900	6.18811000	-7.16498500
H	-10.13907100	12.81565500	1.20942800
H	-13.30667900	4.55718600	1.73489500
H	-9.26928800	8.88133500	3.29041400
H	-0.01792000	10.11726400	-2.22349300

Model C2 structures:

$^{5}Re_{C2}$

C	-0.82106400	11.80574000	-4.85426800
H	-0.73266000	11.60148200	-5.92396000
H	0.17990900	11.75722800	-4.41085200
C	-1.73223500	10.81150000	-4.21622200
N	-1.95690100	10.77036500	-2.84660700
C	-2.79537700	9.76876800	-2.54572300
H	-3.11646700	9.48982200	-1.54484200
N	-3.13339700	9.17243800	-3.68651200
H	-3.69252900	8.28652400	-3.73517800
C	-2.48488100	9.79388800	-4.73561300
H	-2.60410300	9.46141100	-5.75426500
C	2.35144000	12.96375700	-1.68591400
H	2.67672900	12.91172200	-2.73139100
H	3.26037000	13.01732100	-1.07183800
C	1.49998700	11.77424300	-1.30307100
C	0.41750800	11.90396500	-0.42136800
H	0.17763600	12.88384000	-0.01268700
C	-0.34735000	10.80038800	-0.02107900
H	-1.14739800	10.90820700	0.70723000
C	-0.04398300	9.52495600	-0.52739300
O	-0.77709300	8.41933200	-0.21629300
H	-1.67638400	8.67210500	0.16319900
C	1.04344800	9.37376200	-1.40007900
H	1.27748100	8.38318900	-1.77944600
C	1.79708300	10.48438400	-1.77530800
H	2.63777600	10.34635100	-2.45227900
C	6.20963500	11.68551000	0.72916300
H	5.30036000	12.06503000	0.23885000
C	7.28058600	11.40177800	-0.33114600
H	6.94882300	10.65090500	-1.05728000
H	7.53333400	12.31032100	-0.88963100
H	8.20329300	11.02996400	0.13169600
C	5.84196700	10.47334600	1.60156800
H	6.74914600	10.10362100	2.10077200
H	5.16893900	10.80792200	2.40278700
C	5.17506600	9.32218000	0.83815900
H	4.90047200	8.50653700	1.51793700
H	4.25884800	9.65674100	0.33516800
H	5.83871000	8.90091800	0.07456000
C	-5.30383500	9.98019300	3.35518500
H	-6.37468400	10.15653400	3.48121100
C	-5.02745600	9.73500100	1.86012800
H	-5.61325400	8.88373200	1.50247400
H	-5.38605300	10.60481600	1.29436600
C	-3.54736300	9.53166900	1.51112600
O	-2.66340300	9.99924900	2.25083100
O	-3.31823700	8.87318100	0.41565500
C	-4.92574900	8.75373800	4.16698200
O	-5.69157900	7.79390300	4.29457100
N	-3.66982300	8.76785600	4.71713700
H	-3.07123100	9.48696000	4.31522900
C	-2.99002400	7.53263800	5.11952000
H	-3.45645600	6.70086400	4.57606600
C	-1.51111500	7.56845600	4.71707500
H	-1.06572000	8.54951100	4.92323100
H	-0.98387300	6.83734000	5.34004900
C	-1.32488800	7.15311800	3.26046900
O	-2.09494500	6.33773500	2.74897900
N	-0.23925900	7.64608600	2.61650100
H	-0.20993300	7.52326100	1.60456800
H	0.18193700	8.49928400	2.95877400
C	-3.12504400	7.12839800	6.59474300
O	-2.42771800	6.21190000	7.04141400
N	-4.07303300	7.74165400	7.33803300
H	-4.66107500	8.43615200	6.89940200
C	-4.36484600	7.29634400	8.69741400
H	-3.41441500	7.07143200	9.19039600
C	-5.29180200	6.06511300	8.73831300
O	-6.45072900	6.15034100	9.14522300
N	-4.74438500	4.91094000	8.27481400
H	-3.81386300	4.92929500	7.85480500
C	-5.52253900	3.69068500	8.14607200
H	-5.87154700	3.37947400	9.13875300
C	-6.79626600	3.84475800	7.24644800
H	-7.12262300	2.85879100	6.90012200
H	-7.58424000	4.28893900	7.85555600
C	-6.51683500	4.70404500	6.01968000
O	-5.86053900	4.26933100	5.06019500
N	-7.02832000	5.94984200	6.05434000
H	-6.70960800	6.61991900	5.35475000
H	-7.39220800	6.30559800	6.92946900
C	-4.62527800	2.59594600	7.56007400
O	-3.64476700	2.85246300	6.86181200
N	-5.01505700	1.31858900	7.84143100
H	-5.93499300	1.20840100	8.25050100
C	-4.61249100	0.23746300	6.95129100
H	-3.54289800	0.32992200	6.75395500
C	-5.45071500	0.32353900	5.66032500
O	-6.62302900	0.69121200	5.70876300
N	-4.81831600	0.01914400	4.50201800
H	-3.84739400	-0.26972700	4.47308900
C	-5.42844100	0.31541000	3.21808500
H	-6.47790200	0.00516400	3.25684100
C	-5.39650900	1.85410300	2.93006000
H	-5.90328500	2.06089600	1.98232000
H	-5.97148900	2.34618500	3.71783500
C	-4.01586600	2.42528000	2.85541300
N	-3.27632300	2.87042600	3.93920100
H	-3.60228600	2.97497000	4.90179700
C	-2.05786600	3.24990900	3.49530200
H	-1.29134900	3.66242000	4.13246800
N	-1.96636800	3.06594700	2.18513300
C	-3.18482500	2.56394700	1.77380300
H	-3.38048000	2.33034300	0.74073900
C	-4.68046300	-0.47376100	2.13636300
O	-3.55952800	-0.93970200	2.33877300
N	-5.34624100	-0.59176200	0.95272400
H	-6.17752200	-0.02630600	0.84126200
C	-4.67160600	-0.94243600	-0.28425800
H	-3.81380300	-1.56977500	-0.03574100
C	-4.25039700	0.32450300	-1.05213400
O	-4.84361900	1.39146000	-0.85478600
N	-3.21839500	0.16542100	-1.90966200
H	-2.96290300	-0.77847300	-2.18166900
C	-2.77850400	1.20443900	-2.83343800
H	-3.28015100	2.12755900	-2.54463100
C	-1.25025000	1.40061200	-2.78271600
H	-0.73966800	0.44055700	-2.92982300
H	-0.94300500	2.04244900	-3.61725500
C	-0.71585200	2.04884400	-1.51171600
O	-1.55373100	2.31024200	-0.57668300
O	0.49341700	2.34122700	-1.39996100
C	-3.17111500	0.77837200	-4.26120600

O	-3.09436900	-0.39990100	-4.60287800
N	-3.54513300	1.79336300	-5.08179500
H	-3.62712700	2.73066800	-4.68670200
C	-3.99189000	1.57757700	-6.46402700
H	-4.23755400	2.58294200	-6.83174700
C	-2.93084400	0.96838000	-7.42168600
H	-2.95515200	-0.11726600	-7.29195900
C	-3.28245800	1.29772400	-8.88229700
H	-3.24876400	2.38189800	-9.05987500
H	-2.56307400	0.82760600	-9.56309600
H	-4.27827700	0.93047800	-9.13501400
C	-1.51193900	1.46354000	-7.10066100
H	-1.45050100	2.55950300	-7.12953800
H	-1.18650700	1.12202100	-6.11151200
H	-0.81518400	1.06850700	-7.85186600
C	-5.32381600	0.77046200	-6.49754100
O	-5.61037800	-0.00132300	-7.39954700
N	-6.18925100	1.07905500	-5.47689700
H	-5.76442000	1.44174800	-4.63113300
C	-7.36419300	0.25117600	-5.22307500
H	-7.13585400	-0.79388400	-5.46032000
C	-7.74561200	0.38180800	-3.71959700
H	-8.24498700	1.35115000	-3.58216800
C	-8.65530200	-0.74683400	-3.24757400
H	-9.53938100	-0.82293100	-3.88469300
H	-8.11332700	-1.69812900	-3.28921600
H	-8.96725300	-0.57212500	-2.21111000
O	-6.52020800	0.38618600	-2.96544400
H	-6.58348300	1.02423600	-2.23854900
C	-8.58568000	0.63838200	-6.08548600
O	-9.56959800	-0.09345000	-6.14285400
N	-8.50298500	1.85312000	-6.68712700
H	-7.62191100	2.34545400	-6.62208000
C	-9.56444700	2.41275400	-7.50406500
H	-10.43603200	1.76792000	-7.36956300
C	-9.89565400	3.86621900	-7.10333200
H	-10.74278700	4.19776000	-7.71681500
H	-10.22889000	3.87305500	-6.05876500
C	-8.72668400	4.81345900	-7.28517600
C	-8.48153800	5.41505000	-8.52861800
H	-9.16511000	5.23078400	-9.35479500
C	-7.37866200	6.24825500	-8.71839100
H	-7.21110500	6.70805100	-9.68898800
C	-6.49469800	6.49184600	-7.66439700
H	-5.63448400	7.13959800	-7.81316000
C	-6.72164300	5.90155400	-6.41909500
H	-6.03605600	6.08112800	-5.59446400
C	-7.83314300	5.07318400	-6.23436900
H	-8.01158600	4.62506700	-5.25922600
C	-9.63245900	8.06783000	1.87094200
H	-9.98535100	7.93092400	2.89990000
C	-8.30974600	7.30964300	1.69292300
H	-7.88877000	7.51759300	0.69950000
H	-7.59306500	7.64229200	2.44525000
O	-8.46854200	5.90049300	1.88507200
H	-8.82800100	5.51348500	1.06651900
C	-9.41009900	9.55379000	1.60785700
O	-8.45067500	10.15477600	2.09222200
N	-10.31983000	10.15331500	0.78731500
H	-11.04465900	9.57941400	0.37927600
C	-10.14096100	11.50074100	0.26370600
H	-9.25160300	11.89599700	0.75750100
C	-10.00288500	11.53439600	-1.27191600
H	-10.98030000	11.32461900	-1.72655600
H	-9.76521200	12.57069400	-1.55366700
C	-8.97815100	10.57895100	-1.86042500
C	-9.26897700	9.88659300	-3.04453500
H	-10.23055800	10.04167100	-3.52984100
C	-8.34685300	9.00366600	-3.61231500
H	-8.59175400	8.48109900	-4.53361800
C	-7.11409400	8.78494500	-2.99304500
H	-6.40097500	8.08506700	-3.41817400
C	-6.81240800	9.46766700	-1.81130600
H	-5.86250300	9.29722000	-1.30841900
C	-7.73106000	10.36092300	-1.25569500
H	-7.48507600	10.86345800	-0.32494000
C	-10.19446800	2.60944000	0.52070700
H	-9.62850300	1.80671700	1.00344700
H	-11.02217600	2.14606700	-0.03082900
C	-9.31803700	3.42875900	-0.39910700
C	-9.78101400	4.64239100	-0.93815000
H	-10.78391300	4.98582500	-0.68988200
C	-8.98468300	5.39796900	-1.80535800
H	-9.35748600	6.33575400	-2.20860000
C	-7.70954900	4.94743300	-2.15908000
H	-7.08585000	5.53065800	-2.83022200
C	-7.23425200	3.74443900	-1.63047200
H	-6.24110000	3.38956300	-1.89080100
C	-8.03008100	3.00227400	-0.75297800
H	-7.63746200	2.08065800	-0.32889200
C	-0.13935300	-2.26885200	2.32825000
H	0.21250600	-2.54719100	3.32972300
H	0.28630500	-2.98673000	1.61544200
C	0.21589300	-0.85983100	1.98603100
N	1.51407400	-0.36332600	1.99738000
C	1.48135600	0.94628900	1.65516200
N	0.23524800	1.32021700	1.42164700
C	-0.56055500	0.20749200	1.62088600
H	-1.63198400	0.24076400	1.51119900
H	2.34686600	-0.88609300	2.23000400
H	2.34450700	1.58887100	1.59176800
C	3.19661800	3.80359600	-2.56767200
H	2.81571000	3.11799900	-1.80525400
H	3.37407400	4.77881600	-2.10941200
N	2.25845800	3.92978900	-3.67765300
H	2.32822000	3.26470400	-4.44096100
C	1.10176700	4.60884000	-3.61940000
N	0.78820500	5.37494200	-2.56732300
H	-0.11542400	5.83247400	-2.59476900
H	1.18549800	5.21245200	-1.63324800
N	0.28271400	4.57106300	-4.68309200
H	-0.64367500	4.99751500	-4.62014500
H	0.55126600	4.01126500	-5.48669000
Fe	-0.35280900	3.22624100	0.77796600
O	-0.79457600	4.70161300	0.25967100
C	-4.19448400	5.52775600	-0.64588100
C	-2.99934000	5.74976600	-1.56609900
N	-3.88964000	6.06166800	0.71334300
S	-3.39477900	5.66474200	-3.32673600
O	-4.39665500	6.76595400	-3.57357000
O	-2.08894700	5.95072300	-3.99921400
O	-3.95634800	4.31316200	-3.58926800
H	-4.43160100	4.46783900	-0.54880100
H	-5.08054200	6.05409100	-1.00780400
H	-2.55640300	6.73838400	-1.41044500
H	-2.22344700	5.00721000	-1.37016900

H	-3.78486500	7.09845800	0.67523300
H	-2.99714400	5.71455700	1.09967300
H	-4.65047900	5.78691600	1.40125400
C	2.00408800	4.85052400	1.15499800
O	1.29483500	3.80668300	1.52553100
O	2.01736600	5.34433300	0.02883700
C	2.85037600	5.42797900	2.27723400
H	3.61084400	6.09794400	1.87113800
H	3.31686100	4.63379400	2.86743400
O	1.57290600	2.50985200	-6.29232300
H	0.95540400	1.76491100	-6.37823400
H	1.99534600	2.59192600	-7.16163800
O	-5.85323200	5.07010300	2.32277600
H	-6.80218700	5.28875300	2.18066200
H	-5.75335000	4.95534800	3.29297100
H	-1.22711100	-2.37515200	2.31211800
H	-4.79487900	-0.71773000	7.45320700
H	2.19183300	5.99597300	2.94492600
H	4.14147800	3.42940800	-2.96836400
H	6.56139500	12.49509800	1.38264800
H	-1.19487800	12.82942700	-4.73496800
H	1.81184300	13.90656300	-1.54697500
H	-4.74536700	10.85890900	3.69089300
H	-4.87227800	8.09875700	9.23388700
H	-5.35395600	-1.51321500	-0.92467200
H	-9.28200700	2.37841700	-8.56536300
H	-10.98588100	12.13459200	0.56062300
H	-10.63810800	3.22637000	1.31064100
H	-10.39791600	7.65111300	1.20390100

^{5}TS1$_{HA,C1S,C2}$

C	-0.85917600	11.81607700	-4.94611600
H	-0.70468600	11.54799800	-5.99397200
H	0.12125800	11.89652600	-4.46354000
C	-1.69888000	10.78357700	-4.27188900
N	-1.96734800	10.80572600	-2.90985500
C	-2.72439800	9.75293900	-2.57098200
H	-3.05526200	9.51046900	-1.56506600
N	-2.96768500	9.05956000	-3.68065700
H	-3.45206000	8.12843500	-3.69773900
C	-2.34011400	9.67252500	-4.74722000
H	-2.39410800	9.26939200	-5.74569200
C	2.06203100	13.40311600	-1.83236100
H	2.50843300	13.26357600	-2.82319300
H	2.88154600	13.64566600	-1.14301500
C	1.31161500	12.17068300	-1.37922400
C	0.21276400	12.26492100	-0.51161600
H	-0.11218100	13.24507400	-0.16741900
C	-0.45986900	11.12875500	-0.04485700
H	-1.27225100	11.21444100	0.67205300
C	-0.04695500	9.85414700	-0.46928000
O	-0.69211200	8.71301600	-0.09239800
H	-1.61996900	8.91391700	0.25057300
C	1.05752300	9.73892400	-1.32441900
H	1.37255500	8.74949600	-1.64309000
C	1.72061200	10.88356200	-1.76589100
H	2.57654300	10.77404900	-2.42845700
C	5.21597100	11.83619500	0.90338700
H	4.26353000	11.93044200	0.36102900
C	6.35447600	11.60978200	-0.09890500
H	6.20255300	10.70029600	-0.69123800
H	6.43659500	12.44889500	-0.79944100
H	7.31866800	11.51279100	0.41560600
C	5.07705500	10.73234300	1.96550700
H	6.02567500	10.63862200	2.51331200
H	4.32897500	11.04894000	2.70524100
C	4.67406600	9.36322700	1.40317600
H	4.54203600	8.63060900	2.20834800
H	3.72852600	9.42639500	0.84950900
H	5.43188500	8.96393100	0.71958200
C	-5.28005100	9.96358200	3.44546100
H	-6.35539900	10.09907500	3.58311800
C	-5.00529100	9.77962100	1.94146400
H	-5.56185000	8.92049900	1.55750700
H	-5.40047800	10.65444600	1.40923700
C	-3.52083500	9.64576300	1.58070800
O	-2.65356500	10.14508100	2.31963100
O	-3.26771500	9.00492200	0.47961300
C	-4.85520000	8.72583900	4.21705600
O	-5.59133700	7.74214000	4.33598100
N	-3.58995000	8.75912700	4.74468700
H	-3.01427500	9.50454900	4.35727600
C	-2.88149000	7.53754700	5.13801200
H	-3.30159200	6.70032600	4.56633700
C	-1.39031800	7.63571400	4.79100000
H	-0.98121200	8.61072600	5.08286100
H	-0.87048400	6.87497800	5.38334600
C	-1.13764100	7.32474000	3.32025100
O	-1.76870900	6.42150300	2.76347500
N	-0.15125400	8.02115400	2.70950800
H	-0.11818200	7.98196000	1.68960900
H	0.13589800	8.90350700	3.11120200
C	-3.03938100	7.09944700	6.60284500
O	-2.33834400	6.18084700	7.03842200
N	-3.99652500	7.69342800	7.34899300
H	-4.58972600	8.38810700	6.91802700
C	-4.28862300	7.22807700	8.70187500
H	-3.33733400	7.01138300	9.19704300
C	-5.19749600	5.98304300	8.72705500
O	-6.35549700	6.04608700	9.14076000
N	-4.63505800	4.84461300	8.24412400
H	-3.70628700	4.88401300	7.82154200
C	-5.39083000	3.61121000	8.10849100
H	-5.72175300	3.28045300	9.10102700
C	-6.67841800	3.75051700	7.22620300
H	-6.99211400	2.76087600	6.87871300
H	-7.46530700	4.17757200	7.84881100
C	-6.42934900	4.62254400	6.00229600
O	-5.78899500	4.20039500	5.02631600
N	-6.94736500	5.86486000	6.05817200
H	-6.64476700	6.54450900	5.36138800
H	-7.29860400	6.20902300	6.94316300
C	-4.47961200	2.54249600	7.49591600
O	-3.52999100	2.82639600	6.76721600
N	-4.82549100	1.25469000	7.79046900
H	-5.73132900	1.12172100	8.22381200
C	-4.42726700	0.18628600	6.88342500
H	-3.36917100	0.30871400	6.64511200
C	-5.31624900	0.25121300	5.62614600
O	-6.49293800	0.59700100	5.71932600
N	-4.72470300	-0.04714800	4.44575700
H	-3.74971700	-0.31541700	4.37862300
C	-5.39316600	0.22506600	3.18585700
H	-6.43599300	-0.09663600	3.27577700
C	-5.39439100	1.76094100	2.88159300

H	-5.93558600	1.94657400	1.94806000
H	-5.95194200	2.25121100	3.68322200
C	-4.02925900	2.35888800	2.75298100
N	-3.26894700	2.86207400	3.79784300
H	-3.56701500	2.98111500	4.76721300
C	-2.07907600	3.26570300	3.30042700
H	-1.30655000	3.72767700	3.89449400
N	-2.02525300	3.04370400	1.99231400
C	-3.23960000	2.48695300	1.64169600
H	-3.46253000	2.20309800	0.62713900
C	-4.68472500	-0.57672200	2.08575000
O	-3.54705500	-1.01719400	2.24284900
N	-5.40892700	-0.74647200	0.94270600
H	-6.25746000	-0.20330600	0.85530900
C	-4.79121400	-1.13987600	-0.31105000
H	-3.93982300	-1.78312000	-0.08110400
C	-4.36333700	0.09891900	-1.12069300
O	-4.91260200	1.18769800	-0.91585900
N	-3.37605900	-0.10320700	-2.01925000
H	-3.14493200	-1.05466000	-2.28255600
C	-2.88537300	0.93788900	-2.91260600
H	-3.32366000	1.87586000	-2.57414300
C	-1.34689200	1.05641500	-2.85870000
H	-0.87922000	0.06873200	-2.96143600
H	-0.99393800	1.65918400	-3.70296700
C	-0.84770000	1.72822200	-1.58845800
O	-1.67107400	1.87990900	-0.62894400
O	0.32802000	2.16685500	-1.49314900
C	-3.32793200	0.61570600	-4.34979900
O	-3.34136300	-0.53934300	-4.76685200
N	-3.65944300	1.70500100	-5.09450000
H	-3.62746400	2.61992300	-4.64691700
C	-4.15238900	1.61305300	-6.47441200
H	-4.33339200	2.65495900	-6.77159100
C	-3.16109100	0.99295000	-7.49887600
H	-3.26439700	-0.09429300	-7.43955000
C	-3.51726700	1.44511000	-8.92543900
H	-3.40360900	2.53312200	-9.03274100
H	-2.85049600	0.96882600	-9.65391600
H	-4.54371000	1.17263700	-9.17476800
C	-1.70299100	1.35889800	-7.18430700
H	-1.55858100	2.44687100	-7.14916900
H	-1.38288500	0.93473500	-6.22620200
H	-1.05382100	0.96211100	-7.97594800
C	-5.54158800	0.90898600	-6.52589100
O	-5.91818600	0.25813600	-7.48864600
N	-6.34556700	1.17202000	-5.44506400
H	-5.86659800	1.40698500	-4.58353500
C	-7.57580100	0.41671100	-5.23109800
H	-7.43023600	-0.62414200	-5.54144200
C	-7.92610800	0.47167000	-3.71481400
H	-8.33560000	1.46877300	-3.50131700
C	-8.92756400	-0.60420800	-3.30957300
H	-9.82034800	-0.56205800	-3.93767800
H	-8.46999900	-1.59322900	-3.42249000
H	-9.21314700	-0.47606900	-2.25886700
O	-6.70179400	0.30705000	-2.97951100
H	-6.64951800	0.96239300	-2.26684300
C	-8.77160800	0.95078500	-6.04904800
O	-9.79875100	0.28944200	-6.16586900
N	-8.61697400	2.20882100	-6.53897300
H	-7.69790800	2.62451400	-6.46531600
C	-9.62265100	2.87528500	-7.34674900
H	-10.56462000	2.34753900	-7.17942200
C	-9.75776100	4.36605500	-6.97365000
H	-10.56344300	4.79238700	-7.58423600
H	-10.07061900	4.43867100	-5.92529400
C	-8.47730700	5.14755500	-7.19086700
C	-8.16096400	5.66485400	-8.45598100
H	-8.86744600	5.54359100	-9.27441800
C	-6.95588900	6.33204900	-8.67781100
H	-6.73271500	6.72751500	-9.66537800
C	-6.03991800	6.49104400	-7.63496300
H	-5.10002700	7.00856800	-7.81004100
C	-6.33830200	5.98624800	-6.36730300
H	-5.63227700	6.10451600	-5.54866800
C	-7.55152300	5.32432100	-6.15078200
H	-7.78317400	4.93861100	-5.16009500
C	-9.53901600	8.00641900	1.82684000
H	-9.89966100	7.87169900	2.85323700
C	-8.20533500	7.26352200	1.66550200
H	-7.77725300	7.47103800	0.67490500
H	-7.50032400	7.60969200	2.42270300
O	-8.34629000	5.85462300	1.86591300
H	-8.71028900	5.45878400	1.05358900
C	-9.33010300	9.49288500	1.55667000
O	-8.39476100	10.11205400	2.06482300
N	-10.22032600	10.06993000	0.69948000
H	-10.92923600	9.48330000	0.28180100
C	-10.04847600	11.41564700	0.16962800
H	-9.18493500	11.83257300	0.69084400
C	-9.85765900	11.43859600	-1.36057100
H	-10.81284600	11.19887200	-1.84653200
H	-9.63748600	12.47811800	-1.64468700
C	-8.78782900	10.50594500	-1.90387900
C	-9.01366800	9.80267000	-3.09584600
H	-9.95854200	9.93212300	-3.61983900
C	-8.04828800	8.94085600	-3.62287700
H	-8.24303500	8.40809000	-4.55022800
C	-6.83660800	8.75486800	-2.95306800
H	-6.09213500	8.06913100	-3.34754100
C	-6.59885000	9.44876000	-1.76326200
H	-5.66744900	9.30440500	-1.21952300
C	-7.56093000	10.32117800	-1.24956700
H	-7.36394600	10.83326800	-0.31255600
C	-10.14517200	2.54645300	0.32915000
H	-9.63113700	2.03373800	1.14944500
H	-10.60940800	1.77457300	-0.29866000
C	-9.19243900	3.39843200	-0.47771800
C	-9.60186900	4.62718200	-1.02085100
H	-10.61959900	4.97156000	-0.84692700
C	-8.73184900	5.39832800	-1.80220900
H	-9.06594300	6.34753200	-2.21259300
C	-7.43510000	4.94747400	-2.05947200
H	-6.75698100	5.53955100	-2.66770500
C	-7.00940100	3.72918300	-1.51944800
H	-6.00142600	3.36884600	-1.70339400
C	-7.87876200	2.97220600	-0.73119800
H	-7.52477100	2.03740800	-0.30232000
C	-0.04092000	-2.26657600	2.64978400
H	0.19921100	-2.42573000	3.70877400
H	0.50981300	-3.01755800	2.06876600
C	0.27167100	-0.87392900	2.21155100
N	1.51713500	-0.27659100	2.36276700
C	1.45144700	0.98687100	1.87437300
N	0.23854200	1.23481500	1.41399500

C	-0.50339400	0.08631000	1.61618400
H	-1.54352900	0.02379500	1.34055900
H	2.33531800	-0.70578900	2.77164500
H	2.27192700	1.68695900	1.87631600
C	3.21746000	3.23383400	-2.78582400
H	2.75755800	2.62458600	-2.00155700
H	3.50342800	4.19922500	-2.36193500
N	2.30603300	3.42397900	-3.90930500
H	2.31624500	2.73840200	-4.65703500
C	1.20975300	4.19999800	-3.85745600
N	0.97542700	5.02355200	-2.83584000
H	0.07907000	5.50223300	-2.85270100
H	1.39100300	4.87897700	-1.90772500
N	0.37137300	4.20050200	-4.91119200
H	-0.51531300	4.69253800	-4.82028800
H	0.55389200	3.57250700	-5.68694300
Fe	-0.44165900	3.11660400	0.51921200
O	-0.99799600	4.53971500	-0.27682000
C	-3.93027500	5.57802700	-0.51637600
C	-2.73040100	5.84560800	-1.40158200
N	-3.72895800	6.18667700	0.82903200
S	-3.02557900	5.54934700	-3.14720100
O	-4.06940300	6.57788300	-3.53161400
O	-1.71876500	5.83267500	-3.81747800
O	-3.52124500	4.15797100	-3.29678100
H	-4.09148100	4.50688400	-0.38196100
H	-4.84441500	6.01131200	-0.93426400
H	-2.31596500	6.85203000	-1.29716500
H	-1.80099200	5.08025400	-0.96717100
H	-3.68915300	7.22611100	0.75030300
H	-2.83311500	5.91154500	1.26601100
H	-4.50764700	5.88031700	1.48446800
C	1.95978100	4.79345200	0.90728800
O	1.13671900	3.85455600	1.31023700
O	2.16467400	5.10874700	-0.26409300
C	2.68681800	5.49198800	2.04514300
H	3.53017900	6.06715900	1.65809000
H	3.02949600	4.76900600	2.79166100
O	1.49424100	1.98550400	-6.46569400
H	0.83482700	1.27701700	-6.54454000
H	1.90457700	2.05038000	-7.34225000
O	-5.71619300	5.09476900	2.33071200
H	-6.66769800	5.28144900	2.16252800
H	-5.64968500	4.93683600	3.29823600
H	-1.11036300	-2.45026000	2.51722800
H	-4.56377300	-0.77446400	7.38946000
H	1.98730800	6.17418300	2.54285700
H	4.11623400	2.74004500	-3.16201300
H	5.37571100	12.79621800	1.41273400
H	-1.33399100	12.80382900	-4.91601200
H	1.40811500	14.28088300	-1.87646100
H	-4.75005200	10.84972800	3.80675600
H	-4.80986200	8.01731100	9.24455900
H	-5.51273800	-1.70882000	-0.90847000
H	-9.37595800	2.78370200	-8.41414300
H	-10.91525500	12.03558000	0.43069600
H	-10.95497600	3.14352900	0.76054200
H	-10.29257900	7.57626300	1.15480900

^{5}TS1$_{HA,C1R,C2}$

C	-0.63735600	11.29145600	-4.69086800
H	-0.53308700	10.95803700	-5.72608700
H	0.34807800	11.25255600	-4.21358200
C	-1.60978800	10.42127600	-3.96778900
N	-1.85018600	10.53579400	-2.60554200
C	-2.74939100	9.62336900	-2.21392300
H	-3.07870600	9.47681400	-1.18638600
N	-3.11169600	8.92994600	-3.29491200
H	-3.70984300	8.07586200	-3.29811200
C	-2.41502600	9.39968900	-4.39126500
H	-2.54059200	8.96524600	-5.36978300
C	2.33586100	13.26460400	-1.92265100
H	3.19009400	13.55743200	-1.29805100
H	1.69294200	14.14545700	-2.02388800
C	1.58816600	12.09749400	-1.31782000
C	0.46584600	12.29519800	-0.50084500
H	0.11749400	13.30822200	-0.30867600
C	-0.20175000	11.22393300	0.10718700
H	-1.03138800	11.39702600	0.78817000
C	0.24432700	9.90880000	-0.11484600
O	-0.38691900	8.82918500	0.41753700
H	-1.33387100	9.06416400	0.68409200
C	1.36964200	9.69224500	-0.92366200
H	1.70694000	8.67198300	-1.08469900
C	2.02488500	10.77496600	-1.50852800
H	2.89826500	10.58875500	-2.13028200
C	5.91040500	11.47531900	0.39737900
H	4.87501000	11.66856800	0.07949800
C	6.75311900	11.07751200	-0.82091000
H	6.36635600	10.17526600	-1.30863900
H	6.76603500	11.87737600	-1.57041700
H	7.79266300	10.87750500	-0.53253100
C	5.89817500	10.43323500	1.52883900
H	6.93066000	10.24480500	1.85624900
H	5.37793200	10.86271000	2.39569100
C	5.23006800	9.10404000	1.15533000
H	5.20422300	8.42302600	2.01451900
H	4.19640600	9.26225900	0.82235900
H	5.76454300	8.59114900	0.34759000
C	-5.25688500	10.15392000	3.59784600
H	-6.33552700	10.31977600	3.62624600
C	-4.83937400	9.81453200	2.15480000
H	-5.25872600	8.84959900	1.85222800
H	-5.28851200	10.55313700	1.47888100
C	-3.32584800	9.80102600	1.90375500
O	-2.54409500	10.36575200	2.68851100
O	-2.95564500	9.16731200	0.83279100
C	-4.94119000	9.00928000	4.54792200
O	-5.81901800	8.22549300	4.94487000
N	-3.63923900	8.89041600	4.91893500
H	-3.01409200	9.54244400	4.44234500
C	-3.02952600	7.65113600	5.42096400
H	-3.40882100	6.81119700	4.82126900
C	-1.51021500	7.71582000	5.21991700
H	-1.11605100	8.66843100	5.59391800
H	-1.07105700	6.92428100	5.83555200
C	-1.11380600	7.46157400	3.77031200
O	-1.65683200	6.56570800	3.11641700
N	-0.10145100	8.21855300	3.27944400
H	0.02340000	8.22581900	2.26550200

H	0.11900000	9.08999300	3.74260500
C	-3.32596500	7.25430800	6.88331500
O	-2.57237900	6.46812500	7.46640300
N	-4.45512700	7.73589300	7.45299300
H	-5.15850500	8.12563200	6.82980300
C	-4.86476300	7.27141600	8.77429700
H	-3.96356200	7.15308800	9.38178400
C	-5.65453700	5.95233700	8.72427300
O	-6.84193200	5.90325800	9.04734500
N	-4.96897100	4.87267300	8.25964100
H	-4.00600100	4.98233600	7.94241100
C	-5.63274700	3.60194300	8.02852900
H	-6.03262200	3.22695600	8.97894200
C	-6.84652300	3.69967300	7.03878800
H	-7.07785100	2.70392600	6.64757800
H	-7.70416800	4.07073500	7.60040400
C	-6.53597600	4.61806600	5.86470000
O	-5.78183600	4.25874200	4.94396500
N	-7.12662900	5.82726700	5.89814400
H	-6.79369900	6.56059700	5.27440100
H	-7.58017200	6.12387200	6.75379400
C	-4.62369000	2.59632800	7.46745400
O	-3.66128700	2.93708200	6.78222900
N	-4.90975800	1.28876500	7.74840000
H	-5.82544600	1.10939600	8.14288500
C	-4.43255500	0.24644500	6.85076500
H	-3.38242200	0.43724600	6.62264800
C	-5.30963900	0.25323200	5.58384500
O	-6.50059000	0.55442400	5.65697800
N	-4.69825200	-0.03971800	4.41251600
H	-3.71438000	-0.27585000	4.35398500
C	-5.37264100	0.18771500	3.14770100
H	-6.39669900	-0.19162500	3.23035900
C	-5.45742500	1.72025200	2.83656900
H	-6.00280600	1.86550600	1.89739300
H	-6.04781500	2.18248900	3.63146600
C	-4.12463500	2.38906900	2.71559300
N	-3.40675400	2.95291100	3.75975100
H	-3.73965200	3.09963500	4.71375300
C	-2.23073400	3.40274400	3.27171800
H	-1.50043900	3.93590000	3.85935000
N	-2.14209900	3.14789200	1.97028300
C	-3.32208100	2.52337000	1.61508800
H	-3.51465900	2.20290700	0.60559000
C	-4.61244300	-0.57998400	2.05666300
O	-3.46495400	-0.98386300	2.23729500
N	-5.30342100	-0.76581200	0.89611200
H	-6.16925000	-0.25430100	0.79032100
C	-4.64753500	-1.14730500	-0.34166000
H	-3.76722200	-1.74022000	-0.08703900
C	-4.27098300	0.09648000	-1.16956400
O	-4.86457500	1.16529900	-0.98028800
N	-3.27589700	-0.08192100	-2.06439000
H	-3.01483400	-1.02869500	-2.31900600
C	-2.83857800	0.95244600	-2.99241900
H	-3.36681500	1.86582600	-2.72175700
C	-1.31611400	1.19876900	-2.89788200
H	-0.76874900	0.25075300	-2.97771000
H	-0.99024900	1.81946400	-3.74036700
C	-0.90189500	1.91256000	-1.62071600
O	-1.76564900	2.04758900	-0.69590500
O	0.25311600	2.39929100	-1.48041600
C	-3.20482900	0.51682300	-4.42325900

O	-3.16012200	-0.66483000	-4.75373600
N	-3.52994100	1.53869100	-5.26036600
H	-3.58250900	2.47686400	-4.86882700
C	-3.99545500	1.33375300	-6.63916100
H	-4.17538100	2.34802100	-7.02032500
C	-2.97962200	0.64513000	-7.59062700
H	-3.06151600	-0.43425100	-7.43573500
C	-3.32423800	0.96175700	-9.05581900
H	-3.23322200	2.03862700	-9.25718400
H	-2.63576500	0.43935500	-9.73022100
H	-4.34003400	0.64375700	-9.29478400
C	-1.53336800	1.06981400	-7.29140400
H	-1.41782400	2.16070500	-7.34699800
H	-1.21627800	0.73221600	-6.29820500
H	-0.86429400	0.62077500	-8.03679400
C	-5.37951600	0.61674000	-6.65613200
O	-5.71858200	-0.15144900	-7.54227300
N	-6.21773500	1.00022300	-5.63746100
H	-5.76101400	1.34304100	-4.79987400
C	-7.43435400	0.24454700	-5.35147000
H	-7.27616300	-0.81106200	-5.59828300
C	-7.75683400	0.39087400	-3.83567100
H	-8.18762800	1.39039200	-3.67755500
C	-8.72291300	-0.67792100	-3.33596400
H	-9.63322500	-0.69144200	-3.93962000
H	-8.24727100	-1.66275600	-3.39992700
H	-8.98542600	-0.48880500	-2.28803700
O	-6.50652600	0.31007900	-3.13325800
H	-6.51645700	0.89740400	-2.36168000
C	-8.65593200	0.70905300	-6.17331700
O	-9.68504500	0.04049800	-6.19838200
N	-8.51463600	1.91529500	-6.78182600
H	-7.60647000	2.35744700	-6.73059200
C	-9.56793900	2.54681500	-7.55552700
H	-10.45167400	1.91256700	-7.45446000
C	-9.87193500	3.98030500	-7.06719500
H	-10.71196100	4.36445500	-7.65942700
H	-10.20646700	3.92921600	-6.02424700
C	-8.68425800	4.91293800	-7.19098200
C	-8.42860600	5.58934800	-8.39332100
H	-9.11939300	5.47692000	-9.22633500
C	-7.30607200	6.40594000	-8.53357600
H	-7.13107800	6.92510000	-9.47238500
C	-6.41167900	6.55661900	-7.47100800
H	-5.53595500	7.19109600	-7.58261300
C	-6.64955400	5.89204000	-6.26587200
H	-5.95680500	5.99578000	-5.43439400
C	-7.78153900	5.08216100	-6.12964500
H	-7.96690900	4.57626700	-5.18457800
C	-9.34400500	8.06042700	1.70238800
H	-9.74399300	7.93252000	2.71594800
C	-7.96246600	7.40426800	1.61765200
H	-7.49267900	7.64481700	0.65384300
H	-7.32278100	7.79165000	2.41099000
O	-8.01944500	5.98826400	1.81151700
H	-8.39835400	5.58131400	1.01164200
C	-9.23454300	9.54882700	1.37944100
O	-8.24755300	10.20827600	1.70317200
N	-10.29476500	10.07961600	0.70368500
H	-11.03932000	9.45710100	0.42153900
C	-10.28384300	11.42668700	0.15116700
H	-9.36927200	11.89239300	0.52274300
C	-10.33957000	11.43935300	-1.39048100

H	-11.33731600	11.11932300	-1.71850600
H	-10.24098900	12.48551500	-1.71351800
C	-9.29520400	10.57460000	-2.07356100
C	-9.67475200	9.63937000	-3.04621500
H	-10.72583700	9.54302400	-3.31062800
C	-8.72730800	8.83785400	-3.68990400
H	-9.04125400	8.12610200	-4.44895000
C	-7.37542400	8.95399600	-3.36078900
H	-6.63874000	8.32506400	-3.85300000
C	-6.98494500	9.88313700	-2.39282600
H	-5.93517500	9.98717200	-2.12896400
C	-7.93259500	10.68691400	-1.75856000
H	-7.60960500	11.39349600	-0.99910600
C	-10.02785300	2.50379700	0.52000000
H	-9.83820900	2.71062100	1.58167700
H	-9.84029500	1.43721200	0.35645100
C	-9.15664500	3.36075500	-0.36946500
C	-9.59679700	4.61741600	-0.81751800
H	-10.59639500	4.95538200	-0.55096100
C	-8.78119500	5.42679300	-1.61754400
H	-9.14192700	6.39389600	-1.95850900
C	-7.50927000	4.98756300	-1.99614600
H	-6.87201400	5.61257700	-2.61494900
C	-7.06246900	3.73411900	-1.56711900
H	-6.08526900	3.36694100	-1.86762700
C	-7.87697700	2.93734700	-0.75792200
H	-7.50593600	1.97100800	-0.42295900
C	0.08187700	-2.10773500	2.61129600
H	0.35382400	-2.25867100	3.66374500
H	0.64504500	-2.83769700	2.01569900
C	0.33308800	-0.70458200	2.16720700
N	1.56492800	-0.07025800	2.26930900
C	1.44049000	1.19213600	1.78996400
N	0.20246500	1.40573600	1.38121200
C	-0.49435000	0.23438700	1.60923000
H	-1.54308300	0.13911100	1.37982100
H	2.41141400	-0.47484700	2.64450400
H	2.24070400	1.91482600	1.76112600
C	3.11862100	3.74238300	-2.84096400
H	2.72978400	3.06296900	-2.07527000
H	3.36087800	4.69955900	-2.37319900
N	2.15558700	3.93028900	-3.92115900
H	2.19929300	3.30184100	-4.71658200
C	0.98906400	4.58340800	-3.76932000
N	0.73119200	5.32094400	-2.69146500
H	-0.22192800	5.65933400	-2.59172000
H	1.23924700	5.19772000	-1.80716300
N	0.09753100	4.54743900	-4.77841200
H	-0.82003600	4.95992000	-4.62367100
H	0.29127800	3.96785100	-5.58822100
Fe	-0.54926800	3.29064700	0.50340300
O	-1.21839700	4.77586500	-0.28058500
C	-3.64662800	6.44455200	-0.54959100
C	-3.41950300	5.15081500	-1.29633800
N	-3.26479100	6.36088500	0.88522200
S	-3.43607900	5.39706800	-3.08965100
O	-4.46128700	6.48270600	-3.31149800
O	-2.07284400	5.89028500	-3.46012000
O	-3.80481700	4.09231600	-3.68693200
H	-4.68966400	6.76698800	-0.60951000
H	-3.01672500	7.22230000	-0.97942200
H	-2.21704200	4.78441900	-0.95961500
H	-4.04844300	4.30900700	-1.00271300
H	-3.10952500	7.33643700	1.20255000
H	-2.36827200	5.85627400	0.98667900
H	-4.00580200	5.92952000	1.49845500
C	1.83178500	5.05522400	0.94952700
O	1.00410400	4.09259900	1.28325800
O	2.04734800	5.43635900	-0.20084400
C	2.55171700	5.67816500	2.13272500
H	3.34892300	6.33814900	1.78581100
H	2.96221000	4.90016700	2.78443300
O	1.36086500	2.53318700	-6.49517700
H	0.82662700	1.72512200	-6.56535600
H	1.72529700	2.67456100	-7.38301300
O	-5.36129000	5.59164800	2.48053300
H	-6.29532800	5.58634000	2.17368100
H	-5.38952900	5.17841300	3.37135200
H	-0.98295300	-2.33042900	2.50441300
H	-4.51084000	-0.72123400	7.35634900
H	1.83535600	6.25685700	2.72690600
H	4.03174800	3.32785500	-3.27339300
H	6.29013200	12.42492600	0.79779600
H	-0.96597700	12.33727700	-4.70268200
H	2.73443700	13.02141800	-2.91421500
H	-4.73991900	11.06563800	3.91452000
H	-5.51660800	8.01725200	9.23149400
H	-5.32706500	-1.76685100	-0.93843600
H	-9.28889800	2.57075000	-8.61764300
H	-11.12941000	12.00245100	0.54791600
H	-11.09139000	2.68877900	0.33780500
H	-10.04004200	7.56138700	1.01566600

^{5}TS1$_{HA,C2S,C2}$

C	-0.21102400	11.55134900	-4.78014700
H	-0.10594300	11.36010100	-5.85072000
H	0.77203600	11.43463100	-4.31077700
C	-1.19940300	10.60553800	-4.18432500
N	-1.44298100	10.53613500	-2.82035200
C	-2.36057900	9.59508800	-2.56166200
H	-2.71685700	9.32867100	-1.57024800
N	-2.72908800	9.06074600	-3.72577700
H	-3.36185900	8.23127000	-3.83568100
C	-2.02027400	9.66531200	-4.74558600
H	-2.15349100	9.37828100	-5.77636400
C	2.58555100	12.98541700	-1.61176200
H	3.05186200	12.78687200	-2.58356900
H	3.39687900	13.22372100	-0.91135600
C	1.77305000	11.80487900	-1.12853600
C	0.59507100	11.98456400	-0.38880300
H	0.25271000	12.99373100	-0.16734700
C	-0.14010200	10.89768600	0.10436200
H	-1.02237600	11.05048000	0.72105500
C	0.29579500	9.58773700	-0.16027600
O	-0.39291200	8.49053500	0.25631400
H	-1.36006000	8.71071400	0.44085400
C	1.48010500	9.38790900	-0.88519200
H	1.80994400	8.36929800	-1.07035300
C	2.19905200	10.48461700	-1.35734500
H	3.11414000	10.31224000	-1.92017300
C	5.84559000	11.54854600	1.03628900
H	4.87211400	11.62591500	0.52944800
C	6.94727900	11.31275500	-0.00414000
H	6.78119900	10.39183600	-0.57484600
H	6.99659100	12.14001100	-0.72163800

H	7.93061900	11.23186900	0.47568400
C	5.75607300	10.46378700	2.12302800
H	6.72574600	10.38671500	2.63533900
H	5.03442300	10.78816900	2.88500300
C	5.34264000	9.08138800	1.60232000
H	5.25388100	8.36270200	2.42570600
H	4.37228800	9.12748800	1.09121100
H	6.07353900	8.67701800	0.89277200
C	-5.32429700	9.86691000	3.24979200
H	-6.40496100	10.02115900	3.25859800
C	-4.90241100	9.35104800	1.85984700
H	-5.24467200	8.31932900	1.72014900
H	-5.42413400	9.93907600	1.09462900
C	-3.39812400	9.41859200	1.56182000
O	-2.63067100	10.04852900	2.31216800
O	-3.01699300	8.78197400	0.49610800
C	-4.98796300	8.85748800	4.33469600
O	-5.84697200	8.10201300	4.81836800
N	-3.68551100	8.81623300	4.72348600
H	-3.07671300	9.41748400	4.16526400
C	-3.04617000	7.64346600	5.33839600
H	-3.40484800	6.74413400	4.81715600
C	-1.53057000	7.72393300	5.12281400
H	-1.15417000	8.71616500	5.39891200
H	-1.06921800	7.00320700	5.80577000
C	-1.14961900	7.34419200	3.69882400
O	-1.70852900	6.39234300	3.14029800
N	-0.14882100	8.04722000	3.12414800
H	-0.03996200	7.96926400	2.11018500
H	0.09980500	8.94527500	3.51645100
C	-3.33403200	7.35476300	6.82690200
O	-2.54887900	6.65258700	7.47246400
N	-4.49862200	7.80814700	7.34495500
H	-5.20668300	8.11862000	6.68199000
C	-4.91505800	7.38856600	8.67873600
H	-4.02145800	7.33285800	9.30609400
C	-5.65169100	6.03778400	8.67721600
O	-6.83001800	5.95113900	9.02470900
N	-4.93534800	4.97247700	8.22504500
H	-3.97433200	5.10171800	7.91175000
C	-5.55229700	3.67059600	8.03873600
H	-5.90871100	3.29739900	9.00728800
C	-6.79780900	3.69732300	7.08482300
H	-6.99874000	2.68458100	6.72163500
H	-7.65253500	4.04521700	7.66536700
C	-6.56596300	4.59883200	5.87938000
O	-5.83525400	4.24461000	4.93846800
N	-7.20187000	5.78567700	5.90663400
H	-6.91180100	6.51284900	5.25513900
H	-7.63393200	6.08653200	6.77197900
C	-4.51793900	2.69534600	7.46899600
O	-3.57810100	3.06558900	6.76626900
N	-4.74967400	1.38196200	7.76667900
H	-5.64960700	1.17182500	8.18162200
C	-4.24348700	0.34332500	6.88012500
H	-3.20745600	0.57661200	6.62739800
C	-5.14490400	0.28639500	5.63056900
O	-6.34190000	0.55434300	5.72154500
N	-4.55008300	-0.02159200	4.45263500
H	-3.55689700	-0.20902400	4.37947000
C	-5.24596500	0.21303000	3.19940400
H	-6.27220100	-0.15258500	3.30515200
C	-5.31241200	1.74755400	2.89212600

H	-5.85951000	1.90300300	1.95657700
H	-5.88898600	2.21884500	3.69191800
C	-3.97120000	2.39854900	2.76365600
N	-3.24317400	2.95535500	3.80483400
H	-3.55790800	3.09116500	4.76736000
C	-2.07019000	3.40748300	3.30685200
H	-1.33358300	3.93205600	3.89326500
N	-1.99523900	3.17038500	2.00209800
C	-3.17446500	2.53829200	1.65853500
H	-3.37579000	2.22533800	0.64840100
C	-4.52082100	-0.54406700	2.08163600
O	-3.34928000	-0.89988600	2.20079800
N	-5.26361700	-0.75531900	0.95732000
H	-6.15646300	-0.28334700	0.90541400
C	-4.64913900	-1.05979000	-0.32240400
H	-3.77004300	-1.68072500	-0.14051300
C	-4.28226100	0.23425500	-1.07396800
O	-4.86102700	1.29152100	-0.80369400
N	-3.29988900	0.11385200	-1.99580300
H	-3.06250900	-0.81712500	-2.32338900
C	-2.93563600	1.19127100	-2.90937000
H	-3.42382400	2.09750600	-2.55352700
C	-1.41193900	1.41171600	-2.95734900
H	-0.89220000	0.46320200	-3.14080100
H	-1.17053300	2.06427600	-3.80500700
C	-0.80925500	2.07259200	-1.72591700
O	-1.60026500	2.34277400	-0.74727900
O	0.40127000	2.36726700	-1.68384100
C	-3.42323400	0.81557400	-4.31946800
O	-3.35926400	-0.34664100	-4.71483300
N	-3.86877600	1.85777500	-5.06692900
H	-3.93200500	2.77564700	-4.62664800
C	-4.40566300	1.69068100	-6.42297600
H	-4.69888800	2.70385000	-6.72926900
C	-3.39892600	1.14697200	-7.47538900
H	-3.40212600	0.05578000	-7.39957100
C	-3.84839800	1.54223500	-8.89217200
H	-3.83920300	2.63409000	-9.01780300
H	-3.17019500	1.11584700	-9.64073000
H	-4.85480400	1.17371500	-9.09647500
C	-1.96768700	1.64485700	-7.21970800
H	-1.91764400	2.74154300	-7.19443500
H	-1.57371700	1.25792200	-6.27340000
H	-1.31594400	1.30001200	-8.03316300
C	-5.72015500	0.85639500	-6.40113400
O	-6.06862300	0.14432300	-7.33064900
N	-6.50616400	1.07713300	-5.29822800
H	-6.03151100	1.39102700	-4.46026900
C	-7.67068500	0.24147300	-5.02972400
H	-7.45311500	-0.79815000	-5.29936000
C	-8.00842900	0.33614500	-3.51247100
H	-8.50710000	1.29977700	-3.34011100
C	-8.90217800	-0.80761500	-3.04702300
H	-9.79696000	-0.87690800	-3.67008100
H	-8.35514900	-1.75427700	-3.11846500
H	-9.19584100	-0.65721000	-2.00171100
O	-6.76887700	0.32423300	-2.77966900
H	-6.78414000	1.02379500	-2.10901300
C	-8.91591900	0.65301900	-5.84706300
O	-9.89399800	-0.08470400	-5.91880900
N	-8.86041900	1.89901700	-6.38422800
H	-7.97091000	2.37885300	-6.34877500
C	-9.92218300	2.46636600	-7.19577900

H	-10.83803900	1.92228300	-6.95226100
C	-10.09183900	3.97537500	-6.92399300
H	-10.93182100	4.33329300	-7.53207100
H	-10.36954300	4.11389500	-5.87233900
C	-8.84521700	4.77717500	-7.24328600
C	-8.58895400	5.20891700	-8.55320900
H	-9.31754500	5.00476200	-9.33494100
C	-7.41626700	5.89727400	-8.86570400
H	-7.24055300	6.22677200	-9.88646800
C	-6.47263900	6.16345000	-7.87040000
H	-5.55966600	6.70050700	-8.11435500
C	-6.71024300	5.74320100	-6.55975100
H	-5.98089100	5.94755900	-5.77955300
C	-7.89149300	5.05942300	-6.25263900
H	-8.07624100	4.73979900	-5.22912800
C	-9.53220800	8.02772200	1.51415600
H	-10.01563200	7.92253500	2.49335100
C	-8.17454800	7.31827100	1.54129700
H	-7.62927600	7.51671000	0.60876300
H	-7.57664800	7.70099000	2.36855400
O	-8.29801300	5.90912800	1.75972600
H	-8.64200500	5.50108500	0.94513500
C	-9.33469500	9.50900500	1.20066100
O	-8.36403700	10.13303700	1.62934100
N	-10.29247100	10.07743900	0.41358800
H	-11.02237300	9.48271100	0.04622300
C	-10.16636100	11.42130900	-0.13359700
H	-9.28654200	11.85510500	0.34454300
C	-10.03868000	11.43267400	-1.67101900
H	-11.00737700	11.16989000	-2.11615100
H	-9.84470600	12.47145500	-1.97504000
C	-8.97393900	10.50765100	-2.23429200
C	-9.27561900	9.65732900	-3.30715100
H	-10.27709600	9.67212000	-3.73262000
C	-8.31228200	8.79903800	-3.84454500
H	-8.56479400	8.15595100	-4.68399000
C	-7.02450800	8.76724800	-3.30521400
H	-6.27452200	8.09389600	-3.71053800
C	-6.71265100	9.61005200	-2.23471000
H	-5.71595200	9.59226000	-1.79879700
C	-7.67325700	10.47516000	-1.70884200
H	-7.41410500	11.10971100	-0.86615600
C	-10.01380500	2.64166300	0.66486800
H	-9.90517600	3.06203900	1.67352900
H	-9.72250500	1.58746000	0.71766500
C	-9.16941400	3.40026700	-0.33367800
C	-9.67575500	4.53350200	-0.99240200
H	-10.70525600	4.83641600	-0.81193700
C	-8.88508500	5.26208600	-1.88879700
H	-9.29304300	6.13835100	-2.38553900
C	-7.57382300	4.85950800	-2.15402300
H	-6.96078500	5.41333200	-2.85882200
C	-7.05408600	3.73299800	-1.50858900
H	-6.03621100	3.41146300	-1.70672300
C	-7.84516000	3.01952400	-0.60374100
H	-7.41983000	2.15657500	-0.09607200
C	0.12513800	-2.05976100	2.17770000
H	0.47709100	-2.29471600	3.19010500
H	0.60701000	-2.76325500	1.48676500
C	0.39747200	-0.63820400	1.81212000
N	1.65632700	-0.05097800	1.87134000
C	1.55147800	1.24367400	1.49299700
N	0.29483800	1.52142500	1.18824700
C	-0.43219800	0.36129900	1.37981100
H	-1.49628800	0.31766900	1.21943900
H	2.51085900	-0.50800400	2.15787000
H	2.36923800	1.94472200	1.45948600
C	3.18362900	3.81645500	-2.94557400
H	2.81583400	3.10854500	-2.19568600
H	3.41110100	4.76595700	-2.45521300
N	2.20571300	4.01684800	-4.00997700
H	2.23105600	3.38360200	-4.80193400
C	1.03158900	4.64880500	-3.82400900
N	0.77998800	5.35495000	-2.72340900
H	-0.17914500	5.66654100	-2.59740400
H	1.28982300	5.20285200	-1.84522500
N	0.13266400	4.63840200	-4.82855700
H	-0.80703000	4.97117900	-4.62005500
H	0.29986000	4.02751900	-5.62281700
Fe	-0.39714100	3.32961900	0.49483300
O	-0.85778700	4.83969700	-0.17153200
C	-3.25828400	5.25370000	-0.36833100
C	-3.85799100	6.12369400	-1.45258100
N	-3.37046400	5.96447000	0.93900500
S	-3.53926600	5.65542500	-3.17624800
O	-4.21530600	6.76304500	-3.94494400
O	-2.05799100	5.68810200	-3.35919400
O	-4.16077500	4.32396700	-3.40158500
H	-2.00247200	5.02654400	-0.45245700
H	-3.71809700	4.27017900	-0.29491500
H	-4.95229300	6.15833700	-1.37118200
H	-3.48943900	7.14710100	-1.35824200
H	-3.31250800	6.99724600	0.79572000
H	-2.58038600	5.74785400	1.57047700
H	-4.26068700	5.73877400	1.46325900
C	1.91783600	5.05057900	0.94308400
O	1.14873400	4.05975300	1.32818700
O	2.07430100	5.42901600	-0.21825100
C	2.67101500	5.70317900	2.09074000
H	3.42925300	6.38762600	1.70609300
H	3.13697400	4.94294300	2.72584100
O	1.23805700	2.56771200	-6.56721700
H	0.64979400	1.79572400	-6.59919400
H	1.52132500	2.70726300	-7.48444000
O	-5.63394500	5.41667200	2.38625600
H	-6.57812000	5.44675000	2.11434600
H	-5.63271700	5.08426600	3.31203400
H	-0.95340000	-2.23435100	2.14766800
H	-4.26725700	-0.61598100	7.40668300
H	1.96243600	6.25958100	2.71472200
H	4.10071300	3.43350000	-3.39845700
H	6.01629900	12.51901500	1.52156300
H	-0.52455900	12.59393500	-4.65147700
H	1.96941400	13.88561100	-1.70992900
H	-4.82288900	10.82024700	3.44616000
H	-5.60426000	8.12632700	9.09217300
H	-5.35881700	-1.62175300	-0.93938200
H	-9.71554100	2.30462900	-8.26341400
H	-11.03140500	12.02837900	0.16106000
H	-11.07681300	2.68434300	0.40638500
H	-10.19086700	7.55330000	0.77515200

^{5}TS1$_{HA,C2R,C2}$

C	0.78221000	11.16302700	-5.13990600
H	0.95390100	10.92424300	-6.19223400
H	1.75378700	11.30030800	-4.65312400
C	0.00429700	10.06956900	-4.48691800
N	-0.24537000	10.03463700	-3.12128300
C	-0.96415000	8.94861100	-2.80643900
H	-1.31832800	8.67882600	-1.80802500
N	-1.19066800	8.28149600	-3.93893900
H	-1.67420400	7.36293700	-4.00150800
C	-0.59879900	8.95110200	-4.99363400
H	-0.65503900	8.58138600	-6.00521200
C	3.11778700	13.70222500	-2.63127200
H	3.76252300	13.28767400	-3.41497900
H	3.74415700	14.36668500	-2.02180200
C	2.48824000	12.61510800	-1.78950700
C	1.17303100	12.72201100	-1.32013000
H	0.58072000	13.59630000	-1.58262400
C	0.60020300	11.74308500	-0.49636600
H	-0.41117700	11.85880000	-0.11651100
C	1.35427700	10.61677500	-0.11683200
O	0.84795100	9.64333700	0.67706900
H	-0.14894900	9.76985900	0.81576000
C	2.67070400	10.49280700	-0.58820400
H	3.25042000	9.62055600	-0.30099300
C	3.21856600	11.47714200	-1.40603000
H	4.24350500	11.36197100	-1.75242600
C	0.30899900	13.75179900	3.08412200
H	0.88044200	13.36756700	2.22712900
C	1.26354700	14.42810700	4.07569700
H	2.02026300	13.73279200	4.45633300
H	1.79473400	15.26294600	3.60427700
H	0.71639100	14.82781100	4.93884000
C	-0.52761600	12.60999400	3.68662600
H	-1.09468000	12.99680500	4.54565200
H	-1.26900200	12.28456600	2.94429500
C	0.29000200	11.38745900	4.12320300
H	-0.36097900	10.62398000	4.56770800
H	0.80559500	10.93646300	3.26562000
H	1.04585700	11.64308700	4.87393200
C	-4.16028500	9.91648700	2.58074700
H	-5.19401300	10.20377700	2.77716300
C	-4.03671800	9.48668400	1.10676900
H	-4.63426800	8.58978800	0.91571100
H	-4.46970800	10.27820600	0.48262200
C	-2.60012200	9.24203700	0.63594600
O	-1.69465500	9.97615300	1.13774500
O	-2.40720200	8.33683500	-0.23168300
C	-3.79254300	8.76982500	3.50475800
O	-4.64784500	8.07545600	4.07662800
N	-2.46017000	8.52759600	3.63990900
H	-1.85292500	9.13546600	3.09575500
C	-1.87716800	7.26997100	4.12342600
H	-2.45511100	6.43517900	3.70139600
C	-0.42918800	7.14615800	3.61900600
H	0.06427400	8.12378500	3.58219000
H	0.10989200	6.53962600	4.35635800
C	-0.31841900	6.39897900	2.29799200
O	-1.00056300	5.37320100	2.11090100
N	0.58521700	6.83325400	1.40477500
H	0.74336600	6.27548600	0.56701800
H	0.97526100	7.77188000	1.42246800
C	-1.92577600	7.01871100	5.65205500
O	-1.12937500	6.23542500	6.17450800
N	-2.91662000	7.63025000	6.34407900
H	-3.68956100	8.01515900	5.80582200
C	-3.13156400	7.32880100	7.75371700
H	-2.15879000	7.09787200	8.19510100
C	-4.11212000	6.16509700	7.96731700
O	-5.22715600	6.34632400	8.45682600
N	-3.68401700	4.94273100	7.54837300
H	-2.78098100	4.83794000	7.09025700
C	-4.55783600	3.78425900	7.61228000
H	-4.84904200	3.61523500	8.65670900
C	-5.88773600	3.95416500	6.80083100
H	-6.31078100	2.96856800	6.58043600
H	-6.58825600	4.51073400	7.42386200
C	-5.66637900	4.67071900	5.47374900
O	-5.10612800	4.10674500	4.51921800
N	-6.13628600	5.93082100	5.41568200
H	-5.90463600	6.52053300	4.62024200
H	-6.40559900	6.39543400	6.27379500
C	-3.80952500	2.55469500	7.09383600
O	-2.86935000	2.64134700	6.30355100
N	-4.29368000	1.35884500	7.54217500
H	-5.17978800	1.39340500	8.03204800
C	-4.10341000	0.15651900	6.74044000
H	-3.06723500	0.12767900	6.39845400
C	-5.10212000	0.18773800	5.56466700
O	-6.20831600	0.70690700	5.70893000
N	-4.67753700	-0.33253200	4.38993600
H	-3.75408300	-0.73599100	4.27681300
C	-5.40625800	-0.09584800	3.15518300
H	-6.46702500	-0.30817300	3.32545800
C	-5.27495900	1.40341500	2.72032000
H	-5.86294900	1.56851400	1.81157600
H	-5.71381500	2.01545800	3.51154300
C	-3.86034000	1.80790900	2.45526400
N	-2.95244100	2.22960100	3.41570300
H	-3.15560100	2.49767300	4.37937900
C	-1.74490300	2.36532300	2.82807800
H	-0.86847500	2.72776500	3.34140600
N	-1.82097500	2.04400300	1.54337700
C	-3.13621800	1.71156800	1.29747100
H	-3.47234800	1.41658800	0.31870300
C	-4.83702500	-1.04084500	2.08557300
O	-3.75903700	-1.61167000	2.24405800
N	-5.60889900	-1.17548500	0.96815000
H	-6.37752100	-0.52523800	0.85962400
C	-5.07536600	-1.70618400	-0.27149800
H	-4.18025200	-2.28212900	-0.02621200
C	-4.73897000	-0.56363100	-1.25105300
O	-5.14693800	0.58095700	-1.02087700
N	-3.98715700	-0.90434000	-2.31702900
H	-3.83045400	-1.88417100	-2.51673200
C	-3.39933100	0.06060000	-3.23345100
H	-3.63983400	1.04760900	-2.83678400
C	-1.85951300	-0.09493800	-3.29033300
H	-1.58882300	-1.15504900	-3.38895700
H	-1.45963200	0.41648500	-4.16953100
C	-1.17747400	0.49389300	-2.06508500
O	-1.83264900	0.55739900	-0.97802600
O	-0.00758000	0.96585700	-2.12892700
C	-4.02480400	-0.07403900	-4.64057200
O	-4.55554800	-1.11704500	-5.00616800
N	-3.87572200	1.05132900	-5.39462200

H	-3.53362500	1.87716700	-4.90242500	C	-10.28511800	10.18559900	-1.03268900
C	-4.58850400	1.32084900	-6.65688900	C	-10.97453500	8.97595000	-0.87466200
H	-4.18001900	2.28088900	-7.00107200	H	-11.86607900	8.94803700	-0.25049400
C	-4.36851200	0.27495700	-7.76934000	C	-10.54184300	7.81012600	-1.51093400
H	-4.96980800	-0.60210700	-7.51685600	H	-11.08836500	6.88187100	-1.37039200
C	-4.85614300	0.83357200	-9.11588400	C	-9.40507000	7.83202800	-2.32235600
H	-4.28651600	1.72690800	-9.40813400	H	-9.06947400	6.92004000	-2.80972800
H	-4.72942000	0.08613800	-9.90704100	C	-8.71678000	9.03696000	-2.49573900
H	-5.91572400	1.09592600	-9.07204000	H	-7.83423500	9.07131400	-3.13021700
C	-2.89607900	-0.15487300	-7.86499700	C	-9.15227200	10.20018400	-1.85892800
H	-2.23610800	0.71354700	-8.00932800	H	-8.60188900	11.12813800	-1.99893600
H	-2.58703300	-0.69895400	-6.96448800	C	-11.83151600	3.63594300	2.59143800
H	-2.75501600	-0.82412900	-8.72106000	H	-11.51994400	3.88927400	3.61035600
C	-6.09826900	1.54580300	-6.34323400	H	-12.21867400	2.60858500	2.61617500
O	-6.98805700	0.83683900	-6.79000200	C	-10.68796400	3.76546100	1.61102200
N	-6.32925000	2.61469500	-5.51272700	C	-10.93060200	3.96679200	0.24457900
H	-5.53492800	3.08339100	-5.08869000	H	-11.95778900	4.05342400	-0.10438300
C	-7.57500700	2.77003400	-4.77404700	C	-9.88041300	4.05362600	-0.67284000
H	-8.33597600	2.21462300	-5.33418000	H	-10.07561800	4.21160300	-1.72958800
C	-7.48455700	2.16696000	-3.35389500	C	-8.55812900	3.94128400	-0.23554300
H	-8.31541200	2.59108900	-2.78021300	H	-7.74416000	3.99987200	-0.95234700
C	-7.61954200	0.64389700	-3.36073100	C	-8.29724700	3.73652000	1.12282800
H	-8.58583100	0.34814700	-3.78303400	H	-7.26905100	3.66067700	1.46776500
H	-6.83513500	0.15613200	-3.94741400	C	-9.35509900	3.65539200	2.03716200
H	-7.57319300	0.26311900	-2.33390700	H	-9.14525000	3.48672600	3.09213100
O	-6.24292900	2.59549200	-2.77435300	C	0.04613100	-3.25480100	2.51708100
H	-5.94237000	1.91839000	-2.13792800	H	0.41320600	-3.31131800	3.54979800
C	-8.04324800	4.24467500	-4.74969300	H	0.48817600	-4.09132300	1.96086200
O	-8.71469300	4.69199100	-3.81786200	C	0.35213000	-1.93610400	1.88715100
N	-7.72498300	4.95217300	-5.85954200	N	1.63717700	-1.42102300	1.76504100
H	-7.13184000	4.50118900	-6.54490700	C	1.55990800	-0.20904400	1.16189800
C	-8.11471000	6.33488300	-6.08124000	N	0.30227700	0.08256100	0.88385500
H	-8.56025400	6.69332200	-5.15140000	C	-0.45890000	-0.98284900	1.32909200
C	-6.91985000	7.22154500	-6.48670600	H	-1.53410000	-0.99104500	1.23581200
H	-7.27263900	8.26109100	-6.50483400	H	2.48730300	-1.86863700	2.07785300
H	-6.15061600	7.14395700	-5.71043500	H	2.40579800	0.42660900	0.95259600
C	-6.33603700	6.85760400	-7.83823600	C	2.84871000	2.59380500	-3.06442500
C	-6.95486100	7.29110700	-9.02084400	H	2.51238200	2.15896000	-2.11701600
H	-7.84019600	7.92129000	-8.96162800	H	3.08806200	3.64856000	-2.91194800
C	-6.44849900	6.93187700	-10.26985700	N	1.83462400	2.46837100	-4.10333200
H	-6.94098300	7.28112600	-11.17359200	H	1.72479800	1.55122700	-4.51807400
C	-5.30836400	6.12896000	-10.35897500	C	0.72860800	3.23491200	-4.15993100
H	-4.91192500	5.84936800	-11.33141500	N	0.55110600	4.27476600	-3.34095000
C	-4.68190000	5.69361800	-9.19052400	H	-0.30100500	4.81351200	-3.49679300
H	-3.78968900	5.07443500	-9.25128500	H	0.90625500	4.26782100	-2.37627600
C	-5.18999300	6.05482900	-7.93899300	N	-0.15981200	3.01341000	-5.14638600
H	-4.69437300	5.72204200	-7.03040000	H	-1.12903300	3.25724900	-4.93452300
C	-8.20782400	8.78320200	2.59917000	H	-0.00423900	2.21509400	-5.76355600
H	-7.48390000	8.86121900	3.41745400	Fe	-0.44673500	1.89717400	-0.08888700
C	-7.69147700	7.71926700	1.61995200	O	-1.15539200	3.27749900	-0.83451400
H	-8.42407500	7.51594100	0.83052300	C	-2.61598200	5.18319300	-1.20379800
H	-6.78443900	8.09839700	1.14548500	C	-3.79306000	5.07076800	-2.14398600
O	-7.34257500	6.51081700	2.30730200	N	-2.94272800	5.65841100	0.15639000
H	-8.05786200	5.86015100	2.19878200	S	-3.23508900	4.77909200	-3.85614100
C	-8.27566100	10.13130700	1.88937000	O	-2.77369800	3.35358100	-3.93619400
O	-7.25907100	10.65471000	1.43220100	O	-4.38191100	5.08863800	-4.73215900
N	-9.51475700	10.68813700	1.77121300	O	-2.04364700	5.69740100	-4.03467300
H	-10.30440100	10.15340100	2.10608800	H	-1.83769700	5.83571600	-1.59746800
C	-9.78306600	11.80952600	0.87978900	H	-1.98137700	4.07928200	-1.06352500
H	-8.80873600	12.12904500	0.50469700	H	-4.49932900	4.26990500	-1.90253900
C	-10.72655900	11.42592600	-0.28325000	H	-4.35365400	6.01500500	-2.18760800
H	-11.73984300	11.27137200	0.11050100	H	-3.81338300	5.28394500	0.61470400
H	-10.78816900	12.29063600	-0.95703900	H	-2.98215900	6.70885000	0.12345700

H	-2.15509800	5.42928600	0.80872100
C	1.80794700	3.82351600	0.26064700
O	1.30713100	2.62482700	0.36006200
O	1.55207000	4.64289500	-0.63366600
C	2.79788400	4.16466200	1.36538500
H	3.27270800	5.12857000	1.17618400
H	3.56085400	3.38252000	1.43749400
O	0.35123500	0.53197900	-6.58080500
H	-0.49413800	0.07397400	-6.72151000
H	0.77860500	0.53203700	-7.45167900
O	-5.06733800	5.00125400	1.79165300
H	-5.83873700	5.60735400	1.87154900
H	-4.91541600	4.69792800	2.71352100
H	-1.03725600	-3.40056400	2.53920300
H	-4.28872200	-0.72040000	7.36862200
H	2.27421100	4.19791700	2.32692000
H	3.75166400	2.08002300	-3.40307300
H	-0.37545200	14.50963300	2.67843800
H	0.24677600	12.11847500	-5.09263600
H	2.35919100	14.32734300	-3.11402200
H	-3.50792400	10.77709500	2.75715200
H	-3.56100800	8.20086900	8.24998000
H	-5.80232700	-2.38464600	-0.73588100
H	-8.88909500	6.38260800	-6.85934600
H	-10.21571700	12.64513800	1.44424400
H	-12.66940200	4.28776100	2.32058100
H	-9.17260900	8.47963100	3.02335000

Model D structures: QM region only.

$^{5}Re_{DA}$

C	-5.84996766	1.42902070	2.67303459
H	-6.21396766	1.63002070	1.66203459
H	-6.57896766	1.85502070	3.37003459
C	-4.52696766	2.08902070	2.84603459
N	-3.94996766	2.40402070	4.07003459
H	-4.36896766	2.27902070	4.98903459
C	-2.70196766	2.88702070	3.85003459
H	-2.03196766	3.21002070	4.63203459
N	-2.44396766	2.89502070	2.55403459
C	-3.57396766	2.42002070	1.92103459
H	-3.59196766	2.30702070	0.84803459
C	-1.02802570	1.78497613	-2.23703630
H	-0.35102570	1.02697613	-2.63803630
H	-0.91202570	2.69397613	-2.84003630
C	-0.63002570	2.19497613	-0.84103630
O	-1.54902570	2.21197613	0.06296370
O	0.50897430	2.61897613	-0.53503630
C	0.06602451	-1.48597224	4.77903491
H	0.72902451	-1.17997224	5.59703491
H	0.46102451	-2.44397224	4.42003491
C	0.17802451	-0.45097224	3.70803491
N	1.44602451	-0.04697224	3.30903491
C	1.32202451	0.94802776	2.40303491
N	0.04602451	1.20002776	2.17303491
C	-0.67497549	0.32502776	2.97003491
H	-1.75397549	0.33902776	2.98503491
H	2.31602451	-0.38697224	3.70103491
H	2.15202451	1.50902776	2.00103491
Fe	-0.54797857	3.14201928	1.47704079
O	-0.89297857	4.62301928	0.89704079
C	-3.78700000	6.05600000	0.21100000
C	-2.73400000	6.57500000	-0.74500000
N	-3.26600000	6.10100000	1.63000000
S	-2.95300000	5.92100000	-2.45800000
O	-3.91600000	6.82000000	-3.13600000
O	-1.57800000	5.97700000	-3.01300000
O	-3.44200000	4.53000000	-2.24800000
H	-4.00600000	5.00600000	0.01500000
H	-4.71600000	6.63300000	0.22700000
H	-2.72500000	7.66800000	-0.76400000
H	-1.76000000	6.20500000	-0.41800000
H	-3.03400000	7.06500000	1.95100000
H	-2.37100000	5.56900000	1.77200000
H	-3.96100000	5.74000000	2.31300000
C	1.73202143	3.97901928	3.03904079
O	0.45102143	3.67401928	2.99504079
O	2.48502143	3.96901928	2.06804079
C	2.20902143	4.21301928	4.47304079
C	3.22702143	5.34601928	4.60404079
H	2.66202143	3.26101928	4.79404079
H	1.33502143	4.37301928	5.11004079
H	2.76002143	6.30601928	4.36104079
H	4.03902143	5.19101928	3.89104079

$^{5}TS1_{HA,C1S,DA}$

C	-5.81000000	1.47600000	2.69100000
H	-6.16500000	1.68900000	1.67900000
H	-6.53800000	1.90800000	3.38500000
C	-4.48000000	2.12300000	2.87100000
N	-3.88000000	2.38300000	4.09800000
H	-4.28100000	2.22000000	5.01800000
C	-2.63500000	2.87200000	3.87300000
H	-1.95700000	3.15900000	4.66200000
N	-2.39900000	2.93900000	2.57500000
C	-3.54700000	2.49900000	1.94500000
H	-3.58500000	2.42300000	0.87000000
C	-1.08900000	1.74800000	-2.26400000
H	-0.40500000	0.97500000	-2.62500000
H	-0.94000000	2.63900000	-2.88500000
C	-0.75800000	2.17200000	-0.85600000
O	-1.62100000	2.01000000	0.06800000
O	0.30900000	2.79200000	-0.56400000
C	0.06100000	-1.49000000	4.82200000
H	0.73300000	-1.24900000	5.65300000
H	0.43100000	-2.43700000	4.40900000
C	0.19800000	-0.39700000	3.81300000
N	1.47000000	0.06400000	3.50400000
C	1.35400000	1.08300000	2.61700000
N	0.08900000	1.29400000	2.31500000
C	-0.63700000	0.36700000	3.04200000
H	-1.71500000	0.33300000	2.98400000
H	2.32900000	-0.25100000	3.93800000
H	2.19700000	1.64200000	2.24000000
Fe	-0.62700000	3.30900000	1.33500000
O	-1.44300000	4.81100000	0.73400000
C	-3.92400000	6.23100000	0.25600000
C	-2.75000000	6.37400000	-0.69300000
N	-3.39200000	6.14200000	1.65900000
S	-2.93400000	5.82700000	-2.38600000
O	-3.79200000	6.90200000	-2.98000000
O	-1.55500000	5.82500000	-2.92700000
O	-3.57800000	4.50000000	-2.32200000
H	-4.46000000	5.29800000	0.07700000
H	-4.62500000	7.07000000	0.22600000
H	-2.26100000	7.34500000	-0.61300000
H	-1.87600000	5.49400000	-0.19600000
H	-3.07100000	7.05800000	2.03300000
H	-2.47800000	5.49800000	1.63900000
H	-4.09700000	5.78300000	2.32900000
C	2.07800000	4.19600000	2.54700000
O	0.80000000	3.99900000	2.30800000
O	2.94100000	4.25700000	1.68100000
C	2.39200000	4.23900000	4.04700000
C	3.29400000	5.40600000	4.45400000
H	2.89300000	3.28600000	4.27600000
H	1.45000000	4.24500000	4.60400000
H	2.76900000	6.35700000	4.32000000
H	4.17400000	5.42400000	3.80900000

^{5}TS1$_{HA,C1R,DA}$

C	-5.80200000	1.48900000	2.70700000
H	-6.16900000	1.70300000	1.69800000
H	-6.52200000	1.92800000	3.40600000
C	-4.46500000	2.13000000	2.86300000
N	-3.85900000	2.46600000	4.06900000
H	-4.25300000	2.34300000	4.99900000
C	-2.61700000	2.95400000	3.80400000
H	-1.92300000	3.29200000	4.55800000
N	-2.39100000	2.94500000	2.50300000
C	-3.53600000	2.44900000	1.91300000
H	-3.58000000	2.29900000	0.84700000
C	-1.08700000	1.72300000	-2.30600000
H	-0.40900000	0.93600000	-2.64600000
H	-0.93900000	2.59600000	-2.95500000
C	-0.73700000	2.20600000	-0.92000000
O	-1.67400000	2.33800000	-0.05700000
O	0.41400000	2.60000000	-0.60400000
C	0.07600000	-1.45300000	4.77100000
H	0.74000000	-1.16800000	5.59600000
H	0.46300000	-2.41000000	4.39900000
C	0.20300000	-0.40300000	3.71500000
N	1.47600000	0.00000000	3.33700000
C	1.35800000	0.98900000	2.41800000
N	0.09100000	1.24100000	2.16100000
C	-0.63700000	0.37000000	2.95500000
H	-1.71700000	0.38000000	2.95200000
H	2.33900000	-0.33300000	3.75000000
H	2.19600000	1.52300000	1.99900000
Fe	-0.51500000	3.34800000	1.34700000
O	-1.16500000	4.97100000	0.78000000
C	-3.70600000	6.45400000	0.18100000
C	-3.16200000	5.34600000	-0.69500000
N	-3.27500000	6.23000000	1.60700000
S	-3.02300000	5.71700000	-2.45400000
O	-3.34200000	7.15000000	-2.64000000
O	-1.63400000	5.35100000	-2.83800000
O	-4.04300000	4.79600000	-3.02300000
H	-4.79800000	6.52400000	0.18700000
H	-3.30000000	7.41800000	-0.12400000
H	-1.92300000	5.13000000	-0.20100000
H	-3.60300000	4.36600000	-0.51800000
H	-3.10700000	7.12700000	2.10600000
H	-2.29300000	5.71500000	1.62000000
H	-3.96100000	5.70700000	2.18000000
C	1.41000000	4.10200000	2.77500000
O	0.30000000	3.81600000	3.32900000
O	1.52600000	3.94300000	1.51600000
C	2.61600000	4.50900000	3.60000000
C	2.26600000	5.39300000	4.79800000
H	3.33900000	4.98400000	2.93000000
H	3.07600000	3.56900000	3.94200000
H	1.42500000	4.95800000	5.34300000
H	1.96200000	6.38500000	4.45500000

^{5}TS1$_{HA,C2S,DA}$

C	-5.81900000	1.44600000	2.66400000
H	-6.17700000	1.64600000	1.65000000
H	-6.54500000	1.88700000	3.35500000
C	-4.48800000	2.09100000	2.83500000
N	-3.92200000	2.44400000	4.05400000
H	-4.34700000	2.34100000	4.97300000
C	-2.66900000	2.91600000	3.82800000
H	-2.00300000	3.25900000	4.60500000
N	-2.39900000	2.88400000	2.53400000
C	-3.52400000	2.38600000	1.91000000
H	-3.53900000	2.24700000	0.84000000
C	-1.03600000	1.75500000	-2.27300000
H	-0.37600000	1.00600000	-2.71700000
H	-0.93500000	2.68200000	-2.85300000
C	-0.58400000	2.13500000	-0.88800000
O	-1.50000000	2.23600000	0.02400000
O	0.58400000	2.45900000	-0.59700000
C	0.06000000	-1.51400000	4.77200000
H	0.72400000	-1.19100000	5.58300000
H	0.45200000	-2.48300000	4.43700000
C	0.17600000	-0.50800000	3.67500000
N	1.44400000	-0.15200000	3.23400000
C	1.32800000	0.83500000	2.31900000
N	0.05500000	1.12900000	2.12600000
C	-0.67300000	0.28900000	2.95400000
H	-1.75000000	0.34100000	3.00100000
H	2.31200000	-0.51500000	3.60600000
H	2.16200000	1.37300000	1.89500000
Fe	-0.47900000	3.06300000	1.44800000
O	-0.72200000	4.59700000	0.81600000
C	-2.95800000	5.51000000	0.46400000
C	-3.26300000	6.52700000	-0.59900000
N	-3.13700000	6.08200000	1.87000000
S	-2.99900000	5.80500000	-2.28600000
O	-3.68400000	6.76200000	-3.18400000
O	-1.52900000	5.73400000	-2.49700000
O	-3.64000000	4.46800000	-2.19700000
H	-1.74800000	5.04400000	0.51900000
H	-3.62800000	4.65400000	0.42600000
H	-4.31500000	6.84300000	-0.56300000
H	-2.62900000	7.41500000	-0.50000000
H	-2.94800000	7.10200000	1.94700000
H	-2.43900000	5.67200000	2.52200000
H	-4.06500000	5.92200000	2.31300000
C	1.83500000	3.83300000	2.98700000
O	0.54200000	3.58400000	2.94200000
O	2.59600000	3.73600000	2.02700000
C	2.30900000	4.17900000	4.39700000
C	3.07300000	5.50400000	4.44300000
H	2.96300000	3.36100000	4.73000000
H	1.44100000	4.19200000	5.06300000
H	2.48800000	6.29800000	3.97200000
H	4.00800000	5.42200000	3.88400000

^{5}TS1$_{HA,C2R,DA}$

C	-5.76800000	1.43400000	2.63100000
H	-6.12700000	1.63800000	1.61800000
H	-6.48900000	1.88100000	3.32400000
C	-4.42900000	2.06700000	2.79700000
N	-3.83300000	2.36000000	4.01800000
H	-4.24600000	2.23800000	4.94000000
C	-2.57300000	2.80700000	3.78800000
H	-1.88800000	3.10500000	4.56800000
N	-2.32600000	2.81500000	2.49000000
C	-3.47800000	2.38200000	1.86500000
H	-3.51400000	2.28400000	0.79100000
C	-1.05000000	1.63200000	-2.32300000
H	-0.38800000	0.88600000	-2.77000000
H	-0.94400000	2.56200000	-2.89500000
C	-0.62200000	1.99500000	-0.92400000
O	-1.49800000	1.93000000	0.01500000
O	0.50900000	2.46900000	-0.64300000
C	0.07600000	-1.58100000	4.80500000
H	0.73500000	-1.27100000	5.62400000
H	0.45600000	-2.55500000	4.47200000
C	0.22300000	-0.57100000	3.71700000
N	1.50300000	-0.18500000	3.34500000
C	1.41000000	0.78800000	2.41100000
N	0.14300000	1.04200000	2.14000000
C	-0.60500000	0.19100000	2.93600000
H	-1.68400000	0.21100000	2.91800000
H	2.36000000	-0.52200000	3.76600000
H	2.25600000	1.33100000	2.01700000
Fe	-0.45800000	2.98300000	1.34900000
O	-0.89200000	4.44600000	0.69500000
C	-2.40300000	6.29100000	0.35900000
C	-3.49400000	6.46800000	-0.65900000
N	-2.95100000	6.27800000	1.78600000
S	-2.97800000	5.82900000	-2.32900000
O	-3.84300000	6.57300000	-3.27400000
O	-1.53300000	6.14400000	-2.47400000
O	-3.25000000	4.37300000	-2.24400000
H	-1.68200000	7.10900000	0.37800000
H	-1.72700000	5.26900000	0.35900000
H	-4.39000000	5.89700000	-0.38000000
H	-3.77300000	7.52100000	-0.76500000
H	-3.08700000	7.24000000	2.16200000
H	-2.24100000	5.85900000	2.41800000
H	-3.83800000	5.76700000	1.95000000
C	1.96000000	3.82200000	2.81400000
O	0.66400000	3.59800000	2.74100000
O	2.74200000	3.71500000	1.87300000
C	2.40900000	4.16500000	4.23500000
C	3.01700000	5.56900000	4.32300000
H	3.15800000	3.41900000	4.53400000
H	1.55100000	4.06100000	4.90600000
H	2.35100000	6.30000000	3.85700000
H	3.96400000	5.60700000	3.78300000

^{5}IM1$_{HA,C1S,DA}$

C	-5.75600000	1.48100000	2.63600000
H	-6.10200000	1.68700000	1.61900000
H	-6.47000000	1.95500000	3.31800000
C	-4.40300000	2.08500000	2.80700000
N	-3.80100000	2.32200000	4.03700000
H	-4.21200000	2.16700000	4.95500000
C	-2.54300000	2.77800000	3.81900000
H	-1.86000000	3.03600000	4.61400000
N	-2.30000000	2.85000000	2.52300000
C	-3.45600000	2.44200000	1.88600000
H	-3.50000000	2.39000000	0.80900000
C	-1.14500000	1.66500000	-2.29300000
H	-0.51700000	0.88100000	-2.72600000
H	-0.99600000	2.57500000	-2.88300000
C	-0.71600000	2.00700000	-0.89300000
O	-1.43100000	1.67500000	0.10700000
O	0.30400000	2.73400000	-0.67900000
C	0.00800000	-1.63500000	4.84900000
H	0.70400000	-1.49200000	5.68100000
H	0.31400000	-2.57300000	4.37000000
C	0.19900000	-0.48500000	3.92000000
N	1.46800000	0.06300000	3.79500000
C	1.40500000	1.09600000	2.92400000
N	0.18000000	1.23300000	2.45500000
C	-0.57500000	0.24200000	3.05300000
H	-1.63000000	0.14500000	2.84500000
H	2.28300000	-0.21700000	4.32600000
H	2.24000000	1.73000000	2.68000000
Fe	-0.47300000	3.12300000	1.28300000
O	-1.37700000	4.62600000	0.60300000
C	-4.21100000	6.26700000	0.37300000
C	-3.50000000	6.76800000	-0.82400000
N	-3.27700000	6.01600000	1.54700000
S	-2.98600000	5.74200000	-2.15600000
O	-3.36700000	6.51700000	-3.36000000
O	-1.48700000	5.62700000	-2.02300000
O	-3.67700000	4.43900000	-2.01400000
H	-4.68100000	5.30100000	0.17700000
H	-4.96300000	6.97100000	0.75200000
H	-3.14100000	7.79200000	-0.83500000
H	-1.24000000	4.93600000	-0.32800000
H	-2.89800000	6.90400000	1.93500000
H	-2.40100000	5.38100000	1.27200000
H	-3.82400000	5.58900000	2.31700000
C	2.06100000	4.62200000	2.32100000
O	1.09300000	3.76000000	2.05600000
O	2.61900000	5.30700000	1.48100000
C	2.47100000	4.50800000	3.79600000
C	3.27500000	5.66300000	4.38300000
H	3.05800000	3.57800000	3.85900000
H	1.55900000	4.31500000	4.37500000
H	2.67700000	6.57700000	4.42300000
H	4.13800000	5.87800000	3.75000000

^{5}IM1$_{HA,C1R,DA}$

C	-5.74900000	1.48300000	2.63600000
H	-6.11100000	1.68900000	1.62400000
H	-6.46100000	1.94300000	3.33000000
C	-4.39900000	2.09600000	2.78800000
N	-3.77600000	2.37000000	4.00200000
H	-4.17500000	2.24300000	4.93000000
C	-2.51000000	2.79300000	3.75000000
H	-1.80200000	3.07900000	4.51300000
N	-2.28700000	2.80500000	2.45000000
C	-3.45600000	2.39700000	1.84400000
H	-3.51300000	2.30100000	0.77200000
C	-1.12800000	1.61300000	-2.32700000
H	-0.48400000	0.81600000	-2.71200000
H	-0.97800000	2.49200000	-2.96200000
C	-0.71600000	2.03000000	-0.93800000
O	-1.46800000	1.78300000	0.06000000
O	0.32600000	2.72000000	-0.73800000
C	0.12200000	-1.53200000	4.77700000
H	0.79600000	-1.32500000	5.61500000
H	0.45800000	-2.49000000	4.36200000
C	0.32400000	-0.43800000	3.78000000
N	1.60800000	0.07000000	3.62800000
C	1.57800000	1.03100000	2.67500000
N	0.36300000	1.16500000	2.18500000
C	-0.42300000	0.24300000	2.85300000
H	-1.47900000	0.15900000	2.64600000
H	2.41200000	-0.18400000	4.18900000
H	2.43800000	1.61900000	2.39700000
Fe	-0.43800000	3.15400000	1.28800000
O	-1.28400000	4.69700000	0.66000000
C	-3.52200000	7.12800000	0.37600000
C	-4.10200000	6.38100000	-0.75700000
N	-3.16700000	6.22800000	1.55300000
S	-3.18600000	5.77100000	-2.12300000
O	-3.61400000	6.65800000	-3.24000000
O	-1.71700000	5.89500000	-1.84100000
O	-3.63100000	4.36900000	-2.28400000
H	-4.19700000	7.88300000	0.79500000
H	-2.57200000	7.59600000	0.11700000
H	-1.27900000	4.98100000	-0.28400000
H	-5.17100000	6.19400000	-0.77100000
H	-2.72200000	6.81600000	2.29400000
H	-2.43000000	5.52300000	1.32300000
H	-3.98700000	5.78000000	2.00300000
C	1.42400000	4.12200000	2.67000000
O	0.33700000	3.74600000	3.21900000
O	1.57200000	3.95900000	1.41700000
C	2.55200000	4.70000000	3.50200000
C	2.46100000	4.33600000	4.98700000
H	2.54500000	5.78900000	3.36200000
H	3.48800000	4.35600000	3.04400000
H	2.30400000	3.25900000	5.10900000
H	1.60200000	4.82800000	5.44800000

^{5}IM1$_{HA,C2S,DA}$

C	-5.92400000	1.31000000	2.54500000
H	-6.31300000	1.45400000	1.53300000
H	-6.65500000	1.74000000	3.23700000
C	-4.62200000	2.01800000	2.67600000
N	-4.08100000	2.41700000	3.89300000
H	-4.51200000	2.33000000	4.81000000
C	-2.84700000	2.93300000	3.66900000
H	-2.19500000	3.31500000	4.44100000
N	-2.56500000	2.87800000	2.38000000
C	-3.65900000	2.32600000	1.75300000
H	-3.65100000	2.17100000	0.68400000
C	-1.01200000	1.61800000	-2.32300000
H	-0.38600000	0.78600000	-2.65400000
H	-0.87400000	2.42500000	-3.05000000
C	-0.46800000	2.19600000	-1.02500000
O	-1.36000000	2.39100000	-0.07800000
O	0.70600000	2.54000000	-0.91600000
C	0.09000000	-1.45900000	4.68400000
H	0.76500000	-1.15900000	5.49600000
H	0.48100000	-2.41400000	4.31200000
C	0.18000000	-0.41500000	3.61700000
N	1.43800000	-0.03500000	3.16400000
C	1.29800000	0.97700000	2.27800000
N	0.01900000	1.26500000	2.11600000
C	-0.68700000	0.39400000	2.93300000
H	-1.76300000	0.43400000	3.00000000
H	2.31600000	-0.40000000	3.51100000
H	2.11500000	1.52900000	1.84300000
Fe	-0.65700000	3.17300000	1.47500000
O	-0.97000000	4.84800000	0.83500000
C	-3.61500000	7.09500000	0.44400000
C	-4.26300000	6.38600000	-0.62700000
N	-3.03300000	6.32800000	1.54700000
S	-3.09400000	5.81000000	-2.08600000
O	-3.50900000	6.69600000	-3.19700000
O	-1.66100000	6.00200000	-1.69600000
O	-3.47100000	4.39000000	-2.24500000
H	-3.19800000	8.09100000	0.37200000
H	-1.09400000	4.96700000	-0.13200000
H	-4.73700000	5.46200000	-0.27800000
H	-4.99000000	7.01400000	-1.14200000
H	-2.62800000	7.00100000	2.23700000
H	-2.23100000	5.69400000	1.36300000
H	-3.75400000	5.80500000	2.08600000
C	1.35500000	3.94300000	3.09600000
O	0.06300000	3.72100000	3.15200000
O	2.01500000	3.83200000	2.05600000
C	1.99000000	4.25600000	4.44500000
C	2.89000000	5.49500000	4.40600000
H	2.58000000	3.37500000	4.73700000
H	1.19100000	4.35500000	5.18500000
H	2.37600000	6.31900000	3.90400000
H	3.79600000	5.29300000	3.83100000

^{5}IM1$_{HA,C2R,DA}$

C	-5.93500000	1.35100000	2.61600000
H	-6.32100000	1.51200000	1.60500000
H	-6.66000000	1.78300000	3.31300000
C	-4.62300000	2.03900000	2.75400000
N	-4.07000000	2.41500000	3.97300000
H	-4.49900000	2.32100000	4.89000000
C	-2.83300000	2.92500000	3.74600000
H	-2.17200000	3.29100000	4.51700000
N	-2.56000000	2.88500000	2.45400000
C	-3.66400000	2.35200000	1.82700000
H	-3.66600000	2.21100000	0.75600000
C	-1.04000000	1.69000000	-2.28700000
H	-0.42300000	0.88800000	-2.69700000
H	-0.91600000	2.56100000	-2.94200000
C	-0.48500000	2.15800000	-0.95600000
O	-1.38600000	2.38500000	-0.02500000
O	0.70700000	2.40700000	-0.80000000
C	0.07100000	-1.47000000	4.72200000
H	0.74100000	-1.15800000	5.53400000
H	0.46700000	-2.42800000	4.36400000
C	0.16500000	-0.43900000	3.64400000
N	1.42200000	-0.07000000	3.18000000
C	1.28300000	0.94000000	2.29100000
N	0.00500000	1.23700000	2.14000000
C	-0.70100000	0.37500000	2.96600000
H	-1.77700000	0.42300000	3.04000000
H	2.30000000	-0.44000000	3.52200000
H	2.09900000	1.48700000	1.84800000
Fe	-0.64700000	3.14600000	1.52000000
O	-0.92800000	4.82100000	0.86900000
C	-4.10000000	6.13600000	0.45200000
C	-3.76900000	6.81800000	-0.78200000
N	-3.14300000	6.09200000	1.55600000
S	-2.94600000	5.74800000	-2.15000000
O	-3.16500000	6.60300000	-3.33700000
O	-1.49200000	5.58900000	-1.82000000
O	-3.69700000	4.47700000	-2.14800000
H	-1.06500000	4.90200000	-0.10500000
H	-4.82100000	5.33200000	0.51400000
H	-4.68400000	7.13700000	-1.28400000
H	-3.11600000	7.68200000	-0.63000000
H	-2.84300000	7.03800000	1.89100000
H	-2.24800000	5.58100000	1.42400000
H	-3.60200000	5.68100000	2.38600000
C	1.39600000	3.92200000	3.13200000
O	0.10600000	3.67600000	3.18300000
O	2.06900000	3.82200000	2.10100000
C	2.00600000	4.25500000	4.48800000
C	2.88300000	5.51000000	4.45200000
H	2.61000000	3.38700000	4.79300000
H	1.19600000	4.34600000	5.21700000
H	2.34000000	6.33700000	3.98700000
H	3.77500000	5.33500000	3.84700000

^{5}TS2$_{reb,C1S,DA}$

C	-5.89300000	1.44900000	2.64700000
H	-6.26500000	1.63600000	1.63600000
H	-6.61500000	1.89200000	3.34100000
C	-4.57100000	2.12100000	2.79200000
N	-3.93100000	2.34600000	4.00700000
H	-4.29000000	2.12700000	4.93500000
C	-2.72500000	2.90800000	3.76100000
H	-2.02400000	3.17600000	4.53700000
N	-2.54800000	3.06200000	2.46000000
C	-3.69600000	2.59000000	1.85100000
H	-3.77200000	2.56400000	0.77400000
C	-1.24000000	1.66100000	-2.29800000
H	-0.56600000	0.86600000	-2.63200000
H	-1.08300000	2.52900000	-2.94700000
C	-0.90400000	2.13700000	-0.91400000
O	-1.71800000	1.94600000	0.04800000
O	0.13100000	2.83600000	-0.68500000
C	-0.04400000	-1.54900000	4.83300000
H	0.65100000	-1.35900000	5.65700000
H	0.29500000	-2.48800000	4.37500000
C	0.08900000	-0.41800000	3.86900000
N	1.34900000	0.10700000	3.63000000
C	1.22200000	1.13300000	2.75000000
N	-0.03300000	1.28500000	2.38200000
C	-0.74500000	0.31600000	3.06300000
H	-1.81600000	0.23200000	2.95100000
H	2.19900000	-0.17000000	4.10400000
H	2.04900000	1.74100000	2.42700000
Fe	-0.75100000	3.35200000	1.21500000
O	-1.55800000	4.91200000	0.41700000
C	-4.03500000	6.09300000	0.41900000
C	-2.89600000	6.30500000	-0.49600000
N	-3.49700000	6.12600000	1.83600000
S	-2.83900000	5.65300000	-2.14000000
O	-3.42000000	6.68900000	-3.02800000
O	-1.35600000	5.48300000	-2.33200000
O	-3.60100000	4.38100000	-2.12200000
H	-4.48800000	5.11000000	0.28500000
H	-4.81900000	6.86500000	0.40500000
H	-2.34500000	7.23200000	-0.36300000
H	-1.11100000	5.09200000	-0.44100000
H	-3.22700000	7.08400000	2.13300000
H	-2.53500000	5.55800000	1.86400000
H	-4.16900000	5.75800000	2.53600000
C	1.92100000	4.55900000	2.29300000
O	0.76900000	3.94400000	2.10400000
O	2.59800000	5.04400000	1.39900000
C	2.35300000	4.44300000	3.76300000
C	3.23400000	5.56600000	4.30400000
H	2.89200000	3.48400000	3.83100000
H	1.44700000	4.31700000	4.36500000
H	2.68900000	6.51600000	4.30400000
H	4.10600000	5.69700000	3.66200000

^{5}TS2$_{reb,C1R,DA}$

C	-5.99700000	1.40800000	2.59500000
H	-6.40100000	1.55300000	1.59000000
H	-6.72100000	1.83200000	3.29900000
C	-4.70900000	2.14600000	2.70600000
N	-4.11000000	2.49200000	3.90700000
H	-4.46500000	2.30800000	4.84300000
C	-2.93800000	3.12100000	3.64000000
H	-2.26400000	3.47500000	4.40600000
N	-2.74400000	3.20600000	2.33300000
C	-3.84300000	2.60600000	1.74800000
H	-3.92800000	2.50900000	0.67500000
C	-0.94600000	1.21400000	-2.54600000
H	-0.56900000	0.35600000	-3.10500000
H	-0.73800000	2.13200000	-3.10500000
C	-0.26000000	1.32500000	-1.18700000
O	-0.41000000	2.51700000	-0.65400000
O	0.36800000	0.39200000	-0.69900000
C	0.00400000	-1.28900000	4.68200000
H	0.70400000	-1.04600000	5.49300000
H	0.39000000	-2.20700000	4.22300000
C	0.03200000	-0.16400000	3.69800000
N	1.25200000	0.28400000	3.21500000
C	1.03300000	1.33100000	2.38300000
N	-0.25700000	1.59000000	2.29300000
C	-0.88700000	0.65700000	3.09900000
H	-1.96300000	0.64000000	3.20400000
H	2.15800000	-0.05400000	3.51200000
H	1.81900000	1.87600000	1.88300000
Fe	-0.85500000	3.40500000	1.20200000
O	-1.67200000	4.35000000	-0.05600000
C	-2.74200000	6.49400000	0.31500000
C	-2.78000000	5.17100000	-0.41600000
N	-2.96500000	6.32500000	1.81000000
S	-2.74800000	5.47900000	-2.28400000
O	-2.78200000	6.94500000	-2.47900000
O	-1.44500000	4.84200000	-2.63000000
O	-3.93500000	4.76300000	-2.78300000
H	-3.51900000	7.17600000	-0.02500000
H	-1.76600000	6.97100000	0.23300000
H	-1.27900000	3.90900000	-0.90300000
H	-3.72400000	4.65700000	-0.19400000
H	-2.86600000	7.23900000	2.30600000
H	-2.25400000	5.71300000	2.26200000
H	-3.88500000	5.93500000	2.09400000
C	1.87600000	4.43000000	1.87000000
O	0.56800000	4.50400000	1.93300000
O	2.49000000	3.94300000	0.92000000
C	2.62600000	5.00800000	3.07700000
C	2.31900000	4.30800000	4.41300000
H	2.37400000	6.07000000	3.16400000
H	3.69200000	4.94000000	2.84000000
H	2.40000000	3.22000000	4.31200000
H	1.29600000	4.51400000	4.74100000

^{5}TS2$_{reb,C2S,DA}$

C	-5.99900000	1.27600000	2.54600000
H	-6.39500000	1.41900000	1.53700000
H	-6.71200000	1.72800000	3.24200000
C	-4.68000000	1.95500000	2.65900000
N	-4.12400000	2.36900000	3.86300000
H	-4.54800000	2.30200000	4.78500000
C	-2.87600000	2.84700000	3.62000000
H	-2.21000000	3.22300000	4.38300000
N	-2.60200000	2.75700000	2.33200000
C	-3.71200000	2.21600000	1.72500000
H	-3.70700000	2.02000000	0.66200000
C	-1.13200000	1.42400000	-2.43200000
H	-0.56200000	0.60400000	-2.87300000
H	-0.98600000	2.30500000	-3.06900000
C	-0.53400000	1.84900000	-1.10600000
O	-1.41600000	2.18700000	-0.19100000
O	0.67600000	1.98800000	-0.95500000
C	0.00900000	-1.54200000	4.72200000
H	0.68200000	-1.23200000	5.53100000
H	0.38900000	-2.51200000	4.37600000
C	0.11800000	-0.52400000	3.63600000
N	1.37900000	-0.13700000	3.20300000
C	1.24200000	0.85000000	2.28800000
N	-0.03600000	1.11400000	2.09100000
C	-0.74500000	0.25400000	2.91300000
H	-1.82400000	0.28000000	2.95600000
H	2.25400000	-0.47900000	3.58100000
H	2.06100000	1.40700000	1.86100000
Fe	-0.70000000	3.00500000	1.35600000
O	-1.12500000	4.64800000	0.60700000
C	-2.58100000	6.20200000	0.39800000
C	-3.59100000	5.68200000	-0.53900000
N	-2.88200000	6.13800000	1.83700000
S	-2.91900000	5.58000000	-2.26200000
O	-2.76400000	6.98300000	-2.69300000
O	-1.60400000	4.86300000	-2.13400000
O	-3.94400000	4.81300000	-2.99100000
H	-1.95600000	7.05800000	0.18700000
H	-1.17300000	4.57800000	-0.38100000
H	-3.91500000	4.68300000	-0.22300000
H	-4.47800000	6.33200000	-0.60700000
H	-2.93500000	7.09800000	2.24700000
H	-2.09400000	5.70300000	2.35500000
H	-3.75600000	5.64900000	2.10800000
C	1.34700000	3.87800000	2.95000000
O	0.05500000	3.65500000	2.99900000
O	2.02100000	3.75000000	1.92100000
C	1.96700000	4.21500000	4.30500000
C	2.79300000	5.50700000	4.28600000
H	2.61000000	3.36900000	4.58600000
H	1.16300000	4.25900000	5.04500000
H	2.22900000	6.30800000	3.80000000
H	3.70600000	5.36800000	3.70300000

^{5}TS2$_{reb,C2R,DA}$

C	-6.00800000	1.31000000	2.59700000
H	-6.39200000	1.46500000	1.58500000
H	-6.73000000	1.75300000	3.29100000
C	-4.69000000	1.98900000	2.73200000
N	-4.15000000	2.40500000	3.94400000
H	-4.58600000	2.34000000	4.86000000
C	-2.89600000	2.87500000	3.71600000
H	-2.24100000	3.25500000	4.48600000
N	-2.60500000	2.77800000	2.43200000
C	-3.70800000	2.23900000	1.81200000
H	-3.69400000	2.05000000	0.74800000
C	-1.14200000	1.56300000	-2.35600000
H	-0.53600000	0.75100000	-2.76500000
H	-1.00900000	2.43000000	-3.01400000
C	-0.58600000	2.03400000	-1.02900000
O	-1.48400000	2.25200000	-0.09500000
O	0.60500000	2.29400000	-0.87200000
C	0.03700000	-1.48300000	4.69200000
H	0.71200000	-1.18300000	5.50300000
H	0.42200000	-2.44400000	4.32800000
C	0.13400000	-0.44700000	3.62100000
N	1.39200000	-0.03600000	3.19800000
C	1.24400000	0.96000000	2.29300000
N	-0.03700000	1.20400000	2.09100000
C	-0.73700000	0.32400000	2.90000000
H	-1.81600000	0.33200000	2.93700000
H	2.27000000	-0.37000000	3.57400000
H	2.05600000	1.53100000	1.87200000
Fe	-0.74200000	3.09900000	1.40600000
O	-1.23300000	4.73400000	0.74400000
C	-3.32700000	5.53300000	0.56600000
C	-3.47500000	6.46900000	-0.56100000
N	-3.14100000	6.06900000	1.91500000
S	-2.93000000	5.67100000	-2.14400000
O	-3.26900000	6.66600000	-3.18100000
O	-1.45200000	5.44400000	-2.00100000
O	-3.71400000	4.41700000	-2.20500000
H	-1.17400000	4.84400000	-0.23800000
H	-3.81600000	4.57300000	0.59100000
H	-4.52200000	6.76100000	-0.73500000
H	-2.88500000	7.38000000	-0.40800000
H	-2.98100000	7.09700000	1.96300000
H	-2.27900000	5.68900000	2.35400000
H	-3.93900000	5.87300000	2.55600000
C	1.34300000	3.98400000	2.95200000
O	0.06300000	3.69400000	3.02500000
O	1.99800000	3.93800000	1.90600000
C	1.96800000	4.28100000	4.31400000
C	2.81700000	5.55600000	4.32900000
H	2.59300000	3.41500000	4.57600000
H	1.16300000	4.31900000	5.05400000
H	2.26700000	6.38000000	3.86500000
H	3.72800000	5.41700000	3.74500000

^{5}Prod$_{hydro,C1S,DA}$

C	-5.95100000	1.25900000	2.46300000
H	-6.28900000	1.36600000	1.42900000
H	-6.70600000	1.73000000	3.10000000
C	-4.65800000	1.98000000	2.63200000
N	-4.15900000	2.34900000	3.87300000
H	-4.58500000	2.18000000	4.78100000
C	-2.97300000	2.97400000	3.69500000
H	-2.36400000	3.33500000	4.51000000
N	-2.67100000	3.03000000	2.40600000
C	-3.71500000	2.41500000	1.73600000
H	-3.68000000	2.30400000	0.66200000
C	-1.17000000	1.23700000	-2.16800000
H	-0.67300000	0.38800000	-1.69000000
H	-0.77700000	1.37600000	-3.17600000
C	-0.90700000	2.48200000	-1.34100000
O	-1.23300000	2.37000000	-0.08600000
O	-0.47500000	3.51000000	-1.87300000
C	-0.05500000	-1.51100000	4.75400000
H	0.67000000	-1.40800000	5.56800000
H	0.25400000	-2.40400000	4.19600000
C	0.05500000	-0.29400000	3.89900000
N	1.31000000	0.19700000	3.57500000
C	1.15300000	1.32400000	2.83900000
N	-0.12500000	1.58500000	2.65200000
C	-0.81700000	0.57400000	3.29400000
H	-1.89700000	0.54000000	3.28500000
H	2.19000000	-0.16700000	3.91800000
H	1.96900000	1.91800000	2.46800000
Fe	-0.77000000	3.39400000	1.51700000
O	-1.37900000	5.23900000	-0.14200000
C	-3.62500000	5.91300000	0.29500000
C	-2.39100000	6.12400000	-0.56300000
N	-3.25400000	6.02000000	1.76000000
S	-2.85200000	5.90500000	-2.34600000
O	-3.76300000	7.03100000	-2.66100000
O	-1.52300000	5.99500000	-2.99000000
O	-3.49600000	4.56700000	-2.37600000
H	-4.03200000	4.91000000	0.16900000
H	-4.39200000	6.66700000	0.11600000
H	-2.06600000	7.16800000	-0.44800000
H	-0.98500000	4.70700000	-0.91500000
H	-2.96000000	6.98000000	2.03400000
H	-2.45500000	5.39500000	2.00700000
H	-4.04500000	5.75500000	2.38000000
C	2.06200000	4.65600000	2.04900000
O	0.96300000	4.10700000	1.58200000
O	2.87400000	5.22300000	1.33100000
C	2.33100000	4.40100000	3.54500000
C	3.10000000	5.51000000	4.26400000
H	2.93200000	3.48000000	3.58100000
H	1.38700000	4.16300000	4.05100000
H	2.51600000	6.43500000	4.29600000
H	4.01100000	5.73300000	3.70700000

$^5Prod_{hydro,C1R,DA}$

C	-5.65600000	1.53500000	2.49700000
H	-6.02800000	1.69100000	1.48000000
H	-6.38000000	2.00100000	3.17600000
C	-4.33000000	2.21200000	2.62200000
N	-3.73900000	2.56300000	3.83100000
H	-4.13000000	2.41600000	4.75900000
C	-2.51500000	3.09600000	3.57100000
H	-1.83200000	3.44100000	4.33200000
N	-2.28200000	3.11400000	2.26900000
C	-3.40900000	2.57500000	1.67400000
H	-3.43900000	2.43200000	0.60400000
C	-1.00400000	1.76300000	-2.51900000
H	-0.38000000	0.95300000	-2.90700000
H	-0.91800000	2.59900000	-3.22400000
C	-0.44100000	2.29700000	-1.21100000
O	-1.28700000	2.47400000	-0.23700000
O	0.75100000	2.62200000	-1.09800000
C	0.10400000	-1.48900000	4.86700000
H	0.75900000	-1.23600000	5.70700000
H	0.46700000	-2.45300000	4.48800000
C	0.28100000	-0.42700000	3.83400000
N	1.56100000	0.01700000	3.55600000
C	1.48500000	0.97300000	2.60500000
N	0.23200000	1.17100000	2.22500000
C	-0.52100000	0.29500000	2.99200000
H	-1.59900000	0.28400000	2.91900000
H	2.40100000	-0.26700000	4.04700000
H	2.34500000	1.52300000	2.25600000
Fe	-0.22600000	3.03800000	1.33300000
O	-1.62800000	6.05900000	0.03900000
C	-3.77300000	7.00500000	0.11700000
C	-2.92400000	5.89300000	-0.50900000
N	-3.56700000	6.98400000	1.63100000
S	-2.93200000	6.06400000	-2.37700000
O	-4.00400000	7.04700000	-2.69300000
O	-1.57300000	6.53600000	-2.70800000
O	-3.23300000	4.68400000	-2.80700000
H	-4.84200000	6.90100000	-0.05800000
H	-3.43900000	7.99100000	-0.20000000
H	-1.30800000	5.21800000	0.41500000
H	-3.34900000	4.91200000	-0.26900000
H	-3.50800000	7.93400000	2.04800000
H	-2.63100000	6.57400000	1.85400000
H	-4.27900000	6.45700000	2.17200000
C	1.33400000	4.36800000	2.72900000
O	0.38300000	3.81500000	3.35200000
O	1.32200000	4.40800000	1.44400000
C	2.55100000	4.87100000	3.48800000
C	2.46900000	4.63200000	5.00000000
H	2.69500000	5.93100000	3.24800000
H	3.41600000	4.36400000	3.03700000
H	2.04400000	3.64300000	5.19900000
H	1.79500000	5.35800000	5.47000000

$^5Prod_{hydro,C2S,DA}$

C	-5.82000000	1.50400000	2.62300000
H	-6.17500000	1.68100000	1.60400000
H	-6.52200000	2.00600000	3.29700000
C	-4.45800000	2.09300000	2.77300000
N	-3.88500000	2.38400000	4.00300000
H	-4.33400000	2.31100000	4.91400000
C	-2.60800000	2.78100000	3.79800000
H	-1.94300000	3.07800000	4.59600000
N	-2.31900000	2.77300000	2.50500000
C	-3.46800000	2.35900000	1.86300000
H	-3.50200000	2.26600000	0.78900000
C	-1.13200000	1.89900000	-2.10500000
H	-0.48200000	1.08100000	-2.41900000
H	-1.00100000	2.77200000	-2.75200000
C	-0.82300000	2.33000000	-0.69500000
O	-1.14200000	3.51300000	-0.32900000
O	-0.29700000	1.54500000	0.14400000
C	-0.11800000	-1.80300000	4.85400000
H	0.64700000	-2.08800000	5.57900000
H	-0.02200000	-2.53700000	4.04700000
C	0.19200000	-0.45400000	4.32100000
N	0.81800000	0.53300000	5.06500000
C	0.86700000	1.66500000	4.31500000
N	0.30100000	1.45300000	3.13900000
C	-0.11500000	0.14200000	3.12900000
H	-0.62500000	-0.27600000	2.27400000
H	1.20800000	0.42400000	5.99200000
H	1.31400000	2.59500000	4.63600000
Fe	-0.34200000	2.92900000	1.76000000
O	-1.36600000	6.02500000	-0.14400000
C	-2.56800000	6.44500000	-0.19300000
C	-3.60800000	5.71800000	-0.88300000
N	-3.43400000	6.23100000	2.06800000
S	-3.22600000	5.88900000	-2.74500000
O	-3.36300000	7.33100000	-3.00300000
O	-1.83700000	5.36500000	-2.84700000
O	-4.26800000	5.03700000	-3.33800000
H	-2.68600000	7.48800000	0.08800000
H	-1.27700000	5.05400000	-0.44200000
H	-3.56400000	4.65000000	-0.66800000
H	-4.59500000	6.13300000	-0.68300000
H	-3.31200000	7.19700000	2.42700000
H	-2.69900000	5.72400000	2.58500000
H	-4.31800000	5.90300000	2.49600000
C	2.08300000	4.50600000	2.09400000
O	0.90900000	4.12500000	2.54200000
O	2.39000000	4.53500000	0.90600000
C	3.10700000	4.80900000	3.19900000
C	2.51700000	5.29000000	4.53500000
H	3.83400000	5.51600000	2.79200000
H	3.65700000	3.86500000	3.33100000
H	1.51300000	4.87500000	4.65400000
H	2.40900000	6.38000000	4.54700000

^{5}Prod$_{hydro,C2R,DA}$

C	-5.72700000	1.44800000	2.66900000
H	-6.05900000	1.64500000	1.64600000
H	-6.46100000	1.91100000	3.33600000
C	-4.38900000	2.07600000	2.86400000
N	-3.84800000	2.40200000	4.10000000
H	-4.27400000	2.25100000	5.01100000
C	-2.60100000	2.90400000	3.90600000
H	-1.95900000	3.22800000	4.71100000
N	-2.30700000	2.93000000	2.61400000
C	-3.41600000	2.41600000	1.96200000
H	-3.38800000	2.27500000	0.89100000
C	-1.07900000	1.79200000	-2.32400000
H	-0.45200000	0.96700000	-2.67800000
H	-0.93900000	2.61900000	-3.02900000
C	-0.56500000	2.31100000	-0.99300000
O	-1.40300000	2.34200000	0.00200000
O	0.58800000	2.75200000	-0.88500000
C	0.05000000	-1.64600000	4.92800000
H	0.73300000	-1.50500000	5.77100000
H	0.36200000	-2.58300000	4.45000000
C	0.24400000	-0.49300000	4.00500000
N	1.51300000	0.04500000	3.84800000
C	1.43500000	1.08500000	2.98400000
N	0.19700000	1.23500000	2.55200000
C	-0.54900000	0.25200000	3.17200000
H	-1.61300000	0.17000000	3.00600000
H	2.33900000	-0.23200000	4.36400000
H	2.26300000	1.72900000	2.73900000
Fe	-0.39000000	3.07700000	1.53900000
O	-1.49100000	5.28300000	0.18500000
C	-2.84200000	5.43700000	0.35800000
C	-3.48500000	6.46300000	-0.55800000
N	-2.98300000	5.89600000	1.83000000
S	-3.15800000	5.96300000	-2.29800000
O	-3.68800000	7.03900000	-3.14700000
O	-1.64600000	5.86900000	-2.33400000
O	-3.81500000	4.65000000	-2.42100000
H	-1.30700000	5.44900000	-0.80500000
H	-3.39200000	4.49000000	0.36000000
H	-4.56700000	6.51100000	-0.40000000
H	-3.04500000	7.45200000	-0.38700000
H	-2.73700000	6.89500000	1.99300000
H	-2.30600000	5.36000000	2.41200000
H	-3.92300000	5.72400000	2.23800000
C	2.03300000	4.76700000	2.18900000
O	1.08200000	3.89400000	2.44700000
O	2.18000000	5.36300000	1.12900000
C	3.07300000	4.89200000	3.31600000
C	2.55300000	4.65800000	4.73800000
H	3.55800000	5.86800000	3.21500000
H	3.85000000	4.14900000	3.07600000
H	1.95300000	3.74400000	4.77300000
H	1.90100000	5.47700000	5.05600000
